# A multi-center prospective cohort study to evaluate the effect of differential pricing and health systems strengthening on access to medicines and management of hypertension and diabetes in Ghana: A study protocol

**DOI:** 10.12688/gatesopenres.12797.2

**Published:** 2018-05-03

**Authors:** Linda Meta Mobula, Stephen Sarfo, Lynda Arthur, Gilbert Burnham, Jacob Plange-Rhule, Daniel Ansong, Edith Gavor, David Ofori-Adjei

**Affiliations:** 1 Johns Hopkins School of Medicine, Baltimore, MD, 21205, USA; 2 Johns Hopkins School of Public Health, Baltimore, MD, 21205, USA; 3 Department of Medicine, Kwame Nkrumah University of Science and Technology, Kumasi, Ghana; 4 Ghana Access and Affordability Program, Accra, Ghana; 5 Ghana College of Physicians and Surgeons, Accra, Ghana; 6 Department of Child Health, School of Medical Sciences, Kwame Nkrumah University of Science & Technology, Kumasi, Ghana; 7 Ministry of Health, Accra, Ghana; 8 Department of Medicine and Therapeutics, School of Medicine and Dentistry, University of Ghana, Accra, Ghana

**Keywords:** Hypertension, Diabetes, Access, Non-communicable Diseases, Ghana, Affordability, LMIC, Differential Pricing

## Abstract

**Background:** There is evidence to suggest that the prevalence of non-communicable diseases (NCDs), in particular cardiovascular diseases and diabetes, are being recognized as forming a substantial proportion of the burden of disease among populations in Low- and Middle-Income Countries (LMICs).  Access to treatment is likely a key barrier to the control and prevention of NCD outcomes.  Differential pricing, an approach used to price drugs based on the purchasing power of patients in different socioeconomic segments, has been shown to be beneficial and leads to improved access and affordability.

**Methods:** This is a quasi-experimental study, with a pragmatic trial design, to be conducted over the course of three years. A mixed methods design will be used to evaluate the effects of health systems strengthening and differential pricing on the management of diabetes, hypertension and selected cancers in Ghana. A public private partnership was established between all sites that will receive multi-level interventions, including health systems strengthening  and access to medicines interventions.

**Study populations and sites:** Study participants will include individuals with new or previously diagnosed hypertension and diabetes (n=3,300), who present to two major referral hospitals, Komfo Anokye Teaching Hospital and Tamale Teaching Hospital, as well as three district hospitals, namely Kings Medical Centre, Agogo Presbyterian District Hospital, and Atua Government Hospital.

**Discussion:** The objective of this study aims to test approaches intended to improve access to drugs for the treatment of hypertension and diabetes, and improve disease control. Patients with these conditions will benefit from health systems strengthening interventions (education, counseling, improved management of disease), and increased access to innovative medicines via differential pricing.

Pilot programs also will facilitate health system strengthening at the participating institutions, which includes training of clinicians and updating of guidelines and production of protocols for the treatment of diabetes, hypertension and cancer.

## Introduction

United Nations Sustainable Development goal 3 calls to ensure healthy lives and promote the well-being, including reducing by one third premature mortality from non-communicable diseases through prevention and treatment ^1^. Non-communicable diseases (NCDs), in particular cardiovascular diseases (CVDs), diabetes, and cancer, form a substantial proportion of the burden of disease among populations in Low- and Middle-Income Countries (LMICs). Globally, NCDs are the cause of death of more than 36 million people annually, with 80% of deaths occurring in LMICs. Current projections indicate that by 2020 the largest increases in NCD deaths will occur in Africa ^2^. The World Health Organization (WHO) Global Action Plan for the Prevention and Control of Noncommunicable Diseases 2013–2020 (NCD Global Action Plan) was endorsed by the 66 ^th^ World Health Assembly to improve availability of essential medicines in both public and private facilities and reduce premature mortality from NCDs by 25% by the year 2025 ^3^.

Essential medicines to treat NCDs have limited availability and affordability, especially in public sector settings in LMICs ^4– 6^. Approximately 90% of individuals in LMIC purchase medicines out-of-pocket, thus leading to personal expenditures for medicines being the highest expenditure after food ^7^. As a result, medicines are unaffordable for large sections of the global population and are a major burden on government budgets.

The Access and Affordability Initiative (Initiative or AAI) brings together four major research-based biopharmaceutical companies – Merck, Sharp and Dohme Corp. (MSD), is a subsidiary of Merck & Co., Inc., Kenilworth, N.J., U.S.A., Novartis, Pfizer and Sanofi (each a Participant Company and collectively the Participant Companies) that are working with committed governments and other stakeholders, including the Bill & Melinda Gates Foundation and Johns Hopkins University. The aim of the Initiative is to better understand how within-country differential pricing of innovative medicines, as determined voluntarily and independently by each participating pharmaceutical company, coupled with health systems strengthening can affect the management of hypertension, diabetes mellitus and selected cancers in LMICs. The Bill & Melinda Gates Foundation is contributing to this important effort by providing funding to test the underlying hypothesis of the program: that differentiating the prices of different income levels within a country may significantly increase access to medicines.

Differential pricing (or DP) is an approach by which manufacturers price their medicines to reflect payer’s ability to pay ^8^. DP can be implemented among countries – for example, with lower prices offered in lower-income countries – and among different patient groups within countries, reflecting their respective abilities to pay for medicines. Through differential pricing, the prices of medicines in low access populations in LMICs are more affordable and, when coupled with needed health system improvements, has the potential to dramatically increase access to medicines for specific conditions among lower income segments of the population. This approach has already been used to increase access to vaccines, malaria and HIV treatment in many countries ^8^. Expanding the approach to cover a broader range of medicines and to include greater use of differential pricing to reach patients *within* LMICs, could substantially improve access. The WHO has called for action to implement cost-effective interventions for NCDs, focusing on common risk factors for cardiovascular disease, chronic respiratory disease, cancer, and diabetes ^9, 10^.

With this aim in mind, a public-private partnership, the Ghana Access and Affordability Program or GAAP, was established between the four Participant Companies and the Government of Ghana with the goal of undertaking a study to assess whether differential pricing can be a sustainable and measurable tool to increase access to innovative medicines. A similar study, the Philippines Access to Medicines Program, is currently underway in the Palawan Province (unpublished study, Aguedo Troy Gepte IV [Ateneo School of Government, Philippines], Anthony Rosendo Faraon, Winston Pascual and Jovito Dy [Philippines Access to Medicines Project, Philippines], Shannon Doocy [Johns Hopkins School of Public Health, USA]). Ghana, like other LMICs, is currently experiencing an epidemiologic transition characterized by a dual burden of disease, with NCDs increasingly exerting added pressure on health systems, which are already struggling to cope with infectious diseases ^11^. Recent local studies have provided data pointing to increasing rates of hypertension, diabetes mellitus and various cancers ^11– 17^. Thus the prevention and control of these NCDs present a significant health challenge in the face of a combination of factors, which include weak health systems, limited affordability and accessibility to effective and safe medicines, poverty and poor patient education.

The vicious cycle of poverty, disease and economic underdevelopment has led to the creation of significant gaps in access to medicines for NCDs in LMICs, particularly in availability, affordability and quality of products ^18, 19^. Weak supply chain infrastructure, inadequate delivery systems and lack of trained personnel at the periphery of the health system impede patient access to medicines. Geographic accessibility to health services is a problem particularly in rural areas. The Ghana Health Facility level 2 survey found that the percentage of patients taking more than one hour to travel to medicine dispensing facilities was 11.7% and 0% for the public and private sector respectively, indicating a better geographical accessibility for private dispensaries. In addition, the average transport costs to the public and private dispensary facilities comprise 0.4 and 0.1 respectively of the minimum daily salary, indicating a relatively high burden for poor people traveling to public health facilities compared to private drug dispensaries ^20^.

Procurement processes are generally planned, but there is a perception that regulations are not effectively implemented in Ghana, which prevents efficient procurement in some instances, leading to fragmented procurement processes in public health facilities, as well as frequent stock-outs of medicines. Limited human resources in some districts devoted to financial management can also potentially affect the procurement and supply of medicines to clients.

The Ghanaian National Health Insurance Scheme (NHIS) was introduced in 2003 to improve access to basic healthcare services, especially for the poor and the vulnerable ^20^. Beneficiaries of the NHIS pay an annual premium, which is subsidized by the government; this subsidy is obtained principally from a tax-based system organized through salary deductions and a National Health Insurance levy (NHIL) of 2.5% on purchases of consumer goods in registered businesses and institutions in the country. Theoretically, contributions are aligned with one’s ability to pay, but in reality this seems variable. For the informal sector, community health insurance committees are in place to identify and categorize residents into social groups to enable individuals in each group to pay in line with their ability to do so.

## Reimbursement and Payment Mechanism

Under NHIS, payments to providers (clinics, hospitals, contracted private pharmacies) are made based on claims submitted by the provider to which the insured patient belongs. These rules are generally based on the Standard Treatment Guidelines (STG) published by GNDP, although NHIA issued its own abbreviated version of the guideline that lists treatment options for certain conditions and may differ from STG in some details. The NHIA Medicines List defines which drugs (listed by INN) can be prescribed and how much is reimbursed for each drug. It is generally based on the EML but again differs in some details and includes more drugs than the EML ^20^. The NHIS Medicines List defines drugs that can be prescribed and how much is reimbursed for each drug. The NHIS Medicine List is generally based on the Essential Medicine List (EML) promulgated by the Ministry of Health. The EML comprises a list of minimum medicine needs for a basic health-care system, but includes additional drugs. Patients do not need to make any co-payments under current regulations for those purchasing products on the NHIS Medicine List. However, preliminary unpublished data from an institutional appraisal performed at six pilot sites identified the following barriers to access to safe and effective medicines for the management of hypertension and diabetes: (a) inability of low- and middle-income patients to afford out of pocket medicines that are not on the NHIS; (b) medicine shortages or stock-outs, and (c) lack of availability of medicines preferred by prescribers. Therefore, alternative strategies that could supplement the NHIS would be crucial in addressing the limited range of medications on the NHIS Medicine List, as well as reducing the cost of out-of-pocket payments for non-insured medicines.

## Protocol

### Specific objectives and aims

The overarching objective of the study is to test approaches intended to improve access to innovator medicines for underserved populations in Ghana by improving their availability and affordability. The focus of the study is patients who currently have limited or no access to innovative medicines that are not on the EML for the management of hypertension and diabetes.

The study is designed to assess

(1) the effect of differential pricing on access to and control of medicines for the treatment of hypertension and diabetes;

(2) adherence to treatment and the level of disease complications among patients with hypertension and diabetes patients;

(3) the impact of effective supply chain management on access to medicines; and

(4) the impact of health systems strengthening interventions, which includes training on clinical management and supply chain management on the outcomes of hypertension and diabetes.

Whether and to what extent to engage in differential pricing was determined independently by each participating company.

We define access as having medicines continuously available and affordable at public or private health facilities or medicine outlets that are within one hour’s walk of the population, per the World Health Organizations standard definition ^21^.

### Ethical approval

Ethical approval was obtained from the Ghana Health Services Ethical Committee (GHS-ERC: 12/07/14) and the Committee on Human Research, Publications and Ethics (CHRPE) Kwame Nkrumah University of Science and Technology, School of Medical Sciences & Komfo Anokye Teaching Hospital (CHRPE/AP/298/14).

### Study design

This is a quasi-experimental study with a pragmatic trial design, designed to examine access, health and economic outcomes for hypertension, diabetes and cancer patients. The study will follow enrolled patients, who have consented to participate at five hypertension and diabetes specialty and general clinics in Ghana.

A prospective cohort will be established to look at health, economic and access outcomes for all hypertension and diabetes patients enrolled in the study. The study will follow enrolled patients in five (5) hypertension and diabetes specialty clinics in Ghana, as they are provided with routine care.

A nested study with a quasi-experimental design will be used to evaluate the effects of health systems strengthening and differential pricing in Ghana. In-country differential pricing was used to introduce innovator medicines at all hospital sites participating in the study. These sites located in urban, semi-urban and rural settings were chosen given the socio-economic diversity of the patient population. *Innovator medicines*, which are not available on the National EML list, will be available to patients, should their doctors, in the exercise of independent professional judgement, choose to start or switch to these medicines from their previous regimens. The innovator medicines are offered at a differential price for those meeting criteria for poverty ^22^ or market price for those who do not meet criteria. All medicines are prescribed based on existing protocols provided by the National Standard Treatment Guidelines ^23^.

***Assessment of eligibility for placement in Market Price (MP) versus Differential Pricing (DP) arm.*** A set of criteria based on the Multidimensional Poverty Index (MDI), an international poverty measure tool developed by the Oxford Poverty and Human Development Initiative (OPHI), will be used to determine if a patient qualifies for differential pricing ^22^. The criteria were validated by the Ghana Statistical Services (GSS) in 2010 ^24^. The MDI measures the nature and magnitude of overlapping deprivation at the household level. It is expressed as a percentage of deprivations the poor face in the following three dimensions: health, education and living standards, using ten indicators. The GSS substituted Maternal Mortality as an indicator instead of Malnutrition. Participants will be considered to be deprived based on: 

      (i) Household income, based on Ghana’s minimum wage

                                OR                                

      (ii) Multidimensional poverty index score (≥6/18)

Treatment selection is entirely an independent clinical decision, with no inducements of any sort being given to doctors to choose one medicine over another. Physicians are not being encouraged to utilize innovator medicines but will be guided by the clinical indication for their use. There is not a placebo/non-treatment group.

### Study population and settings

**Figure 1.**
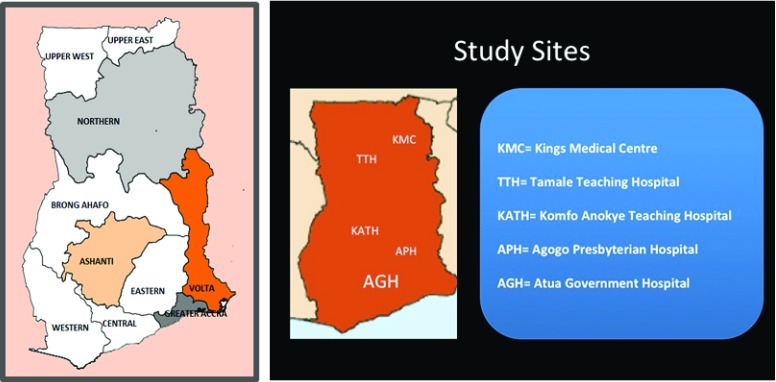
Map of Ghana with regions, with location of target facilities.

Participants will be new or previously diagnosed hypertension and type II diabetes patients who present to one of the participating major referral or district hospitals for medical care. These sites were selected based on the ecological zones of northern savanna, central forest, mixed zone and the coastal belt ( Figure 1). The following inclusion criteria will be applied in patient recruitment:

1. Adult patients 18 years of age and older.
2. Patients with new or previously known diagnosis of hypertension, with SBP> 140 mm Hg and DBP > 90 mm Hg, presenting for routine hypertension management at a polyclinic or established hypertension clinic.
3. Patients with new or previously diagnosed of type II diabetes (fasting serum glucose of 126 mg/dl (7mmol/L) or HbA1 _C_ >7%) presenting for routine diabetes management at a clinic focusing on hypertension or diabetes at one of the participating clinics.

Patients will be excluded based on the following criteria:

1. Patients that are unstable or symptomatic with hypertensive emergency or urgency, requiring hospitalization.
2. Patients with hyperglycemia or hypoglycemia requiring hospitalization.

### Project location and target facilities

***Study sites.*** This is a multi-center study that will be conducted at the following sites.

The project will be implemented at Five Public Health Facilities in four regions of Ghana (see Figure 1).

- The Northern Region – Tamale Teaching Hospital (TTH) & Kings Medical Centre (KMC).
- The Ashanti Region – Komfo Anokye Teaching Hospital (KATH) & Agogo Presbyterian District Hospital.
- The Eastern Region – Atua Government Hospital.

Table 1 includes additional characteristics of the sites.

**Table 1.** Characteristics of study sites.

Site	KATH	Agogo	Atua	Tamale	KMC
*Clinic characteristics*					
Rural/urban	Urban	Rural	Rural	Urban	Rural
Region/District	Regional	District	District	Regional	District
Number of beds	1000	250	135	465	58
Population ( **2010 census**)	2,035,064	140,694	96,982	371,351	112,331
Catchment area	Ashanti Region, Some parts of the Northern, Upper East and West, Brong Ahafo, Western Region, Eastern Region	Asante Akyem North and South, Ejisu Juaben, Togo, Cote D’Ivoire, Burkina Faso	Lower and Manya Krobo Municipalities, Dangbe West, Upper Manya Krobo District	3 Northern Regions, some parts of the Brong Ahafo, Northern part of the Volta Region and Togo and Southern part of Burkina Faso	Tolon and Kumbungu District
*Study characteristics*					
Study physicians (n)	9	4	4	4	2
Study physician assistants (n)	0	1	3	0	1
Study pharmacists (n)	10	2	5	4	1
Research Assistants (n)	8	5	3	4	2
Diabetes clinic (Y/N)	Y	Y	Y	N	Y
Hypertension clinic (Y/N)	Y	Y	Y	Y	Y

### Health systems strengthening interventions

***1. Development of guidelines***

Clinical guidelines will be developed for the study and used as the major tool for training health professionals involved in the study and for improved disease management. The importance of uniform clinical approaches to hypertension and type 2-diabetes is critical, as are standard counseling messages.

***2. Training***

    a. Patient education ( Supplementary File 1)

Patients will receive education and counseling on the medications prescribed for their condition, hypertension and/or diabetes. Additionally patients attending the clinics will be educated about diabetes and hypertension. Education will focus on self-management, disease prevention and control, medication adherence, etc.

    b. Provider training ( Supplementary File 2)

The provider education intervention aims to improve blood pressure and diabetes control by delivering tailored educational resources to health professionals. On-going site level support will be provided throughout the study duration by the Clinical Coordinator (DOA) to ensure compliance with clinical protocols.

    c. Supply chain management ( Supplementary File 3)

The supply chain in the context of this study consists of all stages involved directly or indirectly in fulfilling the request for the supply of medicines needed for the improved health of hypertensive and diabetic patients involved in this study. The supply chain protocol will thus involve the forecasting, ordering, procurement, supply of medicines for the target population in the appropriate quality and quantity, storage of the medicines in the main pharmacy, supply/distribution to the stores of the outlets where medicines will be issued and supplied to patients, and finally storage by the patients and its appropriate utilization.

As part of the health systems strengthening activities in this pilot, training in supply chain management shall be conducted for all the pharmacy staff and other logisticians that would be involved in the management and supply of medicines for hypertension, diabetes and cancer at the pilot sites. After which improvement in the availability, storage, supply throughout the various points in the hospitals and to patients shall be assessed using a check list of indicators attached, as well as occasional focus group discussions during monitoring and supervisory visits. Other supply chain parameters to be assessed will include minimization of stock outs post training intervention, including improved forecasting, ordering, prompt supply, reporting systems, improved storage conditions, and continuous availability of the product in usable forms to patients. Company-appointed local distributors will supply DP medicines to the pilot facilities. The head of the pharmacy department and the procurement personnel at participating facilities will apply supply chain management training obtained in the forecasting ordering and procurement of the DP medicines. The pilot shall establish IT structures in the pharmacy departments and stores of facilities for tracking flow of DP medicines from distributors to the pilot hospitals and for their supply to patients.

### Recruitment, follow-up and data collection procedures for prospective cohort study

***Recruitment process.*** Participants will be recruited into the study by Research Assistants using standardized methodology established for the study. Briefly, patients with diagnosis of diabetes, hypertension or cancer will be approached to participate in the study during clinic registration at established diabetes and hypertension clinics. For sites that do not have an established hypertension or diabetes clinic, patients that meet the inclusion and exclusion criteria will be referred by the physician at an outpatient clinic to the study. Recruitment will take place in a private area and efforts will be made to ensure privacy and confidentiality during the entire process. Eligibility to the DP group will be determined based on the Multidimensional Poverty Index described. This will be communicated to the physician, who when writing a prescription for innovative drugs will indicate whether a patient is eligible for market price or differential price. Physicians may use their discretion to prescribe medications at DP to patients placed on MP based on further interview with the patient on their ability to afford innovator medicine at DP.

**Figure 2.**
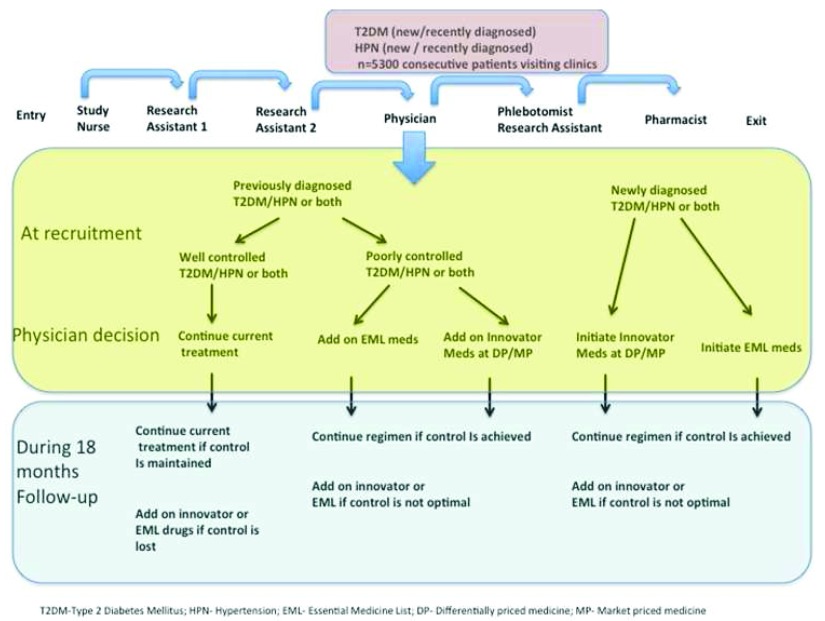
Flow of patients in clinic.

Physicians will not be in anyway influenced to prescribe an innovative drug, but are able to add this drug if they feel it is clinically indicated. If the patient is well-controlled, they will remain on their current treatment. If the physician thinks it is clinically indicated to add an additional drug for blood pressure control (i.e. blood pressure remains greater than 140/90mmHg after multiple attempts at control or uncontrolled diabetes), a drug from the innovative drug list will be added to the regimen. This decision is to be made independently by the physician based on his/her clinical judgment. Physicians will not be encouraged to utilize innovative drugs but may do so if in the exercise of their independent judgment there is a clear clinical indication for their use. Figure 2 displays a flow chart which explains how the study will be integrated as part of routine delivery of care.

**Figure 3.**
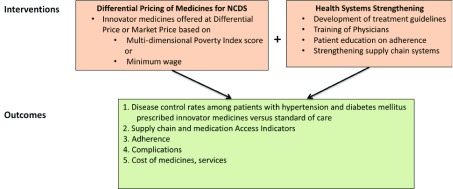
Access and Affordability Initiative conceptual framework.

The study staff will not be blinded to participant intervention status. It is up to the physician to decide which arm of the study the participant should be part of, based on clinical indications for the DP drugs. There will not be a placebo/non-treatment group. A conceptual framework of the GAAP study can be found in Figure 3.

***Informed consent.*** Trained Research Assistants will obtain written informed consent from participants in a private setting prior to enrollment. A case report form will be used to determine whether the participant meets eligibility criteria. For illiterate adults, a thumbprint from the patient or legally authorized signature will be obtained from a legal representative.

***Data collection.*** Two Research Assistants will determine eligibility for the study based on the inclusion/exclusion criteria and obtained informed consent. Data that is being collected includes:

(a) *Demographic and household information*: At baseline, a questionnaire assessing socio-economic and -demographic status, household income, current use of medicines, insurance status, hospitalizations, and complications of their illness will be administered. The assessment of socio-economic and -demographic characteristics for patients within the target population will be based on a pre-validated questionnaire from the Ghana Living Standards Survey, 2010 ^17^. Questions on risk factors were modified from the World Health Organization STEPS questionnaire ^25^.
(b) *Clinical data*: Vital signs, including blood pressure, height, weight, waist circumference measurement, as well as fasting blood sugars and other laboratory tests (e.g., Hemoglobin A1C, serum creatinine). Please refer to the GAAP Clinical Data Form in the Supplementary File 3 for more information. In the blood pressure measurement intervention, each site was provided with an automated blood pressure measurement device (Omron HEM-907XL). Two consecutive measurements will be collected. At initial rollout, education sessions will be held at each site to introduce the devices and provide clinicians and staff with evidence for the importance of accurate blood pressure measurements. Culturally and linguistically tailored posters explaining the new procedure for blood pressure measurement will be strategically placed throughout the clinics to promote patient engagement. To improve sustainability of the intervention, the device maintenance and personnel training responsibilities will be transferred to the organization’s quality improvement department and key staff at individual clinic sites.
(c) *Laboratory data:* A quality-assured central laboratory will be contracted to run all biochemistry panels to ensure uniformity of data across sites, including hemoglobin A1C every 6 months, creatinine at baseline and at the end of the study, routine fasting blood sugars.

If patients decline to join the study, they will receive treatment according to national treatment guidelines. The window for enrollment has been set at six months.

Those joining the study will have data recorded concerning their compliance, control, complications, treatment from the doctors and knowledge about their disease regardless of whether they are receiving the innovative medicines or not.

If participants are diagnosed with complications from diabetes or hypertension, they will be referred to the appropriate hospital resources.

The pharmacist will (1) dispense medications appropriately with reference to the patient unique ID and (2) advise patients on their appropriate use.

Participants would then be followed up for the period of up to 18 months allowing for maximum of 6 months recruitment of incident cases within each selected site.

An inventory will be conducted weekly to determine what and how many medicines are dispensed to patients. Research Assistants will use an observational check list and questionnaires (see Supplementary File 3) on a weekly/monthly basis to determine if medicines are appropriately stored, the incidence of stock-outs, if patients receive appropriate counseling from pharmacists/dispensing technicians, if patients understand how to take and store the medicines they receive, and if medicines prescribed are dispensed as directed. Prescription refills were tracked on a monthly basis. Dispensed medicines will be tracked using an inventory and entered into RedCap. Each pharmacy will identify one individual that will be in charge of recording this information and putting it into a database on a weekly basis.

Furthermore, patients who are not enrolled into the study but are prescribed study medications by their physicians may access them at market price. For accountability purposes, details of such as patients such as age, gender, medical diagnosis and study medication prescribed, will be recorded to help track study medicines usage at the study site.

In addition, key informant interviews will be conducted on supply chain management, using a checklist guide to ascertain current practices in relation to supply of drugs, source of purchases, types of medicines and storage facilities present.

All of the drugs supplied by the pharmaceutical companies are approved and registered by the Food and Drugs Authority (FDA) in Ghana ( Table 2).

**Table 2.** List of innovator medicines.

Amlodipine besylate	CARDIOVASCULAR DRUG	REGISTERED WITH FDA
Atorvastatin calcium		
Ibersartan	CARDIOVASCULAR DRUG	REGISTERED WITH FDA
Ibersartan and HCTZ	CARDIOVASCULAR DRUG	REGISTERED WITH FDA
Ibersartan	CARDIOVASCULAR DRUG	REGISTERED WITH FDA
Losartan 50mg	CARDIOVASCULAR DRUG	REGISTERED WITH FDA
Losartan 50mg + HCTZ 12.5mg	CARDIOVASCULAR DRUG	REGISTERED WITH FDA
Losartan 100mg + HCTZ 12.5mg/tabs	CARDIOVASCULAR DRUG	REGISTERED WITH FDA
Sitagliptin 25mg, 50mg, 100mg	DIABETIC DRUG	REGISTERED WITH FDA
Sitagliptin 50mg + Metformin 850mg	DIABETIC DRUG	REGISTERED WITH FDA
Sitagliptin 50mg + Metformin 1000mg	DIABETIC DRUG	REGISTERED WITH FDA
Insuline Glargine	DIABETIC DRUG	REGISTERED WITH FDA

### Study sample size

For the purposes of the nested study, the effect of differential pricing on health and access outcomes will be analyzed. A sample size of 2,744 was calculated with an alpha of 5% and power of 80% in order to detect a 5% difference between differential price group versus market price group among the study participants to estimate health outcomes (controlled versus uncontrolled disease) as a dichotomous variable. This calculation was done assuming that 15% of patients would be on DP and 10% on MP, with an equal number of hypertension and diabetes patients. Assuming a loss-to-follow up rate of 10%, it is estimated that 3018 patients would need to be recruited. If a 20% loss to follow up rate is assumed, then 3292 patients would need to be recruited. We therefore propose to recruit 3,300 patients with hypertension, diabetes, or both diabetes mellitus and hypertension.

### Focus groups

We will conduct a contextual qualitative analysis to determine attitudes/perceptions about chronic disease management. Key Informant Interviews (KII) will be conducted with health workers (Medical Doctors & Nurses) involved in the GAAP pilot interventions at the study health facilities and patients enrolled into the program. Focus Group Discussions (FGD) will be conducted with diabetic and hypertensive patients enrolled into the project. Purposive sampling method will be used to select participants for the interviews. First of all, patients with hypertension and diabetes recruited into the GAAP program in the five study sites will be selected by the research assistants working on the project, based on their availability, date, time and venue for the discussions assessed through phone calls.

All the interviews will be transcribed verbatim after repeatedly listening to the recordings. The transcripts will be uploaded onto QSR Nvivo 10 software to facilitate data management and coding. Guided by the objectives of the study and the themes contained in the interview guides, a codebook will be developed to facilitate data coding and analysis. The coding process involved two stages: first, the data will be coded into major themes while at the second stage, the data will be coded into sub-themes. Thematic analysis framework will be used to analyze the data. The records will be reviewed and analyzed by trained investigators to categorize the themes that arise.

Supplementary File 4 includes the focus group discussion outlines.

### Key informant interviews

Physicians, physician assistants and nurses involved in the study will participate in key informant interviews to elicit their perspectives on access and affordability of study medicines. Written informed consent will be obtained and a questionnaire guide will be designed to conduct this qualitative aspect of the study (please refer to GAAP qualitative survey in Supplementary File 4).

### Study outcome measures

Two main primary outcomes will be assessed: access to medicines for the treatment of diabetes and hypertension, and disease control. Secondary outcomes, such as medication adherence, patient behavior, knowledge and practices, cost of medicines, complications, number of hospitalizations, will be evaluated as detailed in Table 3.

**Table 3.** Study outcome measures.

	Measure	Frequency	Notes and definition
PRIMARY OUTCOMES			
*A. Disease control*			
1. Hypertension control	Proportion of patients with controlled blood pressure	Routine (each visit)	Uncontrolled BP ≥140/≥90 (or ≥130/≥80 if DM or chronic kidney disease)
2. Diabetes control	Proportion of patients with controlled diabetes	0,6,12 months	Uncontrolled Diabetes if HbA1C < 7 or FBS < 126 mg/dl
*B. Supply chain/access to medicines*			
	Number of stock-outs	Monthly	
	Ability to access GAAP medicines at enrolled sites	Monthly	
	Consumption rates of GAAP medicines (uptake or prescription refills)	Monthly	
	Number inventory checks, forecasting reports, procurement events/cycles for GAAP medicines and other medicines in therapeutic classes	Weekly	
	Assessment of prescriber patterns for the GAAP medicines from patient files and prescriber	0, 6, 12, 18 months	
	Source of supply	0, 6, 12, 18 months	
	Delivery time from ordering	0, 6, 12, 18 months	
	Frequency of orders	0, 6, 12, 18 months	
	Mode of delivery to health facility	0, 6, 12, 18 months	
SECONDARY OUTCOMES			
*A. Adherence to medicine*			
Hypertension medication adherence	The Hills-Bone Compliance to Blood Pressure Therapy Scale	0,6,12, 18 months	14 items assessing study participant’s self-reported adherence (reduced sodium intake, appointment keeping and medication taking). Items are assumed to be additive, and, when summed, the total score ranges from 14 (minimum) to 56 (maximum)
Diabetes medication adherence	Morisky, Green and Levine (MGL) Medication Adherence Questionnaire	0,6,12, 18 months	1 item used to determine adherence
*B. Knowledge, Attitude and Practice*			
	Questionnaire (qualitative)	0,6,12, 18 months	Questionnaire assessing knowledge about hypertension and diabetes
*C. Cost of medicines*			
	Out of pocket (OOP) cost	0,6,12, 18 months	Computed by person months on treatment with the same medication
	Cost to Health System	0,6,12, 18 months	Assessed both with and without the effects of price differentials
*D. Complications*			
	Proportion of patients with renal failure (proteinuria or GFR consistent with chronic renal failure)	0,6,12, 18 months	Measurement of GFR, BUN/Cr, urine protein
	Proportion of patients with lower limb amputations, peripheral neuropathy, retinopathy, stroke cardiovascular events, stroke	0,6,12, 18 months	Self-report or medical records

***Primary outcomes***

1. Access to medicines and supply chain
a) Assess incidence of stock-outs of innovative and other medicines in the therapeutic classes via questionnaire, observation and inspection of inventory records
b) Assess the frequency of enrolled patients’ difficulties in accessing innovative medicines and others in the therapeutic classes at pilot facilities; percentage of patients sent with prescriptions by hospital pharmacy/prescribers to community pharmacies for innovative medicines and other medicines used in the management of the therapeutic areas
c) Number inventory checks, forecasting reports, procurement events/cycles per month, week or quarter for innovative medicines and other medicines in therapeutic classes
d) Consumption rates and/or patterns of the innovative medicines from pharmacy records
e) Assessment of prescriber patterns for the innovative medicines from patient files and prescriber
f) Source of supply
g) Delivery time from ordering
h) Frequency of orders
i) Mode of delivery to health facility
2. Disease control

  A. Hypertension

Blood pressure control: Blood pressure will be recorded at each visit per routine. For the purposes of analysis, we will compare blood pressure at baseline, 6 months, 1 year, 18 months to see if there is improvement in blood pressure. We will compare the proportion of persons achieving blood pressure control (<140/90 mm Hg) at 18 months between the two groups using chi-square statistics and various longitudinal data analysis methods. Blood pressure measurements will be done according to a pre-existing protocol, which uses automated measurement methods.

  B. Diabetes

Glycemic control: FBS or HbA1C will be assessed at baseline and every 6 months. The proportion of patients with achieved glycemic control will be compared using chi-squared statistics (HbA1C < 7% or FBS < 7mmol/l or 126 mg/dl).

***Secondary outcomes***

(1) Medication adherence
  a. Hypertension: The Hills-Bone Compliance to Blood Pressure Therapy Scale (14 items) assesses patient’s self-reported adherence with reduced sodium intake, appointment keeping and medication taking ^26^.
  b. Diabetes: A questionnaire derived from the Morisky, Green and Levine (MGL) Medication Adherence Questionnaire, was used to determine adherence to Diabetes treatment ^27^.
(2) Patient behaviors and knowledge and practices

An exit interview and questionnaire will be administered to patients to assess knowledge about their illness as well as self-management behaviors pre and post- counseling intervention. This will include items such as diet, exercise, smoking and alcohol. In addition it will assess some standard measures of knowledge about their specific disease. Part of this exit interview will also include satisfaction and trust questions.

(3) Cost of medicines
  a. Out-of-pocket costs (OOP): measured for both those receiving differentially priced medications and those paying full Ghana pricing for the same medication. The OOP costs are computed by person months on treatment with the same medication. This will be determined via the enrollment survey and exit interviews every 6 months. We will also determine the percentage of patients who use co-payments for medicines that are not on the EML.
  b. Costs to the health system: both with and without the effects of price differentials will be estimated in the enrollment questionnaire and in the exit interviews (every 6 months).
(4) Number of hospitalizations
  a. Hypertension

We will compare the number of persons requiring hospitalizations for hypertension urgency or emergency between the two groups using chi-square statistics.

b. Diabetes

We will compare the proportion of persons requiring hospitalization for hyperglycemia, hypoglycemia or diabetic ketoacidosis between the two groups using chi-square statistics.

(5) Complications from hypertension and diabetes

We will compare the proportion of persons with complications from diabetes and hypertension including: nephropathy, retinopathy, lower limb amputation, and cardiovascular disease events (incidence of ischemic heart disease) and stroke. Blood and urine will be obtained to assess the presence of chronic kidney disease at baseline and every 6 months. Estimated GFR will be calculated using the CKD-EPI equation. Urine analysis (UA) will be obtained to assess for the presence of proteinuria. Medical records will be reviewed to assess if a physician has noted the presence of complications such as retinopathy, amputations, stroke, myocardial infarction or foot infections.

(6) Economic analysis

An economic analysis will be conducted which will include a cost analysis of the interventions, analysis of the following: efficient use of differentially priced medicines, value analysis of DP, supply chain structure and incentives and affordability index.

### Statistical analyses

Data analysis will include descriptive statistics (means, medians), as well as comparison of standard of care patients and patients prescribed study medicines, looking at the following outcomes: access to treatment, disease control (diabetes and hypertension), treatment adherence, supply chain, out of pocket expenditures and average cost of medicines. Patients with a dual diagnosis of hypertension and diabetes will be analyzed separately. Statistical analysis will be performed using SPSS version 19.

Logistic regressions will be performed in order to analyze dichotomous outcomes (hypertension and diabetes control versus uncontrolled) and continuous outcomes (for longitudinal analyses of disease control), controlling for confounding and mediating variables (education, marital status, age, household income, duration of diagnosis, area of residence, adherence to medications and duration on innovator medications) in the model. Continuous outcomes (for example, out-of-pocket medication expenditure, number of hospitalizations, proportion of patients with complications in the two groups) will be analyzed using multiple linear regression and mixed-effects models, controlling for confounding and mediating variables. Pre-post analyses of the DP and MP groups will be conducted separately. Longitudinal analysis methods, such as Kaplan-Meier, Cox proportional hazards regression or Poisson’s regression, will be employed to monitor trends over time. Qualitative data will be analyzed from the records from the survey using thematic approaches for processing with the aid of Nvivo (version 9) or similar software.

### Safety

Only products that have been previously been approved and registered in Ghana by the FDA will be utilized in the pilot study. Adverse events will be closely monitored at each site using standardized reporting forms. Side effects will be graded as mild (grade 1), moderate (grade 2), severe (grade 3), life threatening (grade 4) or fatal (grade 5) according to the NIH/NCI Common Toxicity Criteria for each of the innovator medicines ^28^. Where the side effects are deemed severe or life-threatening, the medication will be stopped immediately and patient admitted for appropriate management of the side effect. The list of potential side effects of each of the innovator medicines will be provided to prescribers at each of the pilot sites. Since the medications for the study have marketing authorization from the FDA for use in Ghana, reports of adverse events will be captured on forms used on post-market surveillance and forwarded to the authorities for their notification.

### Follow-up

Participants will attend clinic visits per their routine schedule. It is likely that these patients will be returning on a monthly or every 2 months based on the clinician’s recommendations. All scheduled visits will be entered into the REDcap electronic database. Provisions will be made for out-of-scheduled visits. However, specific data will be collected at six-month intervals, for a total duration of 18 months for each patient. For patients that are lost to follow-up, research assistants will inquire about the cause of loss to follow-up. For patients who die at a health facility or at home, there will be an inquiry about the cause of death by either a chart review or inquiry with the family.

### Trial status

Patient enrollment began in July 2014 and by May 2015, 3,300 patients were successfully recruited. Provider education and pharmacist supply chain training began in November 2015. The study was completed on June 30, 2017. Data analysis is currently ongoing. Results of the study will be provided to the Ghana Ministry of Health, as well as relevant policy makers. The study outcomes will be summarized in the form of scientific manuscripts, which will be submitted to peer-reviewed journals. The Study Data, which is relevant to a publication authored by the investigators, will also be available for review in a public data repository.

Patients on study medicines will continue to have access to the medicines at the differential price after the study ends. The participating companies undertake to ensure study medicines are available at the project price to all participants that are enrolled in this pilot study and are on the study medicines, for as long as the medicines are available in Ghana.

## Discussion

This study aims to test approaches intended to improve both access to treatment as well as blood pressure and diabetes control, by introducing differentially priced drugs for patients meeting poverty criteria and implementing a multi-level health systems intervention. Because we have applied a pragmatic study design, our interventions take place within existing clinic practices. The data generated by the study will allow stakeholders to guide decision-making in the development of policies and procedures to improve access to medications for the treatment of hypertension and type 2 diabetes. A recent commentary in the Lancet suggested that innovative collaborations between stakeholders are needed to achieve WHO’s NCD Global Action Plan to achieve 80% availability of essential medicines in both public and private facilities in the next decade ^29^. In addition, the pilot will facilitate health system strengthening at the participating institutions, which includes training of clinicians and production of guidelines for the treatment of diabetes, hypertension and cancer. Further, it will provide descriptive information on the management of hypertension and diabetes at representative sites in Ghana and longitudinal data on the course of disease over the study period.

Access to treatment and control of chronic diseases are influenced by a myriad of factors including availability, access and affordability of quality assured medications, cost of treatment, adherence to long-term medications and therapeutic lifestyle interventions, prescriber knowledge and compliance to established treatment guidelines to name a few.

Our mixed methods study has designed robust measures to capture the multi-dimensional indicators that influence access to and affordability of care, control and management of diabetes mellitus, hypertension and cancers in Ghana. This study is also important as it will provide insight into the current drivers of poor control rates of non-communicable diseases in resource-limited settings. In addition, qualitative studies will capture the attitudes of both patients and physicians towards the treatment of chronic disease.

Strengths of this study include the wide geographic distribution in several regions of Ghana to capture the experiences in the management of patients with NCDs in rural, semi-urban and urban settings, a prospective design with 18 months of follow-up for each participant to assess outcome indicators, and a sample size that is powered to allow for robust evaluation of the main outcomes. A potential limitation of the study is the absence of a control group; however, we have proposed a comparison of patients prescribed study medications with those not prescribed any differentially priced medications during the entire study period. When comparing the MP and DP groups, these two groups might not be similar; as they will likely differ based on socio-economic status. Data on potential confounders will be collected and accounted for as part of the analysis.

In addition, validation of the multi-dimensional poverty index was not done as home visits were not conducted. Also, a separate supply chain was created for the study with a single sourcing agreement established at each of the facilities. However, once procurement was done at the facility level, medicines were integrated in the existing supply chain at facilities.

In conclusion, our study is poised to evaluate differential pricing of innovative medicines, based on poverty index criteria, as a model for improving access to and affordability of these quality-assured medicines in the management of hypertension, diabetes and cancers. Health systems strengthening interventions that have been implemented will help provide guidance on policies designed to improve the control rates of these prevalent non-communicable diseases in resource-limited settings.

## Ethics statement

Ethical approval was obtained from the Ghana Health Services and the Committee on Human Research, Publications and Ethics (CHRPE/AP/298/14) Kwame Nkrumah University of Science and Technology, School of Medical Sciences & Komfo Anokye Teaching Hospital (ID No. GHS-ERC: 12/07/14). All patients provided written informed consent to participate in the study. The Johns Hopkins Bloomberg School of Public Health waived the right for Ethical approval (IRB No. 0005836), as it was only involved in secondary data analysis.

## Data availability

All data underlying the results are available as part of the article and no additional source data are required.

## Supplementary material

Supplementary File 1: Promotional materials for patients about diabetes and hypertension.

Supplementary File 2: Treatment guidelines.

Supplementary File 3: Data collection tools, including the dispensing register form, the monthly supply chain checklist, the monthly stock report, the supply chain questionnaire, participant booklet and GAAP clinical data form.

Supplementary File 4: Qualitative survey materials, including focus group discussion outlines and interview questions.
