# Supplementary material for: A multi-center prospective cohort study to evaluate the effect of differential pricing and health systems strengthening on access to medicines and management of hypertension and diabetes in Ghana: A study protocol
Source: Gates Open Res. 2018 May 3;2:6. Originally published 2018 Feb 7. [Version 2] doi: 10.12688/gatesopenres.12797.2 (PMC5941285; doi:10.12688/gatesopenres.12797.2)

ghana access and affordability program

EFFECTS OF UNTREATED DIABETES

BLINDNESS

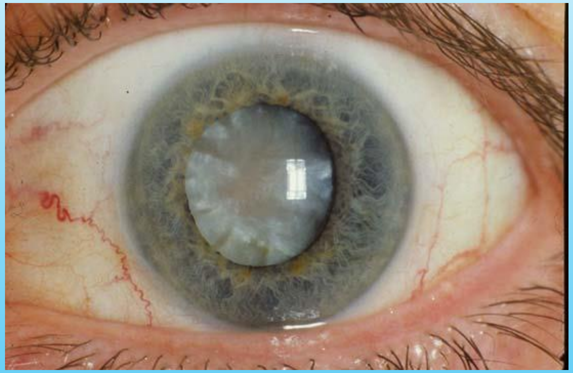

HEART PROBLEMS

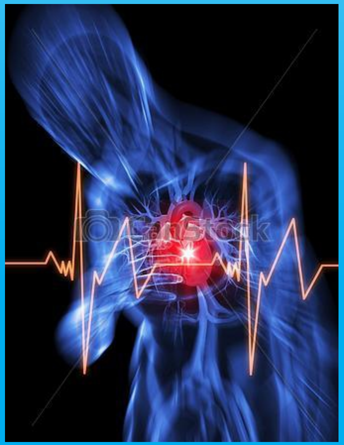

Heart attack

FOOT/LEG PROBLEMS

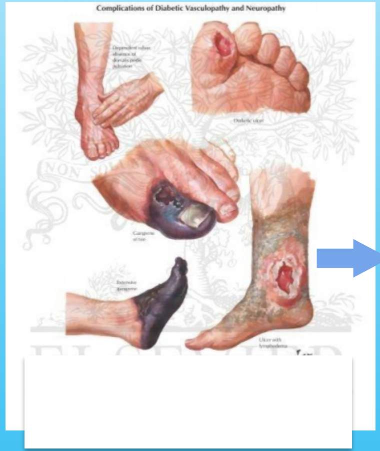

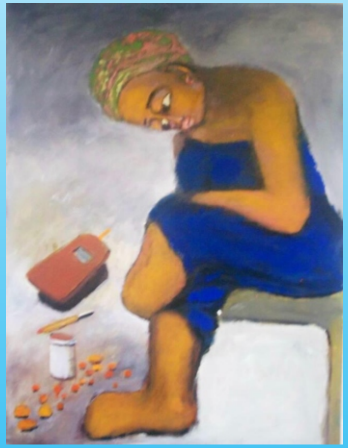

KIDNEY PROBLEMS

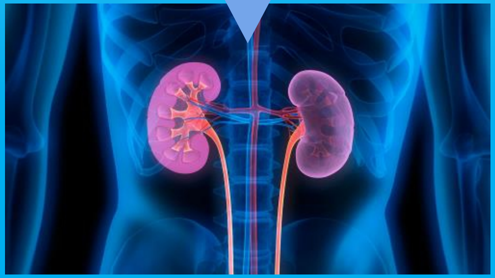

DIABETES

Eat healthy fruits and vegetables

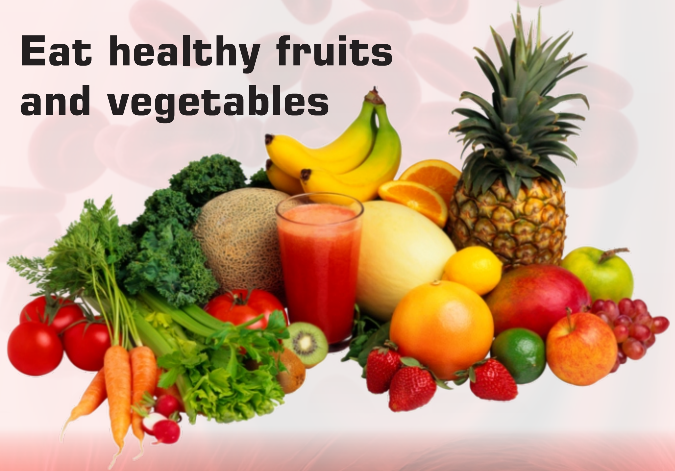

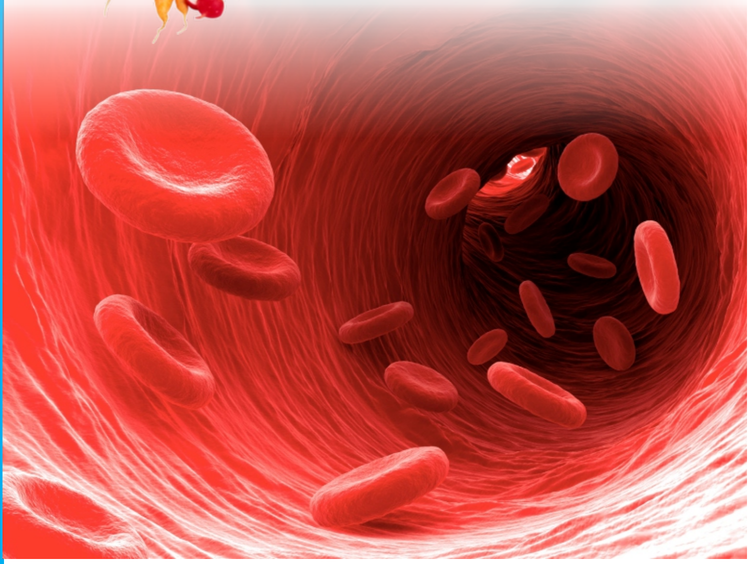

ghana access and affordability program

WHAT IS DIABETES?

Diabetes is adisease that causes sugar levels in your blood to rise higher than normal.

Insulin is produced by your body to use sugar as energy

When you have diabetes, your body doesn'tuse insulin well.

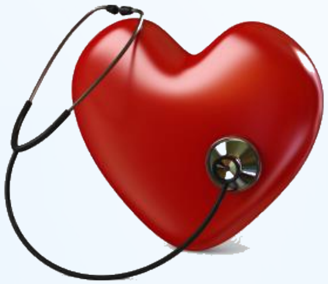

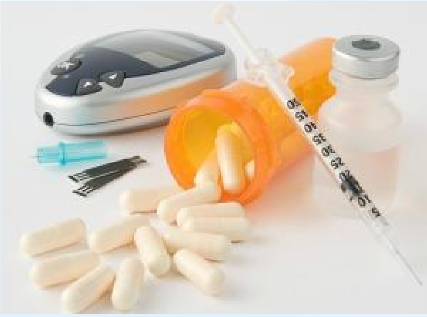

HOW DO I TAKE CARE OF MY DIABETES?

- EXERCISE AS MUCH AS YOU CAN
- TAKE YOUR MEDICINE EVERYDAY
- MAINTAIN A HEALTHY WEIGHT
- EAT FRUITS AND VEGETABLES

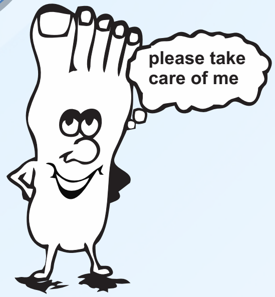

WHAT ARE THE EFFECTS OF UNTREATED DIABETES?

- BLINDNESS
- HEART ATTACK
- NERVE DAMAGE-PAIN IN HANDS AND LEGS
- GANGRENE,ULCERS
- KIDNEY PROBLEMS

SIGNS OF DIABETES

- INCREASED THIRST
- INCREASED URINATION

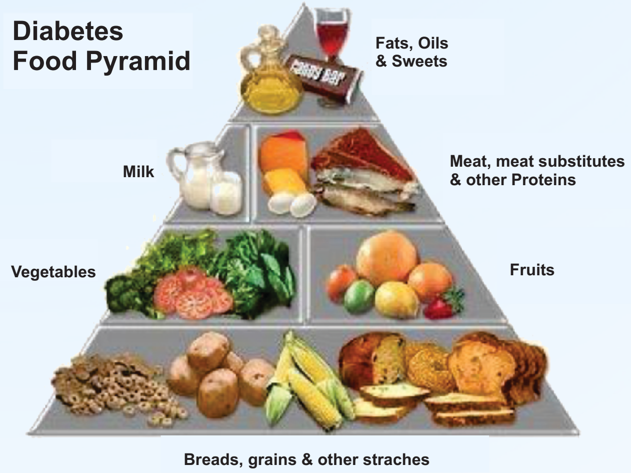

Supplement: Supplementary file 1 [file gatesopenres-2-13889-s0000.tgz › 9a33f689-a47d-49cd-ac0e-1e600a1f5f91.zip!/DIABETES_PATIENT_EDUCATION_TYPESETTING.pdf]
