# Supplementary material for: A multi-center prospective cohort study to evaluate the effect of differential pricing and health systems strengthening on access to medicines and management of hypertension and diabetes in Ghana: A study protocol
Source: Gates Open Res. 2018 May 3;2:6. Originally published 2018 Feb 7. [Version 2] doi: 10.12688/gatesopenres.12797.2 (PMC5941285; doi:10.12688/gatesopenres.12797.2)
Supplement: Supplementary file 1 [file gatesopenres-2-13889-s0000.tgz › 9a33f689-a47d-49cd-ac0e-1e600a1f5f91.zip!/HYPERTENTION_PATIENT_EDUCATION_TYPETTING.pdf]

COMPLICATIONS OF HYPERTENSION

BLINDNESS

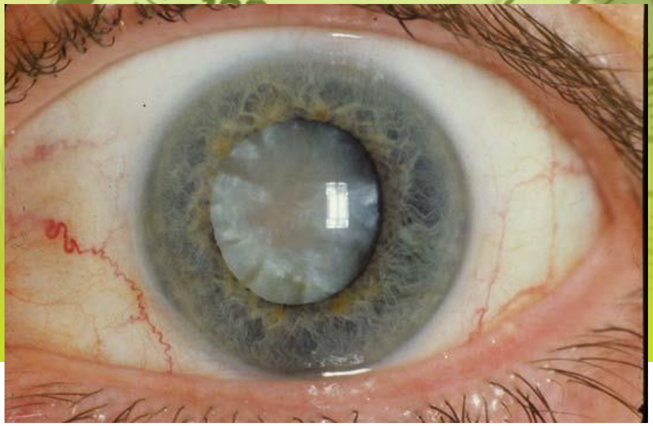

STROKE

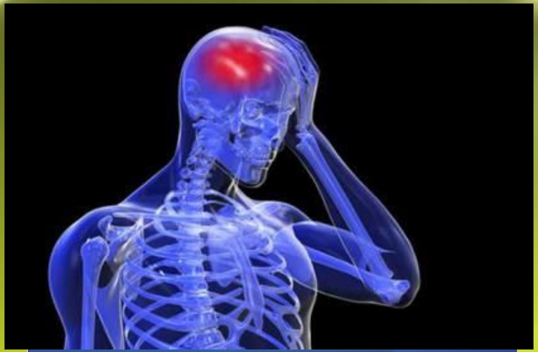

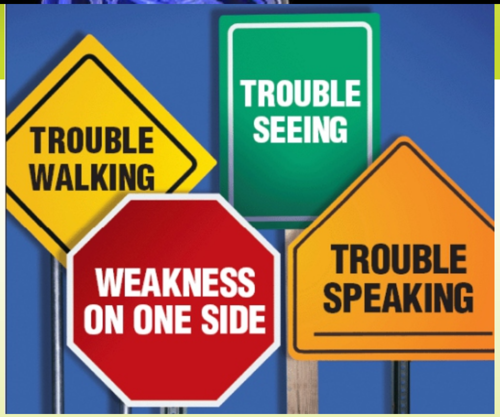

HEART PROBLEMS

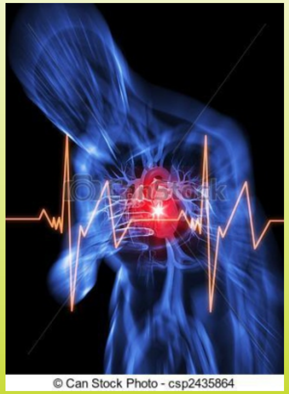

HEART ATTACK

HEART FAILURE

KIDNEY PROBLEMS

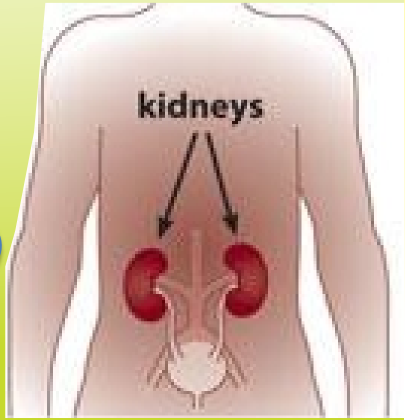

HYPERTENSION

(HIGH BLOOD PRESSURE)

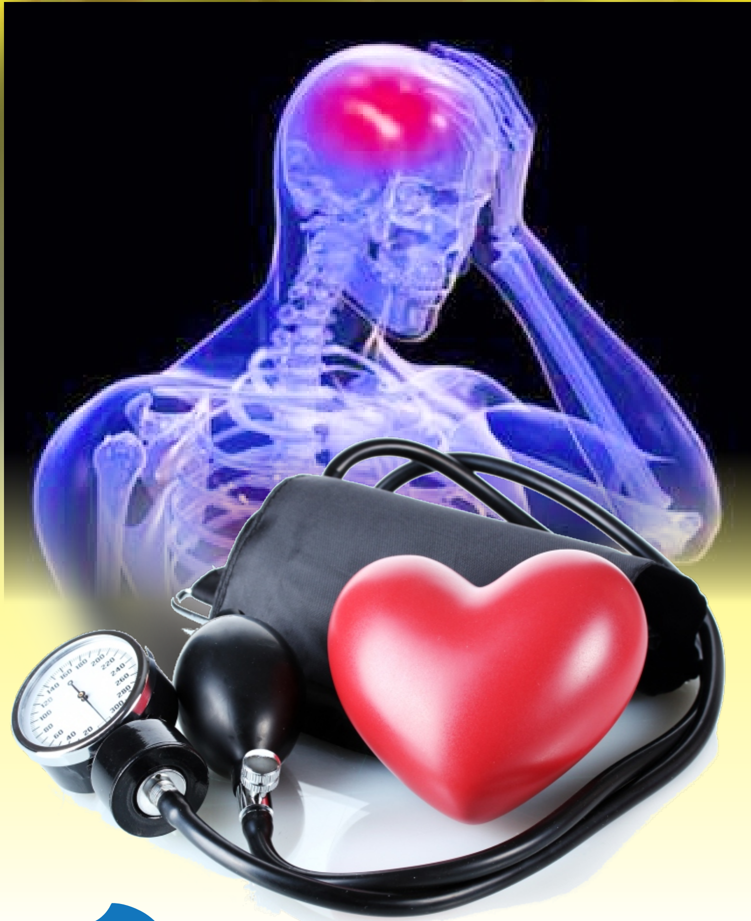

ghana access and affordability program

WHAT IS BLOOD PRESSURE?

Blood pressure is the force of blood moving through your arteries

HOW DO YOU CONTROL YOUR BLOOD PRESSURE?

Take your Medicine everyday
Exercise-be active everyday
Eat less salt
Eat a healthy diet with less fat and more fruits and vegetables

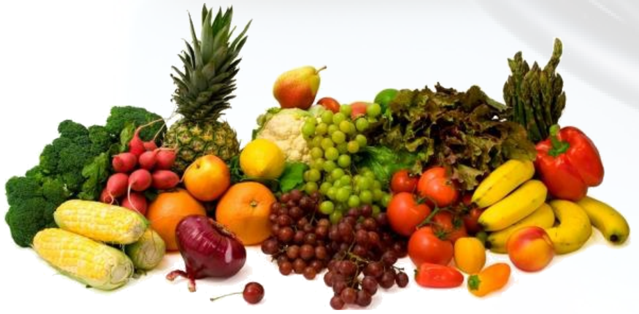

ghana access and affordability program

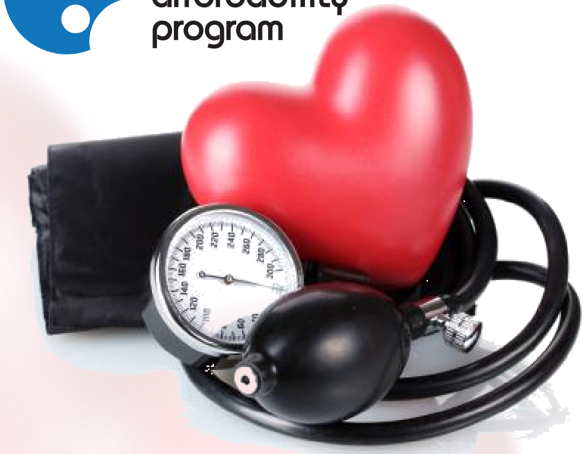

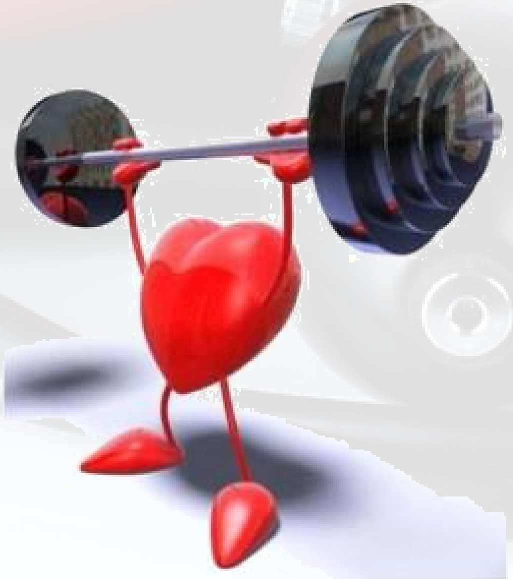

WHAT IS A GOOD BLOOD PRESSURE?

Ideally, a good blood pressure is less than 120/80

High blood pressure is greater than 140/90

120 → THE TOP NUMBER IS THE SYSTOLIC BP = FORCE OF BLOOD IN YOUR ARTERIES WHEN YOUR HEART BEATS

80 → THE BOTTOM NUMBER IS THE DIASTOLIC BP = FORCE OF BLOOD IN YOUR ARTERIES WHEN YOUR HEART RELAXES

WHAT ARE EFFECTS OF UNTREATED HIGH BLOOD PRESSURE

- STROKE
- EYE DAMAGE
- KIDNEY PROBLEMS
- HEART
- LEG PAIN
