# Supplementary material for: A multi-center prospective cohort study to evaluate the effect of differential pricing and health systems strengthening on access to medicines and management of hypertension and diabetes in Ghana: A study protocol
Source: Gates Open Res. 2018 May 3;2:6. Originally published 2018 Feb 7. [Version 2] doi: 10.12688/gatesopenres.12797.2 (PMC5941285; doi:10.12688/gatesopenres.12797.2)

GHANA ACCESS and AFFORDABILITY PROGRAM PILOT STUDY

GUIDELINES FOR THE MANAGEMENT OF SELECTED CANCERS

PURPOSE OF GUIDELINES

These guidelines are provided to assist doctors involved in the management of selected cancers in the Ghana Access and Affordability Project pilot study. It is acknowledged that the management of patients with cancers in study will take place at specialised facilities where the expertise exists. The selected cancers are breast, colorectal and prostate cancers.

The guidelines were prepared by:

Dr Baffour Awuah, Radiation Oncologist, Komfo Anokye Teaching Hospital, Kumasi

Dr Verna Vanderpuye, Radiation Oncologist, Korle Bu Teaching Hospital, Accra

Mrs Amah Nkansah, Chief Pharmacist, Korle Bu Teaching Hospital, Accra

Facilitators:

Professor David Ofori-Adjei, Clinical Coordinator, GAAP

Mrs Lynda Arthur, Programme Manager, GAAP

Ms Sarah Asare, GAAP Secretariat

DEFINITIONS

Chemotherapy refers to the treatment of cancer with drugs that kill the cancer cells Chemotherapy drugs can be given in a variety of ways, including intravenously by injection, intravenously with a pump, or even in pill form taken by mouth.

Palliative chemotherapy refers to drugs given when the cancer is advanced and spread to other parts of the body; and when surgery cannot eliminate the cancer. This treatment may shrink tumours, alleviate symptoms, and prolong life.

Adjuvant chemotherapy refers to chemotherapy after surgical removal of the cancer; and may refers to s given after the cancer is surgically removed.

Neoadjuvant chemotherapy refers to chemotherapy given before surgery to aid in shrinking a tumour. Chemotherapy may also be given with radiotherapy.

BREAST CANCERS

These guidelines are for patients with breast cancer (newly diagnosed or treated).

Definition:
Cancer that forms in the tissues of the breast

Patient Evaluation and Staging
Histologically confirmed lesion
Hormone receptor status
TNM (Tumour size, lymph **n**ode involvement and **m**etastasis) staging

CHOICE OF THERAPY

The choice of therapy depends on:

- Age
- Menopausal status
- Histologically confirmed lesion
- Hormone receptor status
- Stage of disease and prognostic factors.
- Co-morbidity

TREATMENT GOALS

- Improved quality of life as determined by the European Union's Cancer QOL questionnaire
- Control of tumour growth
- Alleviating disease symptoms
- Improve progression free survival or prolong life

TREATMENT OPTIONS

The various treatment options are given below with their indications and choice of medications.

Surgery
The lesion must be resectable upfront or following neo-adjuvant chemotherapy,
Modified radical mastectomy is recommended for lesions greater than 5cm, repeatedly positive margins and skin involvement.
Lumpectomy and axillary dissection are indicated for lesions less than 5cm

Radiotherapy

- All post lumpectomy and axillary clearance
- Post mastectomy radiotherapy for all node positive, positive margins, greater than 5cm,
- Radiotherapy alone for unresectable lesions and palliation.
- Complete all chemotherapy cycles prior to radiation

Hormone therapy
Hormone therapy is for patients who are oestrogen or progesterone receptor positive
Types of hormone therapy are:

- Total hormone receptor blockage with Aromatase inhibitors; use Goserelin for pre-menopausal patients with positive receptor status
- Selective Oestrogen Receptor Modulators, for example, Tamoxifen

- Aromatase inhibitors (Anastrazole or Letrozole or Exemestane) for postmenopausal women

Chemotherapy
Chemotherapy can be given to all patients with cancer of the breast greater than 1cm including patients who are triple negative (oestrogen receptor, progesterone receptor and Human Epidermal Growth Factor receptor (Her2/neu) negative)
Chemotherapy may be given as Neo-adjuvant, Adjuvant or for Palliation as monotherapy.
Any of the following combinations can be used:

First line treatment
CMF = Cyclophosphamide, Methotrexate and 5-fluorouracil
CAF = Cyclophosphamide, Adriamycin (Doxorubicin) and 5-fluorouracil
CAF + T = Cyclophosphamide, Adriamycin (Doxorubicin), 5-fluorouracil plus Taxane (Paclitaxel)
AC + T = Adriamycin, Cyclophosphamide + Taxane (Paclitaxel)
DC = Docetaxel , cyclophosphamide
TAC = Taxane (Docetaxel), Adriamycin and Cyclophosphamide

Second line treatment
Cannot repeat Adriamycin as maximum life time dose is about 400mg/m2. Patients may receive Taxanes , capecitabine, vinca alkaloids, Gemcitabine , erubulin in combination or as single agents

Metastatic lesions
Zoledronic acid for bone metastasis
Palliative radiotherapy for bone, brain and occasionally lung metastases
Chemotherapy or hormonal therapies if patient is receptor positive but choice of medications depends on response to previous therapies and disease free interval.

Target therapy
For patients positive for Her2/neu give Trastuzumab with chemotherapy but cannot be given with adriamycin concomitantly.

Sequential therapy
Chemotherapy followed by hormonal therapy and not vice versa
Avoid hormonal therapy concomitant with chemotherapy as this is detrimental to the patient.
Single agent chemotherapy given one after the other is also called sequential therapy.

Combination therapy
Chemotherapy. (Avoid adriamycin + Target Therapy)
Hormone Therapy (preferably aromatase inhibitors) + Target Therapy

EARLY STAGE BREAST CANCER

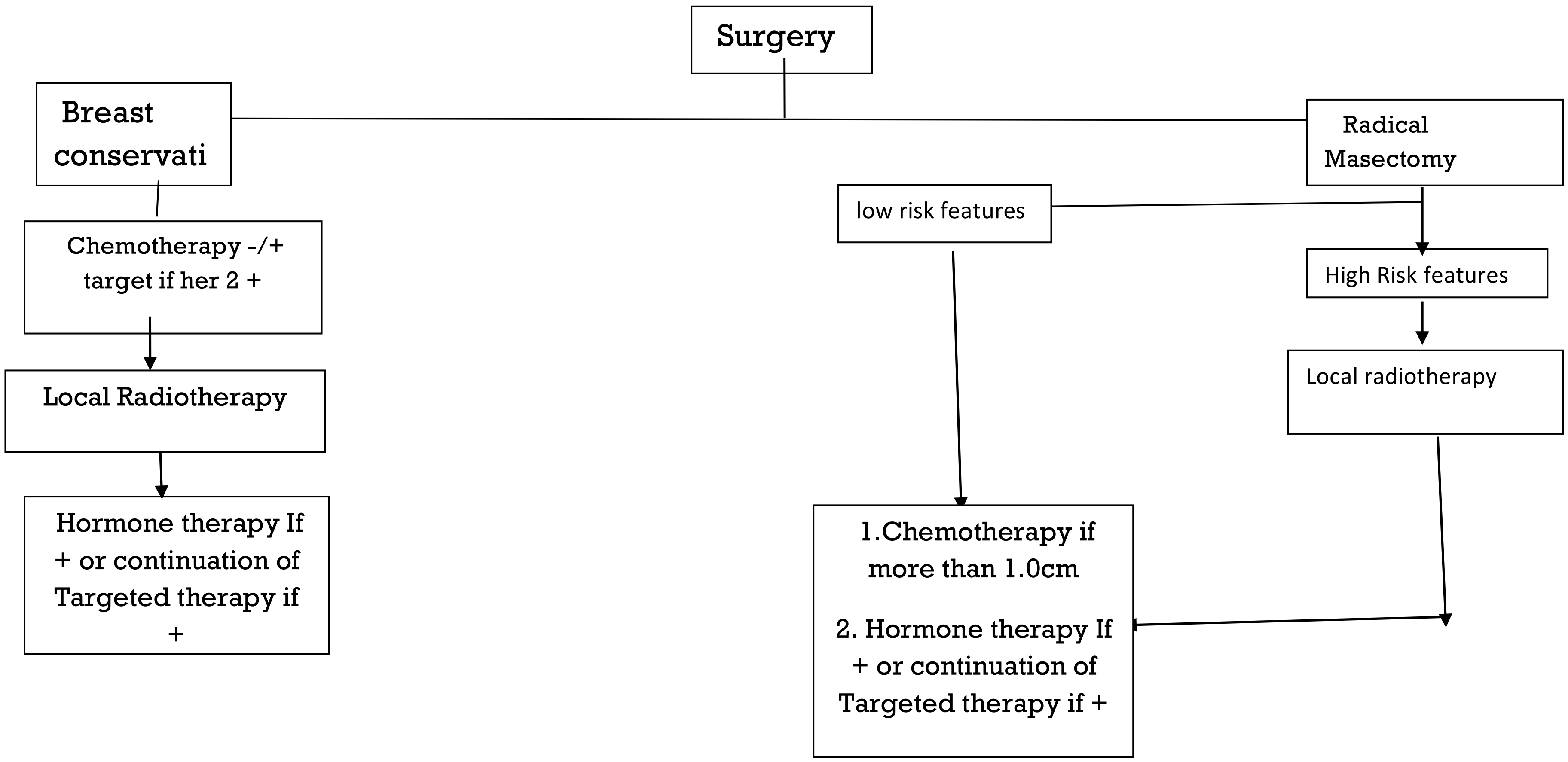

LOCALLY ADVANCED BREAST CANCER

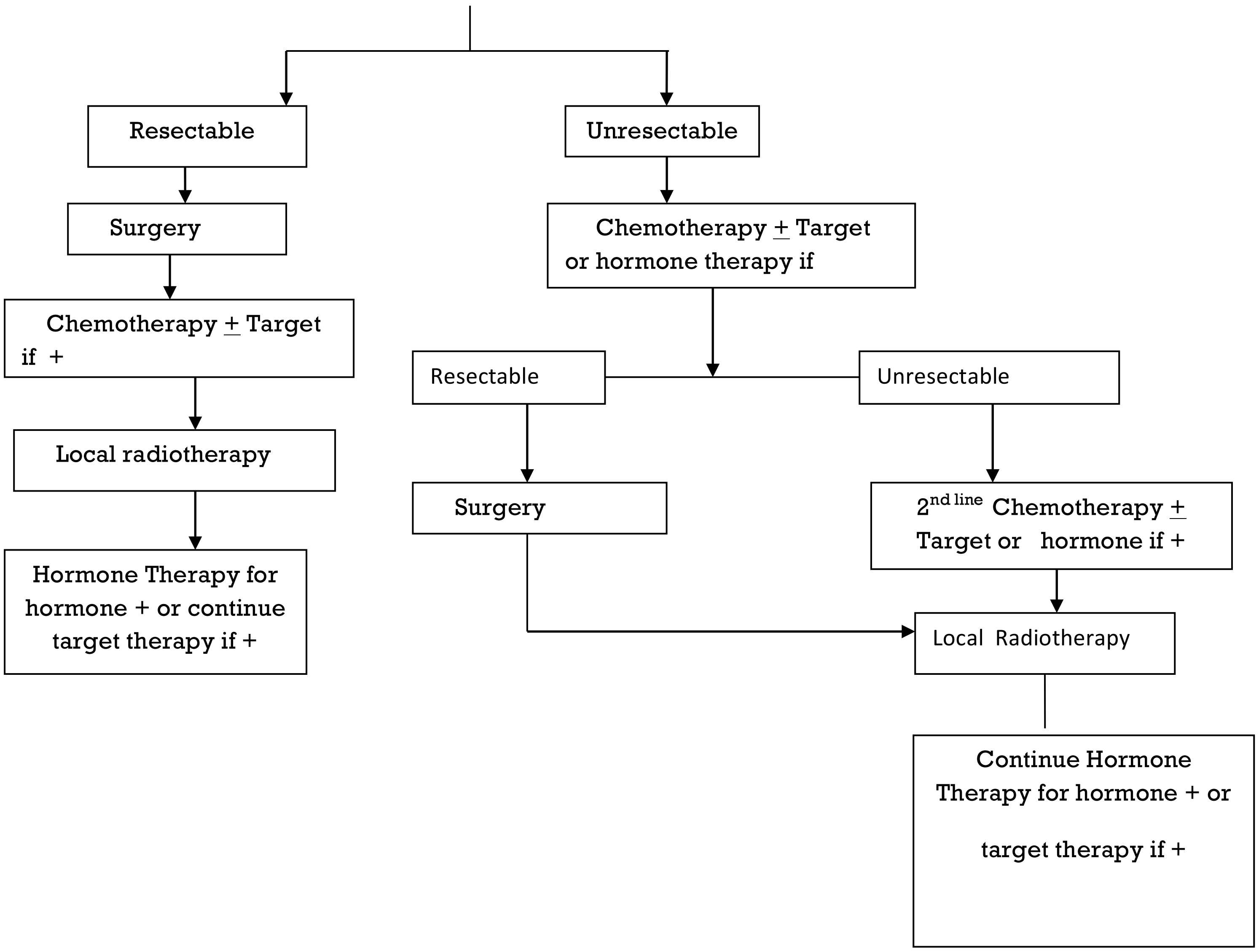

METASTATIC BREAST CANCER

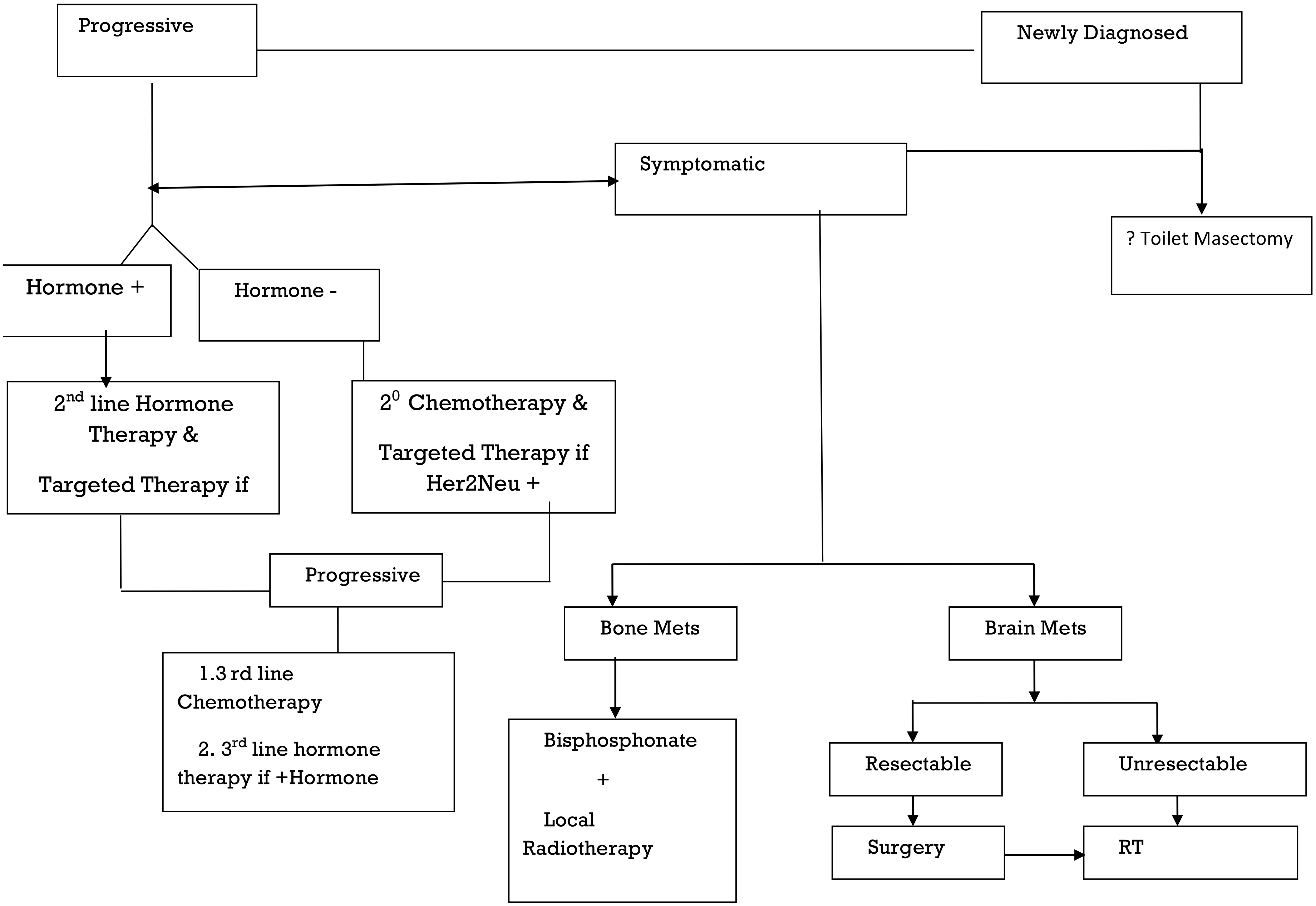

COLORECTAL CANCERS (CRC)

These guidelines are provided for the management of patients with adenocarcinoma of the colon and rectum confirmed by histology

Patient Evaluation and Staging

- History and examination
- FBC, BUE and Creatinine
- Radiological examination - CT Scan/MRI
- Serum Carcino-embryonic antigen

CHOICE OF THERAPY

The following factors may influence the choice of therapy:
Performance as per Karnofsky score[1]
Previous therapies

TREATMENT GOALS

The target of treatment is to achieve:
1. Cure or
2. Palliation

- Progression-free survival
- Disease-free survival
- Long term survival

Goals for palliation

- Controlling tumour growth
- Alleviating tumour rated symptoms
- Prolong life
- Improve quality of life

Note: The possibility of long-term survival should be entertained during the course of treatment in case there is a dramatic clinical response

TREATMENT OPTIONS

Localized disease:

Surgery followed by adjuvant chemotherapy if T4 and or any lymph node positive
Neo-adjuvant chemo-radiation followed by surgery and then adjuvant chemotherapy for rectal cancers or
Surgery upfront followed by chemo-radiation and adjuvant chemotherapy where indicated**.**

Metastatic disease:

In addressing the primary tumour in metastatic CRC:

---

[1] The Karnofsky score ranges from 100 to 0 and is an attempt to quantify cancer patients' general well being and activities of daily life. This measure is used to determine whether they can receive chemotherapy, whether dose adjustment is necessary, and as a measure for the required intensity of palliative care.

Decide whether the Primary CRC tumour requires surgical intervention (risk of obstruction, risk of GIT bleeding). These are the only indications for primary tumour resection in the metastatic setting.

NB: Effective neo-adjuvant chemotherapy can deal with the above risk in metastases.

Effective chemotherapy prevents the risk of major abdominal surgery, which could postpone the initiation of systemic therapy by several weeks leading to poorer outcomes.

Neo-adjuvant chemotherapy can be given for up to 2-3 cycles, followed by evaluation for resection of metastasis (liver, lung, ovary) and primary

Exposure to all chemotherapy drugs shows effectiveness in colorectal cancer by improvement in survival but should be weighed against quality of life.

MEDICINES FOR CRC

Folfox = Folinic acid + 5-Fluorouracil (bolus + infusional 5FU) + Oxaliplatin
Xelox = capecitabine + oxaliplatin
Folfiri = Folinic acid + 5-fluorouracil + Irinotecan
Bevicicumab or cetuximab with above combinations or capecitabine alone (cetuximab for patients with the gene KRAS wild type only)
Recommended single agents capecitabine, irinotecan or targeted therapies

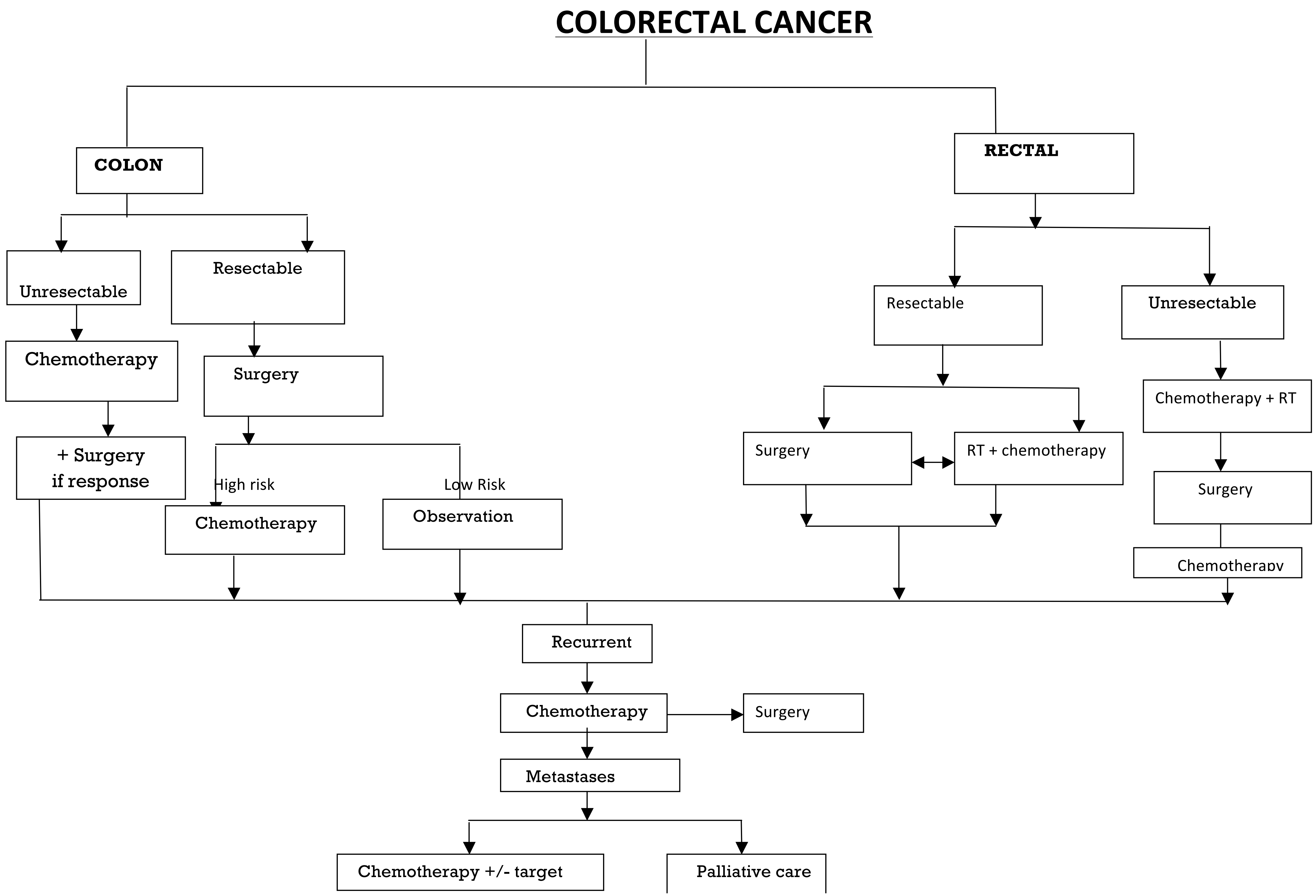
COLORECTAL CANCER
COLON
Unresectable
Resectable
Chemotherapy
Surgery
+ Surgery
if response
High risk
Low Risk
Chemotherapy
Observation
RECTAL
Resectable
Unresectable
Chemotherapy + RT
Surgery
RT + chemotherapy
Surgery
Chemotherapy
Recurrent
Chemotherapy
Surgery
Metastases
Chemotherapy +/- target
Palliative care

Prostate Cancer

In this guideline the focus is on patients with confirmed biopsy report of adenocarcinoma of the prostate gland.

DIAGNOSIS

Definition **-** Cancer that forms in tissues of the prostate gland.

Patient Evaluation and Staging

Tissue biopsy (pathological) plus elevated PSA (biochemical) and/or Bone and other organ metastasis (Radiological)

Prognostic stratification into risk groups

The stratification into risk groups is based on Gleason score (histopathology), Prostatic Specific Antigen (PSA) level (ng/ml) and Tumour staging.

Low risk	Gleason score ≤6, PSA ≤10, ≤T2A
Moderate risk	Gleason score =6, PSA 11- 20, T2b
High risk	Gleason score 8-10, PSA > 20, ≥ T2C

The presence of two intermediate risk factors is considered high risk; and the presence of one high risk factor regroups patient to high-risk category, for example PSA of 25ng/ml and Gleason score of 6 is considered a high-risk patient.

CHOICE OF THERAPY

The choice of therapy will be influenced by

- Previous surgery, radiotherapy and hormone therapy for prostate cancer
- Risk grouping
- Levels of testosterone (<50 microgram per deciliter or 17nmol/L)
- Performance status of the patient as determine by Karnofsky score[2] of less than 70
- Full patient evaluation and staging of disease

TREATMENT GOALS

- Improved quality of life as determined by the European Union's Cancer QOL questionnaire
- Control of tumour growth

---

[2] The Karnofsky score ranges from 100 to 0 and is an attempt to quantify cancer patients' general well-being and activities of daily life. This measure is used to determine whether they can receive chemotherapy, whether dose adjustment is necessary, and as a measure for the required intensity of palliative care.

- Alleviating disease symptoms
- Improve progression free survival or prolong life

TREATMENT OPTIONS

Localized tumours:

Intermediate to High-risk patients should receive radical prostatectomy or radiotherapy +/ - androgen deprivation therapy

High-risk patient should preferable receive androgen deprivation with local radiotherapy.

Recurrence or biochemical failure:

Radiotherapy is recommended if none previously received with or without androgen deprivation if testosterone is normal or secondary androgen deprivation if castrate.

Metastatic lesions:

Hormone sensitive (normal testosterone)

- Primary androgen deprivation with orchidectomy
- Secondary Androgen Deprivation Therapy using hormone treatment agents e.g. goserelin , bicluthetimide , flutamide, diethylstilboesterol, aminoglutetimide
- In high burden disease consider chemotherapy with androgen deprivation.

Hormone refractory or non responsive

- Stilbesterol
- Chemotherapy with docetaxel and prednisolone, carbitaxel, abiterone , enzulethemide
- Bisphosphonates - Zoledronic acid or ibandronate for bone metastasis.

Metastatic lesions may be treated with radiotherapy.

PROSTATE CANCER

LOCALISED

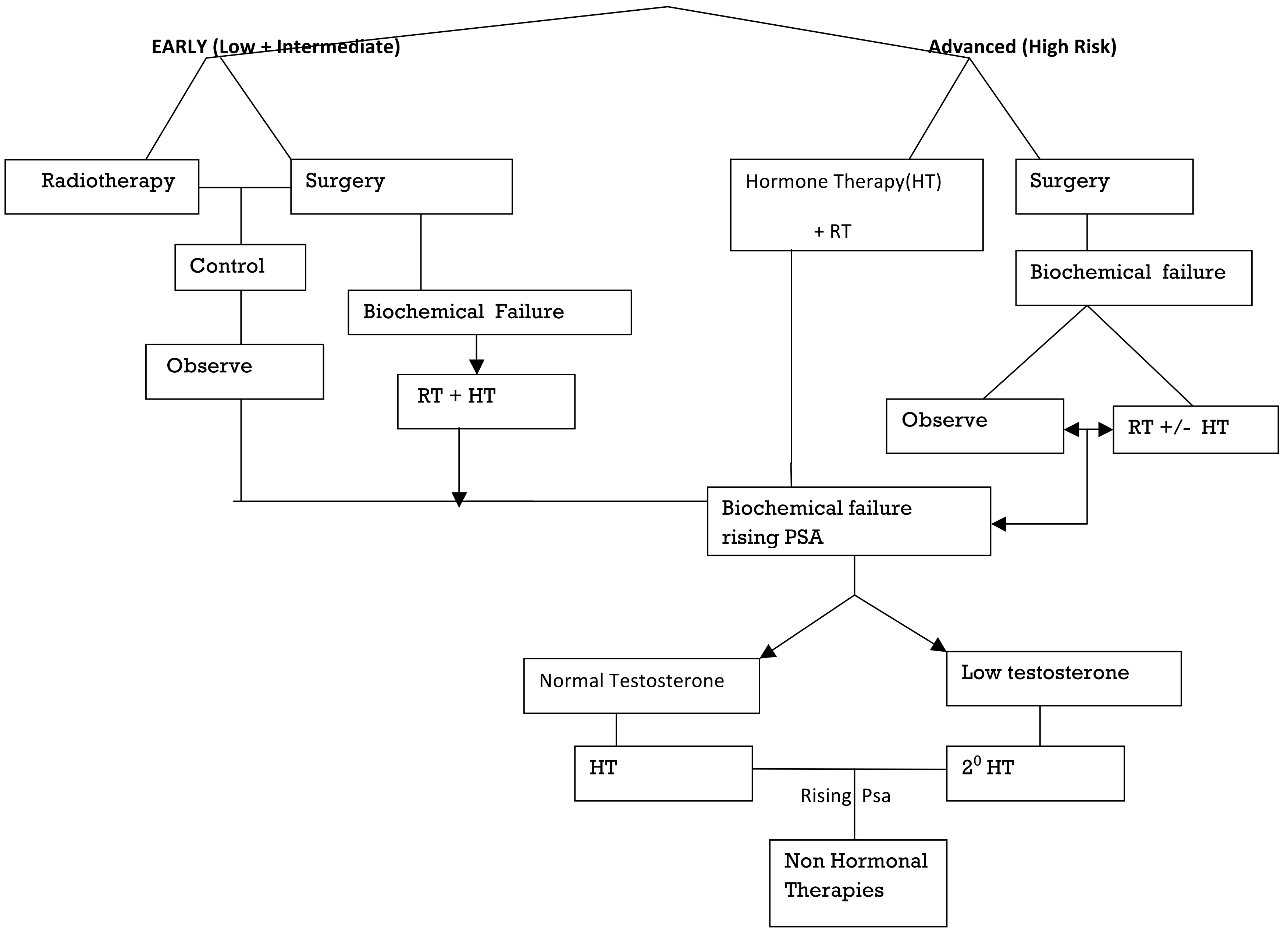

METASTATIC PROSTATE

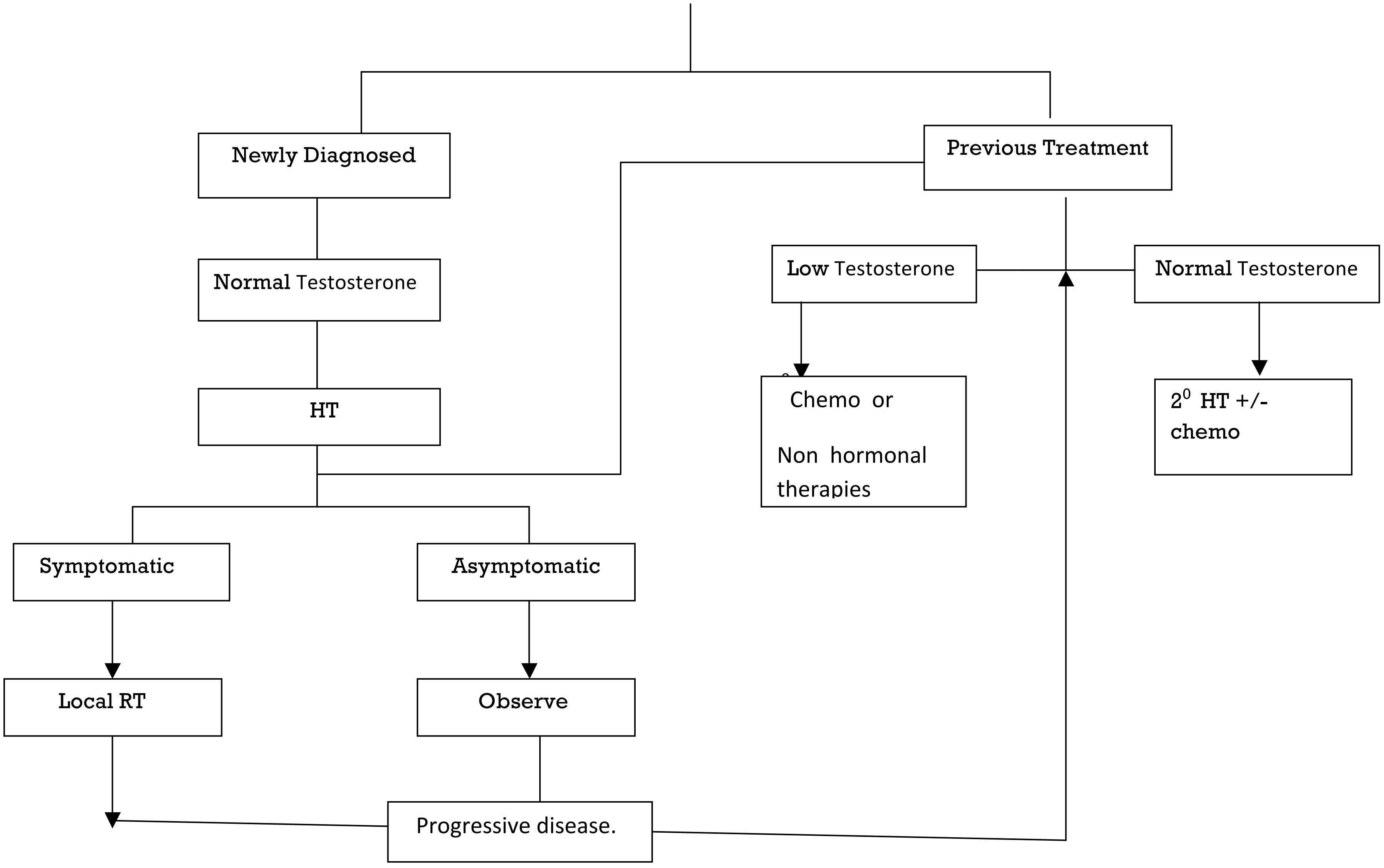

Supplement: Supplementary file 2 [file gatesopenres-2-13889-s0001.tgz › 410bf10f-812b-418c-b376-cb2c39f662dc.zip!/TREATMENT GUIDELINES/Cancer Treatment Guidelines Final pdf.pdf]
