# Supplementary material for: A multi-center prospective cohort study to evaluate the effect of differential pricing and health systems strengthening on access to medicines and management of hypertension and diabetes in Ghana: A study protocol
Source: Gates Open Res. 2018 May 3;2:6. Originally published 2018 Feb 7. [Version 2] doi: 10.12688/gatesopenres.12797.2 (PMC5941285; doi:10.12688/gatesopenres.12797.2)
Supplement: Supplementary file 2 [file gatesopenres-2-13889-s0001.tgz › 410bf10f-812b-418c-b376-cb2c39f662dc.zip!/TREATMENT GUIDELINES/FINALMANUAL_PPB.pdf]

TABLE OF CONTENTS

Table of contents 1
Definitions..... 5
Acronyms and Abbreviations 7

CHAPTER 1: INTRODUCTION 8
1.1 Introduction..... 8
1.1.1 Objectives of the Manual 8
1.1.2 Application and Limitations of Manual 8
1.2 Exceptions to the Manual..... 9
1.2.1 Reasons for Exceptions 9
1.3 Revisions to the Manual..... 9
1.3.1 Reasons for Revision..... 9
1.3.2 Request for Revisions by a Procurement Entity..... 10
1.4 Use of Standard Procurement Forms..... 10
1.4.1 SPF 1 – Procurement Requisition..... 10
1.4.2 SPF 2 – Submission for Approving Authority's Review 10
1.4.3 SPF 3 – Evaluation Report (Goods and Works) 10
1.4.4 SPF 4 – Evaluation Report (Services) 11
1.4.5 SPF 5 – Monthly Procurement Report 11
1.5 Procurement Numbering System 11
1.5.1 Purpose:..... 11
1.5.2 Structure of the Procurement Number..... 11
1.5.3 Use of Procurement Numbers..... 11
1.6 Public Procurement Principles..... 12
1.7 Transparency, Accountability, and Ethics..... 12
1.7.1 Reporting..... 12
1.7.2 Examples of Unethical Conduct 13
1.7.3 Obligations of Public Servants..... 13
1.7.4 Responsibilities of Officers..... 13
1.7.5 Responsibilities of the Private Sector..... 14
1.7.6 Sanctions for Non-compliance with Ethical Standards..... 14
1.8 Internal and Independent Monitoring..... 15
1.8.1 Reporting and Implementation of Recommendations..... 15
1.9 Correspondence/Communications..... 15
1.10 Documentation/Records Management 15
1.10.1 Documentary Records 15
1.10.2 Filing/Record Management System 16
1.10.3 Procurement Unit Records Management 16

CHAPTER 2: PROCUREMENT STRUCTURES..... 18
2.1 Public Procurement Board (PPB)..... 18
2.2 Procurement Entity..... 18
2.3 The Head of Procurement Entity..... 20
2.3.1 Approval of Contract Awards by the Head of Entity..... 20
2.4 Procurement Unit..... 21
2.5 Entity Tender Committee 21
2.6 Tender Evaluation Panels..... 23
2.6.1 Composition of Tender Evaluation Panels..... 23
2.6.2 Proceedings of Tender Evaluation Panels..... 23
2.7 Tender Review Boards 24
2.8 Originating Departments, Projects or Units..... 25
2.9 Stores Departments..... 25
2.10 Inspection/Takeover Teams 26

CHAPTER 3: PROCUREMENT PLANNING AND BUDGETING..... 27
3.1 Procurement Planning 27
3.2 The Budget Preparation Process..... 27
3.2.1 Contents of the Procurement Plan 28
3.2.2 Head of Procurement Entity Actions..... 28

3.2.3 Procurement Unit Actions 29
3.2.4 Stores Departments Actions 29
3.2.5 Heads of Department, Units, Projects and Programmes 30
3.3 Guidelines for Determining Completion Timescales 31
3.3.1 Estimated lead times for ICT (Goods) 31
3.3.2 Estimated lead times for NCT (Goods) 31
3.3.3 Estimated lead times for ICT (Works) 31
3.3.4 Estimated lead times for NCT (Works) 31
3.3.5 Estimated lead times - Request for Quotation 32
3.4 Plan Monitoring and Updating 32

CHAPTER 4: PROCUREMENT OF GOODS 33
4.1 Definition of Goods 33
4.2 Procurement Methods 33
4.2.1 International Competitive Tendering 33
4.2.2 National Competitive Tendering 33
4.2.3 Restricted Tendering 33
4.2.4 Two-Stage Tendering 34
4.2.5 Single Source 34
4.2.6 Request for Quotations (RFQ) 34
4.3 Specification of Requirement 34
4.4 Pre-qualification of Tenderers for Goods 35
4.4.1 Pre-qualification Criteria 35
4.4.2 Advertisement for pre-qualification 36
4.4.3 Pre-qualification evaluation 36
4.5 Tendering Procedures 36
4.5.1 Standard Tender Document 36
4.5.2 Preparation of Tender Document 37
4.5.4 Invitation Advertisements for Competitive Tenders (ICT and NCT) 38
4.5.5 Contents of the Advertisement 39
4.6 Sale of Tender Documents for Competitive Tenders 39
4.7 Issue of Invitations for Restricted Tender 40
4.8 Issue of Requests for Quotations 40
4.8.1 Opening of Standard Requests for Quotations 40
4.8.2 Evaluation of RFQ 41
4.8.3 Award of Contract 42
4.9 Tender Clarifications 42
4.10 Extension of Tender Closing Date 42
4.11 Rejection of Tenders Before Tender Opening 43
4.12 Receipt of Tenders 43
4.13 Opening of Tenders 44
4.14 Tender Evaluation 46
4.14.1 Preliminary Examination of Tenders 46
4.14.2 Responsiveness of Tenders 47
4.14.3 Financial Evaluation of Tenders 49
4.14.5 Post -Qualification (Repeat Tender Qualifications) 50
4.14.6 Evaluation Reports and Recommendations 50
4.15 Review and Approval of Evaluation Report 52
4.16 Extension of Tender Validity 52
4.18 Award of Contract 54
4.18.1 Preparation of a Contract 54
4.18.3 Notification to Unsuccessful Tenderers 56
4.18.4 Return of Tender Securities 56
4.19 Contract Management 56
4.19.1 Contract Effectiveness 56
4.19.2 Contract Supervision and Administration 57
4.20 Receipt and Inspection of Goods 57
4.20.1 Collecting Goods from the Port of Entry 57
4.20.2 Delivery of Goods 58
4.20.3 Inspection of Goods 58
4.20.4 Issue to the User Department by Stores 58

4.21 Payment for Goods 59
4.21.1 Payments by Letter of Credit 59
4.22 Delays in Performance 60
4.23 Resolution of Contractual Disputes 61
4.24 Termination of the Contract 61
4.25 Contract Amendment 62

CHAPTER 5: PROCUREMENT OF WORKS 63
5.1 Definition of Works 63
5.2 Procurement Methods 63
5.2.1 International Competitive Tendering 63
5.2.2 National Competitive Tendering 63
5.2.3 Restricted Tendering 63
5.2.4 Two-Stage Tendering 64
5.2.5 Single Source 64
5.2.6 Request for Quotations (RFQ) 64
5.3 Specification of Requirements 64
5.4 Pre-Qualification of Contractors 65
5.4.1 Pre-qualification Criteria 65
5.4.2 Advertisement for pre-qualification 65
5.4.3 Pre-qualification evaluation 65
5.4.4 Advertisement for pre-qualification 66
5.4.5 Pre-qualification document 66
5.5 Tendering Procedures 67
5.5.1 Standard Tender Document 67
5.5.2 Preparation of Tender Document 67
5.5.4 Invitation Advertisements for Competitive Tenders (ICT and NCT) 69
5.5.5 Contents of the Advertisement 69
5.6 Sale of Tender Documents for Competitive Tenders 70
5.7 Issue of Invitations for Restricted Tender 70
5.8 Issue of Requests for Quotations 70
5.8.1 Opening of Standard Requests for Quotations 71
5.8.2 Evaluation of Contract for RFQ 72
5.9 Tender Clarifications 72
5.10 Extension of Tender Closing Date 73
5.11 Rejection of Tender Before Opening 73
5.12 Receipt of Tenders 73
5.13 Opening of Tenders 74
5.14 Tender Evaluation 76
5.14.1 Preliminary Examination of Tenders 76
5.14.2 Responsiveness of Tenders 77
5.14.3 Financial Evaluation of Tenders 79
5.14.4 Evaluation Reports and Recommendations 80
5.15 Review and Approval of Evaluation Report 82
5.16 Extension of Tender Validity 82
5.17 Award of Contract 83
5.17.1 Award Notification 84
5.17.2 Notification to Unsuccessful Tenderers 85
5.17.3 Return of Tender Securities 85
5.18 Contract Management 85
5.18.1 Contract Effectiveness 85
5.19 Contract Supervision And Administration 86
5.20 Initial and Final Takeover of Works 87
5.20.1 Defects Liability Period 87
5.21 Payment for Works 87
5.22 Delays in Performance 87
5.23 Resolution of Contractual Disputes 88
5.24 Termination of the Contract 89
5.25 Contract Amendment 89

CHAPTER 6: THE SELECTION AND EMPLOYMENT OF CONSULTANTS.....91
6.1 Definition of Consultancy Service.....91
6.2 Special Features of Consultancy service.....91
6.2.1 Merit-Point Systems.....91
6.2.2 Two-envelope Tendering.....92
6.3.1 Quality and Cost Based Selection (QCBS).....92
6.4 Use of Individual Consultants.....95
6.5 The Steps Of The Procurement Process.....95
6.5.4 EOI Criteria.....98
6.5.5 Advertisement for Expressions of Interest.....98
6.5.6 Evaluation of EOI and Short listing.....99
6.5.8 Technical Evaluation Criteria.....102
6.5.9 Minimum Technical Score.....103
6.5.10 Financial Evaluation Criteria.....103
6.5.11 Weighting of Technical and Financial Scores.....103
6.6 Contract Agreement for Employment of Consultants.....103
6.6.1 Lump Sum (Fixed Price) Contracts.....104
6. 6.2 Time-Based Contract......104
6.6.3 Retainer and/or Contingency (Success) Fee Contract......104
6.6.4 Percentage Contract......105
6.6.5 Definite Delivery Contract (Price Agreement)......105
6.7 Tender And Performance Security.....105
6.8 Receipt And Opening Of Technnical Proposals.....106
6.9 Evaluation Of Technical Proposals.....107
6.10 Opening Of Financial Proposals.....108
6.11 Evaluation of Financial Proposals.....110
6.12 Negotiation of Contract.....111
6.13 Award of Contract.....112
6.13.2 Notification to Unsuccessful Tenderers.....113
6.14 Contract Management.....114
6.14.1 Contract Supervision and Administration.....114
6.14.2 Payment for Consultant Services.....114
6.14.3 Resolution of Contractual Disputes.....114
6.14.4 Termination of the Contract.....115
6.14.5 Contract Amendment.....115

CHAPTER 7 PROCUREMENT REPORTING, MONITORING & EVALUATION.....117
7.1 Procurement Reporting.....117
7.1.1 Section A: Procurements in Process above GHC 50 million.....117
7.1.2 Section B: Contracts Signed or Procurements Cancelled above GHC 50 million.....118
7.1.3 Section C: Contracts Completed above GHC 50 million.....118
7.1.4 Section D: Minor Procurements under GHC 50 million.....118
7.2 Procurement Monitoring and Evaluation.....119
7.2.1 Sources of Information.....119
7.2.2 Action by the Public Procurement Board on Receipt of Form SPF5.....119
7.2.3. Other Monitoring and Evaluation Activities by the PPB.....120
7.2.4 Action by the Procurement Entity.....121

CHAPTER 8: COMPLAINTS ON THE PROCURMENT PROCESS.....122
8.1 Review of Procurement Process.....122
8.1.1 Review by Head of Procurement Entity.....122
8.1.2 Administrative Review by the Public Procurement Board.....122
8.1.3 Rules Applicable to Review Proceedings.....124
8.1.4 Suspension of procurement proceedings.....124

CHAPTER 9: GUIDELINES FOR DISPOSAL OF GOODS & EQUIPMENT.....126
9.1 Introduction.....126
9.2 Definitions.....126
9.3 Authority to Dispose.....126
9.4 Disposal Options.....127
9.4.1 Transfer to Government Department or Other Public Entity......127

9.4.2 Sale by Public Tender. 127
9.4.3 Sale by Public Auction. 128
9.4.4 Destruction, Dumping or Burying 128
9.5 DETERMINATION OF MARKET VALUE OF ASSETS 128
9.6 REVENUE FROM SALES 129

APPENDICES 130

DEFINITIONS

The terms used in this Manual have the following meanings assigned to them.

WORD OR TERM	DEFINITION/DESCRIPTION
Head of Procurement Entity	The Vote Controller or highest-ranked executive officer in a Procurement Entity.
Asset Disposal	The formal process of disposal of assets which are no longer of use to a Procurement Entity.
Authorised Person	Any person who has been granted the power to authorise a transaction or otherwise commit the Procurement Entity.
Code of Ethics	A statement of the standards of practice and conduct to be followed by all Public officials and officials of Government owned organisations in procurement activities.
Development Partner	An International Financing Institution (such as the World Bank or African Development Bank) providing external funds to the Government
Evaluation Panel	A panel established by the Tender Committee to undertake evaluation and ranking of tenders, proposals and quotations for procurement.
INCOTERMS	A set of trade terms (13 in number) for which precise definitions have been set down by the International Chamber of Commerce (Paris) which when used in a contract, clearly indicate the rights and obligations of the procuring entity and the supplier (e g CIF, CIP, FOB).
Originating Officer	An officer of the Procurement Entity having formal authority to initiate a procurement process.
Procurement Entity	A Procurement Entity is an organization or person that has legal or administrative mandate for procurement purposes.
Procurement Officer	An officer of the Procurement Entity having formal authority to undertake procurement activity.
Request for Proposals	The term commonly used for tenders in the procurement of consultancy services, where the Procurement Entity invites proposals from short listed consultants.

WORD OR TERM	DEFINITION/DESCRIPTION
Request for Quotations	A simple procurement procedure for the purchase of low value goods, works and simple non-consultancy services.
Tender Committee	A team of senior officers in an entity with responsibility of ensuring that every stage of the procurement activities are followed in accordance with the Act.
Tender Opening Committee	A committee established by a Entity Tender Committee to conduct the opening of tenders and tenders for procurement.

ACRONYMS AND ABBREVIATIONS

ACB	Anti-Corruption Bureau
BOQ	Bill of Quantities
CFR	Cost and Freight
CIF	Carriage Insurance and Freight
CIP	Carriage and Insurance Paid
CPT	Carriage Paid to
EOI	Expression of Interest
EXW	Ex-Works
FBS	Fixed Budget Selection
FOB	Free on Board
GCC	General Conditions of Contract
ISO	International Standards Organisation
ITT	Instructions to Tenderers
LC or L/C	Letter of Credit
LCS	Least Cost Selection
GHC	Cedis
LOI	Letter of Invitation
MOF	Ministry of Finance
MTEF	Medium Term Expenditure Framework
QBS	Quality Based Selection
QCBS	Quality and Cost Based Selection
PIU	Project Implementation Unit
PPB	Public Procurement Board
PPM	Public Procurement Manual
RFP	Request for Proposals
RFQ	Request for Quotations
STD	Standard Tender Document
SCC	Special Conditions of Contract
SPF	Standard Procurement Form
TOR	Terms of Reference

CHAPTER 1: INTRODUCTION

1.1 INTRODUCTION

The Government of Ghana enacted the Public Procurement Act, 2003, (Act 663) in December, 2003. The Act became effective/operational on 27th August 2004 and Public Procurement in Ghana therefore is subject to the Act and its implementing Regulations and Administrative Instructions. The Act and Regulations provide the general rules governing Public Procurement. This manual provides guidelines and step-by-step procedures to assist Procurement Entities to undertake public procurement in accordance with the Act.

1.1.1 Objectives of the Manual

This Manual details the standards and procedures to be followed in the procurement of goods, works and services within the public sector, and also includes a section for guidance on asset disposal procedures. These standards, policies and procedures are designed to:

- provide uniform procedures for the procurement of goods, works and services and for asset disposal;
- ensure transparency and accountability in all operations, and consistency with the guidelines of Development Partners where necessary;
- ensure the consistent application of the provisions of the Act and Regulations; and
- promote the consistent application of best procurement practices and international standards.

1.1.2 Application and Limitations of Manual

This Manual applies to the procurement operations of all Government organisations as defined in the Act section 2 and the Regulations.

In the event that Public Sector entities have their own specialist procurement regulations and procedures manuals, the manual shall be subjected to formal certification and approval by the Public Procurement Board.

The Manual covers the standard procurement cycle from procurement planning and initiation of procurement to final delivery or performance and contract completion. It will help to:

- remove or minimise the need for making new decisions every time a comparable situation arises;
- provide a point of reference against which principles and practices can be evaluated; and
- serve as a training manual for procurement practitioners in the public sector and provide guidance based on best international practices.

Consistent application of the provisions and procedures of the Manual throughout the public service is essential to achieve improved efficiency,

transparency, uniformity of documents and decisions, and reduced costs of procurement for the Government.

It is intended that the Manual will be subjected to continuous review by the Public Procurement Board to reflect amendments in legislation, changing needs, the commercial environment, and the adoption of new improved procedures and practices.

1.2 EXCEPTIONS TO THE MANUAL

1.2.1 Reasons for Exceptions

Exceptions to the provisions of this Manual may arise for a number of reasons including the following:

- a special situation, such as an extreme emergency;
- one-off requirements introduced as special provisions in a loan or credit facility which are not otherwise covered in this Manual and do not constitute a revision to the Manual,
- the necessary use of non-standard tender documents for specialist procurements;
- new legislation or government policy change which causes any part of this Manual to be inconsistent with the new provisions until an appropriate revision has been approved.

Exceptions should normally be of a short-term and one-off nature. Where they are recurrent, or become repetitive, then the Public Procurement Board shall decide whether there is a need to introduce revisions in the Manual to reflect the necessary change, in accordance with Section 1.3 of this Manual.

Where an exception is considered essential, the Head of the Procurement Entity may submit a formal request to the PPB detailing the circumstances and including a full justification. The PPB may approve such requests by issue of a specific waiver or reject the request.

Exceptions and waivers granted shall be carefully reviewed in the monitoring activities of the PPB and subject to audit.

The PPB will review all exception requests to determine whether the relevant provisions in the Manual need to be amended.

1.3 REVISIONS TO THE MANUAL

1.3.1 Reasons for Revision

A number of factors may necessitate a need for revision of this Manual including:

- new legislation;
- policy changes by Government or a funding body;
- introduction of new improved practices, policies or procedures;
- removal of outdated practices, policies or procedures;
- lessons gained from practical experience.

1.3.2 Request for Revisions by a Procurement Entity

Where a revision to the Manual is deemed necessary, a formal request may be submitted to PPB in accordance with the following procedure:

- ❑ The request shall be prepared and approved by the relevant Tender Committee, and authorised by the Head of Procurement Entity.
- ❑ The request shall state the purpose of the revision and provide a comprehensive justification, together with the precise changes suggested to the relevant text of the Manual.
- ❑ The PPB will formally consider each request, seek legal and procurement practice advice and may agree to a general modification of the Manual, a specific exemption to be granted to the requesting Procurement Entity, or may reject the request giving reasons.

1.4 USE OF STANDARD PROCUREMENT FORMS

To assist in the adoption and use of standard practices and procedures in public sector procurement, the following Standard Procurement Forms (SPFs) (see appendices) are introduced within this Manual:

1.4.1 SPF 1 – Procurement Requisition

This form establishes the preliminary specification, the budget availability and authority for the procurement, and requires the allocation of a Procurement Number to be used for procurement tracking and monitoring.

1.4.2 SPF 2 – Submission for Approving Authority's Review

This Form has four Sections as follows:

- ❑ SPF2 A – Request for Approval of Specifications, Procurement Method and Tender Documents.
- ❑ SPF2 B – Request for Approval of Evaluation Report and Contract Award Recommendations
- ❑ SPF2 C – Request for Approval of Contract Document and Contract Signature
- ❑ SPF2 D – Request for Contract Amendment

The purpose of this Form is to standardise the format of submissions to the approving authority, to act as a checklist of information to be provided to the Committee, and to ensure that submissions are presented with the approval of an authorised responsible officer. Use of the Form eliminates the need for individual covering letters to be prepared on each application to the Tender Committee.

1.4.3 SPF 3 – Evaluation Report (Goods and Works)

Standard evaluation format for the procurement of goods and works.

The purpose of this form is to standardise and improve the content of evaluation reports submitted for approval.

1.4.4 SPF 4 – Evaluation Report (Services)

Standard evaluation format for technical and financial evaluations of consultant services. This format has two Sections for separate Technical and Financial Evaluations to allow for "two-envelope" tendering.

1.4.5 SPF 5 – Monthly Procurement Report

Monthly report to be submitted to the Public Procurement Board by each Tender Committee.

1.5 PROCUREMENT NUMBERING SYSTEM

1.5.1 Purpose:

The purpose of the Procurement Numbering system is to ensure each requirement is given a unique identification to assist in the tracking, monitoring and audit of procurement, and in the tracing and maintenance of records and files. The Procurement Number is assigned at the requisition stage and will form the basis for document filing and management information systems.

Procurement Numbers are initiated and allocated for a procurement package and entered into a register maintained by the procurement unit. Numbers must not be re-used, even if the original procurement is cancelled.

1.5.2 Structure of the Procurement Number

The basic Procurement Number shall identify as a minimum:

- ❑ The Procurement Entity
- ❑ The Department or Project.
- ❑ The Financial Year (e.g. 2005).
- ❑ A unique Sequence Number for each requirement.

The Public Procurement Board will issue further detailed guidelines on the construction of procurement numbers to ensure compatibility with existing numbering systems for procurement planning, MTEF and the pre-numbered Activity and Expenditure Initiation (A&EI) standard form.

1.5.3 Use of Procurement Numbers

The procurement number must appear on all correspondence and documents related to the procurement.

1.6 PUBLIC PROCUREMENT PRINCIPLES

The overall objective of the public procurement system is to provide value for money to the Government by ensuring that public funds are spent in a transparent, efficient and fair manner.

This Manual incorporates policy provisions and procedures to promote transparency, accountability and ethics in the operation, management and reporting of procurement and asset disposal. All Public Servants shall consistently apply these policies and procedures, together with professional judgement and good management

1.7 TRANSPARENCY, ACCOUNTABILITY, AND ETHICS

The procurement system must ensure value for money in the procurement of goods, services and works. The procurement system aims at procuring goods, services and works of the right quality, at the right price, at the right time and at the right place through an open competitive tendering process. The Government is entrusted with public funds to provide services to the public. The use of public money must be conducted in a transparent and open manner, allowing stakeholders and the general public access to information on procurement actions by the Government as well as a means to control and audit all procurement cases. Furthermore, the rules governing public procurement must be readily available to all interested parties.

The Government requires that:

- all public officials and practitioners of procurement shall be held accountable and responsible for their actions;
- all suppliers, contractors and consultants will be treated fairly and given equal opportunity to obtain contracts with the Government;
- procurement shall be done in the most efficient manner, upholding the principles of value for money, transparency and fairness
- funds will be used solely for the purposes for which they have been entrusted;
- appropriate procedures of the Government or the Development Partners are applied;
- all transactions are properly authorised and fully supported by written records;
- value for money can be demonstrated by comparison with market rates; and
- an appropriate Code of Ethics is followed by all staff involved in the procurement process.

1.7.1 Reporting

A Public Servant has a duty to report any unethical conduct by a colleague, a tenderer or a supplier to his superiors or to the auditors.

1.7.2 Examples of Unethical Conduct

The following are examples of the type of conduct prohibited by the Code of Ethics:

- Revealing confidential or "inside information" either directly or indirectly to any tenderer or prospective tenderer;
- Discussing a procurement with any tenderer or prospective tenderer outside the official rules and procedures for conducting procurements;
- Favouring or discriminating against any tenderer or prospective tenderer in the drafting of technical specifications or standards or the evaluation of tenders;
- Destroying, damaging, hiding, removing, or improperly changing any official procurement document;
- Accepting or requesting money, travel, meals, entertainment, gifts, favours, discounts or anything of material value from tenderers or prospective tenderers;
- Discussing or accepting future employment with a tenderer or prospective tenderer;
- Requesting any other Public Servant or Government official representing the Procurement Entity in a procurement to violate the public procurement rules or procedures;
- Ignoring evidence that the Code of Ethics has been violated by a member of the Tender Committee, Public Servant or other employee or representative of the Procurement Entity;
- Ignoring illegal or unethical activity by tenderers or prospective tenderers, including any offer of personal inducements or rewards.

1.7.3 Obligations of Public Servants

The guiding principles of ethical behaviour are impartiality, independence and integrity. Ethical behaviour must be promoted and supported by appropriate systems and procedures, but systems and strict adherence to procedures alone cannot create an ethical and accountable procurement function.

Public Servants involved in procurement are obliged to follow the Civil Service Code and the regulations governing public procurement. Moreover, it is absolutely essential that all procurement activities are carried out in a manner above reproach, with complete impartiality and with no preferential treatment. The procurement process must allow suppliers, contractors and consultants to compete for business on a fair basis. Public officials associated with the procurement function, therefore, are responsible for protecting the integrity of the procurement process and maintaining fairness in the Government's treatment of all suppliers, contractors and consultants.

1.7.4 Responsibilities of Officers

Senior officers in a Procurement Entity have responsibility to:

- ❑ Provide instructions and guidance to staff, especially where they may be exposed to opportunities for accusations of fraud or corruption;
- ❑ Encourage staff to follow ethical principles and practices and create an environment where transparency and openness are the standard;

- ❑ Ensure that all staff comply with instructions and guidelines, including the maintenance of documented records;
- ❑ Set appropriate levels of financial delegation, and ensure a proper separation and rotation of duties.

Junior officers in a Procurement Entity have responsibility to:

- ❑ Follow management instructions and procedural guidelines;
- ❑ Be alert to and report any indications of unethical behaviour;
- ❑ Seek guidance from a manager or auditor on any concerns that may arise.

1.7.5 Responsibilities of the Private Sector

The Private Sector is not exempt from responsibility to act ethically and transparently in procurement proceedings with the Government of Ghana. This message is to be reinforced by requiring tenderers to sign a formal declaration before participating in any procurement process:

"I hereby certify that I and all officers of the Company which I represent will uphold the highest ethical standards in doing business with the Government of Ghana. We will not attempt to bribe government officials with money or other goods or services; we will not request favourable treatment and we will not seek information about competitors or other information regarding the procurement process. I understand that if I, or other officers of the Company which I represent are found to have breached this commitment, that sanctions will be made against the Company, including being debarred from doing business with the Government of Ghana for a period of 5 years."

1.7.6 Sanctions for Non-compliance with Ethical Standards

Due to the special and additional public trust placed on procurement professionals as custodians of public funds, a special and additional level of rules, compliance, obligations and sanctions governing the activities of all officials engaged in any way in public procurement regardless of their rank or function will be applied.

Adherence to the provisions of the Act and Regulations by Public Officials is obligatory and failure to do so will be considered an offence subject to sanctions. The level of sanctions will be published separately and will be graduated depending on the offence. Any official suspected of non-adherence to the rules and regulations will be suspended and his or her benefits, including salary, withheld pending investigation. Officials found misappropriating government funds, regardless of the value, will be summarily dismissed. Other offences involving non-adherence to the rules and regulations, including negligence and irresponsibility will result in sanctions as specified in the Act.

Any supplier, contractor or consultant who attempts to influence a procurement process, or the administration of a contract by any unfair method, will be subject to sanctions. This may include debarment of the company from Government contracts for a period of five years, in addition to any remedies that may be sought in a court of law.

1.8 INTERNAL AND INDEPENDENT MONITORING

Each Procurement Entity shall ensure that regular internal monitoring of procurement activity is undertaken in accordance with the principles and provisions of the Act, and the Manual, to ensure the overall effectiveness of procurement.

Regular monitoring of procurement in a Procurement Entity will be conducted by the PPB. The PPB will assess issues of professional efficiency and effectiveness in procurement, may call for detailed assessments or explanations on individual procurements, and will issue reports of findings.

1.8.1 Reporting and Implementation of Recommendations

All reports arising from such monitoring activities shall be communicated to the Head of the Procurement Entity for appropriate action. Additionally, independent monitoring and external audit reports may be communicated to Development Partners or other funding agencies where the report covers contracts and/or projects funded by them.

On receipt of a monitoring report, the Procurement Entity shall take appropriate action to:

- ❑ remedy any adverse findings, weaknesses or anomalies identified in the report;
- ❑ implement any recommendations for improvement;
- ❑ impose the specified sanctions on individual Public Officials.

1.9 CORRESPONDENCE/COMMUNICATIONS

Correspondence and communications are essential to the management of relationships with suppliers and other bodies but communications issued by a Procurement Entity may commit the Procurement Entity or bind it to a course of action with legal, contractual or financial implications. Therefore, all procurement correspondence and communications issued by a Procurement Entity shall be made by persons authorised in accordance with their duties and responsibilities.

1.10 DOCUMENTATION/RECORDS MANAGEMENT

1.10.1 Documentary Records

Documentary records, both in print or electronic format, are essential for efficient and effective management of activities, provide evidence in support of decisions and actions taken, and provide an audit trail for verification of transparency, accountability and effectiveness. Efficient records management is essential to ensure effective storage, retrieval and use with due regard to security, integrity and confidentiality.

All officers shall ensure they obtain, retain and maintain appropriate documentation supporting the activities for which they are responsible.

Senior officers involved in approval or authorisation shall ensure that any transactions they approve or authorise are backed by appropriate supporting documentation.

1.10.2 Filing/Record Management System

Procurement Units have a general responsibility for maintaining Procurement Dossiers and Contracts Registers, but each Procurement Entity must ensure that complete documentation is maintained in respect of all procurement activities and for contracts and agreements entered into.

Responsibility for the official maintenance of record files, authority to access files, and co-ordination of management and follow-up actions must be clearly defined in each Procurement Entity to avoid:

- partial and incomplete records being held by several different departments and individuals;
- unauthorised access to records; or
- failure to take any necessary action at the right time.

1.10.3 Procurement Unit Records Management

All documents regarding a particular procurement shall be kept for future reference and monitoring and control purposes. The recommended filing structure for a record of procurement shall include the following documentation:

- Original Request from Originating Officer
- Correspondence with Originating Officer
- Correspondence with Suppliers, Contractors and Consultants/ (pre-order only)
- Internal Correspondence within Procurement Unit
- Requests for Expressions of Interest
- Shortlist or Advertisement
- Tender documents and Specifications/Terms of Reference
- Correspondence with suppliers relating to Tender
- Tenders, Quotations or Pro-forma invoices received
- Tender opening records
- Record of Tender Securities and release
- Evaluation Report
- Submission to the relevant authority & Minutes of proceeding
- Notice of Contract Award and publication
- Contract/Purchase Order & Acknowledgement of receipt
- Delivery/Executions Documentation
- Inspection and Acceptance Reports
- After sales warranty and performance claims
- Copy of Record of Commitment of Funds (A&EI Form)
- Copy of Invoices or Certificates
- Copy of Payment Vouchers

- ❑ Record of Retentions and release
- ❑ Performance Security and release.

CHAPTER 2: PROCUREMENT STRUCTURES

2.1 PUBLIC PROCUREMENT BOARD (PPB)

The Public Procurement Board acts as the statutory advisory and co-ordinating body on procurement. All Procurement Entities, Tender Committees and Tender Review Boards are required to abide by the technical guidelines and regulatory instructions issued by the PPB.

The PPB has responsibility for the overall co-ordination, direction and development of Government procurement practices and procedures. The Board is entrusted with 21 functions under Section 3 of the Act which may be summarised as follows:

- Formulation/Development of public procurement policy, setting standards, rules, instructions and other regulatory instruments on public procurement
- Monitoring compliance with requirements established by legislation;
- Obtaining and ensuring dissemination of information relating to public procurement;
- Facilitating and supporting capacity building in public procurement;
- Organising and participating in administrative review of complaints and appeals on public procurement;
- Maintaining registers of procurement entities, members and secretaries of tender committees, and of suppliers, contractors, consultants, and records of prices;
- Investigating and debarring from public procurement suppliers, contractors and consultants who have contravened the provisions of the Act and Regulations, and communicating a list of debarred firms to Procurement Entities;
- Through an annual procurement forum, providing a platform for discussion of issues relating to public procurement; and
- Assisting the local business community to become competitive and efficient suppliers to the public sector.

2.2 PROCUREMENT ENTITY

A Procurement Entity is an organization or person that has legal/administrative mandate for procurement purposes.

**CHARACTERISTICS**	EXPLANATION
Uniquely identifiable.	No two entities shall be the same.
No overlap of operational area	The emphasis is on the word “operational” as it defines what the entity is supposed to

	be doing. Eg a Regional Health Directorate cannot belong to both the Regional Coordinating Council and the Ministry of Health. The operational word is "health" and therefore belongs to the Ministry of Health. The word "regional" is only an attribute that distinguishes it from other health directorates.
Has an account holder as head	Has a head of department who is authorized to incur expenses on behalf of the department/organization/institution.
Has a budget	Prepares and submits a budget as stipulated by statute or as required by policy.
Can undertake procurement	Can undertake procurement activities.
Can form contracts	Has the legal/administrative authority to enter into contracts

TYPE OF PROCUREMENT ENTITY	EXAMPLES
1. Central Management agencies	1a. Public Service Commission<br>1b. Office of the President<br>1c. Office of head of Civil Service
2. Ministries Departments and Agencies	2a. Ministry of Health<br>2b. Department of Feeder Roads<br>2c. Ghana Health Service
3. Sub vented Agencies	3a. Ghana Library Board<br>3b. Ghana News Agency<br>3c. Ghana Statistical Service
4. SOEs	4a. Volta River Authority<br>4b. State Insurance Company<br>4c. Electricity Company of Ghana

2.3 THE HEAD OF PROCUREMENT ENTITY

The Head of Procurement Entity and any officer to whom authority is delegated are responsible and accountable for actions taken and for any instructions with regard to the implementation of the Act. He/she is responsible to ensure that provisions of the Act are complied with; and concurrent approval by any Tender Review Board will not absolve him/her from accountability for a contract that may be determined to have been procured in a manner that is inconsistent with the provisions of the Act.

The Head of Entity is required to establish a Procurement Unit to undertake all activities related to procurement within the Entity in accordance with the Act and shall appoint or designate a proficient procurement person with the requisite qualifications, experience and skills as Head of the Procurement Unit to undertake the detailed activities of procurement on behalf of the Procurement Entity.

The Head of Procurement Entity's role in procurement processes shall include:

- ❑ review and approval of recommendations for contract awards submitted by the Procurement Unit;
- ❑ participating as the Chairman or a Member in decisions of a Tender Committee [or Tender Review Board as the case may be];
- ❑ review of decisions on complaints submitted under Section 79 of the Act; and
- ❑ signing contracts on behalf of the Procurement Entity.

The Head of Entity may formally delegate his/her authority to an officer to undertake any of his powers or responsibilities for procurement under the Act or the Regulations.

2.3.1 Approval of Contract Awards by the Head of Entity

Schedule 1 of the Act provides threshold values up to which the Head of Entity may approve the award of contracts. In considering submissions made by the Procurement Unit, the Head of Entity may:

- ❑ approve a submission;
- ❑ reject a submission with reasons; or
- ❑ approve a submission, subject to clarifications or amendments.

The Head of Entity shall not:

- ❑ modify a submission; or
- ❑ reject any submission without good reasons in writing.

Where the Head of Entity has a conflict of interest in any submission, he shall declare his interest in the submission and refer it to the officer to whom authority has been delegated for a decision.

For Contracts above the Head of Entity's threshold as stipulated in Schedule 3, the Head of Entity shall, in consultation with the Procurement Unit, review all documentation and decisions related to the contract and submit the recommendations for approval by the relevant approving authority.

2.4 PROCUREMENT UNIT

A Procurement Unit is an outfit in a Procurement Entity with the responsibility of superintending procurement. The Head of the Procurement Unit (and his/her team) shall be responsible for undertaking and co-ordinating all detailed procurement activities within the procurement entity. This shall include:

- receiving procurement requests from originating officers, checking that the proposed procurement is within the approved procurement plan, and that budgeted funds are available prior to commencement of procurement proceedings;
- ensuring that funds are properly committed prior to issue of any contract or Purchase Order;
- co-ordinating the preparation of specifications, terms of reference, bills of quantities, drawings, short-lists or advertisements, and prequalification, tender or request for quotation documents. Where so required by the Tender Committee, submission of documentation for review and approval by the Tender Committee prior to issue;
- arranging the publication of advertisements and notices of contract award;
- co-ordinating the process of opening of tenders and quotations and ensuring the preparation of formal records of tender or quotation opening in accordance with Section 56 of the Act.
- participating in evaluation activities of the Tender Evaluation Panel where necessary and assisting in preparation of formal Evaluation Reports;
- preparing submissions for approval of award by the appropriate authority in accordance with the threshold values established in Schedule 3 of the Act.
- maintaining and updating the database of suppliers, contractors and consultants;
- participating in negotiations with consultants where necessary or other tenderers where expressly permitted in the Act or Regulations;
- preparing notification of awards and contracts;
- arranging publication of notices of contract awards;
- preparing contract documents and Purchase Orders, in line with the award decision;
- preparing and issuing tender rejection and tenderer debriefing letters;
- preparing contract variations and modifications;
- assisting with the inspection and acceptance of goods, works and services;
- maintaining procurement records in accordance with Section 28 of the Act and the Regulations.

2.5 ENTITY TENDER COMMITTEE

An Entity Tender Committee is required to be established in each Procurement Entity with a structure as defined in Schedule 1 of the Act .

In accordance with Schedule 1 of the Act as well as the Regulations, an Entity Tender Committee is responsible for;

- review and approval of annual Procurement Plans in accordance with Schedule 1 of the Act, and for review and approval of quarterly updates of procurement plans.
- In considering submissions made by the Procurement Unit or the Tender Evaluation Panel, the Tender Committee may:
  - a) approve a submission;
  - b) reject a submission with reasons; or
  - c) approve a submission, subject to clarifications or minor amendments.

The Entity Tender Committee shall not:

- a) modify a submission, including, in particular recommendations for contract award; or
- b) reject any submission without good and justifiable reason.

Award of contract shall be made solely on the basis of information and evaluation criteria provided in the tender documents or request for proposals, and without recourse to any extrinsic evidence, or influenced by personal or political preferences.

All approvals for award of contract must be within the annual procurement plan for the financial year and the Entity Tender Committee must confirm that sufficient funding is available within the remaining vote code balance.

Where any member of the Entity Tender Committee has a conflict of interest in any submission, he shall declare his interest in the submission, leave the meeting while the matter is considered and shall not participate in the deliberations or decision-making process of the Committee in relation to that submission.

Decisions of the Entity Tender Committee should be unanimous. Where unanimity cannot be achieved after extensive discussions, a decision shall be by simple majority vote with the Chairperson of the Entity Tender Committee having a casting vote in the event of a tie. The result of the voting shall be recorded in the minutes of the Entity Tender Committee together with an explanation of the failure to reach a unanimous decision.

The Entity Tender Committee shall provide explanation and justification of its reasons for any rejection to the Head of Entity for re-submission.

All decisions of an Entity Tender Committee, including reasons for any rejections, shall be recorded in minutes and notified to the Procurement Unit and the Tender Evaluation Panel in writing.

If a procurement exceeds (or is estimated to exceed) the financial authority of the Entity Tender Committee, it must be referred immediately with the Committee’s recommendations to the appropriate Tender Review Board for approval.

The Secretary to the Entity Tender Committee shall record minutes of all Tender Committee meetings, which shall include:

- a register of attendance;
- a list of all submissions considered;
- the decision made for each submission, including any major issues discussed, the reasons for any rejections and any clarifications or minor amendments to which the approval is subject;
- any conflicts of interest declared by members; and
- any dissenting opinions among Tender Committee members.

2.6 TENDER EVALUATION PANELS

Each Procurement Entity is required to appoint a Tender Evaluation Panel with the required expertise to evaluate tenders and assist the Tender Committee in its work.

2.6.1 Composition of Tender Evaluation Panels

A Tender Evaluation Panel shall be an ad hoc body of not more than five members constituted for a specific procurement package. The panel shall include members with skills, knowledge and experience relevant to procurement requirements, which may include:

- relevant technical skills;
- end user representation;
- procurement and contracting skills;
- financial management or analysis skills; or
- legal expertise.

Members appointed to the Panel may be staff of the Procurement Unit but no Member of the Entity Tender Committee shall act as a member of the Tender Evaluation Panel, except in an advisory capacity.

To ensure transparency, members of the Tender Evaluation Panel shall not be directly involved in the approval of any award of contract.

2.6.2 Proceedings of Tender Evaluation Panels

Recommendations for award of contract shall be made solely on the basis of information and evaluation criteria provided in the tender documents or request for proposals and without recourse to any extrinsic evidence, or influenced by personal or political preferences.

Where any member of the Tender Evaluation Panel has a conflict of interest in any tender evaluation, he/she shall declare his interest in the tender, leave the meeting while the matter is considered and shall not participate in the deliberations or decision-making process of the Panel in relation to that submission.

A formal evaluation report shall be prepared for each evaluation conducted and signed by all participating Members of the Panel.

Scoring of tenders shall be conducted individually by Members of the Tender Evaluation Panel and the scores formally combined to arrive at a recommendation for contract award. Recommendations of the Tender Evaluation Panel shall be unanimous, but any dissenting views shall be recorded within the evaluation report to be submitted to the Tender Committee.

The Secretary to the Tender Evaluation Panel shall record minutes of all Panel meetings, which shall include:

- a register of attendance;
- a list of all submissions considered and the recommendations made;
- any conflicts of interest declared by members; and
- any dissenting opinions among Tender Evaluation Panel members.

2.7 TENDER REVIEW BOARDS

A Tender Review Board is a body established in accordance with Schedule 2 of the Act to review procurement decisions within the thresholds set out in Schedule 3 of the Act. The Tender Review Board is to review the activities at each step of the procurement cycle which will lead to the selection of the lowest evaluated tender by the Procurement Entity.

Tender Review Boards are established at the following levels:

- Central Tender Review Board;
- Ministerial Tender Review Boards;
- Regional Tender Review Boards;
- District Tender Review Boards.

In considering submissions made by the Procurement Entity, the Tender Review Board may:

- approve a submission;
- reject a submission with reasons; or
- approve a submission, subject to clarifications or minor amendments.

The Tender Review Board cannot:

- modify a submission, including, in particular recommendations for contract award; or
- reject any submission without good and justifiable reason.

Award of contract shall be made solely on the basis of information and evaluation criteria provided in the tender documents or request for proposals and without recourse to any extrinsic evidence, or influenced by personal or political preferences.

All approvals for award of contract must have been captured in the annual procurement plan for the financial year and the Tender Review Board must confirm that sufficient funding is available within the remaining vote code balance.

Where any member of the Tender Review Board has a conflict of interest in any submission, he shall declare his interest in the submission, leave the meeting while the matter is considered and shall not participate in the deliberations or decision-making process of the Board in relation to that submission.

Decisions of the Tender Review Board should be unanimous. Where unanimity cannot be achieved after extensive discussions, a decision shall be by simple majority vote with the Chairperson of the Board having a casting vote in the event of a tie. The result of the voting shall be recorded in the minutes of the Tender Review Board together with an explanation of the failure to reach a unanimous decision.

The Tender Review Board shall provide explanation and justification of its reasons for any rejection to assist the Procurement Entity in remedying any defects in the submission prior to re-submission to the Tender Review Board.

All decisions of a Tender Review Board, including reasons for any rejections, shall be recorded in minutes and notified to the Procurement Entity in writing.

The secretary of the Tender Review Board shall record minutes of all Tender Review Board meetings, which shall include:

- ❑ a register of attendance;
- ❑ a list of all submissions considered;
- ❑ the decision made for each submission, including any major issues discussed, the reasons for any rejections and any clarifications or minor amendments to which the approval is subject;
- ❑ any conflicts of interest declared by members; and
- ❑ any dissenting opinions among Tender Review Board members.

2.8 ORIGINATING DEPARTMENTS, PROJECTS OR UNITS

The Originating Officer, Department, Project, Programme or Unit is the initiator of requests for procurement or asset disposal action and has responsibility to:

- ❑ Ensure that the requirement for procurement is:
  - o justified by the needs of the Department, Project, Programme or Unit;
  - o not for goods already available in the Stores;
  - o within the annual procurement plan; and
  - o covered by available funding in the annual budget or development plan;
- ❑ Prepare the initial specification of the requirement; and
- ❑ Prepare a Procurement Requisition.

2.9 STORES DEPARTMENTS

Stores Departments are responsible for the following procurement activities:

- ❑ preparation of the annual budget for both allocated and unallocated stores.

- receipt of goods; and
- originating department for procurement of stock items.

2.10 INSPECTION/TAKEOVER TEAMS

The receipt of all goods, and the validation of work completed for works and services is subject to inspection and verification an Inspection Team.

CHAPTER 3:PROCUREMENT PLANNING AND BUDGETING

3.1 PROCUREMENT PLANNING

Section 21 of the Act requires Procurement Entities to prepare a procurement plan for each fiscal year, and prepare quarterly updates for approval by the Tender Committee.

The Regulations require that the procurement planning process is fully integrated with applicable budget processes and circulars issued by the Public Procurement Board and the budget preparation instructions of the Ministry of Finance.

Adequate procurement planning and prioritisation of needs by each Procurement Entity is an essential prerequisite to effective purchasing for the following reasons:

- ❑ Funding for procurement is unlikely to be sufficient to meet all requirements, and scarce financial resources must be channelled to ensure that the priority aims of a Procurement Entity are adequately met before spending on less essential procurements.
- ❑ Effective planning allows requirements to be aggregated into larger purchases at lower unit costs, rather than frequent sourcing of quotations for identical items and issuing many individual Local Purchase Orders.
- ❑ Procurement of Common User items may also be aggregated for more than one Procurement Entity into Framework (Call-off) Contracts for six months or a year, to permit further economies of bulk purchasing, saving of time wasted by separate procurements, and a reduction of the need to maintain high stock levels.
- ❑ Publication of realistic annual procurement plans allows the private sector to respond more effectively to the requirements and specifications of Government, through investment in staff and equipment, manufacture and importing of goods, and financial planning.

Structured development of procurement plans is an essential part of the annual budget preparation process and provides a ready checklist for the approval of procurements by Tender Committees and Tender Review Boards, and monitoring of procurement activity by the Public Procurement Board.

3.2 THE BUDGET PREPARATION PROCESS

Annual budgets are prepared each year in accordance with the procedures specified by the Ministry of Finance. Detailed instructions for the preparation of Annual Procurement Plans will be issued by the Public Procurement Board in consultation with the Ministry of Finance.

The Head of Procurement Entity has responsibility for co-ordinating the preparation of the budget for the Procurement Entity, including procurement plans based on previous consumption and estimated requirements for stores replenishment, and the submissions of Departments, Units and Projects. The work of compilation, costing and prioritisation of expenditure for the

Procurement Plan will be undertaken with the assistance of the Procurement Unit.

Preparation of the Annual Procurement Plan should be commenced at least four months before the start of the Financial Year to allow sufficient time for a realistic and accurately costed plan to be compiled.

3.2.1 Contents of the Procurement Plan

The procurement plan for each Procurement Entity shall include:

- a detailed breakdown of the goods, works and services required;
- a schedule of the delivery, implementation or completion dates for all goods, works and services required;
- the source of funding;
- an indication of any items that can be aggregated for procurement as a single package, or for procurement through any applicable arrangements for common use items;
- an estimate of the value of each package of goods, works and services required and the source of funding; and
- details of any committed or planned procurement expenditure under existing multi-year contracts.

In determining the optimum packaging of planned contracts a Procurement Entity shall aggregate procurement requirements, where appropriate, to achieve economies of scale. In deciding where aggregation is appropriate, the Procurement Entity shall consider all relevant factors, including:

- which procurements are of a similar nature and likely to attract the same potential tenderers;
- shelf-life and storage constraints;
- when delivery, implementation or completion is required;
- the optimum size and type of contract to attract the greatest and most responsive competition, taking into account the market structure for the requirement;
- which procurements will be subject to the same tendering requirements and conditions of contract; and
- the potential to realise savings in time or transaction costs or to facilitate contract administration by the Procurement Entity.

3.2.2 Head of Procurement Entity Actions

- Instruct the Procurement Unit to commence development of the Annual Procurement Plan in accordance with the instructions issued by the Public Procurement Board and the Ministry of Finance.
- Ensure that the draft annual procurement plan is reviewed and approved by the Tender Committee not less than one month before the commencement of the new financial year.
- Include the approved annual procurement plan and costings in the Annual Budget submission to the Ministry of Finance.

- If insufficient budget funding is available following notification of the approved Annual Budget, instruct the Procurement Unit to review the Procurement Plan according to service priorities.
- Obtain approval of the final Annual Procurement Plan from the Tender Committee and forward a copy to the Public Procurement Board.
- Ensure that quarterly updates of the procurement plan are prepared for approval by the Tender Committee, and forward a copy of the approved Plan to the Public Procurement Board.

3.2.3 Procurement Unit Actions

- Subject to the instructions of the Head of the Procurement Entity, the Public Procurement Board and the Ministry of Finance, commence the preparation process for the Annual Procurement Plan at least four months before the start of the next Financial Year.
- Issue detailed instructions to the Heads of departments, units, projects and programmes on the format and content of submissions for the Procurement Plan. The instructions should clearly indicate that items omitted from the submissions may not be purchased in the Financial Year without adjustment to the quantities of other items under the same detailed Vote Sub-Head, or the formal issue of a supplementary budget. Note that the Public Procurement Board will provide Procurement planning software and planning templates for use by Procurement Entities, together with detailed instructions and timetables for compilation of the Procurement Plan.
- Receive submissions, check quantities and costing, obtain clarifications and other responses to any queries, analyse and compile all submissions into a procurement plan for the Procurement Entity.
- Submit the draft Annual Procurement Plan through the Head of Procurement Entity to the Tender Committee for review and approval.
- Following approval of the Annual Budget, and in full consultation with Heads of departments, units, projects and programmes, review the Procurement Plan according to service priorities.
- Compile the final Annual Procurement Plan and submit through the Head of Procurement Entity to the Tender Committee for approval.
- Notify individual Heads of departments, units, projects and programmes of their approved procurement plans and annual procurement budgets.

3.2.4 Stores Departments Actions

- Analyse the stores ledger and extract stock descriptions, annual issues and seasonal usage patterns, current stock levels, average purchase quantities, anticipated requirements for the next Financial Year and costings.
- Submit detailed schedules of the annual requirement for stock items, the costs analysed between detailed Vote Codes, and supporting information to the Procurement Unit in accordance with the instructions received.
- Respond to any queries raised by the Procurement Unit.

- Receive notification of the final approved Annual Procurement Plan and budget and use this as an essential guideline for all procurement processes to be undertaken in the new Financial Year.

3.2.5 Heads of Department, Units, Projects and Programmes

Heads of departments, units, projects and programmes are required to ensure the analysis and preparation of Annual Procurement Plans for their own and subordinate areas of control.

Procurements for development partner-funded projects and programmes should also be included but identified in a separate section of the Procurement Plan.

Principal stages are as follows:

- Review previous procurement plans to determine if any planned procurements will need to be carried forward into the new financial year.
- Identify specific procurement requirements and categorise them into the types of procurement, i.e., Goods, Works or Services.
- Aggregate similar items into suitable packages or lots and estimate the cost of each package or lot.
- The procurement thresholds in Schedule 3 of the Act determine the procurement method to be used. It should be noted that the procurement requirement may not be spilt into parts to avoid the use of a specified procurement method.
- Determine the appropriate body responsible for approval, i.e. Head of Entity, Entity Tender Committee or Tender Review Board.
- Complete the procurement template with realistic Lead Time deadlines, taking cognisance of
  - Administrative activities
  - Manufacture, Construction or Service periods
  - Delivery
  - Contract completion
- Note that the dates to appear on the plan are ‘Completion Dates’ i.e. the projected date by which the whole task would have been accomplished
- Respond to any queries raised by the Procurement Unit.
- Receive notification of the final approved Annual Procurement Plan and budget and use this as an essential guideline for all procurement processes undertaken in the new Financial Year.

3.3 GUIDELINES FOR DETERMINING COMPLETION TIMESCALES

3.3.1 Estimated lead times for ICT (Goods)

1.	Preparation of Tender Documents	-	1-6 weeks
2.	Prior review/ETC/TRB approval	-	1-2 weeks
3.	Adverts/Tender Invitation	-	6weeks minimum
4.	Tender Close/ Opening	-	same date
5.	Tender Evaluation and Report Submission	-	2-4 weeks
6.	Approval by ETC/TRB	-	1-2 weeks
7.	Contract Award	-	0-2 weeks
8.	Contract Signature	-	1-4 weeks
9.	Letters of Credit (Goods)	-	2-4 weeks
10.	Delivery	-	6-16 weeks
11.	Inspection and Acceptance	-	0-4 weeks

If there is the need for Prequalification of Suppliers or Contractors then total time for the delivery of the goods or works will have to be expanded by 7-13 weeks to allow for the completion of the prequalification procedures.

3.3.2 Estimated lead times for NCT (Goods)

1.	Preparation of Tender Document	-	1-3 weeks
2.	Prior Review/ETC/ TRB approval	-	1-2 weeks
3.	Advert/Tender Invitation	-	2-4 weeks
4.	Tender Close/Opening	-	same date
6.	Tender Evaluation and Report Submission	-	1-2 weeks
7.	Post Review/No objection	-	1-2 weeks
8.	Contract Award	-	0-2 weeks
9.	Contract Signature	-	1-3 weeks
10.	Letter of Credit	-	1-4 weeks
11.	Delivery	-	As per contract
12.	Inspection and Acceptance	-	0-1 weeks

3.3.3 Estimated lead times for ICT (Works)

1.	Preparation of Tender Documents	-	2weeks minimum-4 weeks
2.	Prior Review/ETC/TRB approval	-	1-2 weeks
3.	Advert/Tender Invitation	-	6-8 weeks
4.	Tender Close/Opening	-	same date
5.	Tender Evaluation and Report Submission	-	2-4 weeks
6.	Post Review / ETC/TRB Approval	-	1-2 weeks
7.	Contract Award	-	1-2 Weeks
8.	Contract Signature	-	2-3 Weeks
9.	Mobilisation (Advance Payment)	-	2-4 weeks
10.	Completion Period		As per contract
11.	Final Acceptance	-	24-52 weeks

3.3.4 Estimated lead times for NCT (Works)

1.	Preparation of Tender Documents	-	2weeks minimum
2.	Prior Review/ETC/TRB	-	1-2 weeks
3.	Advertising/Tender Invitation	-	2-4 weeks

4.	Tender Close/Opening	-	same date
5.	Tender Evaluation and Report Submission	-	2-4 weeks
6.	Post Review /ETC/TRB approval	-	1-2 weeks
7.	Contract Award	-	1-2 Weeks
8.	Contract Signature	-	1-3 Weeks
9.	Mobilisation (Advance Payment)	-	2-4 weeks
10.	Completion Period	-	As per contract
11.	Final Acceptance	-	24 weeks

3.3.5 Estimated lead times - Request for Quotation

1.	Preparation of documents	-	0-1 weeks
2.	Invitation or Solicitation letter	-	0-2 weeks
3.	Tender Close/Opening	-	same date
4.	Evaluation and Submission of Report	-	0-1 week
5.	Award of Contract	-	0-1 week
6.	Contract Signature	-	1 week
7.	Delivery/Completion period	-	1-4 weeks
8.	Inspection and Acceptance	-	0-1 weeks
9.	Final Acceptance (works)		As per Contract

3.4 PLAN MONITORING AND UPDATING

During project execution the original procurement plan should be regularly monitored and updated. The essence is to see how actual performance compares with the planned activities and to make changes in the plan if necessary.

If slippage occurs in the award or execution of one major contract, it may require rescheduling of other related contract awards and deliveries.

The purpose of monitoring is to complete the details of what has actually been executed, to note whether there are major discrepancies with what was anticipated, and make adjustments in the plans so as to give a complete picture of procurement performance.

A full revision and update of the Procurement Plan must be submitted to the Tender Committee for review and approval on a Quarterly basis throughout each Financial Year.

CHAPTER 4: PROCUREMENT OF GOODS

4.1 DEFINITION OF GOODS

Goods means objects of every kind and description including raw materials, products and equipment and objects in solid, liquid or gaseous form, and electricity, as well as services incidental to the supply of the goods if the value of those incidental services does not exceed that of the goods themselves.

4.2 PROCUREMENT METHODS

The procurement methods that can be used for the procurement of Goods are:

- International Competitive Tendering (ICT)
- National Competitive Tendering (NCT)
- Two-Stage Tendering (National or International)
- Restricted Tendering (National or International)
- Single Source (Direct Procurement)
- Request for Quotations (RFQ)

Competitive tendering using ICT or NCT is the preferred method for Government procurement and the use of alternative methods is strictly limited to the provisions of Part IV of the Public Procurement Act.

4.2.1 International Competitive Tendering

International Competitive Tendering is appropriate for high value or complex procurements, or where the supply of goods by their nature or scope, is unlikely to attract adequate local competition. The Act requires the use of ICT for procurement of goods above the threshold stated in Schedule 3.

4.2.2 National Competitive Tendering

National Competitive Tendering is appropriate for lower value procurements, where the goods by their nature or scope are unlikely to attract foreign competition, or where there are justifiable reasons for the Procurement Entity to restrict tendering to domestic suppliers. The Act permits the use of NCT for procurement of goods valued at the thresholds stated in Schedule 3.

4.2.3 Restricted Tendering

Restricted Tendering is a tendering process by direct invitation to a shortlist of pre-qualified, pre-registered or known suppliers, and is subject to a specific approval being granted by the Public Procurement Board:

It is an appropriate method of procurement where:

- the requirement is of a specialised nature or has requirements of public safety, or public security which make an open competitive tender inappropriate;
- due to the urgent nature of the requirement, an open competitive tender is not practical;
- the number of potential suppliers is limited; or

- an open competitive tender has failed to bring an award of contract.

4.2.4 Two-Stage Tendering

Two-stage Tendering is an infrequently used procurement process in which a Procurement Entity invites tenderers in the initial stage to contribute to the detailed specification of the goods. Following review and consultations, new detailed specifications are prepared and a restricted tender issued in the second stage to all participants who were not rejected in the first-stage.

It is an appropriate method of procurement when it is not feasible for the Procurement Entity to formulate detailed specifications for the goods, to identify their characteristics, or the character of the goods is subject to rapid technological advances.

4.2.5 Single Source

Single source procurement from a supplier without competition (direct procurement) is subject to a specific approval being granted by the Public Procurement Board

Single source procurement may be appropriate when:

- the purchase is for urgently needed products, provided this is restricted to the minimum quantity to meet the urgent need until a purchase by other methods can be fulfilled; or
- the requirement can only be supplied by one source for physical, technical or policy reasons, e.g. the required equipment is proprietary and obtainable only from one source.
- when national security (non economical) considerations are paramount.

4.2.6 Request for Quotations (RFQ)

This is also known as “shopping” and is based on comparing price quotations obtained from several suppliers, usually at least three, to ensure competitive prices.

Request for Quotations may be used when:

- the estimated value of the threshold specified in Schedule 3 of the Act;

Standard RFQ documents are particularly suitable for procuring readily available off-the-shelf goods or standard specification items of low value.

4.3 SPECIFICATION OF REQUIREMENT

The description of goods to be procured shall contain or be accompanied by a complete, precise and unambiguous description of the goods required. The description may include drawings where appropriate.

Specifications shall include:

- the purpose and objectives of the goods;
- a full description of the requirement;
- a functional description of the goods, including any environmental or safety features;

- performance parameters, including outputs and any indicators or criteria by which satisfactory performance can be judged;
- process and materials descriptions;
- dimensions, symbols, terminology language, packaging, marking and labelling requirements;
- inspection and testing requirements; and
- any applicable national or international standards required.

5.3.1 Use of Brand Names

Descriptions of goods shall not include any reference to a particular trademark, brand name, patent, design, type, specific origin, producer, manufacturer, catalogue or numbered item, unless there is no other sufficiently precise or intelligible way of describing the requirement, in which case the words “or equivalent” shall be included and the description shall serve only as a benchmark during the evaluation process.

Notwithstanding the above, where a particular trademark, brand name, patent, design, type, specific origin, producer, manufacturer, catalogue or numbered item is essential for reasons of technical compatibility, servicing, maintenance or preservation of warranty conditions, such description may be used, subject to inclusion of the words “or equivalent”. The requirement needs to be fully identified with detailed technical specifications.

If detailed specifications cannot be prepared by the originating Department or Unit, the advice of the Procurement Unit may be sought for assistance or the appointment of a specialist technical advisor.

4.4 PRE-QUALIFICATION OF TENDERERS FOR GOODS

Pre-qualification of tenderers:

- is mandatory for procurement of goods with an estimated value at the threshold value stated in Schedule 3 of the Act.
- is appropriate in circumstances when the high costs of preparing detailed tenders could discourage competition;
- is often used for custom-designed equipment and industrial plant under turnkey contracts;
- ensures that invitations to tender are extended only to those who have adequate capabilities and resources;
- helps to ensure that only tenders from competent suppliers are considered and eliminates disputes that may arise from rejection of lowest tenderers because the tenderers are not considered qualified;
- may be a requirement on a development partner funded procurements.

4.4.1 Pre-qualification Criteria

Pre-qualification is based upon the capability and resources of prospective tenderers to perform the particular contract satisfactorily, taking into account their:

- experience and past performance on similar contracts;
- capabilities with respect to personnel, equipment, and construction or manufacturing facilities;
- required licensing and professional registrations; and
- commercial and financial resources.

4.4.2 Advertisement for pre-qualification

The invitation to pre-qualify for tendering on specific contracts or groups of similar contracts shall be advertised in a similar way to a competitive tender. The scope of the contract and a detailed statement of the requirements for qualification must be provided in the advertisement.

4.4.3 Pre-qualification evaluation

No formal opening of submissions is required but a formal evaluation of the capabilities of each supplier is required by the Tender Evaluation Panel against the criteria as stated in the advertisement.

- Where a large number of suppliers pass the stated qualification criteria, it may not be realistic to invite more than eight to participate in the specific tender. If so, only the best qualified suppliers should be selected and invited to tender.
- All applicants should be informed of the results of pre-qualification. Applicants who fail to meet the pre-qualification criteria should be briefly advised of the reasons. No further correspondence will be entered into regarding applications.
- When pre-qualification is completed, Tender Document should be issued to all qualified tenderers, or to an appropriate shortlist of the qualified tenderers.

4.5 TENDERING PROCEDURES

4.5.1 Standard Tender Document

Schedule 4 of the Act specifies the various Standard Tender Documents. A Standard Tender Document is the set of document issued by the PPB which establishes the goods required (technical specifications), the procurement procedures to be followed, and specifies the proposed contract conditions. It should contain all information necessary to allow the tenderer to prepare a responsive tender or quotation.

The Tender Document must contain information to:

- instruct tenderers on the procedure for submission of tenders;
- describe the goods to be procured;
- inform tenderers of the criteria for evaluation of tenders; and
- define the conditions of any resulting contract.

Use of a Standard Tender Document (STD) format, as defined in Schedule 4 of the Act, is mandatory for all procurement of goods, unless an alternative format has been specifically approved by the Public Procurement Board. Note

that procurement of goods using development partners funds may be subject to the required use of the development partner's standard formats if so specified in the loan or credit agreement, in accordance with Section 96 of the Act.

The updated versions of each approved STD, together with an individual User Guide to assist in preparation of the TD, may be obtained from the Public Procurement Board, or directly downloaded from the PPB website. (www.ppbghana.org)

4.5.2 Preparation of Tender Document

The preparation of tender documents which is a team work, shall be coordinated by the Head of Procurement Unit. Upon receipt of requisition to procure and notification of availability of funds, the Head of Procurement proceed with the preparation of the document.

The tender document must be prepared by modifying the appropriate Standard Tender Document (STD) issued by the PPB. The STDs as listed in schedule 4 of Act 663, may be obtained directly from the PPB or downloading it from the PPB website. (www.ppbghana.org).

The Instructions to Tenderers and the General Conditions of Contracts should not be changed. However, modifications can only be made through the Tender Data sheet and Special Condition of Contract respectively.

The key sections that need completion and modifications are;

a) The Invitation to Tender,

b) The Tender Data Sheet.

c) Technical Specification

d) The Schedule of Requirements.

e) The Special Conditions of Contract) Sample Forms (to be completed by the Supplier)

Actions

The Head of Procurement Unit should:

- ❑ Select the appropriate standard tender document depending on the type of procurement.
- ❑ Complete the Invitation to Tender section with the relevant information which is self explanatory.
- ❑ Consult with the end-user, regarding finalisation of the description of requirements (Specification) for inclusion in the document.
- ❑ Complete the Tender Data Sheet in section II with details of the tendering process e.g. the tender closing date, the address for submission, the evaluation criteria to be applied, any qualification criteria to be applied etc. The text of the Instructions to Tenderers should not be modified.
- ❑ Complete the Special Conditions of Contract in section VIII with the conditions which will apply to the contract e.g. payment terms, delivery/completion period, warranty, liquidated damages. Note that the

Special Conditions of Contract modify the General Conditions of Contract – the text of the General Conditions of Contract must not be modified.

- ❑ Complete the Schedule of Requirement section by describing the required items, quantities etc.

The Sample Forms provide text and format samples for the various forms which are to be completed by the tenderer.

4.5.3 Use of Standard Request for Quotation

The SRFQ shall be used for the procurement of goods for smaller values as stated in the schedules. A Standard Request for Quotations is required for the Request for Quotations method only.

An entity's RFQ document shall contain the following

- o the precise description of the goods required;
- o the rules for the procurement process;
- o the evaluation criteria which will be applied;
- o any qualification criteria which will be applied; and
- o the type and conditions of the proposed contract (purchase order).

Action:

The Head of Procurement Unit should:

- ❑ Consult with the end-user, and technical specialists if required, regarding finalisation of the description for inclusion in the document
- ❑ Complete details of the procurement process e.g. the tender closing date, the address for submission, the evaluation criteria to be applied, any qualification criteria to be applied etc.
- ❑ Complete the Conditions of Contract section with the conditions which will apply to the contract (purchase order) e.g. payment terms, delivery/completion period, warranty, liquidated damages.

4.5.4 Invitation Advertisements for Competitive Tenders (ICT and NCT)

Publication of the advertisement must allow sufficient time before the deadline (or closing day and time of the tender) to enable prospective tenderers to obtain the tender documents and to prepare and submit their tenders. The period allowed should be a minimum of 6 weeks for ICT, and 2 weeks for NCT following the date of the first advertisement, but longer periods may be necessary for more complex requirements.

Procurement Entities must be careful to also allow for the time between submission of the advertisement to the press and the time the advertisements are actually published.

Advertisements for NCT and ICT shall be published:

- in the Public Procurement Bulletin and the PPB website;(www.ppbghana.org) and
- in at least two local daily newspaper of general circulation in Ghana,

In the case of International Competitive Tendering, publication in international newspapers or journals may be used. However, copies of the Invitation to Tender submitted to all foreign Embassies and High Commissions in Ghana shall be deemed to have met this requirement.

4.5.5 Contents of the Advertisement

The information contained in the advertisement must correspond to the information in the Tender Document and in particular, the relevant information in the Tender Data Sheet. The advertisement should include as minimum, brief information on:

- the source of funding;
- the goods to be procured;
- any qualification requirements for tenderers (e.g. a requirement for a minimum level of experience in the subject of the procurement);
- the procedure for obtaining the Tender Document;
- the place and deadline for submission of tenders;
- the fee to be paid for the Tender Documents. This must reflect only the cost of printing and delivery to prospective Tenderers;
- the value of any Tender Security required.

4.6 SALE OF TENDER DOCUMENTS FOR COMPETITIVE TENDERS

The sale of tender documents for competitive tenders will be co-ordinated by the Procurement Unit or the Technical Department as appropriate. The price charged for the documents must reflect only the cost of printing and provision of the tender documents.

The Procurement Entity must ensure that tender documents are available for sale before publication of the invitation. If the stock of tender documents is exhausted, immediate action to print additional copies shall be initiated. It is an offence to deny a prospective tenderer the opportunity to purchase a tender document, and failure to provide a tender document for inspection or purchase by a potential tenderer may constitute grounds for complaint under Part VII of the Act.

Action:

- Prospective tenderers should be allowed to briefly inspect the Tender Document, if requested, before committing to purchase of the document.
- Record the name and address of each purchaser of the Tender Document in a register.
- Fees received from the sale of Tender Documents must be recorded and the income deposited in the Consolidated Fund. A formal Receipt must be issued to the purchaser.

- ❑ If the Tender is subject to conformity with a sample or samples, obtain written acknowledgement from the Tenderer that the sample or samples have been inspected.

4.7 ISSUE OF INVITATIONS FOR RESTRICTED TENDER

The Tender Document for a Restricted Tender must be issued to all short-listed tenderers simultaneously.

Action:

The Head of Procurement Unit should:

- ❑ Prepare the Tender Document together with a personalised Letter of Invitation for each short-listed tenderer.
- ❑ Send the invitation by registered post to all short-listed tenderers, or deliver by hand and obtain a written receipt.
- ❑ If the tender is subject to conformity with a sample or samples, obtain written acknowledgement from the tenderer that the sample or samples have been inspected.

4.8 ISSUE OF REQUESTS FOR QUOTATIONS

The procedure for issue of Requests for Quotations to short-listed tenderers follows the same principles as for Restricted Tender above.

The Standard Tender document formats for Request for Quotations will be used for simple requirements wherever practical. For more complex requirements when the normal SRFQ and Purchase Order documents are not appropriate nor provide an appropriate form of contract, the Procurement Entity may utilise appropriate formats from the other Standard Tender Documents.

4.8.1 Opening of Standard Requests for Quotations

Public opening of the quotations submitted is required. All quotations must be opened on a predetermined date and time by the Procurement Unit to avoid the opportunity for any supplier to become aware of the prices quoted by other suppliers.

The recording of quotations and the evaluation procedures followed are similar to those for tenders except for the following:

- ❑ Quotations will normally be submitted using the appropriate Standard Request for Quotations document;
- ❑ Sealed quotations and any samples must be placed in the tender box;
- ❑ The Procurement Unit will register the receipt of each quotation and keep the envelopes secure and unopened until the date of the RFQ opening;

Action:

The Head of procurement Unit Should:

- ❑ Ensure that the name, organisation represented and contact details of all attendees are recorded in an attendance register.

- ❑ Ensure the security of documents at all times during the procedure to prevent any unauthorised interference with the documents.
- ❑ Bring in the unopened quotations and any samples received and check that the writing on each envelope or sample confirms that it is for the correct RFQ.
- ❑ Open the first quotation after confirming that any conditions regarding marking and sealing of the envelope have been met and the envelope has not been tampered with. Note each quotation with a serial number in sequence as it is opened (e.g. "1 of 4" where the total number of RFQs is 4).
- ❑ Examine the contents of the envelope and identify, stamp and number each RFQ and any separate sections and attachments.
- ❑ Read out the following details from each quotation:
  - o the number allocated to the quotation by the Tender Opening Committee;
  - o name of the supplier;
  - o brief description of the goods offered if the RFQ is for more than one Lot;
  - o the total price quoted; and
  - o any other appropriate information at the discretion of the Chairman.
- ❑ Any obvious failure to provide a responsive quotation shall be noted in the Minutes. These quotations shall normally be rejected.
- ❑ The Chairman and two Members of the Committee shall initial the quotation and all attachments thereto including any samples provided by the supplier. Any corrections to prices or obvious errors and omissions shall be circled in read ink and also initialled.
- ❑ Prepare a record identifying the samples provided by each supplier.
- ❑ Record the details read out of each quotation in the Register of RFQ Opening ensuring that amounts are recorded in words as well as figures, and record all corrections and errors or omissions which are noted in the Minutes of the Meeting.
- ❑ Minutes of the RFQ Opening shall be prepared by the Procurement Unit.

4.8.2 Evaluation of RFQ

Evaluation of quotations shall be undertaken by the Procurement Unit.

Action:

The Evaluation Panel should:

- ❑ Rank all quotations that are responsive to the Request for Quotations according to price and select the lowest evaluated priced RFQ for contract award.
- ❑ Prepare a brief report of the evaluation for the procurement record.

4.8.3 Award of Contract

The head of Procurement Unit should:

- ❑ Ensure availability of funds.
- ❑ Prepare a Purchase Order for signature by the Head of Procurement Entity.
- ❑ Issue the Purchase Order to the selected Supplier.

4.9 TENDER CLARIFICATIONS

The period during which tender clarifications may be requested will be defined in the Tender Document. Requests for clarifications received after this period may be ignored unless it draws attention to a serious flaw in the Tender Document. In all cases, any response to a tenderer must be communicated to all tenderers who received the Tender Document, but without identifying the tenderer who requested the clarification.

Action:

The Head of Procurement Unit should:

- ❑ Record each request for clarification and immediately develop a response in consultation with the appropriate Technical Department.
- ❑ The Procurement Unit should also consider if the proposed response to a request for clarification will require tenderers to undertake additional work, or make substantial modifications to their Tenders, which cannot reasonably be completed by the stated date for submission of Tenders. In this case, the Tender Committee should be consulted for approval to extend the date for submission of Tenders.
- ❑ Issue a written response to the Tenderer and notify details of the query and the response (but without identifying the source of the query) to all other tenderers simultaneously.
- ❑ Place copies of all correspondence on tender clarifications in the Procurement record file.

4.10 EXTENSION OF TENDER CLOSING DATE

The closing date for submission of Tenders may be extended at the discretion of the Procurement Entity for practical or justifiable reasons. The reasons may include modification to the Tender Document after issue and requests for an extension of time by tenderers.

Action:

The Head of Procurement Unit should:

- ❑ Ensure that there is an adequate practical justification for extending the closing date. Extensions should not be granted, for example, where a tenderer has by his own inactivity failed to purchase the Tender Document early enough to permit submission of a responsive tender.
- ❑ Issue an addendum notifying all tenderers of the revised date for submission of Tenders.

- Place copies of all relevant correspondence in the Procurement record file.

4.11 REJECTION OF TENDERS BEFORE TENDER OPENING

A Tender process may be cancelled at any time before the deadline for receipt of Tenders with the approval of the Tender Committee. The grounds for the rejection must have been stated in the Tender Documents.

Justifications for cancellation of tenders are:

- the procurement need has ceased to exist or changed significantly;
- insufficient funding for the procurement;
- significant change in the required technical details, tender conditions, conditions of contract or other details, such that the re-commencement of proceedings is necessary;
- there is evidence of collusion among tenderers; or
- Rejection is deemed to be in the interest of national security.

Action:

The Head of Procurement Unit should:

- Notify all tenderers who have purchased or received the Tender Document of the rejection, and refund any fees paid for the purchase of Tender Document.
- Return any tenders submitted to the tenderer unopened.
- Notify all Departments, Units and Projects involved in the procurement of the cancellation of tender proceedings.

4.12 RECEIPT OF TENDERS

All Tenders shall be deposited in the designated locked tender box (or such other arrangements as stated in the Tender Document) until the Tender Opening. The Procurement Unit will co-ordinate the Tender opening proceedings.

The Tender Document will have provided clear instructions to tenderers on the marking and sealing of Tenders and the procedures to be followed for submission.

The closing date and time for submission of Tenders must be determined in advance and stated in the Tender Document.

Action:

The Head of Procurement Unit should:

- Arrange placement of the tender box in a prominent place and ensure that all reception office staff and staff responsible for the receipt of tenders are fully aware of the Tender and their responsibilities for Tenders received.
- Ensure that the tender box is kept locked until the Tender Opening and that neither the tenderers nor staff have an opportunity to tamper with the box.

- ❑ Register all tenders received by post or by courier service on receipt, stamp with the date and time of receipt, and deposit the envelope unopened in the correct tender box.
- ❑ Direct all tenderers' representatives delivering Tenders by hand to the tender box so that they may place their envelopes directly in the tender box.
- ❑ Ensure that Tenders or samples received which are too large to fit in the tender box are registered and securely retained in a locked room under the control of the Procurement Unit until the Tender Opening.
- ❑ Close and seal the tender box immediately following the deadline for submission of Tenders and transport the tender box to the room where the Tender Opening will take place.
- ❑ If a single tender box is used for multiple Tender procedures, or the tender box is a fixture, open the tender box immediately following the deadline for submission of Tenders and remove all Tenders required for the Tender to be opened, and transport these to the room where the Tender Opening will be held.
- ❑ Ensure that Tenders or samples which were too large to fit in the tender box are also transported to the room where the Tender Opening will be held immediately following the deadline for submission of Tenders.

4.13 OPENING OF TENDERS

Tender Opening shall commence immediately after the close of the tenders (as stated in the tender document)

The Procurement Unit will co-ordinate the Tender Opening, ensure smooth operation of the proceedings, take a register of attendance, prepare Minutes of the opening, and advise the Chairman of the opening session on procedural issues if requested.

A Tender Opening Panel shall comprise at least 3 persons, including a member of the Entity Tender Committee. They shall ensure that minutes of the tender opening proceedings are duly written.

The Chairman of the Tender Opening Panel will control and direct the Tender Opening and not allow tenderer's representatives to interfere with the work of the Panel. Any objections by a tenderer to the procedures or decisions of the Tender Opening should be made in writing to the Head of the Procurement Entity.

For purposes of transparency it is not permitted for a tender opening to be halted or postponed once the process begins.

Action:

The Chairperson shall

- ❑ Open the meeting and outline the procedures to be used for the Tender Opening.
- ❑ Ensure that tenderers representatives are seated separately from the Tender Opening Committee and officials of the Procurement Entity, and that the

name, organisation represented and contact details of all attendees are recorded in an attendance register.

- Ensure the security of documents and Tenders at all times during the Tender Opening procedure to prevent any unauthorised interference with the documents and process.
- Bring in the unopened tender box and/or all Tenders and samples received which have been removed from the tender box or could not be accommodated in the tender box.
- Open the tender box, check that the inscription on each envelope or sample inside confirms that it matches the correct Tender and complies with the wording and sealing required in the Tender documents. Stack all envelopes in clear view of the Tenderers ready for opening. Samples supplied by Tenderers shall be stacked separately after checking to ensure that the Tenderer's name is clearly marked on each sample provided.
- Any Tenders not received by the deadline for the opening of Tenders must be rejected and returned unopened to the tenderer. Tenderers are not permitted to amend their Tender in any way during the Tender Opening or to submit any additional documents during the process.
- Check for any withdrawals or modifications submitted, and match these with the original Tender before proceeding. Withdrawn Tenders shall not be opened once the authenticity of the withdrawal notice has been confirmed.
- Open the first Tender after confirming that all conditions regarding marking and sealing of the envelope have been met and the envelope has not been tampered with. Stamp each copy of the Tender and annotate each Tender with a sequential serial number as it is opened (e.g. "1 of 7" where the total number of Tenders is 7).
- Examine the contents of the envelope and identify, stamp and number all originals and copies, and any separate sections and attachments.
- Read out the following details of each Tender from the Original copy:
  - any Tender modifications or withdrawals;
  - the number allocated to the Tender by the Tender Opening Committee;
  - the name and country of the Tenderer;
  - a brief description of the goods offered if the Tender is for more than one Lot;
  - the currency of the Tender;
  - the total Tender price;
  - any discounts offered;
  - the presence or absence of any required Tender security; and
  - any other appropriate information at the discretion of the Chairperson.
- Pass all Tender Securities to the representative of the Procurement Unit for review, registration and safekeeping.

- Any obvious failure to provide a responsive Tender, such as the absence of Tender security or inadequate Tender security, shall be reported to the Meeting and recorded in the Minutes.
- The Chairperson and two Members of the Committee shall initial the original and two copies of each Tender and all attachments thereto including any samples provided by the Tenderer. Any corrections to prices or obvious errors and omissions shall be circled in red ink and also initialled.
- Record the details read out of each Tender in the Register of Tender Opening ensuring that amounts are recorded in words as well as figures, and record all corrections and errors or omissions which were noted during the Tender Opening and captured in the Minutes of the Tender Opening.
- Mark any samples received identifying the supplier and the Procurement Number and record samples in the register.
- Any envelopes containing substitutions, or modifications, must be subject to the same level of scrutiny, including the reading out of critical details, such as price changes.
- Minutes of the Tender Opening shall be prepared by the Procurement Unit, signed by the Chairman of the Tender Opening Committee and made available to any tenderer involved in the Tender who requests a copy in writing.

4.14 TENDER EVALUATION

The Head of Procurement Entity shall constitute an Evaluation Panel Consisting of a minimum of three persons with the required expertise to conduct the evaluation.

It should be noted that tenderers frequently attempt to contact the Procurement Entity during Tender evaluation, directly or indirectly, to ascertain progress of evaluation, to offer unsolicited clarifications, or to provide criticisms of their competitors. Procurement Entities must evaluate Tenders solely on the basis of the information provided in the respective Tenders and no changes in the Tender price or substance of a Tender will be permitted. No circumstances shall justify meetings or consultation between the Procurement Entity (or its consultants) and tenderers during the Tender evaluation process.

4.14.1 Preliminary Examination of Tenders

The procedures for examination of tenders and clarifications must be conducted in accordance with the specific terms contained in the Tender Document used.

Action:

The Evaluation Panel should:

- Conduct a preliminary examination to determine whether tenders are complete and are responsive to the basic instructions and requirements of the Tender Document including checks that:

- the Tenderer is eligible to tender where this has not been determined prior to inviting tenders;
- all goods offered originate from eligible source countries;
- the Tender has been submitted in the correct format and there are no missing pages;
- any erasures, interlineations, additions or other changes in the Tender have been initialled by the Tenderer;
- any required Tender Security has been submitted, in the correct form and amount and valid for at least the period required;
- the Tender has been submitted without material reservations or deviations from the terms and conditions of the tender document;
- the Tender has been correctly signed and authorised;
- if the tenderer is a joint venture, a copy of the joint venture has been submitted;
- if the tenderer is an agent, an authorisation from the manufacturer has been provided;
- the correct number of copies of the Tender have been submitted;
- the Tender is valid for at least the period required;
- all key documents and information have been submitted;
- any required samples have been submitted; and
- the Tender meets any other key requirements of the Tender Document.

- ❑ If a prequalification procedure was applied, reject and exclude any Tender received from other than the prequalified tenderers.
- ❑ may request clarifications from tenderers concerning ambiguities or inconsistencies in the Tender. As required in the Tender Document, such requests shall be in writing, and no change in the price or scope of the originally offered goods, works, or services may be sought or accepted, except for the correction of arithmetic errors. The responses from tenderers shall also be in writing.
- ❑ Notify tenderers of any such arithmetic corrections and request written, agreement of the tenderer to the correction. If a tenderer does not accept the correction of an arithmetical error, his Tender must be rejected and the Tender Security may be forfeited.

Any communications between the Procurement Entity and a tenderer during the preliminary examination of Tenders shall be made in writing.

4.14.2 Responsiveness of Tenders

Following the preliminary examination of tenders, the Tender Evaluation Panel should examine the tenders to determine whether any issues arising from the preliminary examination affect the responsiveness of an individual tender, and whether each tender is substantially responsive to the technical specification and contract conditions stated in the Tender Document.

- The determination of a Tender's responsiveness is based on the contents of the Tender itself, subject to any clarifications received in the Preliminary Examination of Tenders.
- A substantially responsive Tender is one that conforms to all the instructions, requirements, terms and conditions of the tender documents, without material deviation, reservation or omission. A material deviation, reservation, or omission is one that:
  - affects in any substantial way the scope, quality, or performance of the works, services or supplies specified in the tender documents; or
  - would limit in any substantial way, inconsistent with the tender documents, the rights of the Procurement Entity or the tenderer's obligations under any resulting contract; or
  - if corrected would unfairly affect the competitive position of other tenderers presenting substantially responsive and compliant Tenders.
- Any tender containing a material deviation, reservation or omission, is therefore not substantially responsive, shall be rejected and may not subsequently be made responsive by the tenderer or the Procurement Entity.
- The classification of a deviation, reservation or omission as material or non-material shall be determined by the objectives and requirements of the individual procurement requirement, as stated in the Tender Document, and shall take into account the impact on key factors, such as cost, risk, time and quality.

Material deviations, reservations or omissions may typically include:

  - unacceptable time schedules for delivery compared with requirements stated in the Tender Document;
  - unacceptable alternative technical details, such as inferior design, materials, workmanship, specifications, standards or methodologies; or
  - unacceptable counterproposals with respect to key contract terms and conditions, such as payment terms, price adjustment, liquidated damages, sub-contracting or warranty.

Non-material deviations may include:

  - longer delivery schedules unless this takes delivery past a clearly defined cut-off date stated in the Tender Document;
  - minor deviations in the payment terms required;
  - tenders offering goods to alternative, but equal or superior specifications and performance;
  - tenders offering the specified goods but omitting minor attachments or components (e.g. a missing toolkit for a vehicle).

- The classification of deviations, reservations and omissions as material or non-material must be consistently applied to all Tenders.

- Where a Tender is determined to be substantially responsive, the Evaluation Panel may waive, clarify or correct any non-conformity, error or omission, which does not constitute a material deviation. Such non-conformity, error or omission should be quantified in monetary terms to the extent possible and taken into account in the financial evaluation and comparison of Tenders.

Action:

The Evaluation Panel may:

- Reject and exclude from further evaluation any Tender that is incomplete, unsigned, not accompanied by a Tender Security, not accompanied by essential supporting documents, or not substantially responsive to the technical specifications, contract conditions or other critical requirements stated in the Tender Document.
- Reject and exclude from further evaluation any Tender which contains material deviations, reservations or omissions, and is therefore not substantially responsive.

4.14.3 Financial Evaluation of Tenders

Tenders that are determined to be substantially responsive to the requirements of the Tender Document shall be subjected to financial evaluation to determine the evaluated price of each Tender, and the lowest evaluated tender.

The evaluated price for each Tender is determined by:

- taking the Tender price, as read out at the tender opening;
- correcting any arithmetic errors, in accordance with the methodology stated in the Tender Document;
- applying any non-conditional discounts offered in the Tender;
- making financial adjustments in accordance with the Tender Document for any non-material non-conformity, error or omission. Pricing of components or features which are omitted in a Tender should be based on the highest prices for corresponding components, items, etc., in other responsive Tenders.
- If these are not available, pricing from external sources such as printed parts list, price lists, etc should be used. No additional credits should be given for offered features that exceed the required standards or specification, e.g., additional horsepower or capacity, unless there is a specific provision for this in the Tender Document;
- converting all Tenders to a single currency, using the currency and the source and date of exchange rate indicated in the Tender Document; and
- applying any margin of preference indicated in the Tender Document.

Tenders shall be compared by ranking them according to their evaluated price to determining the Tender with the lowest evaluated price.

5.14.4 Treatment of Discounts

Tenderers may be permitted to offer discounts to their Tenders, which shall be included in the Tender and read out at the tender opening.

Any discount offered after the deadline for submission of Tenders must not be taken into account.

Any non-conditional discounts shall be taken into account in the financial evaluation and comparison of tenders.

Any prompt payment discount included in a Tender shall become a term of the contract, if that Tender is accepted, and utilised by the Procurement Entity, if payment is made in accordance with the terms of the discount. However, prompt payment discounts must not be considered in the evaluation and comparison of tenders.

4.14.5 Post -Qualification (Repeat Tender Qualifications)

Post-Qualification of the lowest evaluated responsive tenderer should be conducted to determine the tenderer's capability to perform the contract. Using the criteria specified in the Tender Document, this review should include an assessment of the tenderer's financial and physical resources available to undertake the contract, including his current workload.

- ❑ If pre-qualification was conducted, the lowest evaluated responsive Tender should be recommended for the award of contract, unless the tenderer's qualifications have since materially deteriorated, or if the tenderer has since received additional work that reduces the available capacity.
- ❑ Where pre-qualification has not taken place, the lowest evaluated responsive Tender should be subjected to post-qualification, according to the procedures described in the Tender Document.
- ❑ If the lowest evaluated responsive tenderer fails post-qualification, his Tender should be rejected, and the next ranked tenderer should then be subjected to post-qualification examination. If successful, this tenderer should receive the award. If not, the process continues for the other tenderers.
- ❑ The rejection of a Tender for reasons of qualification requires substantial justification, which should be clearly documented in the attachments to the Evaluation Report.
- ❑ A history of poor performance may be considered a justification for failing post-qualification if the tenderer is unable to demonstrate that steps have been taken to resolve previous problems.

4.14.6 Evaluation Reports and Recommendations

The Tender Evaluation Panel will prepare an evaluation report for submission to the Tender Committee.

The evaluation report should be prepared using the standard format for the Evaluation of Goods, with relevant narrative and include:

- ❑ a summary of the Tenders received and opened;
- ❑ the results of the preliminary examination;
- ❑ the results of the technical evaluation;

- reasons why any Tenders were declared non-responsive. (Attach copies of selected pages from Tenders if necessary, to show examples of objectionable features);
- details of any non-material deviations, which were accepted and the way in which they were quantified and taken into account in the financial evaluation;
- the evaluated price of each Tender, showing any corrections or adjustments to the tender price and any conversion to a common currency;
- An explanation of:
    - any inconsistencies between prices and modifications to prices read out at Tender opening.
    - any substantial corrections for arithmetic errors which may affect the ranking of Tenderers.
    - any additions, adjustments, and priced deviations that may affect the ranking of Tenderers.
    - any cross-discounts not read out and recorded at the Tender opening.
- the ranking of the Tenders, according to their total evaluated price;
- a statement of the lowest evaluated substantially responsive tender, for each lot where applicable, clearly establishing the actual amount of the contract award;
- confirmation that the total price quoted by the lowest evaluated responsive Tender is acceptable compared with the original estimated cost or the procurement;
- the results of any post-qualification examination; and
- a recommendation to award the contract or contracts to the lowest evaluated responsive Tenderer or combination of Tenderers, or other appropriate recommendation, such as the cancellation of the procurement process. The Evaluation Panel may recommend that all of the Tenders be rejected if:
    - none of the Tenders are found to be responsive;
    - all of the Tenders are unreasonably high in price compared to the cost estimate;
    - none of the Tenderers are qualified;
    - there is deemed to be an absence of competition, (however lack of competition is not usually decided solely on the basis of the number of Tenderers).

The following are to be attached to the evaluation report:

- copies of any correspondence with tenderers who raised objections to the Tender or evaluation process, together with detailed responses;

- o copies of any letters from tenderers requesting clarifications, and responses by the Procurement Entity, and Tender clarifications requested by the Evaluation Panel and Tenderer responses;
- o any separate evaluation report from a consultant, if one was engaged for this purpose;
- o all required Tables and supporting documents.

- ❑ Tender evaluation report should be double-checked, signed and complete before submitting for approval by the Tender Committee.

The evaluation report forms a part of the record of procurement proceedings required under Section 28 of the Act.

4.15 REVIEW AND APPROVAL OF EVALUATION REPORT

The appropriate review authority (Head of Procurement Entity, Tender Committee, District, Regional, Ministry or Central Tender Review Board) in accordance with the thresholds stated in the schedule, will review the evaluation report and recommendations presented in the Evaluation Report. The approval shall be 'One stop' only. i.e. concurrent approvals must be obtained.

The review authority, before giving approval, will ensure that any written complaints made by tenderers concerning the tender process have been addressed and responded to. If the complaint reveals a serious breach of procedures or ethics, the review authority may recommend rejection of the evaluation report and call for re-tendering.

Action:

The Review Body will:

- ❑ check the accuracy of the evaluation and recommendations as submitted;
- ❑ decide whether the price increase is justified if the price of the lowest evaluated responsive Tender is higher than the original estimated cost. If the price is not acceptable, recommend that the Tender should be cancelled.
- ❑ Review scope and re-advertised,
- ❑ reject any evaluation report which contains serious inaccuracies or flaws and return to the Tender Evaluation Panel with written comments for correction and re-submission;
- ❑ ensure that all discussions and decisions of the review authority are reported in the minutes of the meeting.

4.16 EXTENSION OF TENDER VALIDITY

The duration of tender validity is specified in the Tender Document and should be confirmed in the signed Tender Form submitted by each Tenderer.

- ❑ If circumstances occur in which award cannot be made within the original tender validity period, extensions in writing should be requested from Tenderers, in accordance with the Tender Document.

- The evaluation and award of contract should be completed within the period set for the validity of tenders. The date for expiry of tender validity must be monitored and attention drawn to this deadline not less than two weeks before the expiry date.
- If, due to unforeseen circumstances, the task cannot be completed within the set period, the Procurement Unit may contact tenderers to seek their agreement to an extension of the tender validity.
- Tenderers who refuse this request may withdraw from the Tender without incurring any penalty, but tenderers who agree to an extension will also be required to extend their Tender Securities for an appropriate period.
- When an extension of tender validity period is requested, tenderers shall not normally be requested or be permitted to change the quoted price or other conditions of their tenders. However, the Tender Document may provide for an appropriate price adjustment mechanism when requests for second or subsequent extensions are made, to reflect changes in the cost of inputs for the contract over the period of extension.

Action by Procurement Unit:

- Maintain a diary reminder of all tender validity dates and advise the responsible officer not less than two weeks before the expiry date so that appropriate action can be taken in time.
- Determine the additional time required to complete the evaluation and award of the specific contract. Any extension of tender validity should be for the minimum period required to complete the evaluation, to obtain the necessary approvals and to award the contract.
- Prepare an individual letter to each tenderer requesting their formal approval to an extension of the validity of tenders and an extension of their Tender Securities (where necessary) for the required period. Set a date for written responses to be received by the Procurement Entity, stressing that tenderers who do not respond in time will be considered to have withdrawn their tenders.
- Send the letter by registered post or deliver by hand to each tenderer and obtain a receipt.

Tenderers have the right to refuse to grant an extension of tender validity without forfeiting their Tender Security. If a tenderer refuses to extend the validity of his tender then, upon expiry of the original tender validity period, the Tender Security shall be returned and the Tender not considered further.

Only tenderers who respond confirming their unconditional acceptance and enclosing any required extension to their Tender Security may be considered for further evaluation and award of contract.

5.17 NEGOTIATIONS

An entity shall not negotiation on price with the lowest evaluated responsive tenderer, with respect to a tender submitted. If the lowest evaluated tender exceeds the budget for the contract by a substantial margin, the procuring entity shall investigate the causes for the excessive cost and may:

- consider requesting new tenders: or
- subject to approval by the relevant Tender Review Board and guidelines issued by the Public Procurement Board, negotiate a contract with the lowest evaluated tenderer to try and obtain a satisfactory contract.

In no circumstances shall negotiations be conducted with two tenderers simultaneously.

4.18 AWARD OF CONTRACT

Following approval from the relevant review body, the contract will be awarded to the Tenderer who has submitted the lowest evaluated Tender.

Before contacting the Tenderer, a formal commitment of the required funds against the budget of the Procurement Entity must be approved.

Action:

The Head of Procurement unit should:

- ❑ Prepare an Activity and Expenditure Initiation (A&EI) Form and obtain approval of the commitment of funds against the budget of the Procurement Entity.

4.18.1 Preparation of a Contract

The Contract Documents should clearly define:

- the goods to be supplied;
- the rights and obligations of the Procurement Entity and of the Supplier;
- a clear statement on when the contract become binding is important because certain contract clauses could be connected with this date, e.g. Delivery Time, Warranty, Validity of performance Guarantee, etc.

The Contract form in the tender document is to be regarded as a checklist, for the preparation of a final Contract.

Actions:

The Head of Procurement unit should:

- ❑ Complete the form of contract entering details of the selected tenderer and all agreed terms and conditions.

The following documents shall be deemed to form part of the contact:

- the Tender Form and Price schedule submitted by the tenderer.
- the Schedule of Requirements

- the Technical Specification
- the General Condition of Contract
- the Special Condition of Contract: and
- the Notification of Award.

Prepare four copies of the contract ready for signature by each party to the contract

- ❑ Obtain the signature of the Head of Procurement Entity on all copies of the contract.

The eventual distribution of the four copies (minimum) of the Contract will be:

- Original – Procurement Unit;
- 1st Duplicate – Supplier/Contractor;
- 2nd Duplicate – the Technical Department concerned; and
- 3rd Duplicate – Legal Officer.

5.18.2 Award Notification

Notice of the tender award shall be issued promptly to the successful Tenderer. The successful Tenderer shall be required to confirm in writing acceptance of the tender award and submit the appropriate Performance Security (if required).

- ❑ The Tenderer shall be invited to attend for contract signature, or where this is not practical, provided with all four copies of the Contract for signature and return of three signed copies of the Contract to the Procurement Entity.
- ❑ Failure of the Tenderer to confirm acceptance of the award, or to submit the Performance Security, or to sign the contract may constitute grounds for the annulment of the award and forfeiture of the Tenderer's Tender Security. In that event, the Procurement Entity may award the contract to the next lowest evaluated Tenderer, whose offer is substantially responsive and is determined to be qualified to perform the contract satisfactorily.

Procurement Entities are required to submit notice of contract awards to the Public Procurement Board within 30 days of contract signature, for publication on the PPB website and/or in the Procurement Bulletin. The information on contracts awarded/signed shall include the:

- name of the Supplier;
- description of the goods;
- Contract Sum;
- duration of the contract;
- method of procurement used; and
- the source of funding.

4.18.3 Notification to Unsuccessful Tenderers

All unsuccessful Tenderers should be notified immediately once the contract has been awarded. Tender securities of unsuccessful Tenderers should be promptly returned after awards have been made.

4.18.4 Return of Tender Securities

All Tender Securities must be returned immediately to the Tenderer, as soon as they are no longer required, i.e. once the relevant contract is signed and all conditions for contract effectiveness have been met.

4.19 CONTRACT MANAGEMENT

Effective management of contracts is essential to ensure that the objectives of the procurement process are achieved and that all contractual obligations and activities are completed efficiently by both parties to the contract. The Procurement Unit or the Technical Department concerned must ensure that routine monitoring of all current contracts is maintained so that swift remedial measures can be taken when problems arise, or preventative action taken when problems are foreseen.

There are many post-contract issues that need to be dealt with, monitored and resolved before the contract reaches its conclusion including:

- Contract Effectiveness;
- Delivery and Inspections of Goods;
- Insurance Claims;
- Payments to the Supplier;
- Contractual Disputes;
- Delays in Performance;
- Claims for Damages;
- Installation and Commissioning of Equipment;
- Release of Performance Securities and Retentions;
- Contract Closure.

4.19.1 Contract Effectiveness

Although the Contract may have been signed by both parties, the legal effectiveness of the Contract may be dependent on one or more of the following conditions:

- Receipt by the Procurement Entity of the Performance Security;
- Receipt by the Procurement Entity of an Advance Payment Security;
- Receipt by the Supplier of the Advance Payment; or
- Receipt by the Supplier of an acceptable Letter of Credit.

Actions:

The Head of Procurement Unit should ensure that:

- ❑ any Performance Security specified in the contract is received by the Procurement Entity;
- ❑ any Advance Payment specified in the contract is paid immediately when the Advance Payment Security is received from the Supplier or Contractor;
- ❑ any Letter of Credit required is issued promptly.

4.19.2 Contract Supervision and Administration

Contract supervision and administration will be undertaken by the Procurement Unit and the Technical Department as appropriate.

Supervision and administration is straightforward in most procurement of goods but monitoring delivery schedules, processing of documents and the inspection of goods are essential to ensure that the correct goods are delivered on time.

Action:

The Head of Procurement Unit should:

- ❑ Monitor the delivery schedules of all purchases to ensure that they are dispatched and delivered or collected on time.
- ❑ Receive reports on destination inspection of goods and contact the Supplier in writing requesting rectification of any discrepancies or deficiencies.
- ❑ Contact the Supplier or shipper to identify the causes of any delay in delivery;
- ❑ Initiate and supervise any process for claims against insurance or the supplier.

4.20 RECEIPT AND INSPECTION OF GOODS

The Stores Department is responsible for the receipt of goods with assistance from the Procurement Unit and the User Department representatives.

4.20.1 Collecting Goods from the Port of Entry

Port and customs clearance of goods and physical collection at the Port of Entry may be necessary in some circumstances depending on the INCOTERMS used.

Before accepting the consignment the Procurement Entity should:

- ❑ Carefully examine the packages to check they are all present according to the Shipping Documents or Airway Bill and to look for signs of any physical damage.
- ❑ Look also for signs of tampering with any of the packages e.g. cases with broken seals, loose or open tops.
- ❑ Have the packages re-weighed if there is any suspicion that items have been removed, and compare the result with the shipping documents.
- ❑ Note any deficiency either in quantity or in condition on the receipt given in exchange for the goods (a claused receipt).

- If there are shortages, in addition to a claused receipt, a short-landed or discrepancy certificate should also be obtained to assist claims against the Supplier or insurance companies.

4.20.2 Delivery of Goods

On delivery by a Supplier or carrier, the Storekeeper will:

- Receive the goods into temporary storage.
- Examine the apparent condition of the goods and packing.
- Issue an interim receipt, noting the apparent condition, pending the meeting of an Inspections team.
- Receive and register any invoices pending processing (through the Procurement Unit) to the Accounts Department for payment following the report of the Inspections team

4.20.3 Inspection of Goods

An Inspections team [composed of representatives of the Procurement Unit, User Department and the Stores] should meet within two days following the arrival of the goods, and in the presence of the Supplier's representative if he chooses, will:

- Examine the documentation and packaging for compliance with the contract.
- Ask the Supplier to open the packages (or arrange opening of the packages at the Supplier's expense).
- Examine and analyse the goods for conformity with the contract specifications and/or the samples provided.
- Co-ordinate any required laboratory analysis of goods;
- Reject all goods that are damaged or do not conform with the required specifications or samples.
- Prepare an Inspection Report with an original and three copies, recording the delivery, and descriptions, specifications and quantities of the goods examined, and the reasons for accepting or rejecting the goods.
- Release accepted goods to the Stores which will take the items on charge by issue of a Goods Received Note and entry into the Stores Ledger

Any rejected goods should be removed by the Supplier within one week. If the goods are not removed, and it is specified in the contract, the Supplier may be charged a penalty for delay. If the rejected goods are not removed after a reasonable period, the Procurement Entity may apply to a Court of Law for authority to sell the rejected goods.

The Inspections team will follow similar procedures to examine equipment that is delivered to a site or already installed by the Supplier.

4.20.4 Issue to the User Department by Stores

The Stores Department is responsible for maintenance of records of all goods assets held under the custody of individuals, Units, Departments and Entities.

Assets will be taken on charge in the records of the concerned Stores and subject to regular review and audit.

The Storekeeper will:

- ❑ Issue or release items to the User Department which initiated the procurement using a Stores issue Voucher for the dispensation of goods from the Stores.
- ❑ Record the issue from the Stores Ledger and the receipt in the Custody Ledger for the individual, Unit, Department or Programme of the Procurement Entity.

4.21 PAYMENT FOR GOODS

For payments against invoices, the Procurement Entity will:

- ❑ Complete a Payment Voucher ensuring the deduction of any advance payments already made and any contractual penalties incurred by the Supplier.
- ❑ Attach the:
  - o original invoice from the Supplier;
  - o the shipping documents or airway bill, or a delivery note;
  - o original of the Inspection Report;
  - o original Goods Receipt Note;
  - o results of any laboratory analysis;
  - o calculation of any penalties for rejected goods not removed by the Supplier and liquidated damages if allowable under the contract;
  - o copies of relevant information from the contract document, records of approval and financial authorisations.
- ❑ Record the payment in the Contract Register.
- ❑ Forward the Payment Voucher to the Accounts Department for processing.

4.21.1 Payments by Letter of Credit

Letters of credit are documentary credit which represents a commitment of a bank to pay the supplier a certain amount provided the supplier presents stipulated documents evidencing the shipment of the goods within a prescribed period of time.

- ❑ Documentary credit transaction always involves three parties, the issuing bank, (which is normally the buyers bank) the applicant for the credit (usually the buyer) and the beneficiary of the credit (usually the seller).
- ❑ A fourth party may be a second bank, the corresponding (and, sometimes confirmer) of the issuing or originating bank corresponding and confirming banks are usually situated in the seller’s country.
- ❑ Payments by Letter of Credit will depend on the contract agreement between the supplier and the procuring entity and the terms and wording of the Letters of credit.

Action:

The Accounts Department:

- Ensures that there are sufficient funds at the Bank, for the establishment of the Letters of Credit, for all contracts requiring payment by letters of credit
- Completes Letters of Credit forms and ensures that the forms are signed by the authorised signatories to the account. (The forms can be collected from the issuing bank),
- Writes a cover letter instructing Issuing Bank to credit the supplier with the required amounts, upon receipt of the following documents from the supplier.
    a. Invoices
    b. Packing Lists
    c. Certificate of origins
    d. Insurance policy
    e. Bill of Lading/Airway bill
- Collects documents from issuing bank when delivered by supplier

4.22 DELAYS IN PERFORMANCE

Delivery of goods should be completed by the Supplier in accordance with the time schedule prescribed in the Schedule of Requirements. Where this is not the case:

- In accordance with the contract conditions, the Supplier must notify the Procurement Entity in writing of the conditions delaying performance, including full details of the delay, the likely duration and the cause(s).
- The Procurement Entity will immediately assess the situation, and may at its discretion extend the Supplier's time for performance, with or without liquidated damages as specified in the Contract.
- If the time for performance is extended, both parties shall ratify such extension by a formal addendum to the Contract subject to approval by the Tender Committee.
- A delay by the Supplier in the performance of his obligations may render him liable to liquidated damages or his/her performance bond redeemed if specified in the contract document, except where:
    - the delay is as a result of Force Majeure;
    - there is no provision for liquidated damages in the contract;
    - there is no provision for a performance bond.
    - an extension of time is agreed between the two parties without the application of liquidated damages.

The Head of Procurement Unit should:

- Refer to the relevant clauses in the General or Special Conditions of Contract for the procedure to be followed to apply, calculate and claim liquidated damages.

- ❑ Update the Procurement file and Contract Register to reflect any delays in the supplier's performance.
- ❑ Notify the end-user department immediately of all such delays.

4.23 RESOLUTION OF CONTRACTUAL DISPUTES

Most minor disputes may be resolved by discussion and agreement between the responsible officer and the Supplier to rectify the cause of complaint.

Any formal written complaints received from a Supplier should be fully investigated and referred to the Head of the Procurement Entity to authorise correspondence or formal negotiations with the Supplier.

Action:

The Head of Procurement Unit should:

- ❑ Examine the Contract carefully to be aware of all contract conditions relating to the Resolution of Disputes.
- ❑ Determine if the Procurement Entity is at fault or partly at fault, and if so, take appropriate action to rectify the problem.
- ❑ Invite the Supplier to a formal meeting, within 7 days of the complaint, to discuss the issues and try to agree a compromise acceptable to both parties. Ensure that accurate written Minutes are kept of any such meeting. If an agreement is reached which changes any of the conditions of the Contract, approval of the Tender Committee or the Head of the Procurement Entity is required before the agreement can be implemented.
- ❑ If no initial agreement is reached and negotiations conducted by the Head of the Procurement Entity also fail, consider the use of any conciliation or arbitration services as specified in the contract.
- ❑ Prepare any necessary addendum to the Contract for signature.

4.24 TERMINATION OF THE CONTRACT

The parties to the contract normally have the right to terminate the contract, but to protect the Procurement Entity, advice of the Legal Department should always be sought if the Procurement Entity is considering such action. Contracts should not be terminated without examining all possible alternatives, unless the termination is mutually agreed by all parties to the contract.

Action:

The Head of Procurement Entity should:

- ❑ Examine the Contract carefully to be aware of all contract conditions and penalties relating to the Termination of Contract.
- ❑ Follow the advice of the Legal Officer/ Ministry of Justice in the preparation of any correspondence and settlement of any contractual penalties.

4.25 CONTRACT AMENDMENT

Contract amendment may become necessary as a result of the application of price fluctuations specified in the contract, the resolution of disputes, additional or reduced requirements by the Procurement Entity, agreements to extend the time schedule, or from accepted increases or decreases in prices. The contract may allow the Procurement Entity to modify contract values by a pre-determined percentage when this is in the public interest and essential for the work of the Procurement Entity.

All other amendments to costs, quantities, time-periods and other terms and conditions of the contract must be approved by the Tender Committee and confirmed in a formal contract amendment or addendum.

The Procurement Entity will:

- identify and agree with the Supplier the specific clauses in the contract which need to be changed, and the new values or terms and conditions which are to apply;
- prepare a draft contract amendment document for approval by the Tender Committee together with a report justifying the reasons for the amendment,
- following approval by the Tender Committee:
    - record any change in contract value in the Commitment Register and in the Contract Register;
    - record any other contractual changes in the Contract Register;
    - obtain from the supplier/contractor any necessary addition to the performance security;
    - arrange for signature of the contract amendment in four copies;
    - distribute copies in the same way as the original contract.

CHAPTER 5:PROCUREMENT OF WORKS

5.1 DEFINITION OF WORKS

Works means work associated with the construction, reconstruction, demolition, repair or renovation of a building or structure or surface and includes site preparation, excavation, erection, assembly, installation of plant, fixing of equipment and laying out of materials, decoration and finishing, and any incidental activity under a procurement contract.

5.2 PROCUREMENT METHODS

The procurement methods that can be used for the procurement of Works are:

- International Competitive Tendering (ICT)
- National Competitive Tendering (NCT)
- Two-Stage Tendering (National or International)
- Restricted Tendering (National or International)
- Single Source (Direct Procurement)
- Request for Quotations (RFQ)

Competitive tendering using ICT or NCT is the preferred method for public procurement and the use of alternative methods is strictly limited to the provisions of Part IV of the Public Procurement Act.

5.2.1 International Competitive Tendering

International Competitive Tendering is appropriate for high value or complex procurements, or where the works by their nature or scope, are unlikely to attract adequate local competition. The Act requires the use of ICT for procurement of work above the threshold stated in Schedule 3.

5.2.2 National Competitive Tendering

National Competitive Tendering is appropriate for lower value procurements, where the works by their nature or scope are unlikely to attract foreign competition, or where there are justifiable reasons for the Procurement Entity to restrict tendering to domestic contractors. The Act permits the use of NCT for procurement of works valued between the thresholds stated in Schedule 3.

5.2.3 Restricted Tendering

Restricted Tendering is a tendering process by direct invitation to a shortlist of pre-registered or known contractors, and is subject to a specific approval being granted by the Public Procurement Board:

It is an appropriate method of procurement where:

- the requirement is of a specialised nature or has requirements of public safety, or public security which make an open competitive tender inappropriate;
- due to the urgent nature of the requirement, an open competitive tender is not practical;

- ❑ the number of potential contractors is limited; or
- ❑ an open competitive tender has failed to bring an award of contract.

5.2.4 Two-Stage Tendering

Two-stage Tendering is an infrequently used procurement process in which a Procurement Entity invites tenderers in the initial stage to contribute to the detailed specification of the works. Following review and consultations, new detailed specifications for the works are prepared and a restricted tender issued in the second stage to all participants who were not rejected in the first-stage.

It is an appropriate method of procurement when it is not feasible for the Procurement Entity to formulate detailed specifications or plans for the works, to identify their characteristics in a defined manner, or the subject of the works is subject to rapid technological advances.

5.2.5 Single Source

Single source procurement from a supplier without competition (direct procurement) is subject to a specific approval using the Guidelines issued by PPB.

Single source procurement may be appropriate when:

- ❑ the purchase is for urgently needed remedial works, provided this is restricted to the minimum requirement to meet the urgent need until a procurement by other methods can be fulfilled; or
- ❑ the works can only be provided by one source for physical, technical or policy reasons. e.g. requiring the use of proprietary techniques that are obtainable only from one source.

5.2.6 Request for Quotations (RFQ)

This is also known as "shopping" and is based on comparing price quotations obtained from several suppliers, usually at least three, to ensure competitive prices.

Request for Quotations may be used when:

- ❑ the estimated value is less than the threshold specified in Schedule 3 of the Act; and
- ❑ the requirement is for widely available works activities such as redecoration, repairs, and minor alterations which do not require detailed specification and may be readily estimated by a contractor from a simple site visit.
- ❑ For higher value or more complex requirements, it is recommended that a more appropriate Standard Tender Document and contract format should be used to protect the Procurement Entity.

5.3 SPECIFICATION OF REQUIREMENTS

The requirement needs to be fully identified with detailed technical specifications, drawings, and plans as appropriate.

The Technical Department of the Procurement Entity should prepare plans, drawings, detailed specifications and a Bill of Quantities or Activity Schedule for the required works. If the Procurement Entity does not have a recognised Technical Department for this purpose, the assistance of technical ministries - e.g. the Ministry of Water Resource, Works and Housing should be solicited for this task.

5.4 PRE-QUALIFICATION OF CONTRACTORS

Pre-qualification is required for works contracts estimated at above the threshold value stated in Schedule 3 of the Act. However, prequalification proceedings may be used on lower value contracts to identify tenderers who are qualified prior to the submission of tenders.

Pre-qualification

- ❑ is appropriate for large or complex works, or in other circumstances when the high costs of preparing detailed tenders could discourage competition;
- ❑ is often used for contracts to be let under turnkey, design and build, or management contracting;
- ❑ ensures that invitations to tender are extended only to those contractors who have adequate capabilities and resources;
- ❑ helps to ensure that only tenders from competent contractors are considered and eliminates disputes that may arise from rejection of lowest tenderers because the tenderers are not considered qualified;
- ❑ may be required of development partner- funded procurements.

5.4.1 Pre-qualification Criteria

Pre-qualification is based upon the capability and resources of prospective tenderers to perform the particular contract satisfactorily, taking into account their:

- ❑ experience and past performance on similar contracts;
- ❑ capabilities with respect to personnel, equipment, and construction facilities;
- ❑ required licensing and professional registrations; and
- ❑ commercial and financial resources.

5.4.2 Advertisement for pre-qualification

The invitation to pre-qualify for tendering on specific contracts or groups of similar contracts shall be advertised as in Competitive Tendering. The scope of the contract and a detailed statement of the requirements for qualification must be provided in the advertisement.

5.4.3 Pre-qualification evaluation

No formal opening of submissions is required but a formal evaluation of the capabilities of each supplier is required by the Technical Committee against the criteria as stated in the advertisement.

- Where a large number of contractors pass the stated qualification criteria, it may not be realistic to invite more than eight to participate in the specific tender. If so, only the best qualified contractors should be selected and invited to tender.
- Inform all applicants of the results of pre-qualification.. Applicants who fail to meet the pre-qualification criteria should be briefly advised of the reasons. No further correspondence will be entered into regarding applications.
- When pre-qualification is completed, issue the Tender Document to all qualified contractors, or to an appropriate shortlist of the qualified tenderers.

5.4.4 Advertisement for pre-qualification

The invitation to pre-qualify for Tender on specific contracts or groups of similar contracts shall be advertised in the same way as for Competitive Tendering and shall contain the:

- name and address of the Procurement Entity;
- nature and location of the works to be effected;
- desired or required time for the completion of the works;
- criteria and procedures to be used to evaluate the qualifications of contractors;
- means to obtain the pre-qualification documents and the place from where they can be obtained;
- price charged by the Procurement Entity for the pre-qualification documents (which shall reflect only the cost of printing and provision to contractors);
- currency and terms of payment for the pre-qualification documents;
- language in which the pre-qualification documents are available;
- place and deadline for the submission of applications to prequalify and the time allowed for the preparation of pre-qualification applications (which shall not be less than four weeks).

5.4.5 Pre-qualification document

A procurement entity shall supply a set of prequalification documents to each contractor that requests them. The pre-qualification documents shall include:

- instructions to prepare and submit prequalification applications;
- a summary of the main terms; qualifications and conditions required for the procurement contract to be entered into as a result of the procurement proceedings;
- any documentary evidence or other information that must be submitted by contractors to demonstrate their qualifications;
- the manner and place for the submission of applications to pre-qualify and the deadline for the submission, expressed as a specific date and time which allows sufficient time for contractors to prepare and submit their

applications, taking into account the reasonable needs of the Procurement Entity;

- any other requirement that may be established by the Procurement Entity in conformity with the Act and Regulations relating to the preparation and submission of applications to pre-qualify and to the prequalification proceedings.

5.5 TENDERING PROCEDURES

5.5.1 Standard Tender Document

A Standard Tender Document is the set of documents issued by a Procurement Entity, which establishes the works required (technical specifications, plans, bill of quantities or activity schedule), the procurement procedures to be followed, and specifies the proposed contract conditions. It should contain all information necessary to allow the tenderer to prepare a responsive tender or quotation.

The Standard Tender Document must contain information to:

- instruct tenderers on the procedure for submission of tenders;
- describe the works to be procured;
- inform tenderers of the criteria for evaluation of tenders; and
- define the conditions of any resulting contract.

Use of a Standard Tender Document (STD) format, as defined in Schedule 4 of the Act, is mandatory for all procurement of works funded by Government, unless an alternative format has been specifically approved by the Public Procurement Board. Note that procurement of works using development partners funds may be subject to the required use of their standard formats if so specified in the loan or credit agreement, in accordance with Section 96 of the Act.

The updated versions of each approved STD, together with an individual User Guide to assist in preparation of the STD, may be obtained on electronic media from the Public Procurement Board, or directly downloaded from the PPB website. (www.ppbghana.org)

5.5.2 Preparation of Tender Document

The preparation of tender documents is the responsibility of Head of Procurement Unit. Upon receipt of requisition to procure and notification of availability of funds, the Head of Procurement proceeds with the preparation of the document.

The tender document must be prepared by modifying the appropriate Standard Tender Document (STD) issued by the PPB. The STDs as listed in schedule 4 of Act 663, may be obtained directly from the PPB or downloading it from the PPB website. (www.ppbghana.org)

The key sections that need completion and modifications are;

- The Invitation to Tender,
- The Tender Data Sheet.
- Technical Specification/Drawings
- The Bill of Quantities.
- The Conditions of Particular Application
- Forms of Tender, Tender Security, Forms of Agreement and Performance

Security (to be completed by the Contractor)

Actions

The Head of Procurement should:

- ❑ Select the appropriate standard tender document depending on the type of procurement.
- ❑ Complete the Invitation to tender section with the relevant information which is self explanatory.
- ❑ Consult with the end-user, regarding finalisation of the description of requirements (Specification) for inclusion in the document.
- ❑ Complete the Tender Data Sheet in section II with details of the tendering process e.g. the tender closing date, the address for submission, the evaluation criteria to be applied, any qualification criteria to be applied etc. The text of the Instructions to Tenderers should not be modified.
- ❑ Complete the Conditions of Particular Application in section VIII with the conditions which will apply to the contract e.g. payment terms, delivery/completion period, warranty, liquidated damages. Note that the Conditions of Particular Application modify the General Conditions of Contract – the text of the General Conditions of Contract must not be modified.
- ❑ Complete the Bill of Quantities section by describing the required unit quantities etc.

The Sample Forms provide text and format samples for the various forms which are to be completed by the tenderer.

6.5.3 Preparation of Request for Quotation

A Standard Request for Quotations is required for the Request for Quotations method only.

The Standard Request for Quotations is to inform tenderers of:

- the precise description of the Works required;
- the rules for the procurement process;
- the evaluation criteria and methodology which will be applied;

- any qualification criteria which will be applied; and
- the type and conditions of the proposed contract (purchase order).

The Head of Procurement Unit is responsible for drafting the Request for Quotations

Actions

The Head of Procurement Unit should:

- Consult with the end-user, and technical specialists if required, regarding finalisation of the description for inclusion in the document
- Complete details of the procurement process e.g. the tender closing date, the address for submission, the evaluation criteria to be applied, any qualification criteria to be applied etc.
- Complete the Conditions of Contract section with the conditions which will apply to the contract (purchase order) e.g. payment terms, delivery/completion period, warranty, liquidated damages

5.5.4 Invitation Advertisements for Competitive Tenders (ICT and NCT)

Publication of the advertisement must allow sufficient time before the deadline (or closing day and time of the tender) to enable prospective tenderers to obtain the tender documents and to prepare and submit their tenders. The period allowed should be a minimum of 6 weeks for ICT, and 2 weeks for NCT following the date of the first advertisement, but longer periods may be necessary for more complex requirements.

Procurement Entities must be careful to also allow for the time between submission of the advertisement to the press and the time the advertisements are actually published.

Advertisements for National Competitive Tendering shall be published:

- in the Public Procurement Bulletin and the PPB website; and
- in at least in two local daily newspaper of general circulation in Ghana,

In the case of International Competitive Tendering, publication in international newspapers or journals is also required. However, copies of the Invitation to Tender submitted to all foreign Embassies and High Commissions in Ghana shall be deemed to have met this requirement.

5.5.5 Contents of the Advertisement

The information contained in the advertisement must correspond to the information in the Tender Document and in particular, the relevant information in the Tender Data Sheet. The advertisement should include as minimum, brief information on:

- the source of funding;
- the works to be procured;
- any qualification requirements for Tenderers (e.g. a requirement for a minimum level of relevant experience or turnover);

- the procedure for obtaining the Tender Document;
- the place and deadline for submission of tenders;
- the fee to be paid for the Tender Documents. This must reflect only the cost of printing and delivery to prospective Tenderers;
- the value of any Tender Security required.

5.6 SALE OF TENDER DOCUMENTS FOR COMPETITIVE TENDERS

The sale of tender documents for competitive tenders will be co-ordinated by the Procurement Unit or the Technical Department as appropriate. The price charged for the documents must reflect only the cost of printing and provision of the tender documents.

The Procurement Entity must ensure that tender documents are available for sale before publication of the invitation. If the stock of tender documents is exhausted, immediate action to print additional copies shall be initiated. It is an offence to deny a prospective tenderer the opportunity to purchase a tender document, and failure to provide a tender document for inspection or purchase by a potential tenderer may constitute grounds for complaint under Part VII of the Act.

Action:

- Prospective tenderers should be allowed to briefly inspect the Tender Document, if requested, before committing to purchase of the document.
- Record the name and address of each purchaser of the Tender Document in a register.
- Fees received from the sale of Tender Documents must be recorded and the income deposited in the Consolidated Fund. A formal Receipt must be issued to the purchaser.

5.7 ISSUE OF INVITATIONS FOR RESTRICTED TENDER

The Standard Tender Document for a Restricted Tender must be issued to all short-listed contractors simultaneously, either by registered post, or by hand in return for a written receipt.

Action:

The Head of Procurement Unit should:

- Prepare the Tender Document together with a personalised Letter of Invitation for each short-listed contractor.
- Send the invitation by registered post to all short-listed contractors, or deliver by hand and obtain a written receipt.

5.8 ISSUE OF REQUESTS FOR QUOTATIONS

The procedure for issue of Requests for Quotations to short-listed contractors follows the same principles as for Restricted Tender above.

The Standard Request for Quotations will be used for simple requirements wherever practical. For more complex requirements when the normal SRFQ and Purchase Order documents are not appropriate nor provide an appropriate

form of contract, the Procurement Entity may utilise appropriate formats from the other Standard Tender Documents.

5.8.1 Opening of Standard Requests for Quotations

There shall be public opening of the quotations submitted. All quotations must be opened on a predetermined date and time by the Procurement Unit to avoid the opportunity for any Contractor to become aware of the prices quoted by other contractors.

The recording of quotations and the evaluation procedures followed are similar to those for Tenders except for the following:

- Quotations will normally be submitted using the appropriate Standard Request for Quotations document;
- Sealed quotations will be received directly by the Procurement Unit rather than being placed in the tender box;
- The Procurement Unit will register the receipt of each quotation and keep the envelopes secure and unopened until the date of the opening;
- Contractors will not automatically be invited to attend the opening of quotations, but the basic procedures and formality of the Meeting must be maintained.

Action:

The Tender Opening Committee should:

- Ensure that the name, organisation represented and contact details of all attendees are recorded in an attendance register.
- Ensure the security of documents at all times during the procedure to prevent any unauthorised interference with the documents.
- Bring in the unopened quotations and any samples received and check that the writing on each envelope confirms that it is for the correct RFQ.
- Open the first quotation after confirming that any conditions regarding marking and sealing of the envelope have been met and the envelope has not been tampered with. Note each quotation with a serial number in sequence as it is opened (e.g. “1 of 4” where the total number of RFQs is 4).
- Examine the contents of the envelope and identify, stamp and number all originals and copies, and any separate sections and attachments.
- Read out the following details from each quotation:
    - the number allocated to the quotation by the Tender Opening Committee;
    - name of the Contractor;
    - brief description of the works offered if the RFQ is for more than one Lot;
    - the total price quoted; and
    - any other appropriate information at the discretion of the Chairman.

- Any obvious failure to provide a responsive quotation shall be noted in the Minutes. These quotations shall normally be rejected.
- The Chairperson and two Members of the Committee shall initial quotation and all attachments thereto. Any corrections to prices or obvious errors and omissions shall be circled in read ink and also initialled.
- Record the details read out of each quotation in the register of RFQ Opening ensuring that amounts are recorded in words as well as figures, and record all corrections and errors or omissions which are noted in the Minutes of the Meeting.
- Minutes of the RFQ Opening shall be prepared by the Procurement Unit.

5.8.2 Evaluation of Contract for RFQ

Evaluation of quotations shall be undertaken by the Procurement Unit.

Action:

- Rank all quotations that are responsive to the Request for Quotations according to price and select the lowest evaluated priced RFQ for contract award.
- Prepare a brief report of the evaluation for the procurement record.
- Prepare a Purchase Order for signature by the Head of Procurement Entity.
- Issue the Purchase Order to the selected Contractor.

5.9 TENDER CLARIFICATIONS

The period during which tender clarifications may be requested will be defined in the Tender Document. Requests for clarifications received after this period may be ignored unless they draw attention to a serious flaw in the Tender Document. In all cases, any response to a Tenderer must be communicated to all tenderers who received the Tender Document, but without identifying the tenderer who requested the clarification.

Action:

The Head of Procurement Unit should;

- Record each request for clarification and immediately develop a response in consultation with the appropriate Technical Department.
- The Procurement Unit should also consider if the proposed response to a request for clarification will require tenderers to undertake additional work, or make substantial modifications to their Tenders, which cannot reasonably be completed by the stated date for submission of Tenders. In this case, the Tender Committee should be consulted for approval to extend the date for submission of Tenders.
- Issue a written response to the tenderer and notify details of the query and the response (but without identifying the source of the query) to all other tenderers simultaneously.
- Place copies of all correspondence on tender clarifications in the Procurement record file.

5.10 EXTENSION OF TENDER CLOSING DATE

The closing date for submission of Tenders may be extended at the discretion of the Procurement Entity for practical or justifiable reasons. The reasons may include modification to the Tender Document after issue and requests for an extension of time by tenderers.

Action:

The Head of Procurement Unit should:

- ❑ Ensure that there is an adequate practical justification for extending the closing date. Extensions should not be granted, for example, where a tenderer has by his own inactivity failed to purchase the Tender Document early enough to permit submission of a responsive tender.
- ❑ Issue an addendum notifying all Tenderers of the revised date for submission of Tenders.
- ❑ Place copies of all relevant correspondence in the Procurement record file.

5.11 REJECTION OF TENDER BEFORE TENDER OPENING

A Tender process may be cancelled at any time before the deadline for receipt of Tenders with the approval of the Entity Tender Committee. The grounds for the cancellation must have been stated in the Tender Documents.

Justifications for cancellation of tenders are:

- ❑ the need for the works has ceased to exist or changed significantly;
- ❑ insufficient funding for the works;
- ❑ significant change in the required technical details, tender conditions, conditions of contract or other details, such that the re-commencement of proceedings is necessary;
- ❑ insufficient, or no tenders are received;
- ❑ there is evidence of collusion among Tenderers; or
- ❑ cancellation is deemed to be in the interest of national security.

Action:

The head of Procurement unit should:

- ❑ Notify all tenderers who have purchased or received the Tender Document of the cancellation, and refund any fees paid for the purchase of Tender Document.
- ❑ Return any tenders submitted to the tenderer unopened.
- ❑ Notify all Departments, Units and Projects involved in the works of the cancellation of tender proceedings.

5.12 RECEIPT OF TENDERS

All Tenders shall be deposited in the designated locked tender box (or such other arrangements as stated in the Tender Document) until the Tender Opening. The Procurement Unit will co-ordinate the Tender opening proceedings.

The Tender Document will have provided clear instructions to tenderers on the marking and sealing of Tenders and the procedures to be followed for submission.

The closing date and time for submission of Tenders must be determined in advance and stated in the Tender Document.

Action:

The head of Procurement Unit should:

- Arrange placement of the tender box in a prominent place and ensure that all reception office staff and staff responsible for the receipt of tenders are fully aware of the Tender and their responsibilities for Tenders received.
- Ensure that the tender box is kept locked until the Tender Opening and that neither the tenderers nor staffs have an opportunity to tamper with the box.
- Register all tenders received by post or by courier service on receipt, stamp with the date and time of receipt, and deposit the envelope unopened in the correct tender box.
- Direct all tenderers' representatives delivering Tenders by hand to the tender box so that they may place their envelopes directly in the tender box.
- Ensure that Tenders received which are too large to fit in the tender box are registered and securely retained in a locked room under the control of the Procurement Unit until the Tender Opening.
- Close and seal the tender box immediately following the deadline for submission of Tenders and transport the tender box to the room where the Tender Opening will take place.
- If a single tender box is used for multiple Tender procedures, or the tender box is a fixture, open the tender box immediately following the deadline for submission of Tenders and remove all Tenders required for the Tender to be opened, and transport these to the room where the Tender Opening will be held.
- Ensure that Tenders which were too large to fit in the tender box are also transported to the room where the Tender Opening will be held immediately following the deadline for submission of Tenders.

5.13 OPENING OF TENDERS

Tender Opening shall commence immediately after the close of the tenders (as stated in the tender document)

The Procurement Unit will co-ordinate the Tender Opening, ensure smooth operation of the proceedings, take a register of attendance, prepare Minutes of the opening, and advise the Chairperson of the opening session on procedural issues if requested.

A Tender Opening Panel shall comprise at least 3 persons, including a member of the Entity Tender Committee. They shall ensure that minutes of the tender opening proceedings are duly written.

The Chairman of the Tender Opening Panel will control and direct the Tender Opening and not allow tenderer's representatives to interfere with the work of the Panel. Any objections by a tenderer to the procedures or decisions of the Tender Opening should be made in writing to the Head of the Procurement Entity.

For purposes of transparency it is not permitted for a tender opening to be halted or postponed once the process begins.

Action:

The chairperson of the Tender Opening Panel should:

- ❑ Open the meeting and outline the procedures to be used for the Tender Opening.
- ❑ Ensure that tenderers representatives are seated separately from the Tender Opening Panel and officials of the Procurement Entity, and that the name, organisation represented and contact details of all attendees are recorded in an attendance register.
- ❑ Ensure the security of documents and Tenders at all times during the Tender Opening procedure to prevent any unauthorised interference with the documents and process.
- ❑ Bring in the unopened tender box and/or all Tenders and samples received which have been removed from the tender box or could not be accommodated in the tender box.
- ❑ Open the tender box, check that the inscription on each envelope or sample inside confirms that it matches the correct Tender and complies with the wording and sealing required in the Tender documents. Stack all envelopes in clear view of the tenderers ready for opening.
- ❑ Any Tenders not received by the deadline for the opening of Tenders must be rejected and returned unopened to the tenderer. Tenderers are not permitted to amend their Tender in any way during the Tender Opening or to submit any additional documents during the process.
- ❑ Check for any withdrawals or modifications submitted, and match these with the original Tender before proceeding. Withdrawn Tenders shall not be opened once the authenticity of the withdrawal notice has been confirmed.
- ❑ Open the first Tender after confirming that all conditions regarding marking and sealing of the envelope have been met and the envelope has not been tampered with. Stamp each copy of the Tender and annotate each Tender with a sequential serial number as it is opened (e.g. "1 of 7" where the total number of Tenders is 7).
- ❑ Examine the contents of the envelope and identify, stamp and number all originals and copies, and any separate sections and attachments.
- ❑ Read out the following details of each Tender from the Original copy:
  - o any Tender modifications or withdrawals;
  - o the number allocated to the Tender by the Tender Opening Panel;
  - o the name and country of the Tenderer;

  - a brief description of the works offered if the Tender is for more than one Lot;
  - the currency of the Tender;
  - the total Tender price;
  - any discounts offered;
  - the presence or absence of any required Tender Security; and
  - any other appropriate information at the discretion of the Chairman.
- Pass all Tender Securities to the representative of the Procurement Unit for review, registration and safekeeping.
- Any obvious failure to provide a responsive Tender, such as the absence of Tender Security or inadequate Tender Security, shall be reported to the Meeting and recorded in the Minutes.
- The Chairperson and two Members of the Panel shall initial the original of each Tender and all attachments thereto. Any corrections to prices or obvious errors and omissions shall be circled in red ink and also initialled.
- Record the details read out of each Tender in the Register of Tender Opening, ensuring that amounts are recorded in words as well as figures, and record all corrections and errors or omissions which were noted during the Tender Opening and captured in the Minutes of the Tender Opening.
- Any envelopes containing substitutions, or modifications, must be subject to the same level of scrutiny, including the reading out of critical details, such as price changes.
- Minutes of the Tender Opening shall be prepared by the Procurement Unit, signed by the Chairman of the Tender Opening Panel and made available to any Tenderer involved in the Tender who requests a copy in writing.

5.14 TENDER EVALUATION

Tender evaluation will be conducted by a Tender Evaluation Panel consisting of a minimum of three qualified members.

It should be noted that tenderers frequently attempt to contact the Procurement Entity during Tender evaluation, directly or indirectly, to ascertain progress of evaluation, to offer unsolicited clarifications, or to provide criticisms of their competition. The evaluation Panel must evaluate Tenders solely on the basis of the information provided in the respective Tenders and no changes in the Tender price or substance of a Tender will be permitted. No circumstances shall justify meetings or consultation between the Procurement Entity (or its consultants) and tenderers during the Tender evaluation process.

5.14.1 Preliminary Examination of Tenders

The procedures for examination of tenders and clarifications must be conducted in accordance with the specific terms contained in the Tender Document used.

Action:

The Evaluation panel shall:

- ❑ Conduct a preliminary examination to determine whether tenders are complete and are responsive to the basic instructions and requirements of the Tender Document including checks that:
- ❑ the Tenderer is eligible to tender where this has not been determined prior to inviting tenders;
- ❑ all Tenderers are from eligible source countries;
- ❑ the Tender has been submitted in the correct format and there are no missing pages;
- ❑ any erasures, interlineations, additions or other changes in the Tender have been initialled by the Tenderer;
- ❑ any required Tender Security has been submitted, in the correct form and amount and valid for at least the period required;
- ❑ the Tender has been submitted without material reservations or deviations from the terms and conditions of the Tender Document;
- ❑ the Tender has been correctly signed and authorised;
- ❑ if the Tenderer is a joint venture, a copy of the joint venture agreement has been submitted;
- ❑ the correct number of copies of the Tender have been submitted;
- ❑ the Tender is valid for at least the period required;
- ❑ all key documents and information have been submitted; and
- ❑ the Tender meets any other key requirements of the Tender Document.

If a prequalification procedure was applied, reject and exclude any Tender received from other than the pre-qualified tenderers.

- ❑ May request clarifications from tenderers concerning ambiguities or inconsistencies in the Tender. As required in the Tender Document, such requests shall be in writing, and no change in the price or scope of the originally offered works may be sought or accepted, except for the correction of arithmetic errors. The responses from Tenderers shall also be in writing.
- ❑ Notify tenderers of any such arithmetic corrections and request written, agreement of the Tenderer to the correction. If a Tenderer does not accept the correction of an arithmetical error, his Tender must be rejected and the Tender Security may be forfeited.

Any communications between the Procurement Entity and a tenderer during the preliminary examination of Tenders shall be made in writing.

5.14.2 Responsiveness of Tenders

Following the preliminary examination of tenders, the Tender Evaluation Panel should examine the tenders to determine whether any issues arising from the preliminary examination affect the responsiveness of an individual tender, and whether each tender is substantially responsive to the technical specifications and contract conditions stated in the Tender Document.

- The determination of a Tender's responsiveness is based on the contents of the Tender itself, subject to any clarifications received in the Preliminary Examination of Tenders.
- A substantially responsive Tender is one that conforms to all the instructions, requirements, terms and conditions of the tender documents, without material deviation, reservation or omission. A material deviation, reservation, or omission is one that:
  - affects in any substantial way the scope, quality, or performance of the works specified in the Tender Document; or
  - would limit in any substantial way, inconsistent with the Tender Documents, the rights of the Procurement Entity or the Tenderer's obligations under any resulting contract; or
  - if corrected would unfairly affect the competitive position of other Tenderers presenting substantially responsive and compliant Tenders.

Any tender containing a material deviation, reservation or omission, is therefore not substantially responsive, shall be rejected and may not subsequently be made responsive by the Tenderer or the Procurement Entity.

The classification of a deviation, reservation or omission as material or non-material shall be determined by the objectives and requirements of the individual procurement requirement, as stated in the Tender Document, and shall take into account the impact on key factors, such as cost, risk, time and quality.

Material deviations, reservations or omissions may typically include:

- unacceptable time schedules for completion of the works compared with requirements stated in the Tender Document;
- unacceptable alternative technical details, such as inferior design, materials, workmanship, specifications, standards or methodologies; or
- unacceptable counterproposals with respect to key contract terms and conditions, such as payment terms, price adjustment, liquidated damages, sub-contracting or warranty.

Non-material deviations may include:

- longer completion schedules unless this takes completion of the works past a clearly defined cut-off date stated in the Tender Document;
- minor deviations in the payment terms required;
- tenders offering goods to alternative, but equal or superior specifications and performance;
- tenders offering the specified works but failing to price specific items within the bill of quantities or activity schedule.

The classification of deviations, reservations and omissions as material or non-material must be consistently applied to all Tenders.

Where a Tender is determined to be substantially responsive, the Evaluation Panel may waive, clarify or correct any non-conformity, error or omission, which does not constitute a material deviation. Such non-conformity, error or

omission should be quantified in monetary terms to the extent possible and taken into account in the financial evaluation and comparison of Tenders.

Action:

The Evaluation panel may;

- Reject and exclude from further evaluation any Tender that is incomplete, unsigned, not accompanied by a Tender Security, not accompanied by essential supporting documents, or not substantially responsive to the technical specifications, contract conditions or other critical requirements stated in the Tender Document.
- Reject and exclude from further evaluation any Tender which contains material deviations, reservations or omissions, and is therefore not substantially responsive.

5.14.3 Financial Evaluation of Tenders

Tenders that are determined to be substantially responsive to the requirements of the Tender Document shall be subjected to financial evaluation to determine the evaluated price of each Tender, and the lowest evaluated tender.

The evaluated price for each Tender is determined by:

- taking the Tender price, as read out at the tender opening;
- Correct any purely arithmetical errors in tenders in accordance with the procedure stated in the Tender Document. Note that if there is any discrepancy between numbers and written figures, the written figures shall prevail. If both the quantity and the total price are indicated in the Tender form, arithmetic should be checked and if there is a discrepancy, the unit price shall govern. All errors in extension (multiplying the unit price by the quantity) and totalling should be corrected and the tenderer notified. A tenderer cannot be permitted to retain an arithmetical error in extension or totalling and correct the unit price
- applying any non-conditional discounts offered in the Tender;
- making financial adjustments in accordance with the Tender Document for any non-material non-conformity, error or omission. Pricing of works items or features which are clearly omitted in a Tender may be based on the highest prices for corresponding items in other responsive Tenders. Note that by convention, unpriced sub-items in a bill of quantities are normally assumed to be covered in the price of the main item and no adjustment to the evaluated tender price is made in this case. Note also that no additional credits for may be given for offered features that exceed the required standards or specifications;
- converting all Tenders to a single currency, using the currency and the source and date of exchange rate indicated in the Tender Document; and
- applying any margin of preference indicated in the Tender Document.

Tenders shall be compared by ranking them according to their evaluated price to determining the Tender with the lowest evaluated price.

Treatment of Discounts

Tenderers may be permitted to offer discounts to their Tenders, which shall be included in the Tender and read out at the tender opening.

Any discount offered after the deadline for submission of Tenders must not be taken into account.

Any non-conditional discounts shall be taken into account in the financial evaluation and comparison of tenders.

Any prompt payment discount included in a Tender shall become a term of the contract, if that Tender is accepted, and utilised by the Procurement Entity, if payment is made in accordance with the terms of the discount. However, prompt payment discounts must not be considered in the evaluation and comparison of tenders.

Post-Qualification of the lowest evaluated responsive Tenderer should be conducted to determine the Tenderer's physical capability to perform the contract. Using the criteria specified in the Tender Document, this review should include an assessment of the Tenderer's technical, financial and physical resources available to undertake the contract, including his current workload.

- ❑ If pre-qualification was conducted, the lowest evaluated responsive Tender should be recommended for the award of contract, unless the Tenderer's qualifications have since materially deteriorated, or if the Tenderer has since received additional work that reduces the available capacity.
- ❑ Where pre-qualification has not taken place, the lowest evaluated responsive Tender should be subjected to post-qualification, according to the procedures described in the Tender Document.
- ❑ If the lowest evaluated responsive Tenderer fails post-qualification, his Tender should be rejected, and the next ranked Tenderer should then be subjected to post-qualification examination. If successful, this Tenderer should receive the award. If not, the process continues for the other Tenderers.
- ❑ The rejection of a Tender for reasons of qualification requires substantial justification, which should be clearly documented in the attachments to the Evaluation Report.
- ❑ A history of poor performance may be considered a justification for failing post-qualification if the Tenderer is unable to demonstrate that steps have been taken to resolve previous problems.

5.14.4 Evaluation Reports and Recommendations

The Tender Evaluation Panel will prepare an evaluation report for submission to the Tender Committee.

The evaluation report should be prepared using the standard format for the Evaluation of Works, and include:

- o a summary of the Tenders received and opened;
- o the results of the preliminary examination;

- the results of the technical evaluation;
- reasons why any Tenders were declared non-responsive. (Attach copies of selected pages from Tenders if necessary, to show examples of objectionable features);
- details of any non-material deviations, which were accepted and the way in which they were quantified and taken into account in the financial evaluation;
- the evaluated price of each Tender, showing any corrections or adjustments to the tender price and any conversion to a common currency;
- An explanation of:
- any inconsistencies between prices and modifications to prices read out at Tender opening.
- any substantial corrections for arithmetic errors which may affect the ranking of Tenderers.
- any additions, adjustments, and priced deviations that may affect the ranking of Tenderers.
- any cross-discounts not read out and recorded at the Tender opening.
- the ranking of the Tenders, according to their total evaluated price;
- a statement of the lowest evaluated substantially responsive tender, for each lot where applicable, clearly establishing the actual amount of the contract award;
- confirmation that the total price quoted by the lowest evaluated responsive Tender is acceptable compared with the original estimated cost of the procurement;
- the results of any post-qualification examination; and
- a recommendation to award the contract or contracts to the lowest evaluated responsive Tenderer or combination of Tenderers, or other appropriate recommendation, such as the cancellation of the procurement process.

The Evaluation Panel may recommend that all of the Tenders be rejected if:

- none of the Tenders are found to be responsive;
- all of the Tenders are unreasonably high in price compared to the cost estimate;
- none of the Tenderers are qualified;
- there is deemed to be an absence of competition, (however lack of competition is not usually decided solely on the basis of the number of Tenderers).

Attach to the evaluation report:

- copies of any correspondence with Tenderers who raised objections to the Tender or evaluation process, together with detailed responses;

- copies of any letters from Tenderers requesting clarifications, and responses by the Procurement Entity, and Tender clarifications requested by the Procurement Entity and Tenderer responses;
- any separate evaluation report from a consultant, if one was engaged for this purpose;
- all required Tables and supporting documents.

The Evaluation Panel should ensure that the Tender evaluation report is double-checked, signed and complete before submitting for approval by the Tender Committee.

The evaluation report forms a part of the record of procurement proceedings required under Section 28 of the Act.

5.15 REVIEW AND APPROVAL OF EVALUATION REPORT

The appropriate review authority (Head of Procurement Entity, Tender Committee, District, Regional, Ministry or Central Tender Review Board) will review the evaluation report and recommendations presented in the Evaluation Report.

The review authority, before giving approval, will ensure that any written complaints made by Tenderers concerning the tender process have been addressed and responded to. If the complaint reveals a serious breach of procedures or ethics, the review authority may recommend rejection of the evaluation report and call for re-tendering.

Action:

The Review Body will:

- ❑ check the accuracy of the evaluation and recommendations as submitted;
- ❑ decide whether the price increase is justified if the price of the lowest evaluated responsive Tender is higher than the original estimated cost. If the price is not acceptable, recommend that the Tender should be re-advertised, cancelled, or the scope of works reduced;
- ❑ reject any evaluation report which contains serious inaccuracies or flaws and return to the Tender Evaluation Panel with written comments for correction and re-submission;
- ❑ ensure that all discussions and decisions of the review authority are reported in the minutes of the meeting.

5.16 EXTENSION OF TENDER VALIDITY

The duration of tender validity is specified in the Tender Document and should be confirmed in the signed Tender Form submitted by each Tenderer. If circumstances occur in which award cannot be made within the original tender validity period, extensions in writing should be requested from Tenderers, in accordance with the Tender Document.

The evaluation and award of contract should be completed within the period set for the validity of tenders. The date for expiry of tender validity must be monitored and attention drawn to this deadline not less than two weeks before the expiry date.

If, due to unforeseen circumstances, the task cannot be completed within the set period, the Procurement Unit may contact tenderers to seek their agreement to an extension of the tender validity.

Tenderers who refuse this request may withdraw from the Tender without incurring any penalty, but tenderers who agree to an extension will also be required to extend their Tender Securities for an appropriate period.

When an extension of tender validity period is requested, tenderers shall not normally be requested or be permitted to change the quoted price or other conditions of their tenders. However, the Tender Document may provide for an appropriate price adjustment mechanism when requests for second or subsequent extensions are made, to reflect changes in the cost of inputs for the contract over the period of extension.

Action by Procurement Unit:

- ❑ Maintain a diary reminder of all tender validity dates and advise the responsible officer not less than two weeks before the expiry date so that appropriate action can be taken in time.
- ❑ Determine the additional time required to complete the evaluation and award of the specific contract. Any extension of tender validity should be for the minimum period required to complete the evaluation, to obtain the necessary approvals and to award the contract.
- ❑ Prepare an individual letter to each tenderer requesting their formal approval to an extension of the validity of tenders and an extension of their Tender Securities (where necessary) for the required period. Set a date for written responses to be received by the Procurement Entity, stressing that tenderers who do not respond in time will be considered to have withdrawn their tenders.
- ❑ Send the letter by registered post or deliver by hand to each tenderer and obtain a receipt.

Tenderers have the right to refuse to grant an extension of tender validity without forfeiting their Tender Security. If a tenderer refuses to extend the validity of his tender then, upon expiry of the original tender validity period, the Tender Security shall be returned and the Tender not considered further.

Only tenderers who respond confirming their unconditional acceptance and enclosing any required extension to their Tender Security may be considered for further evaluation and award of contract.

5.17 AWARD OF CONTRACT

Following approval from the relevant review body, the contract will be awarded to the Tenderer who has submitted the lowest evaluated Tender.

Before contacting the Tenderer, a formal commitment of the required funds against the budget of the Procurement Entity must be approved.

Action:

- ❑ Prepare/update the Activity and Expenditure Initiation (A&EI) Form and obtain approval of the commitment of funds against the budget of the Procurement Entity.

- ❑ Prepare four copies (minimum) of the contract ready for signature by each party to the contract, and include all specific details relating to the Tenderer, the Conditions of Contract, and the Tenderer's offer. A clear statement on when the contract becomes effective is also essential since certain contract clauses could be connected with this date, e.g. Mobilisation, Site Possession, Validity of Performance Security, etc.
- ❑ Obtain the signature of the Head of Procurement Entity on all copies of the contract.
- ❑ Provision of Performance Security should be a pre requisite for contract signature.

Note that the four copies (minimum) of the Contract will be distributed after signature as follows:

- o Original – Procurement Unit;
- o 1st Duplicate – Supplier/Contractor;
- o 2nd Duplicate – the Technical Department concerned; and
- o 3rd Duplicate – Legal Officer/Ministry of Justice.

- ❑ Ensure that the contract is duly recorded in the Contracts Register maintained by the Procurement Unit.
- ❑ Arrange the immediate return of Tender Securities to all tenderers and the successful Supplier or Contractor as soon as the Contract becomes effective.

5.17.1 Award Notification

Notice of the tender award shall be issued promptly to the successful Tenderer. The successful Tenderer shall be required to confirm in writing acceptance of the tender award and submit the appropriate Performance Security (if required).

The Tenderer shall be invited to attend for contract signature, or where this is not practical, provided with copies of the Contract for signature and return of signed copy of the Contract to the Procurement Entity.

Failure of the Tenderer to confirm acceptance of the award, or to submit the Performance Security, or to sign the contract may constitute grounds for the annulment of the award and forfeiture of the Tenderer's Tender Security. In that event, the Procurement Entity may award the contract to the next lowest evaluated Tenderer, whose offer is substantially responsive and is determined to be qualified to perform the contract satisfactorily.

Procurement Entities are required to submit notice of contract awards to the Public Procurement Board within 30 days of contract signature, for publication on the PPB website and/or in the Procurement Bulletin. The information on contracts awarded/signed shall include the:

- ❑ name of the Contractor;
- ❑ description of the works;
- ❑ Contract Sum;
- ❑ duration of the contract;

- ❑ method of procurement used; and
- ❑ the source of funding.

5.17.2 Notification to Unsuccessful Tenderers

All unsuccessful Tenderers should be notified immediately once the contract has been awarded. Tender securities of unsuccessful Tenderers should be promptly returned after awards have been made.

5.17.3 Return of Tender Securities

All Tender Securities must be returned immediately to the Tenderer, as soon as they are no longer required, i.e. once the relevant contract is signed and all conditions for contract effectiveness have been met.

5.18 CONTRACT MANAGEMENT

Effective management of contracts is essential to ensure that the objectives of the procurement process are achieved and that all contractual obligations and activities are completed efficiently by both parties to the contract. The Procurement Unit or the Technical Department concerned must ensure that routine monitoring of all current contracts is maintained so that swift remedial measures can be taken when problems arise, or preventative action taken when problems are foreseen.

There are many post-contract issues that need to be dealt with, monitored and resolved before the contract reaches its conclusion including:

- o Contract Effectiveness;
- o Appointment of a Project Manager ;
- o Valuation of work done
- o Preparation of Interim and Final Payment Certificates;
- o Deduction of Retentions and payments to the Contractor;
- o Contractual Disputes;
- o Delays in Performance;
- o Claims for Damages;
- o Insurance Claims
- o Initial and Final Acceptance of the works;
- o Release of Performance Securities and Retentions;
- o Contract Closure.

5.18.1 Contract Effectiveness

Although the Contract may have been signed by both parties, the legal effectiveness of the Contract may be dependent on one or more of the following conditions:

- o Receipt by the Procurement Entity of an Advance Payment Security;
- o Receipt by the Supplier of the Advance Payment;
- o Receipt by the Supplier of an acceptable Letter of Credit; or

- o Mobilisation and Site possession by the Contractor.

Actions:

The Head of Procurement should ensure that:

- ❑ any Performance Security specified in the contract is received by the Procurement Entity;
- ❑ any Advance Payment specified in the contract is paid immediately when the Advance Payment Security is received from the Supplier or Contractor;
- ❑ any Letter of Credit required is issued promptly
- ❑ the Contractor meets the agreed dates for mobilisation and possession of the site.

5.19 CONTRACT SUPERVISION AND ADMINISTRATION

Contract supervision and administration will be undertaken by the Project Manager in consultation with the Procurement Unit and the Technical Department.

Supervision and administration of works contracts is often a complex task relying heavily on the experience and qualifications of the Project Manager.

Action:

The Project Manager/Supervision should:

- ❑ Maintain close supervision of the Contractor's performance, work done, materials used, equipment and labour force at site to ensure that potential problems are identified as early as possible.
- ❑ Notify the Contractor in writing requesting rectification of any deficiencies in workmanship, materials used, safety or environmental standards, or other required performance standards
- ❑ Hold regular site meetings with the Contractor to identify the causes of any slippage in the schedule of works.
- ❑ Receive regular progress reports from the Contractor and ensure that written records of any disputes or contract variation orders issued are maintained.
- ❑ Ensure that any significant problems, variation orders, day work claims, compensation events, cost overruns, or slippage in the timetable are brought to the attention of the Procurement Entity.
- ❑ Initiate and supervise any process for claims against insurance or the Contractor.
- ❑ Conduct detailed checks on the Contractors valuation of work performed, remeasure as appropriate, and prepare Interim Payment Certificates, deducting any retention percentage specified in the Contract.
- ❑ Participate in inspections for Interim and Final Handover of the Works and prepare the Final Payment Certificate releasing retention money to the Contractor

5.20 INITIAL AND FINAL TAKEOVER OF WORKS

Initial and final takeover of works shall be certified by an Inspection and Acceptance team comprised of the Project Manager, and a representative from each of the Procurement Unit and the Technical Department.

- ❑ On issue of an initial takeover certificate by the Inspection and Acceptance Committee, part of any retention monies held may be paid to the contractor if specified in the contract.
- ❑ Following the end of the defects liability period and subject to all defects being rectified, , the Inspection and Acceptance team for will certify the final takeover of the Works and sign the Certificate of Completion.
- ❑ On issue of the final takeover certificate by the Inspection and Acceptance team, a Final Certificate shall be raised to authorise payment of the balance of any retention monies due to the contractor.

5.20.1 Defects Liability Period

During the defect liability period, the Contractor is obliged to repair/replace any defects at his/her own cost. However, if the defect is proven to be as a result of a design problem, whereby the Contractor was not involved in the design, then the Procurement Entity will bear the costs of rectification.

5.21 PAYMENT FOR WORKS

For Payment Certificates raised by the Project Manager, the Procurement Entity will:

- ❑ Complete a Payment Certificate ensuring the deduction of any advance payments already made, retention percentages, and any contractual penalties incurred by the Contractor.
- ❑ Attach the:
  - o original valuation from the Contractor and the Payment Certificate issued by the Project Manager;
  - o original of any Interim or Final Inspection Report;
  - o results of any analytical surveys;
  - o calculation of any penalties liquidated damages if allowable under the contract;
  - o copies of relevant information from the contract document, records of approval and financial authorisations.
- ❑ Record the payment in the Contract Register.
- ❑ Forward the Payment Voucher to the Accounts Department for processing.

5.22 DELAYS IN PERFORMANCE

Performance of the works should be completed by the Contractor in accordance with the time schedule prescribed in the Schedule of Requirements. Where this is not the case:

- The Contractor must notify the Procurement Entity, through the Project Manager, in writing of the conditions delaying performance, including full details of the delay, the likely duration and the cause(s).
- The Procurement Entity will immediately assess the situation in consultation with the Project Manager, and may at its discretion extend the Contractor's time for performance, with or without liquidated damages as specified in the Contract.
- If the time for performance is extended, both parties shall ratify such extension by a formal addendum to the Contract subject to approval by the Tender Committee.
- A delay by the Contractor in the performance of his obligations may render him liable to liquidated damages if specified in the contract document, except where:
  - the delay is as a result of Force Majeure;
  - there is no provision for liquidated damages in the contract;
  - an extension of time is agreed between the two parties without the application of liquidated damages.

Actions:

The Project Manager should:

- Refer to the relevant clauses in the Conditions of Contract for the procedure to be followed to apply, calculate and claim liquidated damages.
- Update the Procurement file and Contract Register to reflect any delays in the Contractor's performance.
- Ensure that the end-user department is notified immediately of all such delays.

5.23 RESOLUTION OF CONTRACTUAL DISPUTES

Most minor disputes may be resolved by discussion and agreement between the Project Manager and the Contractor to rectify the cause of complaint.

Any formal written complaints received from a Contractor should be fully investigated and referred to the Head of the Procurement Entity to authorise correspondence or formal negotiations with the Contractor.

Where an adjudicator is appointed under the contract, such adjudication may result in an unfavourable decision in favour of the Contractor, and should therefore be treated as a last resort once all other possibilities for agreement are exhausted

Action:

The Project Manager should:

- Examine the Contract carefully to be aware of all contract conditions relating to the Resolution of Disputes.

- Determine if the Procurement Entity is at fault or partly at fault, and if so, take appropriate action to rectify the problem.
- Invite the Contractor to a formal meeting, within 7 days of the complaint, to discuss the issues and try to agree a compromise acceptable to both parties. Ensure that accurate written Minutes are kept of any such meeting. If an agreement is reached which changes any of the conditions of the Contract, approval of the Entity Tender Committee or the Head of the Procurement Entity is required before the agreement can be implemented.
- If no initial agreement is reached and negotiations conducted by the Head of the Procurement Entity also fail, consider the use of any adjudication or arbitration services as specified in the contract.
- Prepare any necessary addendum to the Contract for signature.

5.24 TERMINATION OF THE CONTRACT

The parties to the contract normally have the right to terminate the contract, but to protect the Procurement Entity, advice of the Legal Department should be sought. Contracts should not be terminated without examining all possible alternatives, unless the termination is mutually agreed by all parties to the contract.

Action:

The Procurement Entity should:

- Examine the Contract carefully to be aware of all contract conditions and penalties relating to the Termination of Contract.
- Follow the advice of the Legal Officer/ Ministry of Justice in the preparation of any correspondence and settlement of any contractual penalties.

5.25 CONTRACT AMENDMENT

Contract amendment may become necessary as a result of the application of price fluctuation specified in the contract, remeasurement, day works, variation orders, compensation events, the resolution of disputes, additional or reduced requirements by the Procurement Entity, agreements to extend the time schedule, or from accepted increases or decreases in prices. The contract may allow the Procurement Entity to modify contract values by a pre-determined percentage when this is in the public interest and essential for the work of the Procurement Entity.

All other amendments to costs, quantities, time-periods and other terms and conditions of the contract must be approved by the Tender Committee and confirmed in a formal contract amendment or addendum.

The Procurement Entity will:

- identify and agree with the Contractor the specific clauses in the contract which need to be changed, and the new values or terms and conditions which are to apply;

- prepare a draft contract amendment document for approval by the Tender Committee together with a report justifying the reasons for the amendment,
- following approval by the Entity Tender Committee:
  - record any change in contract value in the Commitment Register and in the Contract Register;
  - record any other contractual changes in the Contract Register;
  - obtain from the Contractor any necessary addition to the Performance Security;
  - arrange for signature of the contract amendment in four copies;

distribute copies in the same way as the original contract.

CHAPTER 6: THE SELECTION AND EMPLOYMENT OF CONSULTANTS

6.1 DEFINITION OF CONSULTANCY SERVICE

Procurement of Consultant Service is the request for services which are of an intellectual and advisory nature provided by firms or individuals using their professional skills to study, design and organise specific projects, advise clients, conduct training or transfer knowledge.

The types of services include the following:

- Sector, Feasibility Studies.
- Preparation of Tender documents.
- Construction Supervision.
- Project Management.
- Procurement Assistance.
- Reorganization/Privatization.
- Institutional Building.
- Training and Knowledge Transfer
- Management Advice
- Cleaning Services

6.2 SPECIAL FEATURES OF CONSULTANCY SERVICE

The use of merit-point evaluation systems and two-envelope tendering procedures are routine features in the procurement and selection of consultants. Selecting consultants for long or complex assignments on the basis of cost alone is unlikely to achieve the required quality of services.

6.2.1 Merit-Point Systems

A merit point system uses a point-scoring basis to determine the winning Tenderer. Points are awarded for technical capability and usually for the financial cost, according to criteria specified in the Request for Proposals. The Tenderer scoring the highest number of points is usually recommended for the award of contract.

Merit point systems can also be used to evaluate whether tenders pass a minimum technical score to proceed to a final financial evaluation The financial envelopes of all Tenderers whose tender pass the minimum technical score are then opened, and the tender with the lowest price recommended for award of contract.

6.2.2 Two-envelope Tendering

To avoid any chance of the Tenderer's price influencing the technical evaluation under a merit point system, financial bids are submitted in a separate sealed envelope. The financial envelope must only be opened after the technical evaluation is completed and approved by the Entity Tender Committee.

6.3 METHOD 0F SELECTING CONSULTANTS

The procurement of consultant services is a specialised form of procurement requiring tender procedures and documents which are very different from those for standard goods and works.

The methods of selection are as follows:

- Quality and Cost Based selection (QCBS).
- Quality Based Selection (QBS).
- Least Cost Selection (LCS).
- Selection Under Fixed Budget (SFB).
- Selection Based on Consultant's Qualification (SBCQ).
- Single Source Selection (SSS).

6.3.1 Quality and Cost Based Selection (QCBS)

Quality and Cost-Based Selection (QCBS) is the standard method of selection for most consultant services, and uses a merit-point score system. The technical capabilities and experience of the Consultants and Personnel, and the quality of the proposal submitted in response to the Terms of Reference, will receive the major percentage of the total points to be awarded. Only firms/consultant whose technical proposals achieved a minimum technical score will have their financial proposal considered. The general practice in Ghana is that, the best compromise between technical quality and cost of the services is often achieved by allocating 80% of the total points to the technical features of the proposal and 20% to the financial score.

6.3.2 Quality-Based Selection (QBS)

Quality-Based Selection (QBS) may be suitable for complex, difficult to define, or highly specialised assignments, where the best expertise available is required without consideration of the price. In this case, only technical proposals are evaluated, with the winning tenderer being invited for detailed negotiations to agree the price of the services and the contract.

- QBS is suitable for the following types of assignments:
  - complex or highly specialised assignments where it is difficult to define precise Terms of Reference (TOR) and the required input from

the consultants, and for which the client expects the consultants to demonstrate innovation in their proposals (for example, sector studies, multi-sector feasibility studies, design of a hazardous waste remediation plant or of an urban master plan, financial sector reforms);

- assignments that have a long term impact and in which the objective is to have the best experts available (for example, feasibility and structural engineering design of such major infrastructure as large dams, policy studies of national significance, management studies of large government agencies); and
- assignments that can be carried out in very different ways, and therefore proposals may not be directly comparable (for example, management advice, or policy studies in which the value of the services depends on the quality of the analysis).

- The Request for Proposals (RFP) should not indicate the estimated budget, but may provide the estimated number of key staff and time, specifying that this information is given as an indication only, and that consultants are free to propose their own staff compositions and estimates.
- The RFP may require submission of a technical proposal only (without a financial proposal), or request submission of both technical and financial proposals at the same time, but in separate envelopes (two-envelope system). Only the financial envelope of the highest ranked technical proposal is opened. The rest are returned unopened to the tenderers, after the negotiations are successfully concluded.
- If technical proposals only are invited, after evaluating the technical proposals, the Consultant with the highest ranked technical proposal will be invited to submit a detailed financial proposal.
- The Procurement Entity and the Consultant should then negotiate the financial proposal and the contract.
- Other aspects of the selection process are identical to those of QCBS.

6.3.3 Selection under a Fixed Budget

Fixed Budget Selection (FBS) may be used when the assignment is simple, can be clearly defined, and there is only a strictly limited budget available for the services. Consultants are invited to submit their best technical proposal within the fixed budget price and award of contract is made to the highest scoring technical proposal

- This method is only appropriate when:
  - the assignment is simple and can be precisely defined; and
  - when the budget is fixed.
- The RFP will indicate the available budget and request the consultants to provide their best technical and financial proposals in separate sealed envelopes, within the stated budget.
- The TOR must be carefully prepared to ensure that the budget is sufficient for the consultants to perform all of the expected tasks.

- Technical proposals will be evaluated and tenderers who pass the minimum technical score will be invited to a public opening of their financial envelopes.
- Tenderers whose technical proposals fail to meet the minimum technical score will have their financial envelopes returned unopened.
- Any financial proposals that exceed the indicated budget should be rejected.
- The Consultant who has submitted the highest ranked technical proposal within the budget will be selected for award of contract.

6.3.4 Least-Cost Selection

This method is more appropriate to selection of consultants for assignments of a standard or routine nature (audits, engineering design of noncomplex works, etc.) where well-established practices and professional standards exist, and when the contract value is small.

- A minimum qualifying score for the required quality is established and stated in the RFP.
- Technical and Financial Proposals are required to be submitted in separate envelopes by the short-listed tenderers.
- Technical envelopes are opened first and evaluated. Those tenders scoring less than the minimum qualifying score are rejected. The financial envelopes of the remaining tenderers are opened in public.
- The firm with the lowest price is selected for contract award.

6.3.5 Selection Based on Consultants' Qualifications

This method may be appropriate for very small assignments where the need for submission and evaluation of detailed competitive proposals is not justified.

- Information on the consultants' experience and competence relevant to the assignment are requested.
- The firm/consultant with the most appropriate qualifications and references is selected.
- The selected firm is invited to submit a combined technical and financial proposal, and then invited to negotiate the proposal and the contract.

6.3.6 Single-Source Selection

Single-source selection of consultants lacks the benefit of competition in regard to quality and cost, the selection is not transparent, and may encourage unacceptable practices. Therefore, Single-source selection should only be used in exceptional circumstances. The justification for Single-source selection must be examined carefully to ensure economy and efficiency.

- Single-source selection is appropriate if there is a clear advantage over competitive selection for instance:

- for tasks that are a natural continuation of previous work carried out by the Consultant;
- where rapid selection is essential (for example, in an emergency situation);
- for very low value assignments;
- when only one firm is qualified or has the necessary experience for the assignment.

Single source selection would be based on the PPB guideline which is available on the PPB website. (www.ppbghana.org)

6.4 USE OF INDIVIDUAL CONSULTANTS

Individual consultants are normally employed on assignments when:

- teams of personnel are not required;
- additional outside (home office) professional support is not required;
- the experience and qualifications of the individual are the major requirement.

If co-ordination, administration, or collective responsibility may become difficult because of the number of individuals required, it may be advisable to employ a firm.

Individual consultants are selected on their qualifications for the assignment.

- Selection may be on the basis of references or through comparison of qualifications among those expressing interest in the assignment or approached directly by the Procurement Entity.
- Individuals must meet all relevant qualifications and be fully capable of carrying out the assignment.
- Capability is judged on academic and professional background, experience, knowledge of local conditions and culture, administrative systems, and government organisation, and language skills as appropriate.

6.5 THE STEPS OF THE PROCUREMENT PROCESS

The procedures and guidelines below are based on the standard QCBS process of selection although other selection methods are available for use in appropriate circumstances

The procurement of consultancy services will normally include the following steps:

- Preparation of the Terms of Reference (TOR).
- Preparation of a cost estimate and confirmation of available budgeted funds.
- Advertising for expressions of interest (if appropriate) or preparation of the shortlist of consultants.
- Preparation and issue of the Request for Proposals (RFP), including:

    - Letter of Invitation (LOI);
    - Information to Consultants (ITC);
    - Draft contract.
- Receipt of proposals.
- Evaluation of technical proposals.
- Evaluation of financial proposals.
- Final evaluation according to the criteria stated in the RFP.
- Negotiations and Award of the contract to the selected firm.

6.5.1 The Terms of Reference

The Terms of reference (TOR) is the key document in the RFP. It ensures that both the Client and the Consultant are fully aware of the objectives and outputs of the services. Consultant services are expensive, and lack of careful thought, research and preparation of the Terms of Reference by the Client, may result in considerable waste of resources.

The Terms of Reference (TOR) should provide sufficient information to enable consultants to fully understand the services required by the Client, and to prepare proposals that are realistic and competitive.

The TOR must be complete, precise, and clear to minimise request for clarifications from consultants. It must be prepared by staff with the requisite expertise.

The Terms of Reference will normally contain the following headings:

1. Background;
2. Objectives;
3. Scope of the Services;
4. Transfer of Knowledge/Training (when appropriate);
5. Deliverables;
6. Assignment Reports and Schedule of Reports;
7. Facilities, services and resources to be provided by the Client;
8. Assignment Period;
9. Assignment Management & Administration

1. Background:

This should be limited to the necessary general background and introduction to the assignment including overall details of the programme or project.

2. Objectives:

The objectives of the assignment should be specified in clear and unambiguous terms

3. Scope of the Services:

The scope of service defines in detail the specific services/duties/activities that the Consultant is expected to perform during the assignment.

4. Transfer of Knowledge/Training (when appropriate):

The transfer of knowledge includes the level of training or transfer of knowledge activities required by the Consultant. This may include on-the-job training of counterpart staff, training seminars and workshops, or the provision of overseas training facilities.

5. Deliverables:

The Deliverables detail the specific outputs expected from the Consultant. These will include reports, achievements and other measurable progress indicators. For Lump-Sum contracts key deliverables are often defined for use with a schedule of interim part payments.

6. Assignment Reports and Schedule of Reports:

The assignment and schedule reports specify the reporting requirements and frequency of reports, and where appropriate the structure of Reports required. Entities should avoid specifying unnecessary routine reports, since these reports can easily distract the Consultant from more productive work for the Client. It should be noted that reports are no substitute for effective personal management of an assignment.

7. Facilities, services, and resources to be provided by the Client:

The facilities, service and resources detail the nature and extent of facilities that will be made available to the Consultants (office accommodation, equipment, support or counterpart staff, etc.), any services (communication, photocopying, stationery, security, etc.) that will be provided, and any other resources or support that the Client (or the Government) will make available.

8. Assignment Period:

The assignment period indicate the anticipated period of time that the Assignment will take to complete.

9. Management & Administration of the Assignment:

The Management and Administration of the assignment details the management arrangements that will apply to the Consultants during the Assignment (who they will report to, frequency of meetings, etc.).

6. 5.2 Estimating Cost and Budget

Preparation of a well thought-through cost estimate is essential for earmarking a realistic budgetary resource for the envisaged service. The cost estimate should be based on the procurement unit's assessment of the resources needed to carry out the assignment.

Cost estimate includes expenses relating to:

- o consultant staff remuneration;
- o travel and transport;
- o mobilization
- o staff allowances;
- o communications;
- o office rent, supplies, equipment, shipping; and insurance;
- o surveys and training programs;
- o report translation and printing;
- o taxes and duties; and
- o contingencies.

Actions:

The Head of Procurement Unit should:

- ❑ Prepare a cost estimate of the envisaged assignment by adding the remuneration of consultant staff and the direct expenses to be incurred by consultants during the execution of their duties. Those figures are based on an estimate of the staff time (per unit of time, hour, month) required to carry out the services and an estimate of each of the related cost components. Since the estimate of the needed staff time is derived from the TOR, the more exhaustive and detailed the TOR, the more precise the estimate.

6.5.3 Expression of Interest (EOI)

A request for Expressions of Interest is required by the Act for consultant services estimated to cost above the threshold stated in Schedule 3 but may be used on lower value contracts to identify consultants who are qualified prior to the issue of an RFP.

6.5.4 EOI Criteria

Pre-qualification is based upon the capability and resources of prospective consultants to perform the assignment satisfactorily, taking into account their:

- ❑ experience and past performance on similar assignments;
- ❑ capabilities with respect to personnel and facilities;
- ❑ required licensing and professional registrations; and
- ❑ commercial and financial resources.

6.5.5 Advertisement for Expressions of Interest

The request for Expressions of Interest shall be advertised in the same way as for Competitive Tenders and shall contain:

- name and address of the Procurement Entity;
- background for the assignment;
- the scope of the services required;
- the location and required timescale for the completion of the services;
- criteria and procedures to be used to evaluate the qualifications of consultants;
- place and deadline for the submission of EOI (which shall not be less than four weeks following the first advertisement).

6.5.6 Evaluation of EOI and Short listing

No formal opening of submissions is required but a formal evaluation of the capabilities of each consultant is required against the criteria as stated in the advertisement.

The following details of each firm should be assessed in the selection of a short-list of between three and eight consultants:

- general background of the consultant;
- eligibility in terms of country of origin, turnover requirements and any other conditions stated in the advertisement;
- previous experience of similar assignments;
- competence and sector related experience of the firm;
- language proficiency in English;
- relevant experience in Ghana, and other West African States; and
- quality of performance under previous contracts.

Where a large number of consultants pass the stated qualification criteria, it may not be realistic to invite more than eight to participate in the specific RFP. If so, only the best qualified consultants should be selected and invited to respond to the RFP.

Inform all applicants of the results of the evaluation. Applicants who fail to meet the pre-qualification criteria or reach the shortlist should be briefly advised of the reasons. No further correspondence will be entered into regarding applications.
On completion of the process and approval of the shortlist by the Tender Committee, the RFP will be issued to the qualified consultants

6.5.7 Preparation and Issue of Request for Proposal (RFP)

The Request for Proposals (RFP) provides all the information necessary for the short-listed consultants to prepare their proposals. It identifies the evaluation criteria, selection method, and procedures that will be used to evaluate the proposals.

The Standard Request for Proposal which can be adapted for any of the selection methods, includes the following sections:

Section 1: Letter of Invitation (LOI);

Section 2: Information to Consultants (ITC) (including the Data Sheet)

Section 3: Technical Proposal —Standard Forms;

Section 4: Financial Proposal—Standard Forms;

Section 5: Terms of Reference; and

Section 6: Standard Form of Contract.

The SRFP has been designed in such a way that some of its parts cannot be modified by the entity, such as the ITC. Other parts, such as the Data Sheet and TOR, are assignment specific and can be used reflect the assignment conditions.

1. Letter of Invitation (LOI)

The Letter of Invitation states the intention of the entity to enter into a contract for a given assignment and informs the short listed consultants that they are invited to submit a proposal for the assignment. It provides basic information regarding

- the name of the entity and the sources of funds to finance the consulting services;
- the names of the short listed consultants;
- a brief description of the objectives and scope of the assignment;
- the method of selection; and
- the date, time, and address for submission of proposals.

The LOI also instructs consultants to indicate whether they intend to submit their proposal alone or in association with other short listed consultants. This information is necessary to allow the entity to invite other consultants in case one or more short listed consultants decline the invitation or decide to associate, thus reducing competition. In these cases, the deadline for submission of proposals may have to be extended.

2. Information to Consultants

The ITC section contains all information consultants need to prepare responsive proposals. It also informs consultants about the evaluation criteria and sub criteria, their respective weights and the minimum qualifying mark, in order to provide for a fair and transparent selection process. The ITC should not be modified other than through the Data Sheet.

The Data Sheet is the part of the ITC that contains specific information relating to the entity and the assignment.

3. Technical Proposal Standard Forms

This section contains the standard forms which are to be completed by the Consultant as part of the technical proposal. The Consultant is required to

complete and submit these forms pursuant to ITC Clause 3.4 and other requirements of the Request for Proposals:

- 3A. Technical Proposal submission form;
- 3B. Firm's references;
- 3C. Comments and suggestions of consultants on the Terms of Reference and on data, services, and facilities to be provided by the Client;
- 3D. Description of the methodology and work plan for performing the assignment;
- 3E. Team composition and task assignments;
- 3F. Format of curriculum vitae (CV) for proposed professional staff;
- 3G. Time schedule for professional personnel;
- 3H. Activity (work) schedule.

4. Financial Proposal –Standard Forms

This section contains the standard forms which are to be completed by the Consultant in submitting the financial proposal. The Consultant is required to complete and submit the following forms pursuant to ITC Clause 3.6 and in accordance with other requirements included in the Request for Proposals:

- 4A Financial Proposal submission form;
- 4B. Summary of costs;
- 4C. Breakdown of price per activity;
- 4D. Breakdown of remuneration per activity;
- 4E. Reimbursable per activity;
- 4F. Miscellaneous expenses.

5. Standard Forms of Contract

The following are various forms of Contract that an entity can use depending on the nature of the assignment:

- Consultant Services - Complex Time-Based Assignments
- Consultant Services - Lump-Sum Assignments
- Small Time-Based Assignments
- Small Lump-Sum Assignments

6.5.8 Technical Evaluation Criteria

The criteria specified in the RFP should include:

- the firm's relevant experience for the assignment;
- the quality of the methodology proposed;
- the qualifications and experience of the key staff proposed;
- the extent of participation by nationals among key staff in the performance of the assignment.

The marks for each criterion are aggregated to give the total technical score.

The following table shows the normal range of points to be specified for each criterion, which may be adjusted for specific circumstances. The proposed points must be declared in the SRFP.

Figure 1 - Indicative Weighting of Evaluation Criteria (Consultant Services)

Specific relevant experience:	0 to 10 points
Response to the TOR and Methodology Proposed:	20 to 50 points
Key personnel:	30 to 60 points
Training:[1]	0 to 10 points
Participation by nationals:[2]	10 to 15 points
Total:	100 points

The criteria may be divided into sub-criteria to assist the objectivity of the evaluation. For example, sub-criteria under methodology might be *innovation* and *level of detail.* It is usual to use sub-criteria for key staff to evaluate their qualifications, technical experience and language capabilities. The number of sub-criteria should be kept to the essential minimum and must be fully detailed within the RFP.

Consultant's Specific Experience

The points given to experience can be relatively low if this criterion has already been taken into account when short-listing the consultant firms.

Methodology

[1] Transfer of knowledge may be the main objective of some assignments; in such cases, it should be given a higher weight to reflect its importance.

[2] As reflected by Ghana nationals among key staff presented by foreign and national firms constituting 40% or more as provided in margin of preference.

A large number of points should be given to the response and proposed methodology for more complex assignments (for example, multi-disciplinary feasibility or management studies).

Key Personnel

Only the key personnel should normally be evaluated since they will determine the quality of performance. More points should be assigned if the proposed assignment is complex.

- When the assignment depends critically on the performance of a Project Manager or key specialist in a team of individuals, more points should be allocated for this person.
- Individuals should be evaluated on the following sub-criteria as relevant to the task:
  - General Qualifications: General education and training, professional qualifications, length of experience, positions held, time with the consulting firm, experience in similar countries, etc;
  - Adequacy for the Assignment: Specific experience relevant to the assignment in the sector, field, subject, process or activity; and
  - Experience in the Region: Knowledge of local culture, administrative systems, government organisations, etc.

6.5.9 Minimum Technical Score

The minimum qualifying technical score to be achieved for a proposal to proceed to the Financial Evaluation must be specified in the RFP.

6.5.10 Financial Evaluation Criteria

Only proposals that have achieved the pass mark for technical proposals shall be subjected to financial evaluation. In addition to specifying the weighting for technical and financial scores, the RFP must specify the formula for award of points to each proposal price. Normally the lowest priced proposal receives 100 points and the other proposals receive points based on dividing their prices by the lowest priced proposal and multiplying by 100.

The standard procedures for correcting arithmetic and other errors in tender prices will apply to adjust the proposal price before the points are awarded to each proposal.

6.5.11 Weighting of Technical and Financial Scores

The relative weightings for technical and financial scores must be stated in the RFP. This is usually set at 80% for the technical score and 20% for the financial score. In this case the technical score will be multiplied by 80% and the financial score by 20% to give the total score for each proposal

6.6 CONTRACT AGREEMENT FOR EMPLOYMENT OF CONSULTANTS

The type of contract must be selected when preparing the Request for Proposals and included as a draft with all relevant contract terms and conditions in the RFP.

6.6.1 Lump Sum (Fixed Price) Contracts

Lump sum contracts are used mainly for assignments in which both the content and the duration of the services; and the required output of the consultants are clearly defined:

- ❑ Lump sum contracts are widely used for simple planning and feasibility studies, environmental studies, detailed design of standard or common structures, preparation of data processing systems, etc.
- ❑ Payments are linked to defined outputs (deliverables), such as reports, drawings, bills of quantities, tender documents, and software programs.
- ❑ Lump sum contracts are simple to administer because payments are due on attainment of clearly specified outputs.

6. 6.2 Time-Based Contract.

This type of contract is widely used for complex studies, supervision of construction, technical advisory services, and training assignments. It may also be appropriate when:

- ❑ it is difficult to define the full scope of services, or the input of the consultants required to attain the objectives of the assignment;
- ❑ the length of services can be precisely defined and deliverables are only incidental to the main purpose of the assignment;
- ❑ the services are related to activities by others for which the completion period may vary.

Payments are based on:

- ❑ Remuneration: Agreed hourly, daily, weekly, or monthly rates for staff;
- ❑ Reimbursable: Reimbursable items using actual expenses and/or agreed unit prices.
- ❑ The rates for staff remuneration include salary, social costs, overhead, fee (or profit), and, where appropriate, special allowances.

This type of contract must include a maximum amount of total payments (the contract ceiling) to be made to the consultants.

The contract ceiling usually includes a contingency allowance for unforeseen work and duration, and provision for price adjustments, where appropriate.

Time-based contracts need to be closely monitored and administered by the Procurement Entity to ensure that the assignment is progressing satisfactorily, and payments claimed by the consultants are appropriate.

6.6.3 Retainer and/or Contingency (Success) Fee Contract.

Retainer and contingency fee contracts are frequently used when consultants (banks or financial firms) are undertaking specialist financial activities such as preparing companies for sale, in mergers of firms, or in privatisation operations.

- ❑ The remuneration of the Consultant includes a retainer and a success fee, the latter being normally expressed as a percentage of the sale price of the assets.

6.6.4 Percentage Contract.

These contracts are commonly used for architectural services but may be also used in similar circumstances such as for procurement and inspection agents.

- Percentage contracts directly relate the fees paid to the Consultant to the estimated or actual project construction cost, or the cost of the goods procured or inspected.
- Contracts are negotiated on the basis of market standards for the services and/or estimated staff-month costs for the services.
- In the case of architectural or engineering services, percentage contracts lack any incentive for economic design or performance. The use of a percentage contract format for architectural services is only recommended if based on a fixed target cost and covers precisely defined services.

6.6.5 Definite Delivery Contract (Price Agreement).

These contracts are used when there is a need for "on call" specialised services to provide advice or services, the extent and timing of which cannot be defined in advance.

- These are commonly used to retain "advisers" for implementation of complex projects expert adjudicators for dispute resolution panels, institutional reforms, procurement advice, technical troubleshooting, etc. normally for a period of a year or more.
- The Procurement Entity and the firm agree on the unit rates to be paid, and payments are made on the basis of the time and resources actually used.

6.7 TENDER AND PERFORMANCE SECURITY

Tender and performance securities for consultants' services are not recommended for the following reasons:

- tender securities are not an accepted standard for consultanting services tenders and are likely to discourage participation by international consultanting firms;
- enforcement of tender securities may be subject to dispute for example when final negotiations fail to reach a satisfactory conclusion;
- performance securities can be easily abused by the Client as personality clashes or other factors beyond the direct control of the Consultant may affect achievement under the contract;
- there is often a strong element of subjectivity rather than objectivity in determining the success or failure of an assignment;
- securities increase the costs to the consulting industry without evident benefits, and the costs are inevitably passed on to the Client through higher prices.

However, the procurement entity may resort to the use of professional indemnity policy.

6.8 RECEIPT AND OPENING OF TECHNICAL PROPOSALS

Tender Opening shall commence immediately after the deadline for submission of proposals (as stated in the RFP)

The Procurement Unit will co-ordinate the opening, ensure smooth operation of the proceedings, take a register of attendance, prepare Minutes of the opening, and advise the Chairperson of the opening session on procedural issues if requested.

The Tender Opening Panel shall comprise at least 3 persons, including a member of the Entity Tender committee. They shall ensure that minutes of the opening proceedings are duly written.

The Chairperson of the Tender Opening Panel will control and direct the proceedings and not allow consultant's representatives to interfere with the work of the Panel. Any objections by a Consultant to the procedures or decisions of the opening should be made in writing to the Head of the Procurement Entity.

For purposes of transparency, it is not permitted for the opening to be halted or postponed once the process begins.

Actions:

The Chairperson should:

- ❑ Ensure that consultants' representatives are seated separately from the Tender Opening Panel and officials of the Procurement Entity, and that the name, organisation represented and contact details of all attendees are recorded in an attendance register.
- ❑ Shall open the meeting and outline the procedures to be used for the opening of Technical Proposals.
- ❑ Ensure the security of documents at all times during the opening procedure to prevent any unauthorised interference with the documents and process.
- ❑ Open the tender box, check that the inscription on each envelope confirms that it matches the correct proposal and complies with the wording and sealing required in the RFP. Stack all envelopes in clear view ready for opening.
- ❑ Check for any withdrawals or modifications submitted, and match these with the original Proposal before proceeding. Withdrawn proposals shall not be opened once the authenticity of the withdrawal notice has been confirmed.
- ❑ Open the first proposal after confirming that all conditions regarding marking and sealing of the envelope have been met and the envelope has not been tampered with.
- ❑ Examine the Technical and Financial Proposal envelopes inside to confirm that all conditions regarding marking and sealing of both envelopes have been met and the envelopes have not been tampered with.

- Sign as received and place the Financial Proposal envelope unopened in a separate pile and secure the proposals back into the tender box. This should open only after the completion of the Technical Evaluation.
- Examine the contents of the envelope and identify, stamp and number all originals and copies, and any separate sections and attachments.
- Read out the following details of each Technical Proposal from the Original copy:
  - any Proposal modifications or withdrawals;
  - the number allocated to the Proposal by the Tender Opening Panel;
  - the name and country of the Consultant; and
  - any other appropriate information at the discretion of the Chairman.
- Any envelopes containing substitutions, or modifications, must be subject to the same level of scrutiny, including the reading out of critical details.
- Any obvious failure to provide a responsive Proposal shall be reported to the Meeting and recorded in the Minutes.
- The Chairperson and two Members of the Panel shall initial the original of each Technical Proposal and all attachments thereto. Any corrections or obvious errors and omissions noted shall be circled in red ink and also initialled.
- Record the details read out of each Proposal in the Register of Opening, and record any corrections and errors or omissions that were noted during the opening and captured in the Minutes of the Opening.

Minutes of the Opening shall be prepared by the Procurement Unit, signed by the Chairperson of the Tender Opening Panel and made available to any consultant submitting a Proposal who requests a copy in writing.

The Technical Proposals should be handed over to the Tender Evaluation Panel for evaluation.

6.9 EVALUATION OF TECHNICAL PROPOSALS

- The Tender Evaluation Panel should meet shortly before the deadline for submission of the proposals to confirm that there is a common understanding of the evaluation method, the evaluation criteria and sub criteria specified in the data sheet, and the selection procedure. They should also familiarise themselves with the RFP (in particular the TOR). It is important not to wait until after the technical proposals are opened to define the rating system, since these definitions could be biased by the knowledge of the contents of the proposals.

The Evaluation Panel should confirm that its members

- have no conflict of interest;
- understand the rating and scoring system;
- have been provided with evaluation worksheets; and
- agree on how to evaluate the proposals.

A meeting of the Tender Evaluation Panel should be held to examine the Technical Proposals to confirm substantial responsiveness to the conditions specified and that there are no important omissions or deviations from the stated objectives, TOR, or other key requirements of the RFP.

The Tender Evaluation Panel may request clarifications from consultants concerning ambiguities or inconsistencies in the Proposal. Such requests shall be in writing, and no change in the scope of the originally offered services may be sought or accepted. The responses from consultants shall also be in writing.

Each evaluator should conduct his or her detailed technical evaluation independently, and record scores using the format provided in the standard evaluation format provided by the PPB. Evaluators should also separately note particular strengths and weaknesses of each proposal to assist them in justifying the scores they have awarded.

Following completion of individual evaluations, the Evaluation Panel shall meet again to combine the scoring and to discuss the reasons for any wide variations in the scores awarded by individuals.

The Evaluation Panel will then prepare the Technical Evaluation Report using the standard format for approval by the Tender Committee.
Please find a sample Technical and Financial calculations in figure 2 and 3 below.

6.10 OPENING OF FINANCIAL PROPOSALS

Following approval of the Technical Evaluation Report, the Procurement Entity shall notify any firms that failed to reach the required minimum technical score, and invite those firms that passed the minimum score to the opening of Financial Proposals.

The opening of Financial Proposals shall commence immediately after the deadline notified to all consultants invited to attend.

The Procurement Unit will co-ordinate the opening, ensure smooth operation of the proceedings, take a register of attendance, prepare Minutes of the opening, and advise the Chairperson of the opening session on procedural issues if requested.

The Chairperson of the Tender Opening Committee will control and direct the proceedings and not allow consultant's representatives to interfere with the work of the Committee. Any objections by a Consultant to the procedures or decisions of the opening should be made in writing to the Head of the Procurement Entity.

For purposes of transparency it is not permitted for the opening to be halted or postponed once the process begins.

Action:

The Chairperson of the Opening Committee should:

- ❑ Open the meeting and outline the procedures to be used for the opening of Financial Proposals.
- ❑ Ensure that consultants' representatives are seated separately from the Tender Opening Committee and officials of the Procurement Entity, and that the name, organisation represented and contact details of all attendees are recorded in an attendance register.
- ❑ Ensure the security of documents and proposals at all times during the opening procedure to prevent any unauthorised interference with the documents and process.
- ❑ Bring in the unopened Financial Proposals and place in clear view of all participants.
- ❑ Read out the Technical Scores awarded to each consultant during the technical evaluation.
- ❑ Open the first Financial Proposal after confirming with the Consultant that his or her envelope has not been opened or tampered with in any way.
- ❑ Examine the contents of the envelope and identify, stamp and number all originals and copies, and any separate sections and attachments.
- ❑ Read out the following details of each Financial Proposal from the Original copy:
  - o the number allocated to the Proposal by the Tender Opening Committee;
  - o the name and country of the Consultant; the currency of the Proposal;
  - o the total Proposal price;
  - o any discounts offered; and
  - o any other appropriate information at the discretion of the Chairperson.
- ❑ Any obvious failure to provide a responsive proposal shall be reported to the Meeting and recorded in the Minutes.
- ❑ The Chairperson and two Members of the Committee shall initial the original of each Financial Proposal and all attachments thereto. Any corrections or obvious errors and omissions noted shall be circled in red ink and also initialled.
- ❑ Record the details read out of each Proposal in the Register of Opening, and record any corrections and errors or omissions that were noted during the opening and capture in the Minutes of the Opening.

Minutes of the Opening shall be prepared by the Procurement Unit, signed by the Chairman of the Tender Opening Committee and made available to any consultant submitting a Proposal who requests a copy in writing.

The Financial Proposals should be handed over to the Tender Evaluation Panel for evaluation whilst the original is kept under lock and key. .

6.11 EVALUATION OF FINANCIAL PROPOSALS

The Tender Evaluation Panel should examine the Financial Proposals to confirm substantial responsiveness to the conditions specified and that there are no important omissions or deviations from key requirements of the RFP.

The Tender Evaluation Panel may request clarifications from consultants concerning ambiguities or inconsistencies in the Financial Proposal. Such requests shall be in writing, and no change in the price or scope of the originally offered services may be sought or accepted, except for the correction of arithmetic errors. The responses from consultants shall also be in writing.

Action:

The Tender Evaluation Panel shall:

- ❑ Correct any purely arithmetical errors in tenders in accordance with the procedure stated in the RFP. Note that if there is any discrepancy between numbers and written figures, the written figures shall prevail. If both the number and the total price are indicated in the Proposal, arithmetic should be checked and if there is a discrepancy, the unit price shall govern. All errors in extension (multiplying the unit price by the number) and totalling should be corrected and the Consultant notified. A Consultant cannot be permitted to retain an arithmetical error in extension or totalling and correct the unit price. Notify Consultants of any such arithmetic corrections, and request written, agreement of the Consultant to the correction. If a Consultant does not accept the correction of an arithmetical error, his Proposal must be rejected.

  Any communications between the Procurement Entity and a Consultant during the examination of Proposals shall be made in writing
- ❑ Calculate the financial score of each proposal applying the formula specified in the RFP.
- ❑ Combine the technical and financial scores for each proposal using the methodology stated in the RFP, rank proposals in the order of their total scores and recommend the Consultant with the highest score for contract negotiations.
- ❑ Complete the Evaluation Report using the standard format with recommendations for approval by the Tender Committee.

Figure 2: Sample of QCBS Combined Technical /Financial Evaluation-Award Recommendation

Consultants' names	Technical Evaluation			Financial Evaluation		Combined Evaluation	
	Technical Scores[1]	Weighted Scores S(t) x T[2]	Technical rank	Financial Scores[3] S(f)	Weighted Scores S(f) x F[4]	Scores S(t) + S(f) F	Rank
A	92.00	73.60	3	100	20	93.60	1
B	90.00	72.00	4	71.43	14.28	86.28	4
C	88.00	70.40	5	59.66	11.93	82.33	5
D	93.56	74.80	1	66.60	13.32	88.12	3
E	92.89	74.30	2	77.55	15.51	89.81	2

Figure 3: Sample Financial Evaluation Results

Consultants' Names	Proposals' prices[1]		Adjustment[2]	Evaluated price(s)	Conversion to currency of evaluation[3]		Financial scores[4]
	Currency	Amounts (1)	(2)	(3) = (1) + (2)	Exchange Rate(s)[5] (4)	Proposals' price (5)=(3)(4)	(6)
A	GHC	222,000,000	-	222,000,000	N/A	222,000,000	20
B	USD	37,735	-	37,735	8,236	310,785,460	14.28
C	USD	45,180	-	45,180	8,236	372,102,480	11.93
D	USD	40,470	-	40,470	8,236	333,310,920	13.32
E	GHC	286,241,000	-	286,241,000	N/A	286,241,000	15.51

1. Comment if any (e.g., exchange rates); three foreign currencies maximum, plus local currency
2. Arithmetical errors and omissions of items included in the technical proposals. Adjustments may be positive or negative.
3. As per REP.
4. 20 points to the firm with lowest price; other scores to be determined in accordance with provisions of REP.
5. Value of local currency (e.g., USDI = 8236.). Ghana cedis, selling exchange rate at 30/08/2002

6.12 NEGOTIATION OF CONTRACT

Following approval of the Evaluation Report and recommendations by the Tender Committee, the Procurement Entity shall invite the recommended Consultant for contract negotiations in accordance with the procedure stated in the RFP.

- ❑ Negotiation is expected to ensure agreement on all points and to result in an agreed draft contract. Negotiation may include discussions on the technical proposals, the proposed methodology (Work Plan), staffing and

any suggestions that may be made have been made by the consultant to improve the TOR. The Procurement Entity and the Consultant shall then establish the final TOR, staffing, scheduling of services and clarify any general financial issues such as taxes payable and mode of payment.

- ❑ The selected firm should not be allowed to substitute key staff, unless both parties agree that undue delay in the selection process makes such substitution unavoidable or that such changes are critical to meet the objectives of the assignment. Any substitution of key staff by the Consultant will require the offering of an equivalent or better candidate.
- ❑ Where the selection process specifies a time based form of contract, the Consultant is requested to provide justification of the unit rates quoted for individual staff. Negotiation of staff rates and reimbursable expenses may be discussed in this instance. Financial adjustments to any lump-sum price proposals may only be made through minor modifications to the scope of the proposed services.
- ❑ A detailed record of negotiations shall be maintained and signed by the Procurement Entity and the Consultant.
- ❑ The final TOR, agreed methodology and any financial adjustments shall be incorporated in the draft contract for approval of contract award by the Tender Committee or appropriate Review Board.

6.13 AWARD OF CONTRACT

Following approval from the relevant review body, the contract will be awarded.

Before issue of the contract, a formal commitment of the required funds against the budget of the Procurement Entity must be approved.

Action:

The Head of Procurement Unit should:

- ❑ Prepare/update the Activity and Expenditure Initiation (A&EI) Form and obtain approval of the commitment of funds against the budget of the Procurement Entity.
- ❑ Prepare four copies (minimum) of the contract ready for signature by each party to the contract, and include all specific details relating to the Consultant, the Conditions of Contract, and the Consultant's offer. A clear statement on when the contract becomes effective is also essential since certain contract clauses such as mobilisation, and advance payment issues could be connected with this date
- ❑ Obtain the signature of the Head of Procurement Entity on all copies of the contract.
- ❑ Note that the four copies (minimum) of the Contract will be distributed after signature as follows:
  - o Original – Procurement Unit;
  - o 1st Duplicate – Consultant;
  - o 2nd Duplicate – the Technical Department concerned; and

- 3rd Duplicate – Legal Officer.

- Ensure that the contract is duly recorded in the Contracts Register maintained by the Procurement Unit.

6.13.1 Award Notification

A formal notice of tender award shall be issued to the successful Consultant, who will be required to confirm in writing acceptance of the contract award.

- The Consultant shall be invited to attend for contract signature, or where this is not practical, provided with the copies of the Contract for signature and return of the original and two signed copies of the Contract to the Procurement Entity.
- Failure of the Consultant to confirm acceptance of the award, or to sign the contract will constitute grounds for the annulment of the award. In that event, the Procurement Entity may cancel the proceedings and invite the Consultant who submitted the next ranked Proposal for negotiations.
- Procurement Entities are required to submit notice of contract awards to the Public Procurement Board not later than 30 days after contract signature, for publication on the PPB website and/or in the Procurement Bulletin. The information on contracts awarded/signed shall include the:
  - Name of entity;
  - Name of the Consultant;
  - Description of the services;
  - Contract Sum;
  - Start and finished dates of the contract;
  - Method of procurement used; and
  - Source of funding.

6.13.2 Notification to Unsuccessful Tenderers

All unsuccessful Consultants should be notified immediately once the contract has been awarded.

6.14 CONTRACT MANAGEMENT

Effective management of contracts is essential to ensure that the objectives of the procurement process are achieved and that all contractual obligations and activities are completed efficiently by both parties to the contract. The Procurement Unit or the Technical Department concerned must ensure that routine monitoring of all current contracts is maintained so that swift remedial measures can be taken when problems arise, or preventive action taken when problems are foreseen.

6.14.1 Contract Supervision and Administration

Contract supervision and administration shall be undertaken by the Head of the Procurement Unit in consultation with the relevant technical department.

Action:

- ❑ Ensure that any contractual obligations of the procurement entity are provided in a timely and efficient manner
- ❑ Maintain routine supervision of the Consultant's performance and progress to ensure that potential problems are identified as early as possible, and notify the Consultant in writing requesting rectification of any deficiencies in required performance standards;
- ❑ Receive regular progress reports from the Consultant and ensure that targets for submission of key deliverables required are met.
- ❑ Conduct detailed checks on the Consultants claims for payment to ensure that contractual requirements are met and process invoices for payment.
- ❑ Confirm the provision of all contract deliverables before closing the contract file.

6.14.2 Payment for Consultant Services

For release of any Advance Payment or subsequent invoices approved by the Head of Entity. The Head of the Procurement Unit will:

- ❑ Complete a Payment advice and forward to Accounts department. ensuring the deduction of any advance payments already made, and any contractual penalties incurred by the Consultant.
- ❑ Attach the:
  - o original invoice from the Consultant;
  - o copies of relevant information from the contract document, records of approval and financial authorisations.

6.14.3 Resolution of Contractual Disputes

Most minor disputes may be resolved by discussion and agreement between the Head of Procurement Unit and the Consultant to rectify the cause of complaint.

Any formal written complaints received from a Consultant should be fully investigated and referred to the Head of the Procurement Entity to authorise correspondence or formal negotiations with the Consultant.

Action:

The Head of the Procurement Unit should:

- ❑ Examine the Contract carefully to be aware of all contract conditions relating to the Resolution of Disputes.
- ❑ Determine if the Procurement Entity is at fault or partly at fault, and if so, take appropriate action to rectify the problem.
- ❑ Invite the Consultant to a formal meeting, within 7 days of the complaint, to discuss the issues and try to agree a compromise acceptable to both parties. Ensure that accurate written Minutes are kept of any such meeting. If an agreement is reached which changes any of the conditions of the Contract, approval of the Tender Committee or the Head of the Procurement Entity is required before the agreement can be implemented.
- ❑ If no initial agreement is reached and negotiations conducted by the Head of the Procurement Entity also fail, consider the use of any adjudication or arbitration services as specified in the contract.
- ❑ Prepare any necessary addendum to the Contract for signature.

6.14.4 Termination of the Contract

The parties to the contract normally have the right to terminate the contract, but to protect the Procurement Entity, advice of the Legal Department should be sought. Contracts should not be terminated without examining all possible alternatives, unless the termination is mutually agreed by all parties to the contract.

Action:

The Procurement Entity should:

- ❑ Examine the Contract carefully to be aware of all contract conditions and penalties relating to the Termination of Contract.
- ❑ Follow the advice of the Legal Officer in the preparation of any correspondence.

6.14.5 Contract Amendment

Contract amendment may become necessary as a result of the resolution of disputes, additional or reduced requirements by the Procurement Entity, agreements to extend the time schedule, or from accepted increases or decreases in prices. The contract may also allow the Procurement Entity to modify contract values by a pre-determined contingency percentage when this is in the public interest and essential for the work of the Procurement Entity.

All other amendments to costs, services, time-periods and other terms and conditions of the contract must be approved by the Entity Tender Committee and confirmed in a formal contract amendment or addendum.

The Procurement Entity should:

- ❑ identify and agree with the Consultant any specific clauses in the contract which need to be changed, and the new values or terms and conditions which are to apply;
- ❑ prepare a draft contract amendment document for approval by the Tender Committee together with a report justifying the reasons for the amendment,
- ❑ following approval by the Tender Committee:
  - o record any change in contract value in the Commitment Register and in the Contract Register;
  - o record any other contractual changes in the Contract Register;
  - o arrange for signature of the contract amendment in four copies;

distribute copies of the contract amendment in the same way as the original contract.

CHAPTER 7 PROCUREMENT REPORTING, MONITORING & EVALUATION

7.1 PROCUREMENT REPORTING

The routine reporting of procurement activity by all Procurement Entities is essential for procurement planning and budgeting, and for the monitoring, tracking and evaluation of procurement. A regular monthly reporting of procurement activity to the Public Procurement Board is introduced by this Manual.

The Monthly Report of Procurement shall be submitted by each Tender Committee using Form SPF 5 within 14 days of the end of each calendar month.

The Monthly Report format on Form SPF 5 is in five sections as follows:

- Section A: Procurements in Process (estimated cost above GHC 50 million) - Report all procurements which have passed the initial stage of approval by the Tender Committee during the month.
- Section B: Contracts Signed or Procurements Cancelled (above GHC 50 million) -Report all new contracts signed during the month together with any procurements which have been cancelled after receiving an initial procurement approval by the Entity Tender Committee.
- Section C: Contracts Completed (above GHC 50 million) - Report all contracts completed during the month following performance by the supplier, final payment by the Procurement Entity and the release of any performance security.
- Section D: Minor Procurements under GHC 50 million – List individual contracts signed for a value above GHC 10 million during the month, and give totals only by Department or Unit for purchases costing less than GHC 10 million.
- Section E: Tracking of payment above GHC 10 million- Report all payment schedules for contracts above GHC 10 million, indicating delays if any and causes.

7.1.1 Section A: Procurements in Process above GHC 50 million

Record:

- Procurement Number;
- Subject of Procurement;
- Procurement Method approved (Open Tender, Limited Tender, Request for Quotations or Direct Procurement);
- Source of Funds (e.g. Government, World Bank, other donor);
- Type of Approval given by the Entity Tender Committee (e.g. procurement method, evaluation report and contract award);
- Date of Approval; and
- Estimated Contract Value.

7.1.2 Section B: Contracts Signed or Procurements Cancelled above GHC 50 million

Record:

- Procurement Number;
- Subject of Procurement;
- Name of Supplier/Contractor;
- Source of Funds;
- Date of Contract Award;
- Date of Contract Cancellation;
- Contract Value.

7.1.3 Section C: Contracts Completed above GHC 50 million

Record:

- Procurement Number;
- Subject of Procurement;
- Name of Supplier/Contractor;
- Source of Funds;
- Date of Contract Completion;
- Date of Final Payment and Release of Performance Security;
- Total Amount Paid under the Contract.

7.1.4 Section D: Minor Procurements under GHC 50 million

Record:

- Procurement Number;
- Subject of Procurement;
- Name of Supplier/Contractor;
- Procurement Method used (Request for Quotations or Direct Procurement);
- Date of Contract Signature;
- Contract Cost.

For procurements under GHC 10 million, enter the Department, Unit or Project name in "Subject of Procurement" and the total value of contracts or direct payments in the month in "Contract Cost".

7.1.5 Section E: Payment to Suppliers/Contractors/Consultants above GHC 10 million

Record:

- Procurement Number:
- Subject of Procurement;
- Name of Supplier/Contractor/Consultant;

- o Date of Contract Signature;
- o Payment Terms;
- o Status of Payment:
- o Date of Final Payment;

7.2 PROCUREMENT MONITORING AND EVALUATION

Procurement monitoring and evaluation is a process that must be routinely conducted both by Procurement Entities and by the Public Procurement Board to:

b) ensure that procurements are within the annual procurement plan for the Procurement Entity;

c) identify weaknesses and delays in the procurement process;

d) compare prices against market standards;

e) assess performance of Procurement Units and Committees;

f) assess performance of suppliers, contractors and consultants; and to

g) identify any necessary remedial action.

The Public Procurement Board will co-ordinate and lead the monitoring and evaluation process through the methodologies described below.

7.2.1 Sources of Information

Procurement monitoring and evaluation information is obtained from:

h) Requests for deviation from procurement procedures submitted by Procurement Entities;

i) Monthly Reports of Procurement on Form SPF 5;

j) Regular meetings with stakeholders (including representatives of the Private Sector);

k) Routine review and certification of Procurement Entities for decentralised procurement;

l) Reports of the Auditor General and the Internal Audit Agency;

m) Reports of specialist external procurement audits;

n) Tenderer and supplier complaints made to Procurement Entities;

o) Complaints by tenderers referred to the PPB for Administrative Review;

p) Routine sampling of record of procurement proceedings for individual procurements; and

q) Examination of the record of procurement proceedings for any procurement which appears to deviate from compliance with the Act and Regulations.

7.2.2 Action by the Public Procurement Board on Receipt of Form SPF5

The basis of the monitoring system is the Monthly Report on Form SPF5. On receipt of the Monthly Report from a Procurement Entity, the PPB will:

a) Check that procurement activity recorded is within the authority levels of the Entity Tender Committee or Tender Review Board and in accordance with the annual procurement plan;

b) Check that any new procurement processes commenced are within the Procurement Plan for the Procurement Entity;

c) Identify any procurement decision which appears to deviate from compliance with the Regulations, and query this with the Tender Committee/Tender Review Board;

d) Inspect or call for the record of procurement proceedings where serious breaches of the Act, Regulations or Code of Ethics are suspected;

e) Enter the details of each new procurement into the database, using the Procurement Number as the main index key, and update existing information as reported throughout the individual procurement process.

The database information will be used to:

a) provide Procurement Entities with summary reports on procurement activity;

b) compile the statistical analysis of procurement activity for the Annual Report of the PPB;

c) identify the scale and values of common user items that may benefit from the introduction of framework contracts;

d) identify regular procurements of identical items by a Procurement Entity which may indicate that requirements have been split to avoid an approval threshold or a procurement procedure; and

e) determine the individual and average timescales for procurement processes.

7.2.3. Other Monitoring and Evaluation Activities by the PPB

a) Conduct regular meetings with stakeholders (including representatives of the Private Sector) to report on procurement issues and initiatives, and to initiate investigation of complaints and proposals from stakeholders.

b) Undertake periodic reviews of the procurement performance of Procurement Entities and advise on specific issues which need to be addressed.

c) Review and initiate remedial action as appropriate in response to audit reports of the Auditor General, the Internal Audit Agency, and any specialist external procurement auditors.

e) Review and initial remedial action as appropriate in respect of tenderer and supplier complaints.

f) Undertake a programme of routine sampling of records of procurement proceedings for individual procurements and report findings and recommendations to the Procurement Entity.

g) Review procurement procedures, systems, guidelines and standard documentation on a routine basis, and make or introduce recommendations for improvement.

h) Compile all findings and action into the Annual Report of the Public Procurement Board.

7.2.4 Action by the Procurement Entity

The Tender Committee has the general responsibility to monitor procurement activity and remedy any defects before giving approval for procurement actions. The advice of the Public Procurement Board should always be sought if the Committee is unsure of compliance with the Regulations.

The Head of Procurement Entity will ensure that all issues raised by the Public Procurement Board are actioned expeditiously by the Procurement Entity through the Entity Tender Committee and Procurement Unit, and that complete records of procurement are made available for audit purposes and for inspection by the PPB.

CHAPTER 8: COMPLAINTS ON THE PROCUREMENT PROCESS

8.1 REVIEW OF PROCUREMENT PROCESS

Any supplier, contractor or consultant that claims to have suffered, or that may suffer loss or injury due to a breach of a duty imposed on the Procurement Entity by the Act, may seek a review of the procurement process. However, the following shall not be subject to a review process:

- the selection of the method of procurement used;
- the choice of a selection procedure;
- the limitation of procurement proceedings to National Competitive Tendering; or
- a decision by the Procurement Entity to reject tenders, proposals, offers or quotation.

A complaint shall, in the first instance, be submitted in writing to the Head of the Procurement Entity.

8.1.1 Review by Head of Procurement Entity

The Head of the Procurement Entity shall not entertain a complaint unless it was submitted within twenty (20) days after the supplier, contractor or consultant submitting it became aware of the circumstances giving rise to the complaint, or when that supplier, contractor, or consultant should have become aware of those circumstances, whichever is earlier.

The Head of the Procurement Entity may entertain or continue to entertain a complaint after the procurement contract has entered into force.

A Procurement Entity shall attempt to resolve a complaint by mutual agreement with the supplier, contractor or consultant.

The Head of the Procurement Entity shall, within twenty-one days after the submission of the complaint, issue a written decision:

- stating the reasons for the decision; and
- if the complaint is upheld in whole or in part, indicating the corrective measures that are to be taken.

If the Head of the Procurement Entity does not issue a decision within twenty-one days, the supplier, contractor, or consultant, submitting the complaint is entitled to institute proceedings for administrative review by the Public Procurement Board.

Following the institution of proceedings for administrative review by the Public Procurement Board, the competence of the Head of the Procurement Entity to entertain the complaint ceases.

8.1.2 Administrative Review by the Public Procurement Board

A supplier, contractor or consultant entitled to seek review may submit a complaint to the Public Procurement Board:

- within twenty-one days after:

  - o the supplier, contractor or consultant became aware of the circumstances giving rise to the complaint; or
  - o the time when the supplier, contractor or consultant ought to have become aware of those circumstances,
- ❑ if the complaint cannot be submitted through the Head of Procurement Entity because of the entry into force of the procurement contract;
- ❑ if the Head of the Procurement Entity does not entertain the complaint because the procurement contract has entered into force, and the complaint is submitted within twenty-one days after the issuance of the decision by the Head of Procurement Entity not to entertain the complaint;
- ❑ if the Head of the Procurement Entity does not issue a decision within twenty-one days after the submission of the original complaint and the complaint is submitted to the Public Procurement Board within twenty-one days after the expiry of the period for review by the Head of Procurement Entity; or
- ❑ if the supplier, contractor, or consultant claims to be adversely affected by a decision of the Head of the Procurement Entity, and the complaint is submitted within twenty days after the issue of the decision by the Head of the Procurement Entity..

On receipt of a complaint, the PPB shall give notice of the complaint promptly to the Procurement Entity.

The PPB may:

- ❑ declare the legal rules or principles that govern the subject-matter of the complaint;
- ❑ order that the provisions of the Act be complied with;
- ❑ require the Procurement Entity that has acted or proceeded in an illegal manner, or that has reached an illegal decision, to act or to proceed in a legal manner or to reach a legal decision;
- ❑ annul in whole or in part an illegal act or decision of the Procurement Entity, other than any act or decision bringing the procurement contract into force;
- ❑ revise an illegal decision by the Procurement Entity or substitute its own decision for the decision, other than any decision bringing the procurement contract into force;
- ❑ require the payment of compensation for reasonable costs incurred by the supplier, contractor or consultant who submitted the complaint, in connection with the procurement proceedings as a result of an illegal decision of, or procedure followed by the Procurement Entity;
- ❑ order that the procurement proceedings be terminated;
- ❑ dismiss the complaint and require the payment of compensation for reasonable costs incurred by the Procurement Entity or the Public Procurement Board.

The Board shall, within twenty-one days of starting a review, issue a written decision concerning the complaint, stating the reasons for the decision.

8.1.3 Rules Applicable to Review Proceedings

The Head of the Procurement Entity or the Board shall notify the suppliers, contractors, or consultants participating in procurement proceedings about the submission of a complaint and of its substance within 14 working days after the submission of the complaint for review.

A supplier or contractor or any government authority whose interests are or could be affected by the review proceedings is entitled to participate in the review proceedings.

A supplier, contractor, or consultant who fails to participate in the review proceedings is barred from subsequently making the same type of claim.

A copy of the decision of the Head of the Procurement Entity or of the Board shall be furnished within five days after the issue of the decision to the supplier, contractor, or consultant submitting the complaint to the Procurement Entity and to any other supplier, contractor or government authority that has participated in the review proceedings.

After the decision has been taken, the complaint and the decision shall be promptly made available to the general public, but no information shall be disclosed if its disclosure would be contrary to law, would impede law enforcement, would not be in the public interest, would prejudice legitimate commercial interests of the parties or would inhibit fair competition.

8.1.4 Suspension of procurement proceedings

Where review proceedings are initiated, the procurement proceedings may be suspended for 7 days if the complaint:

- is not frivolous;
- contains a declaration which demonstrates that the supplier, contractor or consultant will suffer irreparable damage if the suspension is not granted; and
- is likely to succeed;

and the grant of the suspension will not cause disproportionate harm to the Procurement Entity or to other suppliers, contractors, or consultants.

When the procurement contract enters into force, upon the submission of a complaint, performance of the procurement contract shall be suspended for seven days, if the complaint meets the requirements set out above.

The Head of the Procurement Entity and the Board may extend the suspension in order to preserve the rights of the supplier, contractor, or consultant who is a party to the review pending the disposition of the review proceedings, but the total period of suspension shall not exceed thirty days.

The suspension provided for shall not apply if the Procurement Entity certifies that urgent public interest considerations requires the procurement to proceed.

The certification shall state the grounds for the finding that urgent considerations exist and shall be made a part of the record of the procurement proceedings, and it is conclusive with respect to administrative review.

Any decision by the Procurement Entity under this section and the grounds and circumstances shall be made part of the record of the procurement proceedings.

CHAPTER 9: GUIDELINES FOR DISPOSAL OF GOODS & EQUIPMENT

9.1 INTRODUCTION

Section 83 of the Public Procurement Act,(663) mandates the Head of Procurement Entity to dispose off stocks that become obsolete, redundant and unserviceable or surplus to requirement, in an orderly and systematic manner.

These Guidelines are designed to assist Procurement Entity to dispose off their surplus goods and equipment in an efficient, consistent, equitable and accountable manner. The guidelines provide practical information on how to manage the disposal process and select the most appropriate disposal option.

9.2 DEFINITIONS

The definitions of unserviceable, obsolete or surplus stores, plant and equipment shall be:

- ❑ Obsolete: any item of stores plant and equipment which is rendered incapable of further effective use by developments in technology, incompatibility with associated items, or where the annual maintenance and breakdown costs can be certified to exceed thirty percent (3 0%) of the estimated cost of a new replacement item.
- ❑ Unserviceable: any item of stores, plant and equipment which cannot be used for the intended purpose in its present condition due to major defects or damage, and is beyond economic repair. Classification as beyond economic repair for this purpose shall be determined on the basis that repair costs are certified to cost more than fifty percent (50%) of the current market price of a new replacement item.
- ❑ Surplus: any stores item which has not moved for a period in excess of two years, or any item of plant or equipment which has remained unused for a period in excess of one year, and where no potential use for the item can be envisaged within the Entity.

9.3 AUTHORITY TO DISPOSE

In accordance with the provisions of the Public Procurement Act, 2003 (Act 663):

- ❑ The Heads of the Procurement Unit shall arrange for periodical survey at quarterly interval of all stocks and equipment held by the Unit, to ascertain whether any item have become obsolete, unserviceable or surplus to requirement.
- ❑ The survey shall be undertaken by a team of at least three persons, comprising the following:

- o A representative from the Procurement Entity's Administration//Finance department.
- o The Storekeeper or Stockholder.
- o A senior officer from any public or private institution with special knowledge of the items to be surveyed shall be invited to act as a Technical Person to the committee.

- ❑ The reason for any item becoming surplus, obsolete and unserviceable shall be explained and recommendations submitted on the mode of disposal.
- ❑ The report of the survey shall be submitted to the Head of Procurement Entity. Upon receipt of the report of the survey, the Head of Entity shall instruct the Stores department to dispose off or supervise the disposal of the equipment or supplies in accordance with the recommendation of the survey team.
- ❑ The Board of Survey's recommendations shall be approved by the Head of Procurement Entity and the items shall be disposed of as approved.

9.4 DISPOSAL OPTIONS

The choice of the most appropriate disposal option will normally be influenced by the nature of stocks to be disposed, their location and market value.
The Items shall be disposed off in any of the following manner;

9.4.1 Transfer to Government Department or Other Public Entity.

Transfer to other government departments or other public entities with or without financial adjustment is applicable where; an asset can be usefully deployed by another procurement entity;

Actions:
The Storekeeper should:

- ❑ Liaise with the recipient entity for a formal transfer of ownership and removal of the items from the premises.

.

9.4.2 Sale by Public Tender.

Items with an initial cost of GHC 50million or more must be disposed of by Public tender, unless the item is more than 10 years old.
Sale by Public tender shall also be conducted where the estimated value of the asset, or group of asset packaged together, is of sufficient value to justify the cost of conducting a public tender.

Actions:
The Store keeper should:

- ❑ Request the procurement unit to initiate a formal tender

process which will include:

- o Preparation of tender document
- o Placement of Advertisement
- o Receipt, Opening and Evaluation of Tenders
- o And the items offered to the highest tenderer, subject to a reserve price.

9.4.3 Sale by Public Auction.

Disposal by public auction shall be conducted for items of that have an estimated value of less than GHC 50 million and also when sufficient items can be assembled for disposal to justify the costs of conducting the auction process.

Actions:
The Storekeeper should:

- ❑ Request the procurement unit to invite an Auctioneer to facilitate the auction of the items.
- ❑ Instructions relating to the sale should include timeframe for sale, target revenue, condition and location of assets, reserve price, and end-user restrictions. The instruction will constitute the authority for the Auctioneer to undertake the sale.

9.4.4 Destruction, Dumping or Burying

Disposal by destruction, dumping or burying shall be used where the asset has no residual value and cannot be converted into any other form which subsequently give it value.
To ensure that the destruction, dumping or burying is proper executed, it is recommended that a committee of at least three persons supervise the process.

Actions:
The Committee should:

- ❑ Obtain approval from the relevant Environmental/Health Agency to destroy, dump or bury the items.
- ❑ Should supervise the destruction, dumping or burial, at an appropriate place.

9.5 DETERMINATION OF MARKET VALUE OF ASSETS

- ❑ Valuation can play an important part in the effective and efficient disposal of goods as it provides an important reference point to help procurement entities select the most appropriate disposal option.
- ❑ The most accurate determination of value is always what the competitive market is prepared to pay. In addition to this, there are a number of agents who could provide procurement entities with expert valuations to ensure that the seller’s expectations from sales are realistic. It is suggested,

however, that entities only engage the services of an agent if the goods are of sufficient value.

- It is important to be aware that in some cases, valuers may apportion a value on a good that is based on their replacement cost (for insurance purposes). It should be recognized that this value will often be considerably more than what will eventually be realized at sale. Alternatively, valuers can claim that goods have nil book value after depreciation when in fact they could realize a considerable sum at sale. It is therefore recommended that entities identify an appropriate type of valuation and instruct the valuer accordingly.
- The basis for evaluation should always be market value taking into account that assets should be sold 'as is, where is' without warranties.

9.6 REVENUE FROM SALES

Proceeds from sales shall be paid into the consolidated fund or as may be directed by the Controller and Accountant General.

ANNEX 1

STANDARD PROCUREMENT FORMS

FORM SPF 1 - PROCUREMENT REQUISITION

FORM SPF 2 - SUBMISSION FOR TENDER COMMITTEE APPROVAL

FORM SPF 3 – EVALUATION REPORT (GOODS AND WORKS) ***– please refer to PPB website (www.ppbghana.org), Standard Tender Evaluation Format – Goods & Works.***

FORM SPF 4 – EVALUATION REPORT (SERVICES) -

please refer to PPB website (www.ppbghana.org), Standard Tender Evaluation Format – Selection of Consultants.

FORM SPF 5 - MONTHLY PROCUREMENT REPORT

ANNEX 2

FLOW CHARTS

FLOWCHART 1: SELECTION OF PROCUREMENT METHOD - GOODS

FLOWCHART 2: SELECTION OF PROCUREMENT METHOD - WORKS

ANNEX 3

1. **LOCAL PURCHASE ORDER**
2. **GENERAL CONDITIONS OF CONTRACT FOR LPOs**
