# Supplementary material for: A multi-center prospective cohort study to evaluate the effect of differential pricing and health systems strengthening on access to medicines and management of hypertension and diabetes in Ghana: A study protocol
Source: Gates Open Res. 2018 May 3;2:6. Originally published 2018 Feb 7. [Version 2] doi: 10.12688/gatesopenres.12797.2 (PMC5941285; doi:10.12688/gatesopenres.12797.2)
Supplement: Supplementary file 2 [file gatesopenres-2-13889-s0001.tgz › 410bf10f-812b-418c-b376-cb2c39f662dc.zip!/TREATMENT GUIDELINES/Hypertension_A5_edited final. Aug 12,2015.pdf]

ghana access and affordability program

GUIDELINES

FOR THE TREATMENT OF SYSTEMIC ARTERIAL HYPERTENSION

Ghana Access and Affordability Program (GAAP) Pilot Study

July, 2015

Table of Contents

Preface	ii
Purpose of the Guidelines	iii
Abbreviations	iv
Diagnosis of Hypertension	1
Definition	1
Blood Pressure Measurement	1
Measurement of Waist Circumference	3
Cardiovascular Risk Factors	4
Identification of High Risk Patients	5
Choice of Therapy	5
Recommendations for Titration of antihypertensive therapy	6
Compelling Indications for choice of specific anti-hypertensive agents	7
Compelling Contra-indications to use of specific anti-hypertensive agents	8
List of Anti-hypertensive Medicines	9
Algorithm for the Treatment of Hypertension	11
Annex 1: list of GAAP medicines	13

Preface

Type 2-Diabetes Mellitus, Hypertension and Cancers have emerged as health conditions of public health importance in Ghana, as in many other sub-Saharan African countries. One of the major constraints in the care of patients with these diseases is access to medicines that are of good quality, efficacious and affordable.

The Ghana Access and Affordability Program (GAAP) is a unique public-private initiative with the ultimate goal of improving the health outcomes of patients with non-communicable diseases, by improving access to life-saving medicines. A pilot research project, "The Effects of Differential Pricing and Health System Strengthening on the Management of Type II Diabetes Mellitus, Hypertension and Cancer in Ghana" has been designed to explore some of the key issues around access to medicines and disease outcome. The Pilot Study will be implemented in six health facilities; the Komfo Anokye Teaching Hospital, Agogo Presbyterian Hospital, Kings Medical Centre, Atua Government Hospital, Tamale Teaching Hospital and the Korle-Bu Teaching Hospital.

A team of medical experts with experience in the development of the National Treatment Guidelines and the Essential Medicines List, headed by the GAAP Clinical Coordinator, has developed treatment guidelines and algorithms for the management of Type II Diabetes Mellitus, Hypertension and selected cancers that prescribers will use at each of the Pilot Study sites. These guidelines are based on internationally accepted norms with modifications as related to local conditions and the experience of the experts.

The study has a health systems strengthening component that involves developing capacity for the clinical management of the selected conditions. These treatment guidelines and algorithms will, therefore, also serve as a tool for training prescribers and other health professional in the management of the selected health conditions.

Purpose of Guidelines

These guidelines are prepared for doctors participating in the Ghana Access and Affordability Program (GAAP) Pilot Study. The guidelines are provided to aid better decision making in the management of study participants diagnosed with hypertension in the GAAP Pilot Study.

The guidelines were prepared by:

Professor J. Plange-Rhule, Honorary Consultant Physician, Komfo Anokye Teaching Hospital, Kumasi

Dr. Isaac Owusu, Honorary Consultant Physician, Komfo Anokye Teaching Hospital, Kumasi

With inputs from

Dr. K. Agyenim-Boateng, Honorary Consultant Physician, Komfo Anokye Teaching Hospital, Kumasi

Dr. Francis Agyekum, Physician Specialist, Korle-Bu Teaching Hospital, Accra

Mrs. Amah Nkansah, Chief Pharmacist, Korle-Bu Teaching Hospital, Accra

Facilitators:

Professor David Ofori-Adjei, Clinical Coordinator, GAAP

Mrs. Lynda Arthur, Program Manager, GAAP

Dr. Fred Stephen Sarfo, Principal Investigator, GAAP

Ms. Sarah Asare, Program Officer, GAAP

GAAP Program Office

2nd Floor Yakeima Square,

No. 6 George Walker Bush Highway (NI), Dzorwulu,

ACCRA, GHANA

Tel: +233-302 734772/ 798045

Email: gaapgh@gmail.com

Abbreviations

**ACE-I**	ACE Inhibitor
**ARB**	Angiotensin Receptor Blocker
**BB**	Beta Blocker
**BMI**	Body Mass Index
**BP**	Blood Pressure
**CAD**	Coronary Artery Disease
**CCB**	Calcium Channel Blocker
**CKD**	Chronic Kidney Disease
**GFR**	Glomerular Filtration Rate
**LVH**	Left Ventricular Hypertrophy
**PAD**	Peripheral Artery Disease

Diagnosis of Hypertension

Definition

Hypertension is defined as a clinic systolic blood pressure ≥140mmHg and/or clinic diastolic blood pressure ≥90mmHg irrespective of age.

Blood Pressure Measurement

- Blood pressure will be measured with a validated automated digital sphygmomanometer (Omron Professional HEM907x1) and appropriate cuff size
- Allow the patient to sit for 3-5 minutes before blood pressure is measured.
- Take 3 blood pressure measurements on the Right Arm, 1-2 minutes apart at the first visit; discard the first measurement, and use the average of the last 2 blood pressure measurements to make diagnosis. (Take repeated measurements of Blood Pressure to improve accuracy in patients with atrial fibrillation)

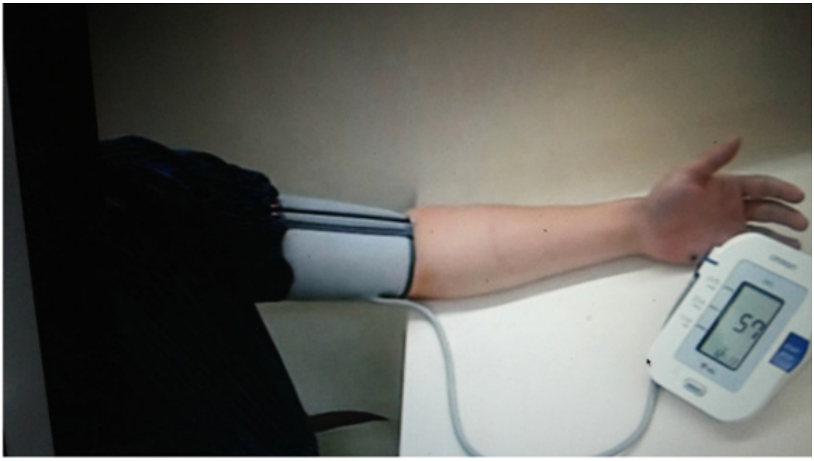

Measurement of Waist Circumference

- Use the illiac crest as the reference point
- Use non-stretcheable Gulick measuring tape
- Measurement should be done at the end of normal expiration
- Measure to the nearest millimetre.

Waist Circumference Measurement Guidelines - Self Measurement

Step 1

Place your in the following manner:

- Stand in front of a mirror
- Ensure your abdomen in unrestricted and clear
- Feet shoulder-width apart

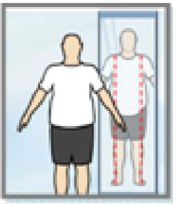

Man

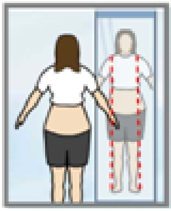

Woman

Step 2

- Wrap the measuring tape around your waist and insert the end of the tape into the appropriate slot.

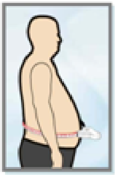

- Locate the uppermost border of your hipbones (iliac crest) on your right-hand side.

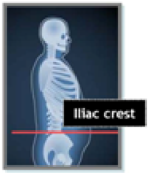

Step 3

- Align the bottom edge of the measuring tape with the top of your hipbones.

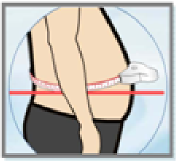

Step 4

- With the help of a mirror ensure that the tape is placed horizontally and wraps all around your abdomen.

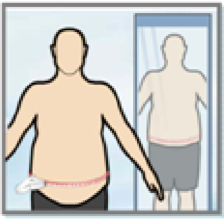

Step 5

- Before taking the measurement, take 2-3 NORMAL breaths.

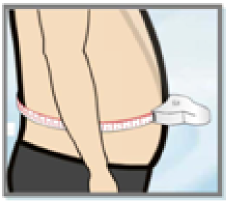

- At the end of the 3rd expiration, make a final adjustment by gently tightening the tape around your abdomen using the tape's central buttom.

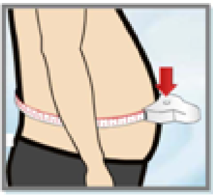

Step 6

- Take the measurement at the end of a NORMAL expiration.
- Before removing the tape, pinch the end of the measuring tape with your fingers closest to your measurement and hold it in position.

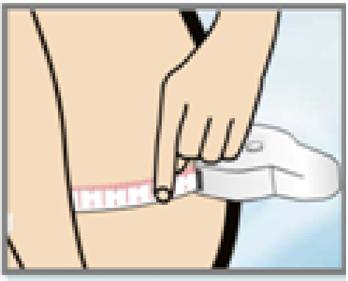

- Note the result.

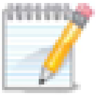

International Chair on Cardiometabolic Risk

www.cardiometabolic-risk.org

© 2009 International Chair on Cardiometabolic Risk. All rights reserved

Cardiovascular Risk Factors

The following features that may be determined from the history, physical examination and laboratory tests can influence the course and outcome of hypertension. They should be looked for in the assessment of the patient.

A. History

- Age (men ≥55 years; women ≥ 65)
- Family history of premature cardiovascular disease (men aged < 55 years; women aged < 65)
- Smoking
- Previous stroke or transient ischaemic attack
- Diabetes mellitus
- Heart failure
- Coronary artery disease

B. Physical Examination

- Obesity (BMI ≥30kg/m^2; Abdominal circumference ≥94cm for men, ≥80cm for women)
- Pulse pressure (in the elderly) ≥60mmHg
- Left ventricular hypertrophy
- Advanced retinopathy: haemorrhages or exudates, papilloedema
- Periperal arterial disease

C. Laboratory Findings

- Left Ventricular hypertrophy (by ECG voltage criteria)
- Left bundle branch block and other electrocardiographic features suggesitive of ischaemic heart disease
- Dyslipidaemia
- Hyperglycaemia (Diabetes Mellitus Type 2)
- Microalbuminuria and Proteinuria
- Reduced e-GFR

Identification of High Risk Patients

- Hypertensive patients (non-diabetic) with three or more risk factors
- Hypertensive patients with diabetes mellitus
- Hypertensive patients with subclinical target organ damage
- Hypertensive patients with established cardiovascular disease

Choice of Therapy

Choice of therapy should be guided by the profile of individual patients

A. Treatment Targets

- For the general population
  - < 140mmHg Systolic
  - < 90mmHg Diastolic
- Diabetes Mellitus and Chronic Kidney Disease patients
  - < 130mmHg systolic
  - < 80mmHg diastolic

B. Non-pharmacological Treatment (Lifestyle management)

- Regular physical exercise
- Weight reduction
- Dietary measures
  - Increased Fruits and Vegetables intake
  - Reduced Salt intake

C. Pharmacological Treatment

Antihypertensive Drugs

First line therapy for black patients should include thiazide diuretic or a CCB, however, any of the four classes of antihypertensives may be used depending on compellining indication or contraindication(s).

Recommendations for Titration of antihypertensive therapy

- Initiate and maintain antihypertensive treatment with any of the first-line drugs.
- In the general black population use thiazide diuretics or calcium channel blockers (CCB) either as monotherapy or in combination therapy.
- Do not use Angiotensin converting enzyme inhibitors (ACE-I) as a first-line drug for uncomplicated hypertension in black patients.
- In the general nonblack population, initial antihypertensive treatment should include a thiazide-type diuretic, calcium channel blocker (CCB), angiotensin-converting enzyme inhibitor (ACE-I), or angiotensin receptor blocker (ARB).
- If goal BP is not achieved with the initial drug, titrate the dose of the initial drug up to the maximum recommended dose to achieve goal BP[1].
- Start dual therapy when blood pressure ≥180/110mmHg
- If goal BP is not achieved with the use of one drug after titration to the maximum recommended dose, add a second drug from the list (thiazide-type diuretic, CCB, ACE-I, or ARB) and titrate up to the maximum recommended dose of the second drug to achieve goal BP .
- Add other anti-hypertensive drugs (such as centrally-agents and/or vasodilators) if target blood pressure is still not achieved with a combination of two first-line drugs.
- If goal BP is not achieved with 2 drugs, select a third drug from the list (thiazide-type diuretic, CCB, ACE-I, or ARB), avoiding the combined use of ACE-I and ARB. Titrate the third drug up to the maximum recommended dose to achieve goal BP
- Refer to an expert if blood pressure remains uncontrolled with the optimal or maximum tolerated doses of three drugs.

---

[1] Do not combine an ACE-I and an angiotensin receptor blocker (ARB) to treat hypertension.

Compelling Indications for choice of specific anti-hypertensive agents

Indication	Goal BP	Initial Drug Treatment Option
General	<140/90	Thiazide diuretic, CCB
Diabetes	<130/80	ACE-I or ARB
Chronic Kidney Disease (CKD) (with and without proteinuria)	<140/90	ACE-I or ARB<br>Avoid if eGFR <15min/ml without renal replacement therapy
Left Venticular Hypertrophy (LVH)	<140/90	ACE-I, ARB, CCB (preferably Amlodipine)
Previous Stroke	<140/90	Thiazide diuretic, CCB
Heart Failure	<140/90	ACE-I/ARB; Cardio-selective B-Blockers - Bisoprolol, Metoprolol, Carvedilol; Loop diuretics (Add Spironelactone in advanced heart failure.
Coronary Artery Disease (CAD)	<140/90	ACE-I or ARB, Beta-blocker, CCB.
Peripheral Artery Disease (PAD)	<140/90	CCB and ACE-I/ARB
Atrial fibrillation	<140/90	ARB/ACE-I, B-blockers

Compelling Contra-indications to use of specific anti-hypertensive agents

In the presence of any of the clinical conditions in this section the specified anti-hypertensives should not be given to the patient.

- Gout - Thiazide diuretics
- Asthma, 2^0 and 3^0 A-V block - Beta-blockers
- Heart failure – CCB
- Bilateral renal artery stenosis and hyperkalaemia - ACE-I and ARB

High Risk Patients

- Initiate statin therapy in hypertensive patients with high cardiovascualar risk profile (irrespective of lipid levels) in addition to the anti-hypertensive therapy.
- Add antiplatelet therapy (low dose aspirin) to antihypertensive drugs in hypertensive patients with a previous cardiovascular event.

Resistant Hypertension

- Make this diagnosis after adequate doses of at least 3 medications (including a thiazide diuretic and a CCB) with good adherence.
- Refer patients to a specialist for further evaluation and management

List of Anti-hypertensive Medicines

Therapeutic Class	Name of Medicine & Strength	Initial Dose (mg)	Dosing Range	Route of Adminis-tration	Frequency of Adminis-tration
ACE-I	Lisinopril	10	10mg – 40mg	PO	Once daily
ACE-I/Thi-azide	Lisinopril/ HCTZ	10/12.5	10/12.5, 20/12.5 mg	PO	Once daily
Alpha-agonist, central	Methyldopa	250	250 mg initial dose<br>500mg to 1g as maintenance dose	PO	Twice or thrice daily
Alpha-blocker, peripheral	Prazosin	0.5	May be slowly increased up to a total daily dose of 20mg in divided doses	PO	Twice or thrice daily
ARB	Ibersartan	75	75mg – 300mg	PO	Once daily
ARB	Losartan	25	50mg-100mg	PO	Once daily
ARB/Thiazide	Ibersartan / HCTZ	150	150mg/12.5mg, 300mg/12.5mg	PO	Once daily
ARB/Thiazide	Losartan/ HCTZ	50/12.5	50mg/12.5mg	PO	Once daily
ARB/Thiazide	Losartan/ HCTZ	100/25	100mg/25mg	PO	Once Daily
Beta-blocker	Atenolol	25	25 – 50mg, 100 mg	PO	Once daily
Beta-blocker	Labetalol	100	200 – 400 mg as maintenance dose	PO	Twice daily
Beta-blocker	Ramipril	2.5	2.5mg - 20 mg	PO	Once daily
Beta-blocker/ Thiazide	Atenolol/ HCTZ	50/12.5	50mg/12.5mg	PO	Once daily
CCB	Amlodipine besylate	5	5mg – 10mg	PO	Once daily
CCB	Nifedipine	10	10 - 20 mg	PO	Twice daily
CCB	Nifedipine slow release	10	10-60 mg	PO	Once or twice daily
CCB	Nifedipine GITS	30	30mg – 60mg	PO	Once daily

Therapeutic Class	Name of Medicine & Strength	Initial Dose (mg)	Dosing Range	Route of Adminis-tration	Frequency of Adminis-tration
CCB	Verapamil	40	240 - 360 mg	PO	Thrice daily when used as monotherapy for hypertension
CCB/Statin	Amlodipine besylate/ Atorvastatin calcium	5/20	5mg/10mg – 5mg/20mg; maxi-mum 10mg/20mg	PO	Once daily
Thiazide	Bendroflu-methiazide	2.5	2.5mg - 5 mg	PO	Once daily
Vasodilator	Hydralazine	10 or 20	10mg - 20 mg	INJ	2-4 hourly
Vasodilator	Hydralazine	10 or 25	10mg-50mg	PO	Four times daily

- When initiating a new antihypertensive or adding a new antihypertensive to an already existing regimen, start by titrating dose and frequency of administration that avoid precipitous drops in blood pressure
- Please consult a pharmacist for guidance on dosing and frequency of administration when unsure.

ALGORITHM FOR THE TREATMENT OF HYPERTENSION

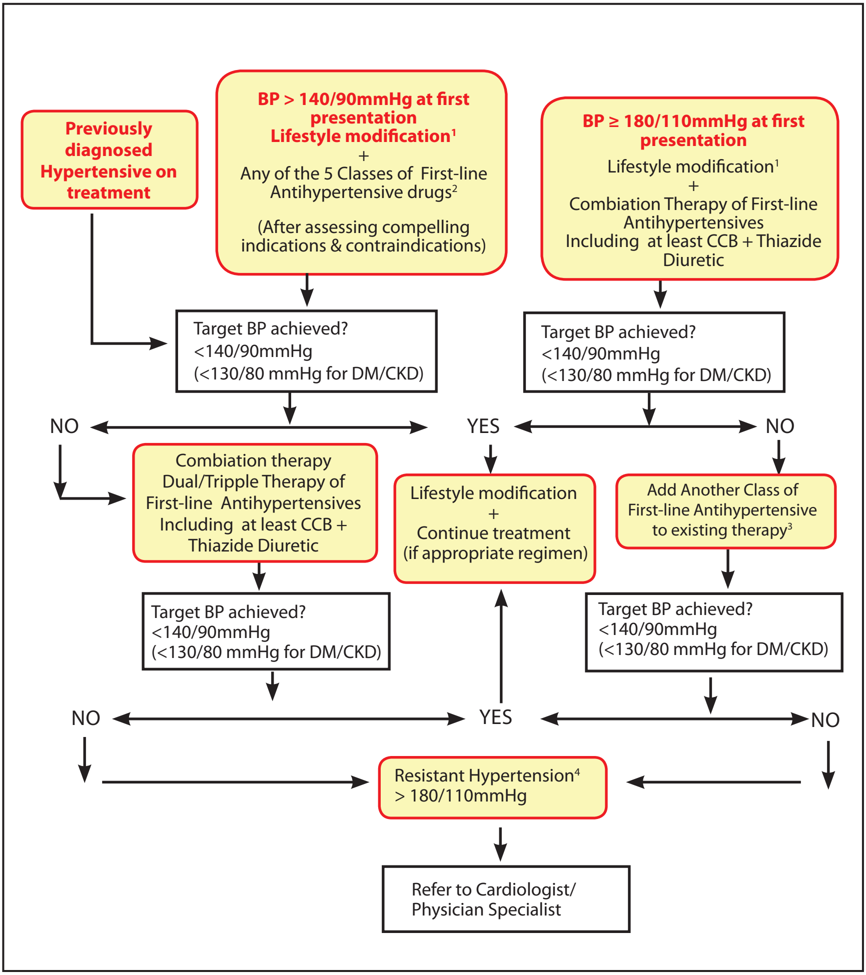

1. Life style – low salt, weight loss, exercise, eat more fruits and vegetables
2. 5 classes of antihypertensive medications- Thiazide, CCB, ACE-I ARB, Beta-blockers; preferably CCB ± Thiazide diuretics
3. ACE-I and ARB should not be combined
4. Consider centrally acting agents (Methyldopa), vasodilators (Hydrallazine). Investigate for secondary causes of hypertension

Compelling Indications

- **LVH** – ACE-I, ARB, CCB preferably Amlodipine
- **Microalbuminuria –** ACE-I, ARB
- **Renal dysfunction** – ACE -I ARB[1]
- **Previous stroke**: Any agent
- **Angina:** B-blocker, CCB preferably verapamil
- **Heart failure:** ACE-I/ARB, Cadio-selective B-Blockers[2]; Diuretics - loop durectics plus Spironolactone
- **Peripheral artery disease:** CCB and ACE-I/ARB
- **DM:** ACE-I /ARB
- **Atrial fibrillation** – ARB /ACE-I, B-blockers

[1]Caution: if eGFR<15min/ml without renal replacement therapy
[2]Cardioselective beta blockers such as bisoprolol, metoprolol, carvedilol

Compelling Contraindications

- **Thiazide diuretics** – Gout
- **Beta Blockers** – Asthma, 2⁰ and 3⁰ AV Block
- **CCB** – Heart Failure
- **ACE-I and ARB** – Bilaterial renal artery stenosis and hyperkalaemia

Patients with High risk for Cardiovascular disease

In addition to antihypertensive additional treament should include

- **Statins** irrespective of serum lipids levels &
- **Antiplatelet therapy**

1. Age > 55yr, LVH, LBBB, Smoking, dyslipidaemia, microalbuminuria, diabetes mellitus, CKD

FACTORS USED FOR STRATIFICATION OF TOTAL CARDIOVASCULAR (CV) RISK

RISK FACTORS

1. Male
2. Age (men 55y; women 65y)
3. Smoking
4. Dyslipidaemia
5. Fasting blood glucose 5.6-6.9mmol/l
6. Obesity
7. Family History of premature CVD

ASYMPTOMATIC ORGAN DAMAGE

1. Pulse pressure >60 mmHg
2. Electrocardiographic LVH
3. Ankle-brachial index <0.9
4. CKD with eGFR 30-60ml/min
5. Microalbuminuria

ESTABLISHED CV OR RENAL DISEASE

1. Stroke /TIA
2. Coronary artery disease
3. Heart failure
4. Symptomatic lower extremities peripheral artery disease
5. CKD with eGFR <30mL/min
6. Advanced retinopathy

ANNEX 1
MEDICINES FOR THE GAAP PILOT STUDY

PRODUCT	MOLECULES	INDICATION
Adriblastina 10MG, 1 VIAL	Doxorubicin	Oncology Drug
Adriblastina 50MG, 1 VIAL	Doxorubicin	Oncology Drug
Aprovel 150MG TABS x28	Ibersartan	Cardiovascular Drug
Aprovel 300MG TABS x 28	Ibersartan	Cardiovascular Drug
Aromasin 25MG TABS 30's	Exemestane	Oncology Drug
Caduet 5/10MG TABS x 30	Amlodipine Besylate/ Atorvastatin Calcium	Cardiovascular Drug
Caduet 10/10MGTABS x 30	Amlodipine Besylate/ Atorvastatin Calcium	Cardiovascular Drug
Co-Aprovel 150/12.5MG TABS x28	Ibersartan/HCTZ	Cardiovascular Drug
Co-Aprovel 300/12.5MG TABS x28	Ibersartan/HCTZ	Cardiovascular Drug
Cozaar 50MG TABS x30	Losartan	Cardiovascular Drug
Cozaar Comp 50MG/12.5MG TABS x30	Losartan/HCTZ	Cardiovascular Drug
Eloxatin 50MG, 1 VIAL	Oxaliplatin	Oncology Drug
Eloxatin 100MG, 1VIAL	Oxaliplatin	Oncology Drug
Fortzaar 100MG/25MG TABS x30	Losartan/HCTZ	Cardiovascular Drug
Janumet 50/850MG TABS x56	Metformin/Sitagliptin	Diabetic Drug
Janumet 50/1000MG TABS x 56	Metformin/Sitagliptin	Diabetic Drug
Januvia 25MG TABS x28	Sitagliptin	Diabetic Drug
Januvia 50MG TABS x28	Sitagliptin	Diabetic Drug
Januvia 100MG TABS x 28	Sitagliptin	Diabetic Drug
Lantus 3*5 (5 vials in a pack)	Insulin Glargine	Diabetic Drug
Taxotere 20MG/0.5ML 1 VIAL	Docetaxel	Oncology Drug
Taxotere 80MG/2ML 1 VIAL	Docetaxel	Oncology Drug

Ghana Access And Affordability Program Pilot Study

© 2015
