# Supplementary material for: A multi-center prospective cohort study to evaluate the effect of differential pricing and health systems strengthening on access to medicines and management of hypertension and diabetes in Ghana: A study protocol
Source: Gates Open Res. 2018 May 3;2:6. Originally published 2018 Feb 7. [Version 2] doi: 10.12688/gatesopenres.12797.2 (PMC5941285; doi:10.12688/gatesopenres.12797.2)
Supplement: Supplementary file 2 [file gatesopenres-2-13889-s0001.tgz › 410bf10f-812b-418c-b376-cb2c39f662dc.zip!/TREATMENT GUIDELINES/TYPE-2 DIABETES_final.pdf]

ghana access and affordability program

GUIDELINES

FOR THE MANAGEMENT OF TYPE-2 DIABETES MELLITUS (T2DM)

Ghana Access and Affordability Program (GAAP) Pilot Study

July, 2015

Table of Contents

Preface	ii
Purpose of the Guidelines	iii
Abbreviations	iv
Diagnosis of Type 2 Diabetes Mellitus	1
Definition	1
Measurements	1
Risk factors	1
Choice of Therapy	2
List of Anti-diabetic Medications	6
Treatment Algorithm for Type 2 Diabetes Mellitus	7
Annex 1: list of GAAP medicines	8

Preface

Type 2-Diabetes Mellitus, Hypertension and Cancers have emerged as health conditions of public health importance in Ghana, as in many other sub-Saharan African countries. One of the major constraints in the care of patients with these diseases is access to medicines that are of good quality, efficacious and affordable.

The Ghana Access and Affordability Program (GAAP) is a unique public-private initiative with the ultimate goal of improving the health outcomes of patients with non-communicable diseases, by improving access to life-saving medicines. A pilot research project, "The Effects of Differential Pricing and Health System Strengthening on the Management of Type II Diabetes Mellitus, Hypertension and Cancer in Ghana" has been designed to explore some of the key issues around access to medicines and disease outcome. The Pilot Study will be implemented in six health facilities; the Komfo Anokye Teaching Hospital, Agogo Presbyterian Hospital, Kings Medical Centre, Atua Government Hospital, Tamale Teaching Hospital and the Korle-Bu Teaching Hospital.

A team of medical experts with experience in the development of the National Treatment Guidelines and the Essential Medicines List, headed by the GAAP Clinical Coordinator, has developed treatment guidelines and algorithms for the management of Type II Diabetes Mellitus, Hypertension and selected cancers that prescribers will use at each of the Pilot Study sites. These guidelines are based on internationally accepted norms with modifications as related to local conditions and the experience of the experts.

The study has a health systems strengthening component that involves developing capacity for the clinical management of the selected conditions. These treatment guidelines and algorithms will, therefore, also serve as a tool for training prescribers and other health professional in the management of the selected health conditions.

Purpose of Guidelines

These guidelines are prepared for doctors participating in the Ghana Access and Affordability Program (GAAP) Pilot Study. The guidelines are provided to aid better decision making in the management of study participants diagnosed with hypertension in the GAAP Pilot Study.

The guidelines were prepared by:

Professor J. Plange-Rhule, Honorary Consultant Physician, Komfo Anokye Teaching Hospital, Kumasi

Dr. Isaac Owusu, Honorary Consultant Physician, Komfo Anokye Teaching Hospital, Kumasi

With inputs from

Dr. K. Agyenim-Boateng, Honorary Consultant Physician, Komfo Anokye Teaching Hospital, Kumasi

Dr. Francis Agyekum, Physician Specialist, Korle-Bu Teaching Hospital, Accra

Mrs. Amah Nkansah, Deputy Director, Pharmaceutical Services, Korle-Bu Teaching Hospital, Accra

Mr. Philip Anum, Rector, Ghana College of Pharmacist

Facilitators:

Professor David Ofori-Adjei, Clinical Coordinator, GAAP

Mrs. Lynda Arthur, Program Manager, GAAP

Dr. Fred Stephen Sarfo, Principal Investigator, GAAP

Ms. Sarah Asare, Program Officer, GAAP

GAAP Program Office

2nd Floor Yakeima Square,

No. 6 George Walker Bush Highway (NI), Dzorwulu,

ACCRA, GHANA

Tel: +233-302 734772/ 798045

Email: gaapgh@gmail.com

Abbreviations

**ACR**	Albumin-Creatinine Ratio
**CHD**	Coronary Heart Disease
**DKA**	Diabetes Ketoacidosis
**DPP-4**	Dipeptidyl Peptidase-4 Inhibitors
**HbA1C**	Hemoglobin A1C
**PAD**	Peripheral Artery Disease
**SU**	Sulfonylurea
**TZD**	Thiazolidinedione
**T2DM**	Type 2 Diabetes Mellitus

Diagnosis of T2DM

Definition

Type 2 diabetes mellitus is characterized by hyperglycaemia resulting from a combination of resistance to insulin action, inadequate insulin secretion, and excessive or inappropriate glucagon secretion.

Poorly controlled T2DM is associated with an array of micro vascular (retinal, renal), macro vascular (coronary artery disease, cerebrovascular and peripheral vascular disease), and neuropathic complications (autonomic and peripheral nerves).

Measurements

Any one of the following test results is diagnostic of T2DM:

1. Random Blood Sugar (RBS) of 11.1mmol/l or more with typical symptoms[1] on one occasion or with hyperglycaemic crisis[2] .
2. Fasting[3] Blood Sugar (FBS) >7.0mmol/l on 2 or more occasions in asymptomatic patients or one FBS >7.0mmol/l with typical symptoms
3. RBS taken 2 hours after 75g glucose load is 11.1mmol/L or more.
4. HbA1c of 6.5% or more

Risk factors

The following features that may be determined from the history, physical examination and laboratory tests can influence the course of T2DM. They should be looked for in the assessment of the patient.

[1] Typical symptoms: polyuria, polydipsia, polyphagia, unexplained weight loss
[2] Hyperglycaemic crisis: diabetic ketoacidosis (DKA), hyperosmolar hyperglycaemic state (HHS)
[3]Fasting: no caloric intake (e.g. food, beverage) for at least 8 hours

A. History

Age
Duration of DM
Personal and/or Family history of CVD
Hypertension
Smoking
Obesity
Physical inactivity

B. Physical Examination

In addition to initial general examination it is essential to examine the optic fundi, tendon reflexes, peripheral sensation and measure blood pressure (sitting and standing).

C. Laboratory Investigations

Urine ketones
Spot urine ACR
Blood urea, electrolytes and creatinine
Fasting blood lipid profile
Glycated haemoglobin (HbA1C)
Full Blood Count
ECG

Choice of Therapy

It is recommended that the choice of therapy should be individualized based on identified risk factors

A. Treatment Targets

The targets of treatment provided below are based on approved guidelines of various established studies or international associations.

1. HbA1C <7.0% except for:
    i. Older people (>60 years) where <8.0% less stringent.
    ii. Healthy high-risk patients (>6.5% but <6.0%)
2. Fasting blood glucose between (5.0 – 7.0) mmol/L

3. 2-hour post-meal blood below 10 mmol/L
4. Blood pressure less than 130/80 mmHg
5. LDL-cholesterol less than 2.5 mmol/L (<1.8 in high risk patients)

B. Non-pharmacologic Treatment

1. Diet therapy

All patients with diabetes require diet therapy.

All patients (and close relations who cook or control their meals) must be referred to a dietician or diet nurse for individualized meal plans.

In general, patients must;

- Avoid refined sugars as in soft drinks, or adding sugar to their beverages.
- Be encouraged to have complex carbohydrates (e.g. kenkey, yam, plantain etc.).
- Have a reduced total caloric content (portions) and an increase in the amount of fibre e.g. vegetables, fruits and cereals.
- Restrict salt intake especially at table

2. Regular exercise

- Regular, simple exercise at least 150mins/week in ambulant patients spread through the week with no more than 2 consecutive days break (Diabetes care 2013)
- Advice on exercise must consider the patient's age and the presence of complications of diabetes and other medical co-morbidities

3. Weight reduction

- Individualised interventions to promote weight loss (including lifestyle, pharmacological or surgical interventions) should be offered to obese adults with type 2 diabetes in order to improve metabolic control.

4. **Moderation of alcohol use**

5. **Salt restriction**

6. **Quit smoking:** Smoking cessation reduces risk of cardiovascular events, including coronary heart disease (CHD), stroke, peripheral arterial disease (PAD) and cardiovascular death.
   - ➲ Healthcare professional involved in the care of people with diabetes mellitus should advise them not to smoke. Those who smoke cigarette but wish to stop should be offered intensive management plus pharmacological therapies.
   - ➲ Patients who have stopped smoking should be monitored by their health care professionals to prevent relapse back into smoking.

C. Pharmacologic Therapy

1. Give Metformin as first line treatment unless there are contraindications (e.g., Acute illness, eGFR <30ml/min/m^2)
2. If Metformin is contraindicated or not tolerated give any of the following as initial therapy:
   i. Sulphonylureas (SU) - Glimepiride, Gliclazide preferred in older people; caution with Glibenclamide in elderly
   ii. Thiazolidinediones (TZD) – Pioglitazone
   iii. Dipeptidyl peptidase - IV (DPP-4) inhibitors-Sitagliptin, Vildagliptin
   iv. Insulins (Insulin Glargine, premixed, short acting insulin)
3. For patients not on target after maximal tolerable doses of initial therapy, add any of the medicines in 2i-iv above as appropriate.

NOTE

- ➲ Early initiation of pharmacologic therapy is associated with improved glycaemic control and reduced long-term complications in T2DM (metabolic memory or legacy effect)
- ➲ Individuals with Type 2 diabetes not responding to maximum

tolerable doses of metformin, sulphonylureas, DPP4 inhibitor or a thiazolidinedione alone, after 3-6 months, could be given combined oral therapy with two or three oral anti-diabetic medications. However, two different products from the same group should never be used together. Fixed dose combinations with metformin may be used.

➲ Insulin therapy should be initiated in patients with type 2 diabetes and an initial HbA1C >9 percent, or if the diabetes is uncontrolled despite optimal oral glycemic therapy after 2-3 months. Metformin combined with insulin is also associated with decreased weight gain, a lower insulin dosage, and less hypoglycemia compared with insulin alone.

➲ Insulin administered for day-to-day diabetes treatment is given subcutaneously, 30 minutes before a meal according to either of the following regimens;
  - Twice daily pre-mixed insulin before breakfast and dinner
  - Three pre-meal regular insulin injections before breakfast, lunch and dinner PLUS long-acting insulin at bedtime

➲ Insulin therapy may be initiated as augmentation, starting at 0.3 unit per kg, or as replacement, starting at 0.6 to 1.0 unit per kg. Augmentation therapy can include basal or bolus insulin.

➲ When using replacement therapy, 50 percent of the total daily insulin dose is given as basal, and 50 percent as bolus, divided up before breakfast, lunch, and dinner. Replacement therapy includes basal-bolus insulin and correction or premixed insulin.

➲ Statin and low dose aspirin therapy should be prescribed for patients above 40 years and/or with additional risk factors.

List of Anti-diabetic Medications

Therapeutic Class	Name of Medicine and Strength	Route of Adminis-tration	Frequency of Adminis-tration	Dosing Range
Biguanide	Metformin 500mg	PO	Twice daily	500mg – 1000mg
DPP-4	Sitagliptin 100mg	PO	Once daily	100mg
DPP-4/Biguanide**	Sitagliptin + Met-formin 50mg +500mg;<br><br>50mg+1000mg	PO	Twice daily	50mg/500mg<br><br>50mg/500mg – 50mg/1000mg
Insulin	Insulin Glargine 100units/ml	Subcut Inj	Once daily	Individualized; Start 10 units per dose; increase dose by 1 unit until FPG <6.0
Sulfonylurea (SU)	Glibenclamide 5mg	PO	Once or twice daily	Up to 15mg max is advised
Sulfonylurea (SU)	Gliclazide 80mg	PO	Once daily	80mg -320mg; doses above 160mg should be taken twice daily
Sulfonylurea (SU)	Glimepiride 1mg, 2mg, 4mg	PO	Once daily	Start 1-2mg for1-2 wks; max 8mg daily
Sulfonylurea (SU)	Tolbutamide 500mg	PO	Once or twice daily	1-2gm/day; maximum 3gm/day
Thiazolidinedione (TZD)	Pioglitazone 15mg, 30mg, 45mg	PO	Once daily	15mg – 30mg; maximum dose 45mg

** For patients inadequately controlled on maximal tolerated dose of metformin monotherapy: For patients not adequately controlled on metformin alone, the usual starting dose should be sitagliptin dosed as 50 mg twice daily (100 mg total daily dose) plus the dose of metformin already being taken. Sitagliptin should not be used in patients with moderate or severe renal impairment (creatinine clearance < 60 ml/min)

Treatment Algorithm for Type 2 Diabetes Mellitus

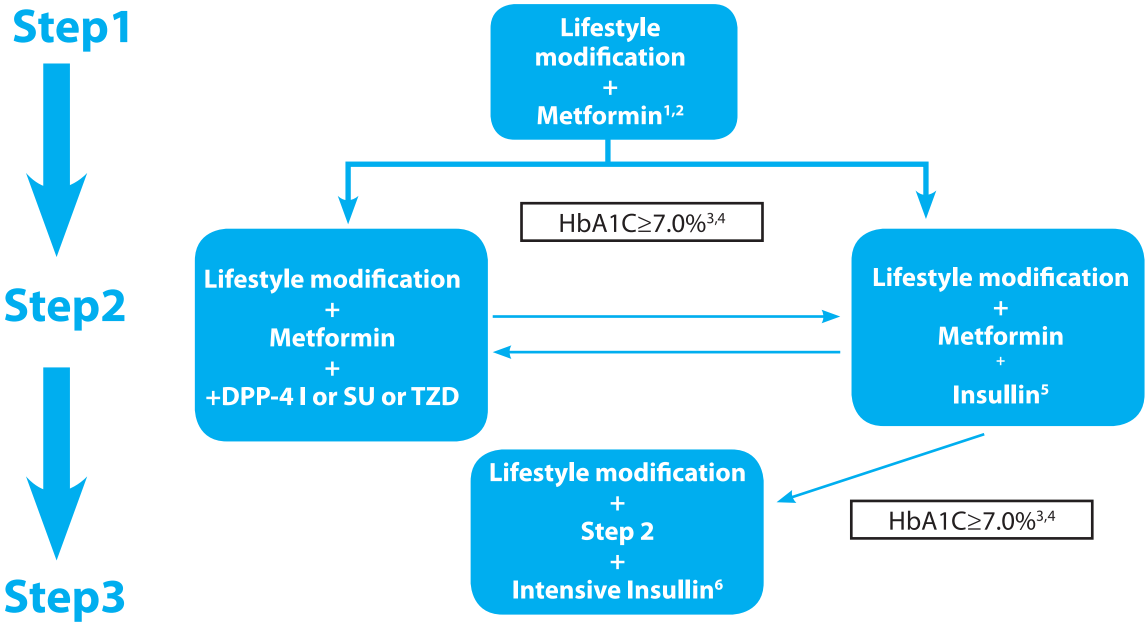

1. For highly motivated patients with HBA1C≥7.5% , a 3-6 month trial of lifesyle modification only before initiation of metformin
2. If Metform is C1 SU or TZD or DPP4-1 may be used as initial therapy.
3. Change after 3-6 months or regimen.
4. For High risk group HBA1C target of 6.5%
5. Use Metformin + Long-acting Insulin or pre-mixed Insulin if HbA1C>8.5%
6. Intensive Insulin - addition of meal time short acting insulin

ghana access and affordability program

ANNEX 1
MEDICINES FOR THE GAAP PILOT STUDY

PRODUCT	MOLECULES	INDICATION
Adriblastina 10MG, 1 VIAL	Doxorubicin	Oncology Drug
Adriblastina 50MG, 1 VIAL	Doxorubicin	Oncology Drug
Aprovel 150MG TABS x28	Ibersartan	Cardiovascular Drug
Aprovel 300MG TABS x 28	Ibersartan	Cardiovascular Drug
Aromasin 25MG TABS 30's	Exemestane	Oncology Drug
Caduet 5/10MG TABS x 30	Amlodipine Besylate/ Atorvastatin Calcium	Cardiovascular Drug
Caduet 10/10MGTABS x 30	Amlodipine Besylate/ Atorvastatin Calcium	Cardiovascular Drug
Co-Aprovel 150/12.5MG TABS x28	Ibersartan/HCTZ	Cardiovascular Drug
Co-Aprovel 300/12.5MG TABS x28	Ibersartan/HCTZ	Cardiovascular Drug
Cozaar 50MG TABS x30	Losartan	Cardiovascular Drug
Cozaar Comp 50MG/12.5MG TABS x30	Losartan/HCTZ	Cardiovascular Drug
Eloxatin 50MG, 1 VIAL	Oxaliplatin	Oncology Drug
Eloxatin 100MG, 1VIAL	Oxaliplatin	Oncology Drug
Fortzaar 100MG/25MG TABS x30	Losartan/HCTZ	Cardiovascular Drug
Janumet 50/850MG TABS x56	Metformin/Sitagliptin	Diabetic Drug
Janumet 50/1000MG TABS x 56	Metformin/Sitagliptin	Diabetic Drug
Januvia 25MG TABS x28	Sitagliptin	Diabetic Drug
Januvia 50MG TABS x28	Sitagliptin	Diabetic Drug
Januvia 100MG TABS x 28	Sitagliptin	Diabetic Drug
Lantus 3*5 (5 vials in a pack)	Insulin Glargine	Diabetic Drug
Taxotere 20MG/0.5ML 1 VIAL	Docetaxel	Oncology Drug
Taxotere 80MG/2ML 1 VIAL	Docetaxel	Oncology Drug

Ghana Access And Affordability Program Pilot Study

© 2015
