# Supplementary material for: A multi-center prospective cohort study to evaluate the effect of differential pricing and health systems strengthening on access to medicines and management of hypertension and diabetes in Ghana: A study protocol
Source: Gates Open Res. 2018 May 3;2:6. Originally published 2018 Feb 7. [Version 2] doi: 10.12688/gatesopenres.12797.2 (PMC5941285; doi:10.12688/gatesopenres.12797.2)
Supplement: Supplementary file 3 [file gatesopenres-2-13889-s0002.tgz › 4dd10987-e8ed-44b0-80bd-b229fe524c9f.zip!/Supplementary File 3 - Data collection tools/GAAP Clinical Data FORM.docx]

**GAAP Clinical Data**

| Location and Date | Response | | Code |  |
| --- | --- | --- | --- | --- |
| Name of physician/prescriber |  | |  | |
| Institution/ Facility ID |  |  | |  |
| Patient Name |  | |  | |
| Date of visit | [____]/[____]/[____]  **dd mm yyyy** | |  | |

*To extract from chart at time of enrollment and during each follow-up visit*

A. Vital Signs

|  | Baseline | 2 months | 4 months | 6 months | 8 months | 10 months | 12 months | 14 months | 16 months | 18 months |
| --- | --- | --- | --- | --- | --- | --- | --- | --- | --- | --- |
| Height (m) |  |  |  |  |  |  |  |  |  |  |
| Weight (kg) |  |  |  |  |  |  |  |  |  |  |
| Systolic blood pressure (mm Hg) |  |  |  |  |  |  |  |  |  |  |
| Diastolic Blood Pressure (mm Hg) |  |  |  |  |  |  |  |  |  |  |
| Pulse (beats/min) |  |  |  |  |  |  |  |  |  |  |

Medications at (for Diabetes or Hypertension)

Medications at time of enrollment

| Medication | Dose, frequency | Date started | Date discontinued |
| --- | --- | --- | --- |
|  |  |  |  |
|  |  |  |  |
|  |  |  |  |
|  |  |  |  |
|  |  |  |  |
|  |  |  |  |
|  |  |  |  |
|  |  |  |  |
|  |  |  |  |

Medication changes made during follow-up visits

| Medication | Dose, frequency | Date started | Date discontinued | Please place a “X” if this medicine is on the DP list |
| --- | --- | --- | --- | --- |
|  |  |  |  |  |
|  |  |  |  |  |
|  |  |  |  |  |
|  |  |  |  |  |
|  |  |  |  |  |
|  |  |  |  |  |
|  |  |  |  |  |
|  |  |  |  |  |
|  |  |  |  |  |

B. Laboratory Data

|  | Baseline | 2 months | 4 months | 6 months | 8 months | 10 months | 12 months | 14 months | 16 months | 18 months |
| --- | --- | --- | --- | --- | --- | --- | --- | --- | --- | --- |
| Fasting Blood Sugar  (mmol/l) |  |  |  |  |  |  |  |  |  |  |
| HbA1C (%) |  |  |  |  |  |  |  |  |  |  |
| Total Cholesterol  (mmol/l) |  |  |  |  |  |  |  |  |  |  |
| HDL  (mmol/l) |  |  |  |  |  |  |  |  |  |  |
| LDL  (mmol/l) |  |  |  |  |  |  |  |  |  |  |
| Triglycerides  (mmol/l) |  |  |  |  |  |  |  |  |  |  |
| Creatinine (umol/l) |  |  |  |  |  |  |  |  |  |  |
| blood urea  (mmol/l) |  |  |  |  |  |  |  |  |  |  |
| Urine protein |  |  |  |  |  |  |  |  |  |  |

C. Complications

|  | Baseline | 2 months | 4 months | 6 months | 8 months | 10 months | 12 months | 14 months | 16 months | 18 months |
| --- | --- | --- | --- | --- | --- | --- | --- | --- | --- | --- |
| Peripheral Neuropathy |  |  |  |  |  |  |  |  |  |  |
| Amputations |  |  |  |  |  |  |  |  |  |  |
| CVD events* |  |  |  |  |  |  |  |  |  |  |
| CVA |  |  |  |  |  |  |  |  |  |  |
| Ulcers |  |  |  |  |  |  |  |  |  |  |
| Renal failure |  |  |  |  |  |  |  |  |  |  |
| Diabetic foot infection |  |  |  |  |  |  |  |  |  |  |
| Peripheral Neuropathy |  |  |  |  |  |  |  |  |  |  |
| Amputations |  |  |  |  |  |  |  |  |  |  |
| Other |  |  |  |  |  |  |  |  |  |  |

* CVD events: acute MI, unstable angina, cardiac mortality

D. Hospitalizations

| Date | Location | Reason for hospitalization |
| --- | --- | --- |
|  |  |  |
|  |  |  |
|  |  |  |
|  |  |  |
|  |  |  |
|  |  |  |
|  |  |  |
|  |  |  |
|  |  |  |
