# Supplementary material for: A multi-center prospective cohort study to evaluate the effect of differential pricing and health systems strengthening on access to medicines and management of hypertension and diabetes in Ghana: A study protocol
Source: Gates Open Res. 2018 May 3;2:6. Originally published 2018 Feb 7. [Version 2] doi: 10.12688/gatesopenres.12797.2 (PMC5941285; doi:10.12688/gatesopenres.12797.2)
Supplement: Supplementary file 3 [file gatesopenres-2-13889-s0002.tgz › 4dd10987-e8ed-44b0-80bd-b229fe524c9f.zip!/Supplementary File 3 - Data collection tools/GAAP DISPENSING REGISTER OCT 1.docx]

**GAAP DISPENSING REGISTER**

| Date | Patient Name | Patient ID number | Medicines Prescribed | | |  | | | Were the medicines purchased? | | Reason for non-purchase | Quantity prescribed (unit tablet) | Quantity served (unit tablet) | Cost (Ghc) |
| --- | --- | --- | --- | --- | --- | --- | --- | --- | --- | --- | --- | --- | --- | --- |
|  |  |  | Name/Strength/Dosage form | DP | MP | Pfizer | Sanofi | MSD | Yes | No |  |  |  |  |
| 18/9/14 | Patience Bonsu | PB1003KAB | Paracetamol-tab-500mg | √ |  | √ |  |  | √ |  |  |  | 40 | 2.40 |
|  |  |  |  |  |  |  |  |  |  |  |  |  |  |  |
|  |  |  |  |  |  |  |  |  |  |  |  |  |  |  |
|  |  |  |  |  |  |  |  |  |  |  |  |  |  |  |
|  |  |  |  |  |  |  |  |  |  |  |  |  |  |  |
|  |  |  |  |  |  |  |  |  |  |  |  |  |  |  |
