# Supplementary material for: A multi-center prospective cohort study to evaluate the effect of differential pricing and health systems strengthening on access to medicines and management of hypertension and diabetes in Ghana: A study protocol
Source: Gates Open Res. 2018 May 3;2:6. Originally published 2018 Feb 7. [Version 2] doi: 10.12688/gatesopenres.12797.2 (PMC5941285; doi:10.12688/gatesopenres.12797.2)
Supplement: Supplementary file 3 [file gatesopenres-2-13889-s0002.tgz › 4dd10987-e8ed-44b0-80bd-b229fe524c9f.zip!/Supplementary File 3 - Data collection tools/Participant booklet.pdf]

PARTICIPANT ID NO--------------------------------

GHANA ACCESS AND AFFORDABILITY PROGRAM (GAAP) PILOT STUDY

STUDY PARTICIPANT BOOKLET

GHANA ACCESS AND AFFORDABILITY PROGRAM (GAAP)

PILOT STUDY

Study Participant Booklet

Table of contents

Participant Information Sheet and Informed Consent Form	Pages 5-7
Eligibility Screener Form	Pages 8-9
Enrollment questionnaire	Pages 10-24
Hypertension KAP questionnaire (baseline)	Pages 25-26
Diabetes KAP questionnaire (baseline)	Pages 27-29
Clinical Data questionnaire	Pages 30-33
Clinical Data questionnaire for nonscheduled visits	Pages 34-45
GAAP Follow-up and KAP questionnaires (6months)	Pages 46-57
GAAP Follow-up and KAP questionnaires (12months)	Pages 58-69
GAAP Follow-up and KAP questionnaires (18months)	Pages 70-81
GAAP Follow-up and KAP questionnaires (24months)	Pages 82-93
Study Outcomes	Page 94

Participant Information Sheet

TITLE OF RESEARCH:

The Effects of Differential Pricing and Health Systems Strengthening activities on the management of Type II Diabetes Mellitus, Hypertension and Cancer in Ghana.

INTRODUCTION:

This is a research study being conducted at several hospitals across Ghana to improve patient access to medicines and healthcare delivery of hypertension, diabetes and cancer.

NATURE OF RESEARCH:

Our project seeks to improve patient access to medicines for efficient healthcare delivery to populations in Ghana, by improving the availability and affordability of quality, safe and highly effective medicines. For this purpose, we are collecting information from patients that will inform us about providing better treatment for these conditions.

PARTICIPANT INVOLVEMENT:

You have been selected to participate in this research activity because you suffer from

diabetes [ ] hypertension [ ] or cancer [ ]. [1]

The research will take place over a period of two years. Members of the research team will ask you questions about your condition and information about your household, if you agree to participate in the study. The research team members will also measure your height, weight and blood pressure. The clinic may do the following things depending on your condition: about a dessert-spoon of blood (10 mls) will be taken to test some of your bodily functions like kidney, liver, fat (cholesterol), general blood level and measure your blood sugar level (if you have diabetes). You will not pay for these tests. The questions, measurements and blood tests will be repeated when you attend the clinic for follow up.

[1] *Select appropriate condition*

POTENTIAL RISKS:

You may suffer slight pain when the blood sample is being with drawn. This, however, will be minimal as the lab technicians taking the blood are experienced.

BENEFITS:

The findings of the research will be important to the care of patients suffering from the health conditions we are looking into. These findings will help clinicians provide better care for you and your family members. You will receive additional education about your condition.

COMPENSATION/PAYMENT:

You will not be given compensation in the form of money or gift items for participating in the study. Being a participant in this study will be of no additional cost beyond the cost of your routine treatment. Your laboratory tests will be paid for by the research team.

CONFIDENTIALITY:

Your information will be kept in a secure place and only the researchers will have access to it. All information obtained from you will not appear anywhere with your name on it and cannot be linked to you in anyway. Your name will not be used in any publication or reports of this study.

VOLUNTARY PARTICIPATION/WITHDRAWAL:

Taking part in this study should be out of your own free will. You are not under any obligation to participate

in this research. Your contribution is entirely voluntary. You also have the right to withdraw from this study at any time should you choose to do so. You are not under any obligation to answer any question you find to be uncomfortable or private.

OUTCOME AND FEEDBACK:

This study will help us gain a better understanding of how to manage hypertension, diabetes and cancer in Ghana. We plan to share the results of this study with the head of this hospital and hope that any relevant consequence will be conveyed to you by the head of this hospital as appropriate. We hope that the outcomes of this study will inform doctors, nurses, pharmacists, policy-makers and the Ministry of Health about how to improve medical care for hypertension, diabetes and cancer.

APPROPRIATE ALTERNATIVE PROCEDURES AND TREATMENT:

If you choose not to participate, this will not affect the treatment of you, your family or your community members in this hospital in any way. You will still receive the same medical care if you refuse to participate in this study.

FUNDING INFORMATION:

The project is funded by the New Venture Fund, an organization in Washington DC in the United States of America.

CONTACTS:

If you have any questions concerning this study, please do not hesitate to contact the Principal Investigator, Dr. Stephen Fred Sarfo of the School of Medical Sciences, Kwame Nkrumah University of Science and Technology (KNUST) on 0243448464. You can also reach him at this

Email address: stephensarfo78@gmail.com.

You may also contact Mrs Hannah Frimpong of the Ghana Health Service Ethical Review Committee on 0507041223 or 0243235225 or by e-mail at: Hannah.frimpong@ghsmail.org

PID (Participant ID No.) [ ]-[ ]/[ ]-[ ]-[ ]-[ ]/[ ]-[ ]/[ ]

Consent Form for the Ghana Access and Affordability Program (GAAP) Pilot Study Participants

PARTICIPANT STATEMENT AND SIGNATURE:

I certify that I voluntarily agree to be a part of this study. The study requirements have been read to me/I have read the study requirements and all my questions have been addressed successfully. I know enough about the purpose, methods, risks and benefits of the research study to decide that I want to take part in it. I understand that I am free to discontinue participation at any time if I so choose without having to explain myself and I understand that this will not affect my future care or treatment. I understand that all information about me will be treated in strict confidence and that I will not be named in any written work arising from this study. I have received a copy of this information leaflet and consent form to keep for myself.

Name ______________________________

Signature or thumbprint of Participant ______________________________

Date ______________

IN CASES OF ILLITERATE PARTICIPANT

____

Witness Name

______________________________ ______________________________

Signature Date

INVESTIGATOR STATEMENT AND SIGNATURE

I have fully explained this research to ______________________________

and have given ample time to read about the study, as well as sufficient information on the study including procedures, risks and benefits, to enable the prospective participant make an informed decision to or not to participate in the study.

Name of person who sought consent ______________________________

Signature of person who sought consent ______________________________

Date ______________

Ghana Access and Affordability Program Pilot Study
Eligibility Screener Form

Screening Date: ☐ ☐-☐ ☐-☐ ☐ ☐ ☐

SECTION A: INCLUSION CRITERIA

Is the participant Age >=18 of years?

YES 1

NO........................2

1. Does the participant have a diagnosis of High Blood Pressure (either new or previously known diagnosis of hypertension, with SBP> 140 mm Hg and DBP > 90 mmHg . Tick as appropriate

YES....................1

NO......................2

If Yes, indicate whether patient is:

Newly diagnosed..1

Previously diagnosed................................2

Blood Pressure Reading number 1 (sitting for 5 minutes)	Systolic (mmHg)
	Diastolic (mmHg)
Blood Pressure Reading number 2 (sitting for 5 minutes)	Systolic (mmHg)
	Diastolic (mmHg)
Today, have you taken drugs (medication) that have been prescribed by a doctor or other health worker for raised blood pressure?	YES....................1
	NO......................2

2. Does the participant have a diagnosis of Diabetes (either new or previously diagnosed of type II diabetes-

- Random blood sugar of >11.1mmol/l with symptoms or
- Fasting blood glucose of >7 mmol/L on a single occasion with symptoms or
- Fasting blood sugar of > 7mmol/l on two occasions without symptoms or
- HbA1C >6.5% or already on treatment for Diabetes)

YES 1

NO.............................2

If Yes, indicate whether patient is:

Newly diagnosed.......................................1

Previously diagnosed...........................2

Question	Response
During the past 12 hours have you had anything to eat or drink, other than water? (IT IS ESSENTIAL THAT THE PARTICIPANT HAS FASTED.)	YES..1<br>NO..2
Fasting blood glucose	[__|__].[__|__] MMOL/L
Random blood glucose (checked at clinic for patients without laboratory measurement of FBS at enrolment)	[__|__].[__|__] MMOL/L
Hemoglobin A1C	[_____|_____]%
Today, have you taken insulin or other drugs (medication) that have been prescribed by a doctor or other health worker for raised blood glucose?	YES..1<br>NO..2

SECTION B: EXCLUSION CRITERIA

The following criteria MUST be answered NO for the participant to be included in the study:		Yes	No
1.	Is the Patient less than 18 years old?	☐	☐
2.	Is the Patient's FBS or RBS today more than 25 mmol/l (hyperglycaemia) or less than 3mmol/l (hypoglycaemia)	☐	☐
3.	Is patient's Blood Pressure today more than 180 / 110mmHg with any of the following complaints: chest pain, shortness of breath, back pain, numbness/ weakness, change in vision, difficulty speaking?	☐	☐
4.	Is the patient not willing to give informed pre-consent, and consent or withdrawal or consent?	☐	☐
If any of the above criteria is answered YES, the participant is NOT eligible for the study and must not be included in the study. Please list reason(s) for ineligibility for screen failure on Participant Eligibility Review page.			

SECTION C: ELIGIBILITY TO PARTICIPATE IN THE STUDY

Does patient qualify to participate in this study?

YES................1

NO..................2

Name of study nurse ______________________________

Signature of study nurse ______________________________

Ghana Access and Affordability Program Pilot Study Enrollment Questionnaire

The Ghana Access and Affordability Program is a research study that is conducting this survey to assess the disease profile of persons living with hypertension, diabetes and cancer. This will help inform authorities on how your ability to pay for certain medications for your disease influences treatment. All information gathered for this questionnaire will be kept CONFIDENTIAL.

Thank you for your participation.

**LOCATION, DATE AND TIME**	**(PLEASE CIRCLE THE CORRECT RESPONSE)**
Interviewer Name	Interviewer ID
Institution/ Facility ID (ENTER INSTITUTION ID FROM LIST PROVIDED)	TAMALE TEACHING HOSPITAL..........1
	KOMFO ANOKYE TEACHING HOSPITAL..........2
	KING'S MEDICAL CENTRE..........3
	AGOGO PRESBYTARIAN HOSPITAL..........4
	ATUA GOVERNMENT HOSPITAL..........5
	KORLE-BU TEACHING..........6
Date of interview (dd-mm-year)	[___] [___]-[___] [___]-[___] [___] [___] [___]
Interview start time: [___] [___] : [___] [___]<br>Interview End time: : [___] [___] : [___] [___]<br>(USE 24 HOUR CLOCK)	Duration of interview [_____] minutes

**CONSENT AND INTERVIEW LANGUAGE**	**(PLEASE CIRCLE THE CORRECT RESPONSE)**
Consent has been read and obtained	YES..........1
	NO..........2 (IF NO,END)
Interview Language (INSERT LANGUAGE)	English..........1
	Other Specify..........2

**SECTION 1: DEMOGRAPHIC INFORMATION (PLEASE CIRCLE THE CORRECT RESPONSE)**		
Question		Response
A1	Gender (RECORD MALE / FEMALE AS OBSERVED)	MALE..........1
		FEMALE..........2

A2	Please provide your date of birth. (dd-mm-year eg:01/ JAN/2014) (if participant cannot provide date of birth, ask for age. If date of birth is unknown, ask for National Health Insurance Card and check date of birth)	[___|___]/[___/___]/[___|___|___|___]
A3	How old are you? (If the age is unknown, help participant estimate their age by interviewing them about their recollection of widely known major events)	[__|__]
A4	Do you have a valid National Health Insurance Scheme card? (Ask to see National Health Insurance Card)	YES (CARD SHOWN)........................1
		YES (CARD NOT SHOWN) 2
		NO........................3
A5	Is the card expired? (PLEASE EXAMINE NATIONAL HEALTH INSURANCE CARD)	YES........................1
		NO........................2
A6	Please indicate the location of your residence (PERSON COMPLETING SURVEY WILL INDICATE THE AREA OF RESIDENCE)	URBAN........................1
		RURAL........................2
		PERIURBAN........................3
A7	Can you please provide your cell phone number; this will be kept Confidential	

**EXPANDED: DEMOGRAPHIC INFORMATION (PLEASE CIRCLE THE CORRECT RESPONSE)**		
A8	What is the highest level of education you have completed? (CHOOSE ONE)	No formal schooling........................0
		Less than primary school........................1
		Non-formal education........................2
		Primary school completed........................3
		Secondary school completed........................4
		High school completed........................5
		College/University completed........................6
		Post graduate degree........................7
		REFUSED........................88
		DON'T KNOW........................99

A9	What is your marital status? CHOOSE ONE)	Never married............1	
		Currently married............2	
		Separated............3	
		Divorced............4	
		Widowed............5	
		Cohabitating............6	
		REFUSED............88	
		DON'T KNOW............99	
A10	Which of the following best describes your work status over the past 12 months? (CHOOSE ONE)	Government employee............1	
		Private Company Employee............2	
		Self-employed............3	
		Student............4	
		Homemaker............5	
		Retired............6	
		Unemployed (able to work) 7	
		Unemployed (unable to work) 8	
		REFUSED............88	
		DON'T KNOW............99	
A11	What is the total number of people living in your household? (Please explain that a household is defined as "as the group that eats from the same pot").	Number of People	[______]
A12	How many people that are 17 or less are in your household (Please Explain That A Household Is Defined As "As The Group That Eats From The Same Pot").	Number of Children	[______]
A13	How many females are in your household? (Please explain that a household is defined as "as the group that eats from the same pot").	Number of females	[______]

SECTION 2: SMOKING		
Question	(PLEASE CIRCLE THE CORRECT RESPONSE)	
B1	Do you currently smoke any tobacco products, such as cigarettes, cigars or pipes? (CHOOSE ONE)	YES..........1 (B2)<br>NO..........2 (B3)<br>REFUSED..........88<br>DON'T KNOW..........99
B2	On average, how many tobacco products (such as cigarettes, cigars or pipes) do you smoke each day?	[_______]
B3	Have you smoked in the past? (CHOOSE ONE)	YES..........1 (B4)<br>NO..........2 (C1)<br>REFUSED..........88<br>DON'T KNOW..........99
B4	How many years have you smoked in total before quitting? (YEARS)	[____]-[____]

SECTION 3: ALCOHOL CONSUMPTION		
Question	(PLEASE CIRCLE THE CORRECT RESPONSE)	
C1	Do you currently use alcoholic beverage such as beer, wine, spirits, palm wine, or akpeteshie? (CHOOSE ONE)	YES..........1 (C2)<br>NO..........2 (D1)<br>REFUSED..........88<br>DON'T KNOW..........99
C2	In the past month, how frequently have you had at least one alcoholic drink? (CHOOSE ONE)	DAILY..........1<br>5-6 days per week..........2<br>3-4 days per week..........3<br>1-2 days per week..........4<br>1-3 days per month..........5<br>NONE..........6<br>NOT APPLICABLE..........7

**SECTION 4: DIET**<br>The next questions ask about the fruits and vegetables that you usually eat. I have a nutrition card here that shows you some examples of local fruits and vegetables. Each picture represents the size of a serving. As you answer these questions please think of a typical week in the last year.		
Question	Response	
D1	In a typical week, on how many days in the past week have you had fruits?	Number of days [_________]<br>(IF ZERO, GO TO QUESTION D3)
D2	How many servings of fruit do you eat on one of those days?	Number of servings [_________]
D3	In a typical week, on how many days in the past week have you had vegetables?	Number of days [_________]<br>(IF ZERO, GO TO QUESTION D5)
D4	How many servings of vegetables do you eat on one of those days?	Number of servings [_________]
D5	In a typical week, how often is salt added to food that has already been prepared? (PLEASE CIRCLE THE CORRECT RESPONSE)	Never.......0
		Rarely.......1
		Occasionally.......,,.......2
		Very often.......3
		REFUSED.......88
		DON'T KNOW.......99

**SECTION 5: PHYSICAL ACTIVITY**		
Question	Response	
E1	Do you frequently perform physical activities that cause a small increase in breathing or make your heart rate go up, such as (fast/brisk) walking, jogging, bicycling or other? (PLEASE CIRCLE THE CORRECT RESPONSE)	YES.......1
		NO.......2 (F1)
E2	How much time (in minutes) do you spend walking (fast/briskly), jogging, bicycling or any other form of exercise on a typical day?	[______] MINUTES

SECTION 6: HISTORY OF RAISED BLOOD PRESSURE		
Question		Response
F1	Have you ever been told by a doctor or other health worker that you have raised blood pressure or hypertension? (PLEASE CIRCLE THE CORRECT RESPONSE)	YES.......1 (F2)
		NO.......2 (G1)
		REFUSED.......88
		DON'T KNOW.......99
F2	How many months/years ago were you first told you had high blood pressure?	[______] [______]<br>YEARS MONTHS

SECTION 7: HYPERTENSION TREATMENT ADHERENCE		
Question		(PLEASE CIRCLE THE CORRECT RESPONSE)
F4	How often did you forget to take your High Blood Pressure medicine in the past two weeks? (CHOOSE ONE)	ALL THE TIME (14/14 DAYS).......1
		MOST OF THE TIME (10/14 DAYS).......2
		SOME OF THE TIME (7/14 DAYS).......3
		NEVER (0/14 DAYS).......4
F5	How often did you decide not to take your High Blood Pressure medicine in the past two weeks? (CHOOSE ONE)	ALL THE TIME (14/14 DAYS).......1
		MOST OF THE TIME (10/14 DAYS).......2
		SOME OF THE TIME (7/14 DAYS).......3
		NEVER (0/14 DAYS).......4
F6	How often do you eat salty food? (CHOOSE ONE)	ALL THE TIME (30/30 DAYS).......1
		MOST OF THE TIME (24/30 DAYS).......2
		SOME OF THE TIME (15/30 DAYS).......3
		NEVER (0/30 DAYS).......4
F7	How often do you add salt to your food before you eat it? (CHOOSE ONE)	ALL THE TIME (30/30 DAYS).......1
		MOST OF THE TIME (24/30 DAYS).......2
		SOME OF THE TIME (15/30 DAYS).......3
		NEVER (0/30 DAYS).......4

F8	How often do you eat fast food (food purchased for take-out)? (CHOOSE ONE)	ALL THE TIME (30/30 DAYS)........1<br>MOST OF THE TIME (24/30 DAYS)........2<br>SOME OF THE TIME (15/30 DAYS)........3<br>NEVER (0/30 DAYS)........4
F9	How often does the clinic make scheduled return appointments for you before you leave? Over the last 3 visits (CHOOSE ONE)	ALL THE TIME (3/3) 100% OF THE TIME)......1<br>MOST OF THE TIME (2/3) (66% OF THE TIME) ....2<br>SOME OF THE TIME (1/3) (33% OF THE TIME) ....3<br>NEVER 4
F10	How often do you miss scheduled clinic appointments? Over the last 3 visits (CHOOSE ONE)	ALL THE TIME (3/3) 100% OF THE TIME)......1<br>MOST OF THE TIME (2/3) (66% OF THE TIME) ....2<br>SOME OF THE TIME (1/3) (33% OF THE TIME) ....3<br>NEVER 4
F11	How often do you forget to get prescriptions refilled? Over the last 3 visits (CHOOSE ONE)	ALL THE TIME (3/3) 100% OF THE TIME)......1<br>MOST OF THE TIME (2/3) (66% OF THE TIME) ....2<br>SOME OF THE TIME (1/3) (33% OF THE TIME) ....3<br>NEVER 4
F12	How often do you run out of High Blood Pressure pills? Over the last 3 visits (CHOOSE ONE)	ALL THE TIME (3/3) 100% OF THE TIME)......1<br>MOST OF THE TIME (2/3) (66% OF THE TIME) ....2<br>SOME OF THE TIME (1/3) (33% OF THE TIME) ....3<br>NEVER 4
F13	How often to you skip your High Blood Pressure medicine before you go to the doctor? Over the last 3 visits. (CHOOSE ONE)	ALL THE TIME (3/3) 100% OF THE TIME)......1<br>MOST OF THE TIME (2/3) (66% OF THE TIME) ....2<br>SOME OF THE TIME (1/3) (33% OF THE TIME) ....3<br>NEVER 4

F14	How often do you miss taking your High Blood Pressure pills when you feel sick? (CHOOSE ONE)	ALL THE TIME (30/30 DAYS)........1
		MOST OF THE TIME (24/30 DAYS)........2
		SOME OF THE TIME (15/30 DAYS)........3
		NEVER (0/30 DAYS)........4
F16	How often do you take someone else's High Blood Pressure pills? (CHOOSE ONE)	ALL THE TIME (30/30 DAYS)........1
		MOST OF THE TIME (24/30 DAYS)........2
		SOME OF THE TIME (15/30 DAYS)........3
		NEVER (0/30 DAYS)........4
F17	How often do you miss taking your high Blood Pressure pills when you are careless? (CHOOSE ONE)	ALL THE TIME (30/30 DAYS)........1
		MOST OF THE TIME (24/30 DAYS)........2
		SOME OF THE TIME (15/30 DAYS)........3
		NEVER (0/30 DAYS)........4

**SECTION 8: HISTORY OF DIABETES**		
Question		Response
G1	Have you ever been told by a doctor or other health worker that you have raised blood sugar or diabetes? (CHOOSE ONE)	YES........1 (G2)
		NO........2 (H1)
		REFUSED........88
		DON'T KNOW........99
G2	How many years/months ago were you first told you had diabetes?	[______] YEARS [______] MONTHS
G3	Are you currently taking medication for diabetes prescribed by a doctor or other health worker? (CHOOSE ONE)	YES........1
		NO........2
		REFUSED........88
		DON'T KNOW........99
G4	Do you take insulin for diabetes?	YES........1
		NO........2
		REFUSED........88
		DON'T KNOW........99

**SECTION 9: DIABETES TREATMENT ADHERENCE**		
Question		(PLEASE CIRCLE THE CORRECT RESPONSE)
G5	Do you sometimes forget to take your diabetes pills? (CHOOSE ONE)	YES........1
		NO........2

G6	People sometimes miss taking their medications for reasons other than forgetting. Thinking over the past two weeks, were there any days when you did not take your diabetes medicine? (CHOOSE ONE)	YES.....1<br>NO.....2
G7	Have you ever cut back or stopped taking your medication without telling your doctor, because you felt worse when you took it? (CHOOSE ONE)	YES.....1<br>NO.....2
G8	When you travel or leave home, do you sometimes forget to bring your diabetes medication? (CHOOSE ONE)	YES.....1<br>NO.....2
G9	Did you take your diabetes medicine yesterday?	YES.....1<br>NO.....2
G10	When you feel like your diabetes is under control, do you sometimes stop taking your medicine? (CHOOSE ONE)	YES.....1<br>NO.....2
G11	Taking medication everyday is a real inconvenience for some people. Do you ever feel hassled about sticking to your diabetes treatment plan? (CHOOSE ONE)	YES.....1<br>NO.....2

SECTION 9: HISTORY OF CANCER		
Question		(PLEASE CIRCLE THE CORRECT RESPONSE)
H1	Have you ever been diagnosed with cancer? (CHOOSE ONE)	YES.....1 (H2)<br>NO.....2<br>REFUSED.....88<br>DON'T KNOW.....99
H2	How many months or years ago were you told you had a cancer or tumor	[______] YEARS [______] MONTHS
H3	If you have cancer, what type of cancer? ((CHOOSE ONE))	PROSTATE.....1<br>LUNG.....2<br>BREAST.....3<br>CERVICAL.....4<br>COLON.....5<br>LIVER.....6<br>OTHER SPECIFY.....7<br>______________________<br>REFUSED.....88<br>DON'T KNOW.....99
H4	Are you currently taking medication for cancer prescribed by a doctor or other health worker? (CHOOSE ONE)	YES.....1<br>NO.....2<br>REFUSED.....88<br>DON'T KNOW.....99

SECTION 10: MEDICATION ADHERENCE		
Question		(PLEASE CIRCLE THE CORRECT RESPONSE)
I1	In the past two weeks, did you forget or miss your medicines for one or more days? (CHOOSE ONE)	YES........1
		NO........2 (J1)
I2	What are the reasons that you did not take your medicine? (RECORD ALL THAT APPLY)	TOO EXPENSIVE........1
		I FORGET........2
		SIDE EFFECTS........3
		RAN OUT OF MEDICINES........4
		OTHER SPECIFY........5 ____________
		NOT APPLICABLE........6

SECTION 11: COMORBIDITIES			
Question			(PLEASE CIRCLE THE CORRECT RESPONSE)
J1	Have you ever been diagnosed with any of the following conditions?	High cholesterol	YES........1
			NO........2
		1a. Heart attack (CHEST PAIN FOR MORE THAN 30 MINUTES WHILE RESTING) or angina (CHEST PAIN WITH ACTIVITY)?	YES........1
			NO........2
		2b. Heart failure (SWELLING IN YOUR BOTH LEGS, SHORTNESS OF BREATH AND SHORTNESS OF BREATH WHEN LYING DOWN)	YES........1
			NO........2
		3=Stroke (WEAKNESS ON ONE SIDE OF THE BODY)	YES........1
			NO........2
		4= HIV	YES........1
			NO........2
		5= Tuberculosis	YES........1
			NO........2

SECTION 12: HOSPITALIZATIONS	
Question	(PLEASE CIRCLE THE CORRECT RESPONSE)

K1	Have you been admitted to the hospital for High Blood pressure in the past year? (CHOOSE ONE)	YES......1
		NO......2
		NOT APPLICABLE......3
K2	Have you been admitted to the hospital for Diabetes in the past year? (CHOOSE ONE)	YES......1
		NO......2
		NOT APPLICABLE......3

SECTION 13: SOURCE OF MEDICATION		
Question		(PLEASE CIRCLE THE CORRECT RESPONSE)
L1	Please indicate where you get your medicine for your diabetes or high blood pressure (RECORD ALL THAT APPLY)	HOSPITAL PHARMACY......1
		COMMUNITY PHARMACY......2
		LICENSED CHEMICAL SELLER......3
		OTHER SPECIFY......4
		REFUSED......88
		DON'T KNOW......99
L2	Have you had problems getting medicines for your high blood pressure or diabetes in the past 6 months? (RECORD ALL THAT APPLY)	YES (HIGH BLOOD PRESSURE)......1
		YES (DIABETES)......2
		YES (BOTH HIGH BLOOD PRESSURE AND DIABETES)......3
		NO TO ALL THE ABOVE......4 (L5)
		REFUSED......88
		DON'T KNOW......99
L3	If you had difficulty obtaining your medicine in the past 6 months, please indicate where. (RECORD ALL THAT APPLY)	HOSPITAL PHARMACY......1
		COMMUNITY PHARMACY......2
		LICENSED CHEMICAL SELLER......3
		OTHER SPECIFY......4
		REFUSED......88
		DON'T KNOW......99

L4	Please specify why it was difficult to obtain your medicine? (RECORD ALL THAT APPLY)	NOT AVAILABLE AT POINT OF CARE.........1
		TOO EXPENSIVE.........2
		NHIS NOT ACCEPTED AT PHARMACY.........3
		OTHER SPECIFY.........4 ________
		NOT APPLICABLE.........5
		REFUSED.........88
		DON'T KNOW.........99
L5	In the past 6 months, have you ever tried to treat your high blood pressure or diabetes with an alternative method? If yes, choose all that apply. If No, (L6) (RECORD ALL THAT APPLY)	HERBAL MEDICINE.........1
		HOMEOPATHY.........2
		ACUPUNCTURE.........3
		TRADITIONAL HEALER.........4
		PRAYER CAMP.........5
		OTHER SPECIFY.........6
		NONE OF THE ABOVE.........7
L6	In the past 6 months, have you been referred to a Licensed Chemical Seller or Community Pharmacy because your medicine was not available at the Hospital Pharmacy?	YES.........1
		NO.........2 (M1)
L7	If yes, please indicate place of referral (where you obtained your medicine)	COMMUNITY PHARMACY.........1
		LICENSED CHEMICAL SELLER.........2
		OTHER SPECIFY.........3 ________

**SECTION 14: HEALTH EXPENDITURE**		
Question		Please circle the correct response
M1	Are all of the medicines prescribed by your physician paid for by NHIS? (	YES.........1 (M3)
		NO.........2 (M2)
M2	How much do you spend on purchasing medicines for high blood pressure, diabetes (or both) each month?	GH¢|__|__|__| . |__|__|
M3	On average, how much does it cost you to travel to this clinic per day (both ways)? (Please include yourself and caregiver if this applies)	GH¢|__|__|__| . |__|__|
M4	How much money can you contribute towards your health care expenses each month?	GH¢|__|__|__| . |__|__|
M5	If you needed to buy additional medicines for your condition, would you?	YES.........1
		NO.........2

SECTION: 15. HOUSEHOLD INCOME		
Question		Response
N1	How many people are earning an income in your household?	
N2	On average, what is the monthly income for the entire household? (CHOOSE ONE)	>1000 GHc.......1
		501-1000 GHc.......2
		101-500GHc.......3
		90-100 GHc.......4
		<90 GHc.......5
		REFUSED.......88
		DON'T KNOW.......99
N3	How many people in the household depend on this monthly income?	
N4	Which of these options add to the net monthly income of your household? (RECORD ALL THAT APPLY)	INCOME FROM COMPANY OR ACTIVITIES.......1
		REMITTANCES from family living in or outside of Ghana.......2
		PENSION OR SSNIT BENEFITS.......3
		DISABILITY BENEFITS (SICKNESS)....4
		PETTY TRADE.......5
		OTHER SPECIFY.......6 ______________

SECTION16: HOUSEHOLD INDICATORS		
Question. Please answer these questions regarding your household. Household is defined as a group of people who eat from the same pot.		(PLEASE CIRCLE THE CORRECT RESPONSE)
O1	Are there any members in your household who have completed 5 years of education?	YES.......1
		NO.......2
O2	Are there any children in your household attending school (from kindergarten up to Primary 6)?	YES.......1
		NO.......2
O3	In the past 12 months, are there any children under the age of 5 who died in your household?	YES.......1
		NO.......2
O4	In the past 12 months, was there a female household member who died while pregnant, during delivery, or within 6 weeks after the end of a pregnancy or childbirth in past 12 months?	YES.......1
		NO.......2
O5	Does your household have electricity or is it connected to the national grid?	YES.......1
		NO.......2
O6	Does your household have access to safe drinking water?	YES.......1
		NO.......2

O7	Does your household have a floor composed of any of the following? (RECORD ALL THAT APPLY)	EARTH......1<br>MUD......2<br>DUNG......3<br>NONE OF THE ABOVE......4
O8	Does your household use any of the following to cook? (RECORD ALL THAT APPLY)	WOOD......1<br>CHARCOAL......2<br>CROP RESIDUE......3<br>SAW DUST......4<br>ANIMAL WASTE......5<br>NONE OF THE ABOVE......6
O9	Does your household have access to a flush toilet, pit latrine, ventilated improved pit?	YES......1<br>NO......2
O10	Do you own the following assets? (RECORD ALL THAT APPLY)	RADIO......1<br>TELEVISION......2<br>REFRIGERATOR......3<br>BICYCLE......5<br>MOTORBIKE......6<br>CAR ......7<br>NONE OF THE ABOVE......8

**Question**	**Explanation of score (please assign a point based on how the patient answers the question)**	**Score (0 or 1)**	**Weight**	**Total = Indicator Score x weight**
O1	Yes=0 pt<br>No=1 pt		3	
O2	Yes=0 pt<br>No=1 pt		3	
O3	Yes=1pt<br>No=0pt		3	
O4	Yes=1 pt<br>No=0 pt		3	
O5	Yes=0 pt<br>No=1 pt		1	
O6	Yes=0 pt<br>No=1 pt		1	
O7	Yes=1 pt<br>No=0 pt		1	
O8	Yes=1 pt<br>No=0 pt		1	
O9	Yes=1 pt<br>No= 0 pt		1	
O10	Yes=0 pt<br>No=1 pt		1	
Total Score		Add total score =		

SECTION 15B: ELIGIBILITY FOR DIFFERENTIAL PRICING (REFER TO MDI)

*1. Household Monthly Income < 90 GHc per month YES..............1

NO...............2

*2. Multidimensional Poverty Index (>=6/18 on Multidimensional Poverty Index) YES..............1

NO...............2

*3. Is the patient eligible for Differential Pricing (if score >=6/18 AND monthly income <90 GH cedis)?

YES..............1

NO...............2

**SECTION 17:LIFESTYLE ADVICE**<br>During the past six months, has a doctor or other health worker advised you to do any of the following:		
P1	Quit using tobacco or don't start	YES..........1
		NO..........2
P2	Reduce salt in your diet	YES..........1
		NO..........2
P3	Eat at least five servings of fruit and/or vegetables each day	YES..........1
		NO..........2
P4	Reduce fat in your diet	YES..........1
		NO..........2
P5	Start or do more physical activity	YES..........1
		NO..........2
P6	Maintain a healthy body weight or lose weight	YES..........1
		NO..........2

**SECTION 18: CANCER SCREENING**<br>During the past six months, has a doctor or other health worker advised you to do any of the following?		
Q1	Breast Cancer screening	YES..........1
		NO..........2
Q2	Colon Cancer screening (fecal blood test)	YES..........1
		NO..........2

Ghana AccessAnd Affordability Program Pilot Study
Hypertension Knowledge Attitude and Practices (KAP) Questionnaire

**LOCATION AND DATE** (PLEASE CIRCLE THE CORRECT RESPONSE)	
Name of person completing questionnaire	
Institution/ Facility ID	TAMALE TEACHING HOSPITAL.........1
	KOMFO-ANOKYE TEACHING HOSPITAL.........2
	KORLE-BU TEACHING HOSPITAL.........3
	KING'S MEDICAL CENTRE.........4
	AGOGO PRESBYTERIAN HOSPITAL.........5
	ATUA GOVERNMENT HOSPITAL.........6
Date of completion of the instrument	[___]/[___]/[___]<br>dd mm year

**WE ARE INTERESTED IN SOME THINGS ABOUT YOU AND YOUR HEALTH. PLEASE RESPOND TO ALL THE QUESTIONS AS BEST AS YOU CAN. THERE ARE NO RIGHT OR WRONG ANSWERS. THE INFORMATION YOU PROVIDE WILL REMAIN CONFIDENTIAL.**		
Question		Please circle the correct response
A1	What does the term hypertension mean? :(SELECT ALL THAT APPLY)	HIGH BLOOD PRESSURE.........1
		HIGH BLOOD SUGAR.........2
		I DON'T KNOW.........3
		OTHER (SPECIFY)
A2	There are two numbers used in measuring blood pressure levels. What should the upper number be?(CHOOSE ONE)	LESS THAN 140.........1
		GREATER THAN 140.........2
		EQUAL TO 140.........3
		I DON'T KNOW.........99
		OTHER (SPECIFY)

A3	What should the lower number be? (CHOOSE ONE)	LESS THAN 90.....................................1
		GREATER THAN 90.......................2
		EQUAL TO 90.......................................3
		I DON'T KNOW....................................99
		OTHER (SPECIFY)
A4	How serious of a health concern has high blood pressure been to you?(CHOOSE ONE)	VERY SERIOUS CONCERN...1
		SOMEWHAT SERIOUS CONCERN...2
		NOT AT ALL SERIOUS CONCERN...3
A5	How important do you think taking medicine is to keep blood pressure under control? (CHOOSE ONE)	VERY IMPORTANT.........................1
		SOMEWHAT IMPORTANT...2
		NOT AT ALL IMPORTANT...3
A6	What is the most important factor(s) in controlling your high blood pressure? (SELECT ALL THAT APPLY)	TAKING MEDICATIONS...1
		EXERCISING...2
		LESS STRESS...3
		QUITTING SMOKING IF YOU ARE SMOKING.............4
		REDUCING SALT INTAKE IN YOUR DIET.........................5
		REDUCING ALCOHOL INTAKE......................6
		LOSING WEIGHT..7
		ALL OF THE ABOVE...8
		I DON'T KNOW...99
		OTHER (SPECIFY)

Ghana Access And Affordability Program Pilot Study
Diabetes Knowledge Attitude and Practices (KAP) Questionnaire

**LOCATION AND DATE** (PLEASE CIRCLE THE CORRECT RESPONSE)	
Name of person completing questionnaire	
Institution/ Facility ID	TAMALE TEACHING HOSPITAL.....1
	KOMFO-ANOKYE TEACHING HOSPITAL.....2
	KORLE-BU TEACHING HOSPITAL.....3
	KING'S MEDICAL CENTRE.....4
	AGOGO PRESBYTERIAN HOSPITAL.....5
	ATUA GOVERNMENT HOSPITAL.....6
Date of completion of the instrument	[___]/[___]/[___]<br>dd mm year

**WE ARE INTERESTED IN SOME THINGS ABOUT YOU AND YOUR HEALTH. PLEASE RESPOND TO ALL THE QUESTIONS AS BEST AS YOU CAN. THERE ARE NO RIGHT OR WRONG ANSWERS. THE INFORMATION YOU PROVIDE WILL REMAIN CONFIDENTIAL.**		
Question		(PLEASE CIRCLE THE CORRECT RESPONSE)
A1	Diabetes is a condition in which the body contains:(SELECT ALL THAT APPLY)	A HIGHER LEVEL OF SUGAR IN THE BODY THAN NORMAL.....1
		A LOWER LEVEL OF SUGAR IN THE
		BODY THAN NORMAL.....2
		EITHER A HIGHER OR A LOWER LEVEL OF SUGAR IN THE BODY THAN NORMAL.....3
		I DON'T KNOW.....4
		OTHER (SPECIFY)
A2	The symptom (s) of diabetes is/are: (SELECT ALL THAT APPLY)	INCREASED FREQUENCY OF URINATION.....1
		INCREASED THIRST AND HUNGER.....2
		INCREASED TIREDNESS.....3
		SLOW HEALING OF WOUNDS.....4
		ALL THE ABOVE.....5
		OTHER (SPECIFY)

A3	Diabetes, if not treated can lead to: (SELECT ALL THAT APPLY)	EYE PROBLEMS...1
		KIDNEY PROBLEMS...2
		FOOT ULCERS...3
		HEART PROBLEMS...4
		ALL THE ABOVE...5
		I DON'T KNOW...6
		OTHER (SPECIFY)
A4	In a diabetic patient, high blood pressure can increase or worsen (SELECT ALL THAT APPLY)	THE RISK OF A HEART ATTACK...1
		THE RISK OF STROKE...2
		THE RISK OF EYE PROBLEMS...3
		THE RISK OF KIDNEY PROBLEMS...4
		ALL THE ABOVE...5
		I DON'T KNOW...6
		OTHER (SPECIFY)
A5	The lifestyle modification (s) required for diabetic patients are/is? (SELECT ALL THAT APPLY)	WEIGHT REDUCTION...1
		STOPPING SMOKING...2
		STOPPING ALCOHOL INTAKE...3
		ALL THE ABOVE...4
		I DON'T KNOW...5
		OTHER (SPECIFY)
A6	The important factors that help in controlling blood sugar are (SELECT ALL THAT APPLY)	A CONTROLLED AND PLANNED DIET...1
		REGULAR EXERCISE...2
		MEDICATION...3
		ALL THE ABOVE...4
		NONE...5
		OTHER (SPECIFY)

A7	A well-balanced diet includes: (SELECT ALL THAT APPLY)	VEGETABLES........1
		FRUITS........2
		CARBOHYDRATES........3
		ALL THE ABOVE........4
		NONE........5
		OTHER (SPECIFY)
A8	For proper foot care, a diabetic patient (SELECT ALL THAT APPLY)	SHOULD INSPECT AND WASH THE FEET DAILY........1
		SHOULD SELECT THE BEST POSSIBLE FOOTWEAR........2
		SHOULD WALK BAREFOOT INSIDE AND OUTSIDE THE HOUSE........3
		SHOULD NOT WALK BAREFOOT INSIDE AND OUTSIDE THE HOUSE........4
		OTHER (SPECIFY)
A9	Upon control of diabetes, the medicines: (SELECT ALL THAT APPLY)	CAN BE STOPPED IMMEDIATELY........1
		CAN BE STOPPED AFTER ONE MONTH........2
		SHOULD BE CONTINUED FOR LIFE........3
		I DON'T KNOW........99
		OTHER (SPECIFY)
A10	How do you manage low blood sugar symptoms? (SELECT ALL THAT APPLY)	BY TAKING SUGAR........1
		BY TAKING MEDICINES........2
		BY TAKING INSULIN........3
		I DON'T KNOW........99
		OTHER (SPECIFY)

GAAP Clinical Data

Location and Date	Response	Code
Institution/ Facility ID		
Patient Name		
Date of visit (eg 01/JAN/2014)	[___]/[___]/[___]<br>dd mm yyyy	

To extract from chart at time of enrollment and during each follow-up visit

A. VITAL SIGNS

	Baseline	2 months	4 months	6 months	8 months	10 months	12 months	14 months	16 months	18 months	20 months	22 months	24 months
Height (cm)		XXX	XXX	XXX	XXX	XXX	XXX	XXX	XXX	XXX	XXX	XXX	XXX
Weight (kg)													
SBP (mm Hg)													
DBP (mm Hg)													
Pulse (beats/ min)													
Waist circum-ference													

B. MEDICATIONS AT (FOR DIABETES OR HYPERTENSION)

Medications at time of enrollment

Medication	Dose, frequency	Date started	Date discontinued	Please check (x) here if medication is on list of Innovator drugs

C. COMPLICATIONS (PRESCRIBER TO INDICATE YES, NO OR NA (FOR NOT ASSESSED) AT EACH VISIT

	Baseline	2 months	4 months	6 months	8 months	10 months	12 months	14 months	16 months	18 months	20 months	22 months	24 month
Peripheral Neuropathy													
CVD events*													
CVA													
Ulcers													
Renal failure													
Diabetic foot infection													
Amputations													
Other medical diagnoses made today?													
Does patient need to be hospitalized today?													
Any hospitalization since last visit?													
If hospitalized, state reason for hospitalization													
Any Side Effects to report#?													
Initials of prescriber													
Date of visit (dd/ mm/yy)													

- **CVD events: acute MI, unstable angina, cardiac mortality*
- *# Please fill in details of side effects under section G.*

D. LABORATORY DATA (PRESCRIBER TO RECORD VALUES)

	Baseline	2 months	4 months	6 months	8 months	10 months	12 months	14 months	16 months	18 months	20 months	22 months	24 months
FBS (mmol/l)													
HbA1C		XXX	XXX		XXX	XXX		XXX	XXX		XXX	XXX	
Total Cholesterol (mmol/l)		XXX	XXX	XXX	XXX	XXX	XXX	XXX	XXX	XXX	XXX	XXX	XXX
HDL (mmol/l)		XXX	XXX	XXX	XXX	XXX	XXX	XXX	XXX	XXX	XXX	XXX	XXX
LDL (mmol/l)		XXX	XXX	XXX	XXX	XXX	XXX	XXX	XXX	XXX	XXX	XXX	XXX
Triglycerides (mmol/l)		XXX	XXX	XXX	XXX	XXX	XXX	XXX	XXX	XXX	XXX	XXX	XXX
Creatinine (umol/l)		XXX	XXX	XXX	XXX	XXX		XXX	XXX		XXX	XXX	XXX
Urine protein		XXX	XXX		XXX	XXX		XXX	XXX		XXX	XXX	

E. PHYSICIAN'S DECISION MAKING ON PATIENT'S TREATMENT REGIMEN

Medication	Dose, frequency	Date started	Date discontinued	State month of follow-up when medication change was made	Please place a "X" if this medicine is on the DP list

F. MEDICATION CHANGES MADE DURING FOLLOW-UP VISITS

Medication	Type of Change*	Dose, frequency	Date started	Date discontinued	State month of follow-up when medication change was made	Please che (x) here i medication i the DP Lis

**1=Substitution of Medication   2=Increase in dose of medication   3=Decrease in dose of medication   4=Withdrawal of medication*

G. SIDE EFFECTS

Please fill in details of side effects below:

Medication	Side effect	Date started	Grade (NIH scale)*

** Indicate if side effects are mild (grade 1), moderate (grade 2), severe (grade 3), life threatening (grade 4) or fatal (grade 5)*

GAAP Clinical Data for non-scheduled visits

Location and Date	Response	Code
Name of physician/prescriber		
Institution/ Facility ID		
Patient Name		
Date of visit (eg 01/JAN/2014)	[___]/[___]/[___]<br>dd mm yyyy	

VITAL SIGNS

Weight (in kg)	
Pulse	
Systolic Blood Pressure (mm Hg)	
Diastolic Blood Pressure (mm Hg)	
Fasting Blood Sugar (only for diabetics)	

1. Please state the reason for the unscheduled visit to the prescriber?

2. Has the patient developed complications related to Diabetes and or hypertension since their last visit?

   Yes ☐ No ☐

3. Use checklist below

	Tick (X) for yes or (O) for no
Peripheral Neuropathy	
CVD events*	
CVA	
Ulcers	
Renal failure	
Diabetic foot infection	
Amputations	
Other	

• *CVD events: acute MI, unstable angina, cardiac mortality.*

PID (Participant ID No.) ☐-☐/☐-☐-☐-☐/☐-☐/☐

4. Was patient hospitalized since last visit? Yes ☐ No ☐

5. If Yes to question 4, please state the reason for hospitalization?

6. Are there any adverse events due to medications patient is taking? Yes ☐ No ☐

7. If yes, to question 6 , please state the side effect(s) below?

Medication	Side effect	Date started	Grade (NIH scale)*

** Indicate if side effects are mild (grade 1), moderate (grade 2), severe (grade 3), life threatening (grade 4) or fatal (grade 5)*

8. Medication changes during this visit? Yes ☐ No ☐

9. If yes to question 8, list new medications prescribed.

MEDICATION CHANGES MADE DURING FOLLOW-UP VISITS

Medication	Type of Change*	Dose, frequency	Date started	Date discontinued	State month of follow-up when medication change was made	Please che (x) here i medication i the DP Lis

** 1=Substitution of Medication 2=Increase in dose of medication 3=Decrease in dose of medication 4=Withdrawal of medication*

GAAP Clinical Data for non-scheduled visits

Location and Date	Response	Code
Name of physician/prescriber		
Institution/ Facility ID		
Patient Name		
Date of visit (eg 01/JAN/2014)	[___]/[___]/[___]<br>dd mm yyyy	

VITAL SIGNS

Weight (in kg)	
Pulse	
Systolic Blood Pressure (mm Hg)	
Diastolic Blood Pressure (mm Hg)	
Fasting Blood Sugar (only for diabetics)	

1. Please state the reason for the unscheduled visit to the prescriber?

2. Has the patient developed complications related to Diabetes and or hypertension since their last visit?

   Yes [ ] No [ ]

3. Use checklist below

	Tick (X) for yes or (O) for no
Peripheral Neuropathy	
CVD events*	
CVA	
Ulcers	
Renal failure	
Diabetic foot infection	
Amputations	
Other	

- *CVD events: acute MI, unstable angina, cardiac mortality.*

4. Was patient hospitalized since last visit? Yes [ ] No [ ]

5. If Yes to question 4, please state the reason for hospitalization?

6. Are there any adverse events due to medications patient is taking? Yes [ ] No [ ]

7. If yes, to question 6 , please state the side effect(s) below?

Medication	Side effect	Date started	Grade (NIH scale)*

** Indicate if side effects are mild (grade 1), moderate (grade 2), severe (grade 3), life threatening (grade 4) or fatal (grade 5)*

8. Medication changes during this visit? Yes ☐ No ☐

9. If yes to question 8, list new medications prescribed.

MEDICATION CHANGES MADE DURING FOLLOW-UP VISITS

Medication	Type of Change*	Dose, frequency	Date started	Date discontinued	State month of follow-up when medication change was made	Please che (x) here if medication i the DP Lis

**1=Substitution of Medication 2=Increase in dose of medication 3=Decrease in dose of medication 4=Withdrawal of medication*

GAAP Clinical Data for non-scheduled visits

Location and Date	Response	Code
Name of physician/prescriber		
Institution/ Facility ID		
Patient Name		
Date of visit (eg 01/JAN/2014)	[___]/[___]/[___]<br>dd mm yyyy	

VITAL SIGNS

Weight (in kg)	
Pulse	
Systolic Blood Pressure (mm Hg)	
Diastolic Blood Pressure (mm Hg)	
Fasting Blood Sugar (only for diabetics)	

1. Please state the reason for the unscheduled visit to the prescriber?

2. Has the patient developed complications related to Diabetes and or hypertension since their last visit?

   Yes No

3. Use checklist below

	Tick (X) for yes or (O) for no
Peripheral Neuropathy	
CVD events*	
CVA	
Ulcers	
Renal failure	
Diabetic foot infection	
Amputations	
Other	

- *CVD events: acute MI, unstable angina, cardiac mortality.*

4. Was patient hospitalized since last visit? Yes [ ] No [ ]

5. If Yes to question 4, please state the reason for hospitalization?

6. Are there any adverse events due to medications patient is taking? Yes [ ] No [ ]

7. If yes, to question 6 , please state the side effect(s) below?

Medication	Side effect	Date started	Grade (NIH scale)*

** Indicate if side effects are mild (grade 1), moderate (grade 2), severe (grade 3), life threatening (grade 4) or fatal (grade 5)*

8. Medication changes during this visit? Yes ☐ No ☐

9. If yes to question 8, list new medications prescribed.

MEDICATION CHANGES MADE DURING FOLLOW-UP VISITS

Medication	Type of Change*	Dose, frequency	Date started	Date discontinued	State month of follow-up when medication change was made	Please che[illegible] (x) here i[illegible] medication i[illegible] the DP Lis[illegible]

** 1=Substitution of Medication 2=Increase in dose of medication 3=Decrease in dose of medication 4=Withdrawal of medication*

GAAP Clinical Data for non-scheduled visits

Location and Date	Response	Code
Name of physician/prescriber		
Institution/ Facility ID		
Patient Name		
Date of visit (eg 01/JAN/2014)	[___]/[___]/[___]<br>dd mm yyyy	

VITAL SIGNS

Weight (in kg)	
Pulse	
Systolic Blood Pressure (mm Hg)	
Diastolic Blood Pressure (mm Hg)	
Fasting Blood Sugar (only for diabetics)	

1. Please state the reason for the unscheduled visit to the prescriber?

2. Has the patient developed complications related to Diabetes and or hypertension since their last visit?

   Yes No

3. Use checklist below

	Tick (X) for yes or (O) for no
Peripheral Neuropathy	
CVD events*	
CVA	
Ulcers	
Renal failure	
Diabetic foot infection	
Amputations	
Other	

• *CVD events: acute MI, unstable angina, cardiac mortality.*

4. Was patient hospitalized since last visit? Yes [ ] No [ ]

5. If Yes to question 4, please state the reason for hospitalization?

6. Are there any adverse events due to medications patient is taking? Yes [ ] No [ ]

7. If yes, to question 6 , please state the side effect(s) below?

Medication	Side effect	Date started	Grade (NIH scale)*

** Indicate if side effects are mild (grade 1), moderate (grade 2), severe (grade 3), life threatening (grade 4) or fatal (grade 5)*

8. Medication changes during this visit? Yes [ ] No [ ]

9. If yes to question 8, list new medications prescribed.

MEDICATION CHANGES MADE DURING FOLLOW-UP VISITS

Medication	Type of Change*	Dose, frequency	Date started	Date discontinued	State month of follow-up when medication change was made	Please che (x) here i medication i the DP Lis

**1=Substitution of Medication 2=Increase in dose of medication 3=Decrease in dose of medication 4=Withdrawal of medication*

PID (Participant ID No.) [ ]-[ ]/[ ]-[ ]-[ ]-[ ]/[ ]-[ ]/[ ]

GAAP Clinical Data for non-scheduled visits

Location and Date	Response	Code
Name of physician/prescriber		
Institution/ Facility ID		
Patient Name		
Date of visit (eg 01/JAN/2014)	[___]/[___]/[___]<br>dd mm yyyy	

VITAL SIGNS

Weight (in kg)	
Pulse	
Systolic Blood Pressure (mm Hg)	
Diastolic Blood Pressure (mm Hg)	
Fasting Blood Sugar (only for diabetics)	

1. Please state the reason for the unscheduled visit to the prescriber?

2. Has the patient developed complications related to Diabetes and or hypertension since their last visit?

   Yes [ ] No [ ]

3. Use checklist below

	Tick (X) for yes or (O) for no
Peripheral Neuropathy	
CVD events*	
CVA	
Ulcers	
Renal failure	
Diabetic foot infection	
Amputations	
Other	

- *CVD events: acute MI, unstable angina, cardiac mortality.*

4. Was patient hospitalized since last visit? Yes [ ] No [ ]

5. If Yes to question 4, please state the reason for hospitalization?

6. Are there any adverse events due to medications patient is taking? Yes [ ] No [ ]

7. If yes, to question 6 , please state the side effect(s) below?

Medication	Side effect	Date started	Grade (NIH scale)*

** Indicate if side effects are mild (grade 1), moderate (grade 2), severe (grade 3), life threatening (grade 4) or fatal (grade 5)*

8. Medication changes during this visit? Yes ☐ No ☐

9. If yes to question 8, list new medications prescribed.

MEDICATION CHANGES MADE DURING FOLLOW-UP VISITS

Medication	Type of Change*	Dose, frequency	Date started	Date discontinued	State month of follow-up when medication change was made	Please che (x) here i medication i the DP Lis

**1=Substitution of Medication 2=Increase in dose of medication 3=Decrease in dose of medication 4=Withdrawal of medication*

GAAP Clinical Data for non-scheduled visits

Location and Date	Response	Code
Name of physician/prescriber		
Institution/ Facility ID		
Patient Name		
Date of visit (eg 01/JAN/2014)	[___]/[___]/[___]<br>dd mm yyyy	

VITAL SIGNS

Weight (in kg)	
Pulse	
Systolic Blood Pressure (mm Hg)	
Diastolic Blood Pressure (mm Hg)	
Fasting Blood Sugar (only for diabetics)	

1. Please state the reason for the unscheduled visit to the prescriber?

2. Has the patient developed complications related to Diabetes and or hypertension since their last visit?

   Yes [ ] No [ ]

3. Use checklist below

	Tick (X) for yes or (O) for no
Peripheral Neuropathy	
CVD events*	
CVA	
Ulcers	
Renal failure	
Diabetic foot infection	
Amputations	
Other	

- *CVD events: acute MI, unstable angina, cardiac mortality.*

4. Was patient hospitalized since last visit? Yes [ ] No [ ]

5. If Yes to question 4, please state the reason for hospitalization?

6. Are there any adverse events due to medications patient is taking? Yes [ ] No [ ]

7. If yes, to question 6 , please state the side effect(s) below?

Medication	Side effect	Date started	Grade (NIH scale)*

** Indicate if side effects are mild (grade 1), moderate (grade 2), severe (grade 3), life threatening (grade 4) or fatal (grade 5)*

8. Medication changes during this visit? Yes ☐ No ☐

9. If yes to question 8, list new medications prescribed.

MEDICATION CHANGES MADE DURING FOLLOW-UP VISITS

Medication	Type of Change*	Dose, frequency	Date started	Date discontinued	State month of follow-up when medication change was made	Please chec (x) here if medication is the DP List

**1=Substitution of Medication 2=Increase in dose of medication 3=Decrease in dose of medication 4=Withdrawal of medication*

GHANA ACCESS AND AFFORDABILITY PROGRAM PILOT STUDY

FOLLOW-UP QUESTIONNAIRE (MONTH 6)

SECTION 1:HEALTH EXPENDITURE		
Follow-up Date (DD-MM-YYYY, eg: 01/JAN/2014)		[_] [_]-[_] [_]-[_] [_][_] [_]
Question		RESPONSE
Q1	Are all of the medicines prescribed by your physician paid for by NHIS? (PLEASE CIRCLE THE CORRECT RESPONSE)	Yes.......1 (Q3)<br>No.......2 (Q2)
Q2	How much do you spend on purchasing medicines for high blood pressure, diabetes (or both) each month?	GH¢|__|__|__|__|. |__|__| CEDIS PESEWAS
Q3	On average, how much does it cost you to travel to this clinic per day (both ways)? (PLEASE INCLUDE YOURSELF AND CAREGIVER IF THIS APPLIES)	GH¢|__|__|__|__|. |__|__| CEDIS PESEWAS
Q4	How much money can you contribute towards your health care expenses each month?	GH¢|__|__|__|__|. |__|__| CEDIS PESEWAS
Q5	If you needed to buy additional medicines for your condition, would you?	YES.......1<br>NO.......2

SECTION 2: MEDICATION ADHERENCE		
Question		(PLEASE CIRCLE THE CORRECT RESPONSE)
R1	In the past two weeks, did you forget or miss your medicines for one or more days?	YES.......1<br>NO.......2
R2	What were the reasons you did not take your prescribed medicines on some days in the past month? (SELECT ALL THAT APPLY)	TOO EXPENSIVE.......1<br>I FORGET.......2<br>SIDE EFFECTS.......3<br>RAN OUT.......4<br>OTHER SPECIFY.......5<br>________________
R3	Did a doctor or health care worker tell you to stop taking a medicine because of a side effect?	YES.......1<br>NO.......2

3. HYPERTENSION TREATMENT ADHERENCE (ONLY FOR PATIENTS WITH HYPERTENSION)		
Question		(PLEASE CIRCLE THE CORRECT RESPONSE)
S1	How often did you forget to take your High Blood Pressure medicine in the past two weeks? (CHOOSE ONE)	ALL THE TIME (14/14 DAYS)........1
		MOST OF THE TIME (10/14 DAYS)........2
		SOME OF THE TIME (7/14 DAYS)........3
		NEVER (0/14 DAYS)........4
S2	How often did you decide not to take your High Blood Pressure medicine in the past two weeks? (CHOOSE ONE)	ALL THE TIME (14/14 DAYS)........1
		MOST OF THE TIME (10/14 DAYS)........2
		SOME OF THE TIME (7/14 DAYS)........3
		NEVER (0/14 DAYS)........4
S3	How often do you eat salty food? (CHOOSE ONE)	ALL THE TIME (30/30 DAYS)........1
		MOST OF THE TIME (24/30 DAYS)........2
		SOME OF THE TIME (15/30 DAYS)........3
		NEVER (0/30 DAYS)........4
S4	How often do you add salt to your food before you eat it? (CHOOSE ONE)	ALL THE TIME (30/30 DAYS)........1
		MOST OF THE TIME (24/30 DAYS)........2
		SOME OF THE TIME (15/30 DAYS)........3
		NEVER (0/30 DAYS)........4
S5	How often do you eat fast food (food purchased for take-out)? (CHOOSE ONE)	ALL THE TIME (30/30 DAYS)........1
		MOST OF THE TIME (24/30 DAYS)........2
		SOME OF THE TIME (15/30 DAYS)........3
		NEVER (0/30 DAYS)........4
S6	How often does the clinic make scheduled return appointments for you before you leave? Over the last 3 visits (CHOOSE ONE)	ALL THE TIME ((3/3) (100% OF THE TIME)........1
		MOST OF THE TIME (2/3) (66% OF THE TIME)........2
		SOME OF THE TIME (1/3) (33% OF THE TIME)........3
		NEVER ....4.

S7	How often do you miss scheduled clinic appointments? Over the last 3 visits (CHOOSE ONE)	ALL THE TIME (3/3) (100% OF THE TIME)..........1<br>MOST OF THE TIME (2/3) (66% OF THE TIME)..........2<br>SOME OF THE TIME (1/3) (33% OF THE TIME)..........3<br>NEVER..........4
S8	How often do you forget to get prescriptions refilled? Over the last 3 visits (CHOOSE ONE)	ALL THE TIME (3/3) (100% OF THE TIME)..........1<br>MOST OF THE TIME (2/3) (66% OF THE TIME)..........2<br>SOME OF THE TIME (1/3) (33% OF THE TIME)..........3<br>NEVER..........4
S9	How often do you run out of High Blood Pressure pills? Over the last 3 visits (CHOOSE ONE)	ALL THE TIME (3/3) (100% OF THE TIME)..........1<br>MOST OF THE TIME (2/3) (66% OF THE TIME)..........2<br>SOME OF THE TIME (1/3) (33% OF THE TIME)..........3<br>NEVER..........4
S10	How often to you skip your High Blood Pressure medicine before you go to the doctor? Over the last 3 visits (CHOOSE ONE)	ALL THE TIME (3/3) (100% OF THE TIME)..........1<br>MOST OF THE TIME (2/3) (66% OF THE TIME)..........2<br>SOME OF THE TIME (1/3) (33% OF THE TIME)..........3<br>NEVER..........4
S11	How often do you miss taking your High Blood Pressure pills when you feel sick? (CHOOSE ONE)	ALL THE TIME (30/30 DAYS)..........1<br>MOST OF THE TIME (24/30 DAYS)..........2<br>SOME OF THE TIME (15/30 DAYS)..........3<br>NEVER (0/30 DAYS)..........4
S12	How often do you miss taking your High Blood Pressure pills when you feel well? (CHOOSE ONE)	ALL THE TIME (30/30 DAYS)..........1<br>MOST OF THE TIME (24/30 DAYS)..........2<br>SOME OF THE TIME (15/30 DAYS)..........3<br>NEVER (0/30 DAYS)..........4

S13	How often do you take someone else's High Blood Pressure pills? (CHOOSE ONE)	ALL THE TIME (30/30 DAYS)............1
		MOST OF THE TIME (24/30 DAYS)............2
		SOME OF THE TIME (15/30 DAYS)............3
		NEVER (0/30 DAYS)............4
S14	How often do you miss taking your high Blood Pressure pills when you are careless or forget? (CHOOSE ONE)	ALL THE TIME (30/30 DAYS)............1
		MOST OF THE TIME (24/30 DAYS)............2
		SOME OF THE TIME (15/30 DAYS)............3
		NEVER (0/30 DAYS)............4

**SECTION 4. DIABETES TREATMENT ADHERENCE (ONLY FOR PATIENTS WITH DIABETES)**		
Question		Response
T1	Do you sometimes forget to take your diabetes pills?	YES............1
		NO............2
T2	People sometimes miss taking their medications for reasons other than forgetting. Thinking over the past two weeks, were there any days when you did not take your diabetes medicine?	YES............1
		NO............2
T3	Have you ever cut back or stopped taking your medication without telling your doctor, because you felt worse when you took it?	YES............1
		NO............2
T4	When you travel or leave home, do you sometimes forget to bring your diabetes medication?	YES............1
		NO............2
T5	Did you take your diabetes medicine yesterday?	YES............1
		NO............2
T6	When you feel like your diabetes is under control, do you sometimes stop taking your medicine?	YES............1
		NO............2
T7	When you feel like your diabetes is under control, do you sometimes stop taking your medicine?	YES............1
		NO............2
T8	Taking medication everyday is a real inconvenience for some people. Do you ever feel hassled about sticking to your diabetes treatment plan?	YES............1
		NO............2

SECTION 5: HOSPITALIZATION		
Question		Response
U1	Have you been admitted to the hospital since your last visit?	YES........1 (U2)<br>NO........2 (V1)
U2	If yes, what were you hospitalized for? (CHOOSE ONE)	HYPERTENSION........1<br>DIABETES........2<br>OTHER SPECIFY........3<br>________________

SECTION 6:MEDICINE ACCESS		
Question		(PLEASE CIRCLE THE CORRECT RESPONSE)
V1	Please indicate where you get your medicine for your diabetes or high blood pressure (SELECT ALL THAT APPLY)	HOSPITAL PHARMACY........1<br>COMMUNITY PHARMACY........2<br>LICENSED CHEMICAL SELLER........3<br>OTHER SPECIFY........4<br>REFUSED........88<br>DON'T KNOW........99
V2	Have you had problems getting medicines for your high blood pressure or diabetes in the past 6 months?	YES (HIGH BLOOD PRESSURE)........1<br>YES (DIABETES)........2<br>YES (BOTH HIGH BLOOD PRESSURE AND DIABETES)....3<br>NO TO ALL THE ABOVE........4 (V5)<br>REFUSED........88<br>DON'T KNOW........99
V3	If you had difficulty obtaining your medicine in the past 6 months, please indicate where.	HOSPITAL PHARMACY........1<br>COMMUNITY PHARMACY........2<br>LICENSED CHEMICAL SELLER........3<br>OTHER SPECIFY........4<br>REFUSED........88<br>DON'T KNOW........99

V4	Please specify why it was difficult to obtain your medicine?	NOT AVAILABLE AT POINT OF CARE.........1<br>TOO EXPENSIVE.........2<br>NHIS NOT ACCEPTED AT PHARMACY.........3<br>OTHER SPECIFY.........4<br>____________<br>NOT APPLICABLE.........5<br>REFUSED.........88<br>DON'T KNOW.........99
V5	In the past 6 months, have you ever tried to treat your high blood pressure or diabetes with an alternative method? If yes, choose all that apply (SELECT ALL THAT APPLY)	HERBAL MEDICINE.........1<br>HOMEOPATHY.........2<br>ACUPUNCTURE.........3<br>TRADITIONAL HEALER.........4<br>PRAYER CAMP.........5<br>OTHER SPECIFY.........6<br>____________<br>NONE OF THE ABOVE.........7
V6	In the past 6 months, have you been referred to a Licensed Chemical Seller or Community Pharmacy because your medicine was not available at the Hospital Pharmacy?	Yes.........1<br>No......... 2 (W1)
V7	If yes, please indicate place of referral (where you obtained your medicine)	Community Pharmacy.........1<br>Licensed Chemical Seller.........2<br>Other Specify.........3<br>____________

**SECTION 7: LIFESTYLE ADVICE**		
During the past six months, has a doctor or other health worker advised you to do any of the following?		
W1	Quit using tobacco or don't start	YES.........1<br>NO.........2
W2	Reduce salt in your diet	YES.........1<br>NO.........2
W3	Eat at least five servings of fruit and/or vegetables each day	YES.........1<br>NO.........2
W4	Reduce fat in your diet	YES.........1<br>NO.........2
W5	Start or do more physical activity	YES.........1<br>NO.........2
W6	Maintain a healthy body weight or lose weight	YES.........1<br>NO.........2

SECTION 8. CANCER SCREENING		
During the past six months, has a doctor or other health worker advised you to do any of the following?		
X1	Breast Cancer screening	YES........1<br>NO........2
X2	Colon Cancer screening (fecal blood test)	YES........1<br>NO........2

PID (Participant ID No.) [ ]-[ ]/[ ]-[ ]-[ ]-[ ]/[ ]-[ ]/[ ]

Ghana Access and Affordability Program Pilot Study

Hypertension Knowledge Attitude And Practices (KAP) Questionnaire

6 MONTHS

Location and Date	(PLEASE CIRCLE THE CORRECT RESPONSE)
Name of person completing questionnaire	
Institution/ Facility ID	TAMALE TEACHING HOSPITAL.......1
	KOMFO-ANOKYE TEACHING HOSPITAL.......2
	KORLE-BU TEACHING HOSPITAL.......3
	KING'S MEDICAL CENTRE.......4
	AGOGO PRESBYTERIAN HOSPITAL.......5
	ATUA GOVERNMENT HOSPITAL.......6
Date of completion of the instrument	[___]/[___]/[___] dd mm year

WE ARE INTERESTED IN SOME THINGS ABOUT YOU AND YOUR HEALTH. PLEASE RESPOND TO ALL THE QUESTIONS AS BEST AS YOU CAN. THERE ARE NO RIGHT OR WRONG ANSWERS. THE INFORMATION YOU PROVIDE WILL REMAIN CONFIDENTIAL.		
Question		Please circle the correct response
A1	What does the term hypertension mean? :(SELECT ALL THAT APPLY)	HIGH BLOOD PRESSURE.......1
		HIGH BLOOD SUGAR.......2
		I DON'T KNOW.......3
		OTHER (SPECIFY)
A2	There are two numbers used in measuring blood pressure levels. What should the upper number be?(CHOOSE ONE)	LESS THAN 140.......1
		GREATER THAN 140.......2
		EQUAL TO 140.......3
		I DON'T KNOW.......99
		OTHER (SPECIFY)
A3	What should the lower number be? (CHOOSE ONE)	LESS THAN 90.......1
		GREATER THAN 90.......2
		EQUAL TO 90.......3
		I DON'T KNOW.......99
		OTHER (SPECIFY)

A4	How serious of a health concern has high blood pressure been to you?(CHOOSE ONE)	VERY SERIOUS CONCERN..1<br>SOMEWHAT SERIOUS CONCERN.......................2<br>NOT AT ALL SERIOUS CONCERN.......................3
A5	How important do you think taking medicine is to keep blood pressure under control? (CHOOSE ONE)	VERY IMPORTANT..1<br>SOMEWHAT IMPORTANT...2<br>NOT AT ALL IMPORTANT...3
A6	What is the most important factor(s) in controlling your high blood pressure? (SELECT ALL THAT APPLY)	TAKING MEDICATIONS...1<br>EXERCISING...2<br>LESS STRESS..3<br>QUITTING SMOKING IF YOU ARE SMOKING.............4<br>REDUCING SALT INTAKE IN YOUR DIET...................5<br>REDUCING ALCOHOL INTAKE...............................6<br>LOSING WEIGHT...7<br>ALL OF THE ABOVE...8<br>I DON'T KNOW..99<br>OTHER (SPECIFY)
A7	High blood pressure can increase or worsen :(SELECT ALL THAT APPLY)	THE RISK OF A HEART ATTACK.............................1<br>THE RISK OF STROKE..2<br>THE RISK OF KIDNEY PROBLEMS..........................3<br>ALL THE ABOVE..4<br>I DON'T KNOW..99<br>OTHER (SPECIFY)

PID (Participant ID No.) [ ]-[ ]/[ ]-[ ]-[ ]-[ ]/[ ]-[ ]/[ ]

Ghana Access and Affordability Program Pilot Study

Diabetes Knowledge Attitude and Practices (KAP) Questionnaire

6 MONTHS

Location and Date	(PLEASE CIRCLE THE CORRECT RESPONSE)
Name of person completing questionnaire	
Institution/ Facility ID	TAMALE TEACHING HOSPITAL..........1
	KOMFO-ANOKYE TEACHING HOSPITAL..........2
	KORLE-BU TEACHING HOSPITAL..........3
	KING'S MEDICAL CENTRE..........4
	AGOGO PRESBYTERIAN HOSPITAL..........5
	ATUA GOVERNMENT HOSPITAL..........6
Date of completion of the instrument	[___]/[___]/[___]<br>dd mm year

WE ARE INTERESTED IN SOME THINGS ABOUT YOU AND YOUR HEALTH. PLEASE RESPOND TO ALL THE QUESTIONS AS BEST AS YOU CAN. THERE ARE NO RIGHT OR WRONG ANSWERS. THE INFORMATION YOU PROVIDE WILL REMAIN CONFIDENTIAL.		
Question		(PLEASE CIRCLE THE CORRECT RESPONSE)
A1	Diabetes is a condition in which the body contains:(SELECT ALL THAT APPLY)	A HIGHER LEVEL OF SUGAR IN THE BODY THAN NORMAL..........1
		A LOWER LEVEL OF SUGAR IN THE BODY THAN NORMAL..........2
		EITHER A HIGHER OR A LOWER LEVEL OF SUGAR IN THE BODY THAN NORMAL..........3
		I DON'T KNOW..........4
		OTHER (SPECIFY)

A2	The symptom (s) of diabetes is/are: (SELECT ALL THAT APPLY)	INCREASED FREQUENCY OF URINATION..........1
		INCREASED THIRST AND HUNGER..........2
		INCREASED TIREDNESS..........3
		SLOW HEALING OF WOUNDS..........4
		ALL THE ABOVE..........5
		OTHER (SPECIFY)
A4	In a diabetic patient, high blood pressure can increase or worsen (SELECT ALL THAT APPLY)	THE RISK OF A HEART ATTACK..........1
		THE RISK OF STROKE..........2
		THE RISK OF EYE PROBLEMS..........3
		THE RISK OF KIDNEY PROBLEMS..........4
		ALL THE ABOVE..........5
		I DON'T KNOW..........6
		OTHER (SPECIFY)
A5	The lifestyle modification (s) required for diabetic patients are/is? (SELECT ALL THAT APPLY)	WEIGHT REDUCTION..........1
		STOPPING SMOKING..........2
		STOPPING ALCOHOL INTAKE..........3
		ALL THE ABOVE..........4
		I DON'T KNOW..........5
		OTHER (SPECIFY)
A6	The important factors that help in controlling blood sugar are (SELECT ALL THAT APPLY)	A CONTROLLED AND PLANNED DIET..........1
		REGULAR EXERCISE..........2
		MEDICATION..........3
		ALL THE ABOVE..........4
		NONE..........5
		OTHER (SPECIFY)

A7	A well-balanced diet includes: (SELECT ALL THAT APPLY)	VEGETABLES.....1
		FRUITS.....2
		CARBOHYDRATES.....3
		ALL THE ABOVE.....4
		NONE.....5
		OTHER (SPECIFY)
A8	For proper foot care, a diabetic patient (SELECT ALL THAT APPLY)	SHOULD INSPECT AND WASH THE FEET DAILY.....1
		SHOULD SELECT THE BEST POSSIBLE FOOTWEAR.....2
		SHOULD WALK BAREFOOT INSIDE AND OUTSIDE THE HOUSE.....3
		SHOULD NOT WALK BAREFOOT INSIDE AND OUTSIDE THE HOUSE.....4
		OTHER (SPECIFY)
A9	Upon control of diabetes, the medicines: (SELECT ALL THAT APPLY)	CAN BE STOPPED IMMEDIATELY.....1
		CAN BE STOPPED AFTER ONE MONTH.....2
		SHOULD BE CONTINUED FOR LIFE.....3
		I DON'T KNOW.....99
		OTHER (SPECIFY)
A10	How do you manage low blood sugar symptoms? (SELECT ALL THAT APPLY)	BY TAKING SUGAR.....1
		BY TAKING MEDICINES.....2
		BY TAKING INSULIN.....3
		I DON'T KNOW.....99
		OTHER (SPECIFY)

PID (Participant ID No.) [ ]-[ ]/[ ]-[ ]-[ ]-[ ]/[ ]-[ ]/[ ]

GHANA ACCESS AND AFFORDABILITY PROGRAM PILOT STUDY

FOLLOW-UP QUESTIONNAIRE (MONTH 12)

SECTION 1:HEALTH EXPENDITURE			
Follow-up Date (DD-MM-YYYY, eg: 01/JAN/2014)		[_] [_]-[_] [_]-[_] [_][_] [_]	
Question		RESPONSE	
Q1	Are all of the medicines prescribed by your physician paid for by NHIS? (PLEASE CIRCLE THE CORRECT RESPONSE)	Yes.....1 (Q2)<br>No.....2 (Q3)	
Q2	How much do you spend on purchasing medicines for high blood pressure, diabetes (or both) each month?	GH¢|__|__|__|__|. |__|__| CEDIS PESEWAS	
Q3	On average, how much does it cost you to travel to this clinic per day (both ways)? (PLEASE INCLUDE YOURSELF AND CAREGIVER IF THIS APPLIES)	GH¢|__|__|__|__|. |__|__| CEDIS PESEWAS	
Q4	How much money can you contribute towards your health care expenses each month?	GH¢|__|__|__|__|. |__|__| CEDIS PESEWAS	
Q5	If you needed to buy additional medicines for your condition, would you?	YES.....1<br>NO.....2	

SECTION 2: MEDICATION ADHERENCE		
Question		(PLEASE CIRCLE THE CORRECT RESPONSE)
R1	In the past two weeks, did you forget or miss your medicines for one or more days?	YES.....1<br>NO.....2
R2	What were the reasons you did not take your prescribed medicines on some days in the past month? (SELECT ALL THAT APPLY)	TOO EXPENSIVE.....1<br>I FORGET.....2<br>SIDE EFFECTS.....3<br>RAN OUT.....4<br>OTHER SPECIFY.....5<br>__________
R3	Did a doctor or health care worker tell you to stop taking a medicine because of a side effect?	YES.....1<br>NO.....2

3. HYPERTENSION TREATMENT ADHERENCE (ONLY FOR PATIENTS WITH HYPERTENSION)		
Question		(PLEASE CIRCLE THE CORRECT RESPONSE)
S1	How often did you forget to take your High Blood Pressure medicine in the past two weeks? (CHOOSE ONE)	ALL THE TIME (14/14 DAYS)........1
		MOST OF THE TIME (10/14 DAYS)........2
		SOME OF THE TIME (7/14 DAYS)........3
		NEVER (0/14 DAYS)........4
S2	How often did you decide not to take your High Blood Pressure medicine in the past two weeks? (CHOOSE ONE)	ALL THE TIME (14/14 DAYS)........1
		MOST OF THE TIME (10/14 DAYS)........2
		SOME OF THE TIME (7/14 DAYS)........3
		NEVER (0/14 DAYS)........4
S3	How often do you eat salty food? (CHOOSE ONE)	ALL THE TIME (30/30 DAYS)........1
		MOST OF THE TIME (24/30 DAYS)........2
		SOME OF THE TIME (15/30 DAYS)........3
		NEVER (0/30 DAYS)........4
S4	How often do you add salt to your food before you eat it? (CHOOSE ONE)	ALL THE TIME (30/30 DAYS)........1
		MOST OF THE TIME (24/30 DAYS)........2
		SOME OF THE TIME (15/30 DAYS)........3
		NEVER (0/30 DAYS)........4
S5	How often do you eat fast food (food purchased for take-out)? (CHOOSE ONE)	ALL THE TIME (30/30 DAYS)........1
		MOST OF THE TIME (24/30 DAYS)........2
		SOME OF THE TIME (15/30 DAYS)........3
		NEVER (0/30 DAYS)........4
S6	How often does the clinic make scheduled return appointments for you before you leave? Over the last 3 visits (CHOOSE ONE)	ALL THE TIME ((3/3) (100% OF THE TIME)........1
		MOST OF THE TIME (2/3) (66% OF THE TIME)........2
		SOME OF THE TIME (1/3) (33% OF THE TIME)........3
		NEVER ....4.

S7	How often do you miss scheduled clinic appointments? Over the last 3 visits (CHOOSE ONE)	ALL THE TIME (3/3) (100% OF THE TIME)........1
		MOST OF THE TIME (2/3) (66% OF THE TIME)........2
		SOME OF THE TIME (1/3) (33% OF THE TIME)........3
		NEVER 4
S8	How often do you forget to get prescriptions refilled? Over the last 3 visits (CHOOSE ONE)	ALL THE TIME (3/3) (100% OF THE TIME)........1
		MOST OF THE TIME (2/3) (66% OF THE TIME)........2
		SOME OF THE TIME (1/3) (33% OF THE TIME)........3
		NEVER 4
S9	How often do you run out of High Blood Pressure pills? Over the last 3 visits (CHOOSE ONE)	ALL THE TIME (3/3) (100% OF THE TIME)........1
		MOST OF THE TIME (2/3) (66% OF THE TIME)........2
		SOME OF THE TIME (1/3) (33% OF THE TIME)........3
		NEVER 4
S10	How often to you skip your High Blood Pressure medicine before you go to the doctor? Over the last 3 visits (CHOOSE ONE)	ALL THE TIME (3/3) (100% OF THE TIME)........1
		MOST OF THE TIME (2/3) (66% OF THE TIME)........2
		SOME OF THE TIME (1/3) (33% OF THE TIME)........3
		NEVER 4
S11	How often do you miss taking your High Blood Pressure pills when you feel sick? (CHOOSE ONE)	ALL THE TIME (30/30 DAYS)........1
		MOST OF THE TIME (24/30 DAYS)........2
		SOME OF THE TIME (15/30 DAYS)........3
		NEVER (0/30 DAYS)........4
S12	How often do you miss taking your High Blood Pressure pills when you feel well? (CHOOSE ONE)	ALL THE TIME (30/30 DAYS)........1
		MOST OF THE TIME (24/30 DAYS)........2
		SOME OF THE TIME (15/30 DAYS)........3
		NEVER (0/30 DAYS)........4

S13	How often do you take someone else's High Blood Pressure pills? (CHOOSE ONE)	ALL THE TIME (30/30 DAYS)..........1
		MOST OF THE TIME (24/30 DAYS)..........2
		SOME OF THE TIME (15/30 DAYS)..........3
		NEVER (0/30 DAYS)..........4
S14	How often do you miss taking your high Blood Pressure pills when you are careless or forget? (CHOOSE ONE)	ALL THE TIME (30/30 DAYS)..........1
		MOST OF THE TIME (24/30 DAYS)..........2
		SOME OF THE TIME (15/30 DAYS)..........3
		NEVER (0/30 DAYS)..........4

**SECTION 4. DIABETES TREATMENT ADHERENCE (ONLY FOR PATIENTS WITH DIABETES)**		
Question		Response
T1	Do you sometimes forget to take your diabetes pills?	YES..........1
		NO..........2
T2	People sometimes miss taking their medications for reasons other than forgetting. Thinking over the past two weeks, were there any days when you did not take your diabetes medicine?	YES..........1
		NO..........2
T3	Have you ever cut back or stopped taking your medication without telling your doctor, because you felt worse when you took it?	YES..........1
		NO..........2
T4	When you travel or leave home, do you sometimes forget to bring your diabetes medication?	YES..........1
		NO..........2
T5	Did you take your diabetes medicine yesterday?	YES..........1
		NO..........2
T6	When you feel like your diabetes is under control, do you sometimes stop taking your medicine?	YES..........1
		NO..........2
T7	When you feel like your diabetes is under control, do you sometimes stop taking your medicine?	YES..........1
		NO..........2
T8	Taking medication everyday is a real inconvenience for some people. Do you ever feel hassled about sticking to your diabetes treatment plan?	YES..........1
		NO..........2

SECTION 5: HOSPITALIZATION		
Question		Response
U1	Have you been admitted to the hospital since your last visit?	YES........1 (U2)<br>NO........2 (V1)
U2	If yes, what were you hospitalized for? (CHOOSE ONE)	HYPERTENSION........1<br>DIABETES........2<br>OTHER SPECIFY........3<br>________________

SECTION 6:MEDICINE ACCESS		
Question		(PLEASE CIRCLE THE CORRECT RESPONSE)
V1	Please indicate where you get your medicine for your diabetes or high blood pressure (SELECT ALL THAT APPLY)	HOSPITAL PHARMACY........1<br>COMMUNITY PHARMACY........2<br>LICENSED CHEMICAL SELLER........3<br>OTHER SPECIFY........4<br>REFUSED........88<br>DON'T KNOW........99
V2	Have you had problems getting medicines for your high blood pressure or diabetes in the past 6 months?	YES (HIGH BLOOD PRESSURE)........1<br>YES (DIABETES)........2<br>YES (BOTH HIGH BLOOD PRESSURE AND DIABETES)....3<br>NO TO ALL THE ABOVE........4 (V5)<br>REFUSED........88<br>DON'T KNOW........99
V3	If you had difficulty obtaining your medicine in the past 6 months, please indicate where.	HOSPITAL PHARMACY........1<br>COMMUNITY PHARMACY........2<br>LICENSED CHEMICAL SELLER........3<br>OTHER SPECIFY........4<br>REFUSED........88<br>DON'T KNOW........99

V4	Please specify why it was difficult to obtain your medicine?	NOT AVAILABLE AT POINT OF CARE.........1
		TOO EXPENSIVE.........2
		NHIS NOT ACCEPTED AT PHARMACY.........3
		OTHER SPECIFY.........4 ____________
		NOT APPLICABLE.........5
		REFUSED.........88
		DON'T KNOW.........99
V5	In the past 6 months, have you ever tried to treat your high blood pressure or diabetes with an alternative method? If yes, choose all that apply (SELECT ALL THAT APPLY)	HERBAL MEDICINE.........1
		HOMEOPATHY 2
		ACUPUNCTURE.........3
		TRADITIONAL HEALER.........4
		PRAYER CAMP.........5
		OTHER SPECIFY.........6 ____________
		NONE OF THE ABOVE.........7
V6	In the past 6 months, have you been referred to a Licensed Chemical Seller or Community Pharmacy because your medicine was not available at the Hospital Pharmacy?	Yes.........1
		No......... 2 (W1)
V7	If yes, please indicate place of referral (where you obtained your medicine)	Community Pharmacy.........1
		Licensed Chemical Seller.........2
		Other Specify.........3 ____________

**SECTION 7: LIFESTYLE ADVICE**		
During the past six months, has a doctor or other health worker advised you to do any of the following?		
W1	Quit using tobacco or don't start	YES.........1
		NO.........2
W2	Reduce salt in your diet	YES.........1
		NO.........2
W3	Eat at least five servings of fruit and/or vegetables each day	YES.........1
		NO.........2
W4	Reduce fat in your diet	YES.........1
		NO.........2
W5	Start or do more physical activity	YES.........1
		NO.........2
W6	Maintain a healthy body weight or lose weight	YES.........1
		NO.........2

SECTION 8. CANCER SCREENING		
During the past six months, has a doctor or other health worker advised you to do any of the following?		
X1	Breast Cancer screening	YES.........1<br>NO.........2
X2	Colon Cancer screening (fecal blood test)	YES.........1<br>NO.........2

PID (Participant ID No.) [ ]-[ ]/[ ]-[ ]-[ ]-[ ]/[ ]-[ ]/[ ]

Ghana Access and Affordability Program Pilot Study

Hypertension Knowledge Attitude and Practices (KAP) Questionnaire

12 MONTHS

Location and Date	(PLEASE CIRCLE THE CORRECT RESPONSE)
Name of person completing questionnaire	
Institution/ Facility ID	TAMALE TEACHING HOSPITAL.............................1
	KOMFO-ANOKYE TEACHING HOSPITAL..................2
	KORLE-BU TEACHING HOSPITAL.........................3
	KING'S MEDICAL CENTRE.......................................4
	AGOGO PRESBYTERIAN HOSPITAL.........................5
	ATUA GOVERNMENT HOSPITAL...........................6
Date of completion of the instrument	[___]/[___]/[___]<br>dd mm year

WE ARE INTERESTED IN SOME THINGS ABOUT YOU AND YOUR HEALTH. PLEASE RESPOND TO ALL THE QUESTIONS AS BEST AS YOU CAN. THERE ARE NO RIGHT OR WRONG ANSWERS. THE INFORMATION YOU PROVIDE WILL REMAIN CONFIDENTIAL.		
Question		Please circle the correct response
A1	What does the term hypertension mean? :(SELECT ALL THAT APPLY)	HIGH BLOOD PRESSURE.................................1
		HIGH BLOOD SUGAR.......................................2
		I DON'T KNOW..3
		OTHER (SPECIFY)
A2	There are two numbers used in measuring blood pressure levels. What should the upper number be?(CHOOSE ONE)	LESS THAN 140..1
		GREATER THAN 140...2
		EQUAL TO 140..3
		I DON'T KNOW..99
		OTHER (SPECIFY)
A3	What should the lower number be? (CHOOSE ONE)	LESS THAN 90...1
		GREATER THAN 90..2
		EQUAL TO 90...3
		I DON'T KNOW..99
		OTHER (SPECIFY)

A4	How serious of a health concern has high blood pressure been to you?(CHOOSE ONE)	VERY SERIOUS CONCERN.........1
		SOMEWHAT SERIOUS CONCERN.........2
		NOT AT ALL SERIOUS CONCERN.........3
A5	How important do you think taking medicine is to keep blood pressure under control? (CHOOSE ONE)	VERY IMPORTANT.........1
		SOMEWHAT IMPORTANT.........2
		NOT AT ALL IMPORTANT.........3
A6	What is the most important factor(s) in controlling your high blood pressure? (SELECT ALL THAT APPLY)	TAKING MEDICATIONS.........1
		EXERCISING.........2
		LESS STRESS.........3
		QUITTING SMOKING IF YOU ARE SMOKING.........4
		REDUCING SALT INTAKE IN YOUR DIET.........5
		REDUCING ALCOHOL INTAKE.........6
		LOSING WEIGHT.........7
		ALL OF THE ABOVE.........8
		I DON'T KNOW.........99
		OTHER (SPECIFY)
A7	High blood pressure can increase or worsen :(SELECT ALL THAT APPLY)	THE RISK OF A HEART ATTACK.........1
		THE RISK OF STROKE.........2
		THE RISK OF KIDNEY PROBLEMS.........3
		ALL THE ABOVE.........4
		I DON'T KNOW.........99
		OTHER (SPECIFY)

PID (Participant ID No.) [ ]-[ ]/[ ]-[ ]-[ ]-[ ]/[ ]-[ ]/[ ]

Ghana Access and Affordability Program Pilot Study

Diabetes Knowledge Attitude and Practices (KAP) Questionnaire

12 MONTHS

Location and Date	(PLEASE CIRCLE THE CORRECT RESPONSE)
Name of person completing questionnaire	
Institution/ Facility ID	TAMALE TEACHING HOSPITAL..........1
	KOMFO-ANOKYE TEACHING HOSPITAL..........2
	KORLE-BU TEACHING HOSPITAL..........3
	KING'S MEDICAL CENTRE..........4
	AGOGO PRESBYTERIAN HOSPITAL..........5
	ATUA GOVERNMENT HOSPITAL..........6
Date of completion of the instrument	[___]/[___]/[___]<br>dd mm year

WE ARE INTERESTED IN SOME THINGS ABOUT YOU AND YOUR HEALTH. PLEASE RESPOND TO ALL THE QUESTIONS AS BEST AS YOU CAN. THERE ARE NO RIGHT OR WRONG ANSWERS. THE INFORMATION YOU PROVIDE WILL REMAIN CONFIDENTIAL.		
Question		(PLEASE CIRCLE THE CORRECT RESPONSE)
A1	Diabetes is a condition in which the body contains:(SELECT ALL THAT APPLY)	A HIGHER LEVEL OF SUGAR IN THE BODY THAN NORMAL..........1
		A LOWER LEVEL OF SUGAR IN THE BODY THAN NORMAL..........2
		EITHER A HIGHER OR A LOWER LEVEL OF SUGAR IN THE BODY THAN NORMAL..........3
		I DON'T KNOW..........4
		OTHER (SPECIFY)

A2	The symptom (s) of diabetes is/are: (SELECT ALL THAT APPLY)	INCREASED FREQUENCY OF URINATION......1
		INCREASED THIRST AND HUNGER......2
		INCREASED TIREDNESS......3
		SLOW HEALING OF WOUNDS......4
		ALL THE ABOVE......5
		OTHER (SPECIFY)
A4	In a diabetic patient, high blood pressure can increase or worsen (SELECT ALL THAT APPLY)	THE RISK OF A HEART ATTACK......1
		THE RISK OF STROKE......2
		THE RISK OF EYE PROBLEMS......3
		THE RISK OF KIDNEY PROBLEMS......4
		ALL THE ABOVE......5
		I DON'T KNOW......6
		OTHER (SPECIFY)
A5	The lifestyle modification (s) required for diabetic patients are/is? (SELECT ALL THAT APPLY)	WEIGHT REDUCTION......1
		STOPPING SMOKING......2
		STOPPING ALCOHOL INTAKE......3
		ALL THE ABOVE......4
		I DON'T KNOW......5
		OTHER (SPECIFY)
A6	The important factors that help in controlling blood sugar are (SELECT ALL THAT APPLY)	A CONTROLLED AND PLANNED DIET......1
		REGULAR EXERCISE......2
		MEDICATION......3
		ALL THE ABOVE......4
		NONE......5
		OTHER (SPECIFY)

A7	A well-balanced diet includes: (SELECT ALL THAT APPLY)	VEGETABLES............1
		FRUITS............2
		CARBOHYDRATES............3
		ALL THE ABOVE............4
		NONE............5
		OTHER (SPECIFY)
A8	For proper foot care, a diabetic patient (SELECT ALL THAT APPLY)	SHOULD INSPECT AND WASH THE FEET DAILY............1
		SHOULD SELECT THE BEST POSSIBLE FOOTWEAR............2
		SHOULD WALK BAREFOOT INSIDE AND OUTSIDE THE HOUSE............3
		SHOULD NOT WALK BAREFOOT INSIDE AND OUTSIDE THE HOUSE............4
		OTHER (SPECIFY)
A9	Upon control of diabetes, the medicines: (SELECT ALL THAT APPLY)	CAN BE STOPPED IMMEDIATELY............1
		CAN BE STOPPED AFTER ONE MONTH......2
		SHOULD BE CONTINUED FOR LIFE............3
		I DON'T KNOW............99
		OTHER (SPECIFY)
A10	How do you manage low blood sugar symptoms? (SELECT ALL THAT APPLY)	BY TAKING SUGAR............1
		BY TAKING MEDICINES............2
		BY TAKING INSULIN............3
		I DON'T KNOW............99
		OTHER (SPECIFY)

GHANA ACCESS AND AFFORDABILITY PROGRAM PILOT STUDY

FOLLOW-UP QUESTIONNAIRE (MONTH 18)

SECTION 1:HEALTH EXPENDITURE		
Follow-up Date (DD-MM-YYYY, eg: 01/JAN/2014)		[__] [__]-[__] [__]-[__] [__][__] [__]
Question		RESPONSE
Q1	Are all of the medicines prescribed by your physician paid for by NHIS? (PLEASE CIRCLE THE CORRECT RESPONSE)	Yes......1 (Q2)<br>No......2 (Q3)
Q2	How much do you spend on purchasing medicines for high blood pressure, diabetes (or both) each month?	GH¢|__|__|__|__|. |__|__| CEDIS PESEWAS
Q3	On average, how much does it cost you to travel to this clinic per day (both ways)? (PLEASE INCLUDE YOURSELF AND CAREGIVER IF THIS APPLIES)	GH¢|__|__|__|__|. |__|__| CEDIS PESEWAS
Q4	How much money can you contribute towards your health care expenses each month?	GH¢|__|__|__|__|. |__|__| CEDIS PESEWAS
Q5	If you needed to buy additional medicines for your condition, would you?	YES......1<br>NO......2

SECTION 2: MEDICATION ADHERENCE		
Question		(PLEASE CIRCLE THE CORRECT RESPONSE)
R1	In the past two weeks, did you forget or miss your medicines for one or more days?	YES......1<br>NO......2
R2	What were the reasons you did not take your prescribed medicines on some days in the past month? (SELECT ALL THAT APPLY)	TOO EXPENSIVE......1<br>I FORGET......2<br>SIDE EFFECTS......3<br>RAN OUT......4<br>OTHER SPECIFY......5<br>________________
R3	Did a doctor or health care worker tell you to stop taking a medicine because of a side effect?	YES......1<br>NO......2

3. HYPERTENSION TREATMENT ADHERENCE (ONLY FOR PATIENTS WITH HYPERTENSION)		
Question		(PLEASE CIRCLE THE CORRECT RESPONSE)
S1	How often did you forget to take your High Blood Pressure medicine in the past two weeks? (CHOOSE ONE)	ALL THE TIME (14/14 DAYS)........1
		MOST OF THE TIME (10/14 DAYS)........2
		SOME OF THE TIME (7/14 DAYS)........3
		NEVER (0/14 DAYS)........4
S2	How often did you decide not to take your High Blood Pressure medicine in the past two weeks? (CHOOSE ONE)	ALL THE TIME (14/14 DAYS)........1
		MOST OF THE TIME (10/14 DAYS)........2
		SOME OF THE TIME (7/14 DAYS)........3
		NEVER (0/14 DAYS)........4
S3	How often do you eat salty food? (CHOOSE ONE)	ALL THE TIME (30/30 DAYS)........1
		MOST OF THE TIME (24/30 DAYS)........2
		SOME OF THE TIME (15/30 DAYS)........3
		NEVER (0/30 DAYS)........4
S4	How often do you add salt to your food before you eat it? (CHOOSE ONE)	ALL THE TIME (30/30 DAYS)........1
		MOST OF THE TIME (24/30 DAYS)........2
		SOME OF THE TIME (15/30 DAYS)........3
		NEVER (0/30 DAYS)........4
S5	How often do you eat fast food (food purchased for take-out)? (CHOOSE ONE)	ALL THE TIME (30/30 DAYS)........1
		MOST OF THE TIME (24/30 DAYS)........2
		SOME OF THE TIME (15/30 DAYS)........3
		NEVER (0/30 DAYS)........4
S6	How often does the clinic make scheduled return appointments for you before you leave? Over the last 3 visits (CHOOSE ONE)	ALL THE TIME ((3/3)<br>(100% OF THE TIME)........1
		MOST OF THE TIME (2/3)<br>(66% OF THE TIME)........2
		SOME OF THE TIME (1/3)<br>(33% OF THE TIME)........3
		NEVER ....4.

S7	How often do you miss scheduled clinic appointments? Over the last 3 visits (CHOOSE ONE)	ALL THE TIME (3/3) (100% OF THE TIME)....1
		MOST OF THE TIME (2/3) (66% OF THE TIME)....2
		SOME OF THE TIME (1/3) (33% OF THE TIME)....3
		NEVER 4
S8	How often do you forget to get prescriptions refilled? Over the last 3 visits (CHOOSE ONE)	ALL THE TIME (3/3) (100% OF THE TIME)....1
		MOST OF THE TIME (2/3) (66% OF THE TIME)....2
		SOME OF THE TIME (1/3) (33% OF THE TIME)....3
		NEVER 4
S9	How often do you run out of High Blood Pressure pills? Over the last 3 visits (CHOOSE ONE)	ALL THE TIME (3/3) (100% OF THE TIME)....1
		MOST OF THE TIME (2/3) (66% OF THE TIME)....2
		SOME OF THE TIME (1/3) (33% OF THE TIME)....3
		NEVER 4
S10	How often to you skip your High Blood Pressure medicine before you go to the doctor? Over the last 3 visits (CHOOSE ONE)	ALL THE TIME (3/3) (100% OF THE TIME)....1
		MOST OF THE TIME (2/3) (66% OF THE TIME)....2
		SOME OF THE TIME (1/3) (33% OF THE TIME)....3
		NEVER 4
S11	How often do you miss taking your High Blood Pressure pills when you feel sick? (CHOOSE ONE)	ALL THE TIME (30/30 DAYS)....1
		MOST OF THE TIME (24/30 DAYS)....2
		SOME OF THE TIME (15/30 DAYS)....3
		NEVER (0/30 DAYS)....4
S12	How often do you miss taking your High Blood Pressure pills when you feel well? (CHOOSE ONE)	ALL THE TIME (30/30 DAYS)....1
		MOST OF THE TIME (24/30 DAYS)....2
		SOME OF THE TIME (15/30 DAYS)....3
		NEVER (0/30 DAYS)....4

S13	How often do you take someone else's High Blood Pressure pills? (CHOOSE ONE)	ALL THE TIME (30/30 DAYS)............1
		MOST OF THE TIME (24/30 DAYS)............2
		SOME OF THE TIME (15/30 DAYS)............3
		NEVER (0/30 DAYS)............4
S14	How often do you miss taking your high Blood Pressure pills when you are careless or forget? (CHOOSE ONE)	ALL THE TIME (30/30 DAYS)............1
		MOST OF THE TIME (24/30 DAYS)............2
		SOME OF THE TIME (15/30 DAYS)............3
		NEVER (0/30 DAYS)............4

**SECTION 4. DIABETES TREATMENT ADHERENCE (ONLY FOR PATIENTS WITH DIABETES)**		
Question		Response
T1	Do you sometimes forget to take your diabetes pills?	YES............1
		NO............2
T2	People sometimes miss taking their medications for reasons other than forgetting. Thinking over the past two weeks, were there any days when you did not take your diabetes medicine?	YES............1
		NO............2
T3	Have you ever cut back or stopped taking your medication without telling your doctor, because you felt worse when you took it?	YES............1
		NO............2
T4	When you travel or leave home, do you sometimes forget to bring your diabetes medication?	YES............1
		NO............2
T5	Did you take your diabetes medicine yesterday?	YES............1
		NO............2
T6	When you feel like your diabetes is under control, do you sometimes stop taking your medicine?	YES............1
		NO............2
T7	When you feel like your diabetes is under control, do you sometimes stop taking your medicine?	YES............1
		NO............2
T8	Taking medication everyday is a real inconvenience for some people. Do you ever feel hassled about sticking to your diabetes treatment plan?	YES............1
		NO............2

SECTION 5: HOSPITALIZATION		
Question		Response
U1	Have you been admitted to the hospital since your last visit?	YES......1 (U2)<br>NO......2 (V1)
U2	If yes, what were you hospitalized for? (CHOOSE ONE)	HYPERTENSION......1<br>DIABETES......2<br>OTHER SPECIFY......3<br>__________

SECTION 6:MEDICINE ACCESS		
Question		(PLEASE CIRCLE THE CORRECT RESPONSE)
V1	Please indicate where you get your medicine for your diabetes or high blood pressure (SELECT ALL THAT APPLY)	HOSPITAL PHARMACY......1<br>COMMUNITY PHARMACY......2<br>LICENSED CHEMICAL SELLER......3<br>OTHER SPECIFY......4<br>REFUSED......88<br>DON'T KNOW......99
V2	Have you had problems getting medicines for your high blood pressure or diabetes in the past 6 months?	YES (HIGH BLOOD PRESSURE)......1<br>YES (DIABETES)......2<br>YES (BOTH HIGH BLOOD PRESSURE AND DIABETES)....3<br>NO TO ALL THE ABOVE......4 (V5)<br>REFUSED......88<br>DON'T KNOW......99
V3	If you had difficulty obtaining your medicine in the past 6 months, please indicate where.	HOSPITAL PHARMACY......1<br>COMMUNITY PHARMACY......2<br>LICENSED CHEMICAL SELLER......3<br>OTHER SPECIFY......4<br>REFUSED......88<br>DON'T KNOW......99

V4	Please specify why it was difficult to obtain your medicine?	NOT AVAILABLE AT POINT OF CARE........1
		TOO EXPENSIVE........2
		NHIS NOT ACCEPTED AT PHARMACY........3
		OTHER SPECIFY........4<br>____________________
		NOT APPLICABLE........5
		REFUSED........88
		DON'T KNOW........99
V5	In the past 6 months, have you ever tried to treat your high blood pressure or diabetes with an alternative method? If yes, choose all that apply (SELECT ALL THAT APPLY)	HERBAL MEDICINE........1
		HOMEOPATHY 2
		ACUPUNCTURE........3
		TRADITIONAL HEALER........4
		PRAYER CAMP........5
		OTHER SPECIFY........6<br>____________________
		NONE OF THE ABOVE........7
V6	In the past 6 months, have you been referred to a Licensed Chemical Seller or Community Pharmacy because your medicine was not available at the Hospital Pharmacy?	Yes........1
		No........ 2 (W1)
V7	If yes, please indicate place of referral (where you obtained your medicine)	Community Pharmacy........1
		Licensed Chemical Seller........2
		Other Specify........3<br>____________________

**SECTION 7: LIFESTYLE ADVICE**		
During the past six months, has a doctor or other health worker advised you to do any of the following?		
W1	Quit using tobacco or don't start	YES........1
		NO........2
W2	Reduce salt in your diet	YES........1
		NO........2
W3	Eat at least five servings of fruit and/or vegetables each day	YES........1
		NO........2
W4	Reduce fat in your diet	YES........1
		NO........2
W5	Start or do more physical activity	YES........1
		NO........2
W6	Maintain a healthy body weight or lose weight	YES........1
		NO........2

SECTION 8. CANCER SCREENING		
During the past six months, has a doctor or other health worker advised you to do any of the following?		
X1	Breast Cancer screening	YES....................1<br>NO....................2
X2	Colon Cancer screening (fecal blood test)	YES....................1<br>NO....................2

PID (Participant ID No.) ☐-☐/☐-☐-☐-☐/☐-☐/☐

Ghana Access and Affordability Program Pilot Study

Hypertension Knowledge Attitude and Practices (KAP) Questionnaire

18 MONTHS

Location and Date	(PLEASE CIRCLE THE CORRECT RESPONSE)
Name of person completing questionnaire	
Institution/ Facility ID	TAMALE TEACHING HOSPITAL..........1
	KOMFO-ANOKYE TEACHING HOSPITAL..........2
	KORLE-BU TEACHING HOSPITAL..........3
	KING'S MEDICAL CENTRE..........4
	AGOGO PRESBYTERIAN HOSPITAL..........5
	ATUA GOVERNMENT HOSPITAL..........6
Date of completion of the instrument	[___]/[___]/[___]<br>dd mm year

WE ARE INTERESTED IN SOME THINGS ABOUT YOU AND YOUR HEALTH. PLEASE RESPOND TO ALL THE QUESTIONS AS BEST AS YOU CAN. THERE ARE NO RIGHT OR WRONG ANSWERS. THE INFORMATION YOU PROVIDE WILL REMAIN CONFIDENTIAL.

Question		Please circle the correct response
A1	What does the term hypertension mean? (SELECT ALL THAT APPLY)	HIGH BLOOD PRESSURE..........1
		HIGH BLOOD SUGAR..........2
		I DON'T KNOW..........3
		OTHER (SPECIFY)
A2	There are two numbers used in measuring blood pressure levels. What should the upper number be?(CHOOSE ONE)	LESS THAN 140..........1
		GREATER THAN 140..........2
		EQUAL TO 140..........3
		I DON'T KNOW..........99
		OTHER (SPECIFY)

A3	What should the lower number be? (CHOOSE ONE)	LESS THAN 90.........1<br>GREATER THAN 90.........2<br>EQUAL TO 90.........3<br>I DON'T KNOW.........99<br>OTHER (SPECIFY)
A4	How serious of a health concern has high blood pressure been to you?(CHOOSE ONE)	VERY SERIOUS CONCERN.........1<br>SOMEWHAT SERIOUS CONCERN.........2<br>NOT AT ALL SERIOUS CONCERN.........3
A5	How important do you think taking medicine is to keep blood pressure under control? (CHOOSE ONE)	VERY IMPORTANT.........1<br>SOMEWHAT IMPORTANT.........2<br>NOT AT ALL IMPORTANT.........3
A6	What is the most important factor(s) in controlling your high blood pressure? (SELECT ALL THAT APPLY)	TAKING MEDICATIONS.........1<br>EXERCISING.........2<br>LESS STRESS.........3<br>QUITTING SMOKING IF YOU ARE SMOKING.........4<br>REDUCING SALT INTAKE IN YOUR DIET......5<br>REDUCING ALCOHOL INTAKE.........6<br>LOSING WEIGHT.........7<br>ALL OF THE ABOVE.........8<br>I DON'T KNOW.........99<br>OTHER (SPECIFY)
A7	High blood pressure can increase or worsen (SELECT ALL THAT APPLY)	THE RISK OF A HEART ATTACK.........1<br>THE RISK OF STROKE.........2<br>THE RISK OF KIDNEY PROBLEMS.........3<br>ALL THE ABOVE.........4<br>I DON'T KNOW.........99<br>OTHER (SPECIFY)

Ghana Access and Affordability Program Pilot Study

Diabetes Knowledge Attitude and Practices (KAP) Questionnaire

18 MONTHS

Location and Date	(PLEASE CIRCLE THE CORRECT RESPONSE)
Name of person completing questionnaire	
Institution/ Facility ID	TAMALE TEACHING HOSPITAL..1
	KOMFO-ANOKYE TEACHING HOSPITAL................2
	KORLE-BU TEACHING HOSPITAL..................................3
	KING'S MEDICAL CENTRE...4
	AGOGO PRESBYTERIAN HOSPITAL............................5
	ATUA GOVERNMENT HOSPITAL..................................6
Date of completion of the instrument	[___]/[___]/[___]<br>dd mm year

WE ARE INTERESTED IN SOME THINGS ABOUT YOU AND YOUR HEALTH. PLEASE RESPOND TO ALL THE QUESTIONS AS BEST AS YOU CAN. THERE ARE NO RIGHT OR WRONG ANSWERS. THE INFORMATION YOU PROVIDE WILL REMAIN CONFIDENTIAL.		
Question		(PLEASE CIRCLE THE CORRECT RESPONSE)
A1	Diabetes is a condition in which the body contains:(SELECT ALL THAT APPLY)	A HIGHER LEVEL OF SUGAR IN THE BODY THAN NORMAL..1
		A LOWER LEVEL OF SUGAR IN THE BODY THAN NORMAL..2
		EITHER A HIGHER OR A LOWER LEVEL OF SUGAR IN THE BODY THAN NORMAL...3
		I DON'T KNOW..4
		OTHER (SPECIFY)

A2	The symptom (s) of diabetes is/are: (SELECT ALL THAT APPLY)	INCREASED FREQUENCY OF URINATION.........1
		INCREASED THIRST AND HUNGER.........2
		INCREASED TIREDNESS.........3
		SLOW HEALING OF WOUNDS.........4
		ALL THE ABOVE.........5
		OTHER (SPECIFY)
A4	In a diabetic patient, high blood pressure can increase or worsen (SELECT ALL THAT APPLY)	THE RISK OF A HEART ATTACK.........1
		THE RISK OF STROKE.........2
		THE RISK OF EYE PROBLEMS.........3
		THE RISK OF KIDNEY PROBLEMS.........4
		ALL THE ABOVE.........5
		I DON'T KNOW.........6
		OTHER (SPECIFY)
A5	The lifestyle modification (s) required for diabetic patients are/is? (SELECT ALL THAT APPLY)	WEIGHT REDUCTION.........1
		STOPPING SMOKING.........2
		STOPPING ALCOHOL INTAKE.........3
		ALL THE ABOVE.........4
		I DON'T KNOW.........5
		OTHER (SPECIFY)
A6	The important factors that help in controlling blood sugar are (SELECT ALL THAT APPLY)	A CONTROLLED AND PLANNED DIET.........1
		REGULAR EXERCISE.........2
		MEDICATION.........3
		ALL THE ABOVE.........4
		NONE.........5
		OTHER (SPECIFY)

A7	A well-balanced diet includes: (SELECT ALL THAT APPLY)	VEGETABLES.......1
		FRUITS.......2
		CARBOHYDRATES.......3
		ALL THE ABOVE.......4
		NONE.......5
		OTHER (SPECIFY)
A8	For proper foot care, a diabetic patient (SELECT ALL THAT APPLY)	SHOULD INSPECT AND WASH THE FEET DAILY.......1
		SHOULD SELECT THE BEST POSSIBLE FOOTWEAR.......2
		SHOULD WALK BAREFOOT INSIDE AND OUTSIDE THE HOUSE.......3
		SHOULD NOT WALK BAREFOOT INSIDE AND OUTSIDE THE HOUSE.......4
		OTHER (SPECIFY)
A9	Upon control of diabetes, the medicines: (SELECT ALL THAT APPLY)	CAN BE STOPPED IMMEDIATELY.......1
		CAN BE STOPPED AFTER ONE MONTH.......2
		SHOULD BE CONTINUED FOR LIFE.......3
		I DON'T KNOW.......99
		OTHER (SPECIFY)
A10	How do you manage low blood sugar symptoms? (SELECT ALL THAT APPLY)	BY TAKING SUGAR.......1
		BY TAKING MEDICINES.......2
		BY TAKING INSULIN.......3
		I DON'T KNOW.......99
		OTHER (SPECIFY)

GHANA ACCESS AND AFFORDABILITY PROGRAM PILOT STUDY

FOLLOW-UP QUESTIONNAIRE (MONTH 24)

SECTION 1:HEALTH EXPENDITURE		
Follow-up Date (DD-MM-YYYY, eg: 01/JAN/2014)		[_] [_]-[_] [_]-[_] [_][_] [_]
Question		RESPONSE
Q1	Are all of the medicines prescribed by your physician paid for by NHIS? (PLEASE CIRCLE THE CORRECT RESPONSE)	Yes.......1 (Q3)<br>No.......2 (Q2)
Q2	How much do you spend on purchasing medicines for high blood pressure, diabetes (or both) each month?	GH¢|__|__|__|__|. |__|__| CEDIS PESEWAS
Q3	On average, how much does it cost you to travel to this clinic per day (both ways)? (PLEASE INCLUDE YOURSELF AND CAREGIVER IF THIS APPLIES)	GH¢|__|__|__|__|. |__|__| CEDIS PESEWAS
Q4	How much money can you contribute towards your health care expenses each month?	GH¢|__|__|__|__|. |__|__| CEDIS PESEWAS
Q5	If you needed to buy additional medicines for your condition, would you?	YES.......1<br>NO.......2

SECTION 2: MEDICATION ADHERENCE		
Question		(PLEASE CIRCLE THE CORRECT RESPONSE)
R1	In the past two weeks, did you forget or miss your medicines for one or more days?	YES.......1<br>NO.......2
R2	What were the reasons you did not take your prescribed medicines on some days in the past month? (SELECT ALL THAT APPLY)	TOO EXPENSIVE.......1<br>I FORGET.......2<br>SIDE EFFECTS.......3<br>RAN OUT.......4<br>OTHER SPECIFY.......5<br>________________
R3	Did a doctor or health care worker tell you to stop taking a medicine because of a side effect?	YES.......1<br>NO.......2

3. HYPERTENSION TREATMENT ADHERENCE (ONLY FOR PATIENTS WITH HYPERTENSION)		
Question		(PLEASE CIRCLE THE CORRECT RESPONSE)
S1	How often did you forget to take your High Blood Pressure medicine in the past two weeks? (CHOOSE ONE)	ALL THE TIME (14/14 DAYS)........1
		MOST OF THE TIME (10/14 DAYS)........2
		SOME OF THE TIME (7/14 DAYS)........3
		NEVER (0/14 DAYS)........4
S2	How often did you decide not to take your High Blood Pressure medicine in the past two weeks? (CHOOSE ONE)	ALL THE TIME (14/14 DAYS)........1
		MOST OF THE TIME (10/14 DAYS)........2
		SOME OF THE TIME (7/14 DAYS)........3
		NEVER (0/14 DAYS)........4
S3	How often do you eat salty food? (CHOOSE ONE)	ALL THE TIME (30/30 DAYS)........1
		MOST OF THE TIME (24/30 DAYS)........2
		SOME OF THE TIME (15/30 DAYS)........3
		NEVER (0/30 DAYS)........4
S4	How often do you add salt to your food before you eat it? (CHOOSE ONE)	ALL THE TIME (30/30 DAYS)........1
		MOST OF THE TIME (24/30 DAYS)........2
		SOME OF THE TIME (15/30 DAYS)........3
		NEVER (0/30 DAYS)........4
S5	How often do you eat fast food (food purchased for take-out)? (CHOOSE ONE)	ALL THE TIME (30/30 DAYS)........1
		MOST OF THE TIME (24/30 DAYS)........2
		SOME OF THE TIME (15/30 DAYS)........3
		NEVER (0/30 DAYS)........4
S6	How often does the clinic make scheduled return appointments for you before you leave? Over the last 3 visits (CHOOSE ONE)	ALL THE TIME ((3/3) (100% OF THE TIME)........1
		MOST OF THE TIME (2/3) (66% OF THE TIME)........2
		SOME OF THE TIME (1/3) (33% OF THE TIME)........3
		NEVER ....4.

S7	How often do you miss scheduled clinic appointments? Over the last 3 visits (CHOOSE ONE)	ALL THE TIME (3/3) (100% OF THE TIME)........1
		MOST OF THE TIME (2/3) (66% OF THE TIME)........2
		SOME OF THE TIME (1/3) (33% OF THE TIME)........3
		NEVER 4
S8	How often do you forget to get prescriptions refilled? Over the last 3 visits (CHOOSE ONE)	ALL THE TIME (3/3) (100% OF THE TIME)........1
		MOST OF THE TIME (2/3) (66% OF THE TIME)........2
		SOME OF THE TIME (1/3) (33% OF THE TIME)........3
		NEVER 4
S9	How often do you run out of High Blood Pressure pills? Over the last 3 visits (CHOOSE ONE)	ALL THE TIME (3/3) (100% OF THE TIME)........1
		MOST OF THE TIME (2/3) (66% OF THE TIME)........2
		SOME OF THE TIME (1/3) (33% OF THE TIME)........3
		NEVER 4
S10	How often to you skip your High Blood Pressure medicine before you go to the doctor? Over the last 3 visits (CHOOSE ONE)	ALL THE TIME (3/3) (100% OF THE TIME)........1
		MOST OF THE TIME (2/3) (66% OF THE TIME)........2
		SOME OF THE TIME (1/3) (33% OF THE TIME)........3
		NEVER 4
S11	How often do you miss taking your High Blood Pressure pills when you feel sick? (CHOOSE ONE)	ALL THE TIME (30/30 DAYS)........1
		MOST OF THE TIME (24/30 DAYS)........2
		SOME OF THE TIME (15/30 DAYS)........3
		NEVER (0/30 DAYS)........4
S12	How often do you miss taking your High Blood Pressure pills when you feel well? (CHOOSE ONE)	ALL THE TIME (30/30 DAYS)........1
		MOST OF THE TIME (24/30 DAYS)........2
		SOME OF THE TIME (15/30 DAYS)........3
		NEVER (0/30 DAYS)........4

S13	How often do you take someone else's High Blood Pressure pills? (CHOOSE ONE)	ALL THE TIME (30/30 DAYS)..........1
		MOST OF THE TIME (24/30 DAYS)..........2
		SOME OF THE TIME (15/30 DAYS)..........3
		NEVER (0/30 DAYS)..........4
S14	How often do you miss taking your high Blood Pressure pills when you are careless or forget? (CHOOSE ONE)	ALL THE TIME (30/30 DAYS)..........1
		MOST OF THE TIME (24/30 DAYS)..........2
		SOME OF THE TIME (15/30 DAYS)..........3
		NEVER (0/30 DAYS)..........4

SECTION 4. DIABETES TREATMENT ADHERENCE (ONLY FOR PATIENTS WITH DIABETES)		
Question		Response
T1	Do you sometimes forget to take your diabetes pills?	YES..........1
		NO..........2
T2	People sometimes miss taking their medications for reasons other than forgetting. Thinking over the past two weeks, were there any days when you did not take your diabetes medicine?	YES..........1
		NO..........2
T3	Have you ever cut back or stopped taking your medication without telling your doctor, because you felt worse when you took it?	YES..........1
		NO..........2
T4	When you travel or leave home, do you sometimes forget to bring your diabetes medication?	YES..........1
		NO..........2
T5	Did you take your diabetes medicine yesterday?	YES..........1
		NO..........2
T6	When you feel like your diabetes is under control, do you sometimes stop taking your medicine?	YES..........1
		NO..........2
T7	When you feel like your diabetes is under control, do you sometimes stop taking your medicine?	YES..........1
		NO..........2
T8	Taking medication everyday is a real inconvenience for some people. Do you ever feel hassled about sticking to your diabetes treatment plan?	YES..........1
		NO..........2

SECTION 5: HOSPITALIZATION		
Question		Response
U1	Have you been admitted to the hospital since your last visit?	YES.....1 (U2)<br>NO.....2 (V1)
U2	If yes, what were you hospitalized for? (CHOOSE ONE)	HYPERTENSION.....1<br>DIABETES.....2<br>OTHER SPECIFY.....3<br>________________

SECTION 6:MEDICINE ACCESS		
Question		(PLEASE CIRCLE THE CORRECT RESPONSE)
V1	Please indicate where you get your medicine for your diabetes or high blood pressure (SELECT ALL THAT APPLY)	HOSPITAL PHARMACY.....1<br>COMMUNITY PHARMACY.....2<br>LICENSED CHEMICAL SELLER.....3<br>OTHER SPECIFY.....4<br>REFUSED.....88<br>DON'T KNOW.....99
V2	Have you had problems getting medicines for your high blood pressure or diabetes in the past 6 months?	YES (HIGH BLOOD PRESSURE).....1<br>YES (DIABETES).....2<br>YES (BOTH HIGH BLOOD PRESSURE AND DIABETES)....3<br>NO TO ALL THE ABOVE.....4 (V5)<br>REFUSED.....88<br>DON'T KNOW.....99
V3	If you had difficulty obtaining your medicine in the past 6 months, please indicate where.	HOSPITAL PHARMACY.....1<br>COMMUNITY PHARMACY.....2<br>LICENSED CHEMICAL SELLER.....3<br>OTHER SPECIFY.....4<br>REFUSED.....88<br>DON'T KNOW.....99

V4	Please specify why it was difficult to obtain your medicine?	NOT AVAILABLE AT POINT OF CARE........1
		TOO EXPENSIVE........2
		NHIS NOT ACCEPTED AT PHARMACY........3
		OTHER SPECIFY........4 ______________
		NOT APPLICABLE........5
		REFUSED........88
		DON'T KNOW........99
V5	In the past 6 months, have you ever tried to treat your high blood pressure or diabetes with an alternative method? If yes, choose all that apply (SELECT ALL THAT APPLY)	HERBAL MEDICINE........1
		HOMEOPATHY 2
		ACUPUNCTURE........3
		TRADITIONAL HEALER........4
		PRAYER CAMP........5
		OTHER SPECIFY........6 ______________
		NONE OF THE ABOVE........7
V6	In the past 6 months, have you been referred to a Licensed Chemical Seller or Community Pharmacy because your medicine was not available at the Hospital Pharmacy?	Yes........1
		No........ 2 (W1)
V7	If yes, please indicate place of referral (where you obtained your medicine)	Community Pharmacy........1
		Licensed Chemical Seller........2
		Other Specify........3 ______________

**SECTION 7: LIFESTYLE ADVICE**		
During the past six months, has a doctor or other health worker advised you to do any of the following?		
W1	Quit using tobacco or don't start	YES........1
		NO........2
W2	Reduce salt in your diet	YES........1
		NO........2
W3	Eat at least five servings of fruit and/or vegetables each day	YES........1
		NO........2
W4	Reduce fat in your diet	YES........1
		NO........2
W5	Start or do more physical activity	YES........1
		NO........2
W6	Maintain a healthy body weight or lose weight	YES........1
		NO........2

SECTION 8. CANCER SCREENING		
During the past six months, has a doctor or other health worker advised you to do any of the following?		
X1	Breast Cancer screening	YES......1<br>NO......2
X2	Colon Cancer screening (fecal blood test)	YES......1<br>NO......2

PID (Participant ID No.) [ ]-[ ]/[ ]-[ ]-[ ]-[ ]/[ ]-[ ]/[ ]

Ghana Access and Affordability Program Pilot Study

Hypertension Knowledge Attitude and Practices (KAP) Questionnaire

24 MONTHS

Location and Date	(PLEASE CIRCLE THE CORRECT RESPONSE)
Name of person completing questionnaire	
Institution/ Facility ID	TAMALE TEACHING HOSPITAL..........1
	KOMFO-ANOKYE TEACHING HOSPITAL..........2
	KORLE-BU TEACHING HOSPITAL..........3
	KING'S MEDICAL CENTRE..........4
	AGOGO PRESBYTERIAN HOSPITAL..........5
	ATUA GOVERNMENT HOSPITAL..........6
Date of completion of the instrument	[___]/[___]/[___]<br>dd mm year

WE ARE INTERESTED IN SOME THINGS ABOUT YOU AND YOUR HEALTH. PLEASE RESPOND TO ALL THE QUESTIONS AS BEST AS YOU CAN. THERE ARE NO RIGHT OR WRONG ANSWERS. THE INFORMATION YOU PROVIDE WILL REMAIN CONFIDENTIAL.		
Question		Please circle the correct response
A1	What does the term hypertension mean? :(SELECT ALL THAT APPLY)	HIGH BLOOD PRESSURE..........1
		HIGH BLOOD SUGAR..........2
		I DON'T KNOW..........3
		OTHER (SPECIFY)
A2	There are two numbers used in measuring blood pressure levels. What should the upper number be?(CHOOSE ONE)	LESS THAN 140..........1
		GREATER THAN 140..........2
		EQUAL TO 140..........3
		I DON'T KNOW..........99
		OTHER (SPECIFY)
A3	What should the lower number be? (CHOOSE ONE)	LESS THAN 90..........1
		GREATER THAN 90..........2
		EQUAL TO 90..........3
		I DON'T KNOW..........99
		OTHER (SPECIFY)

A4	How serious of a health concern has high blood pressure been to you?(CHOOSE ONE)	VERY SERIOUS CONCERN..........1<br>SOMEWHAT SERIOUS CONCERN..........2<br>NOT AT ALL SERIOUS CONCERN..........3
A5	How important do you think taking medicine is to keep blood pressure under control? (CHOOSE ONE)	VERY IMPORTANT..........1<br>SOMEWHAT IMPORTANT..........2<br>NOT AT ALL IMPORTANT..........3
A6	What is the most important factor(s) in controlling your high blood pressure? (SELECT ALL THAT APPLY)	TAKING MEDICATIONS..........1<br>EXERCISING..........2<br>LESS STRESS..........3<br>QUITTING SMOKING IF YOU ARE SMOKING..........4<br>REDUCING SALT INTAKE IN YOUR DIET......5<br>REDUCING ALCOHOL INTAKE..........6<br>LOSING WEIGHT..........7<br>ALL OF THE ABOVE..........8<br>I DON'T KNOW..........99<br>OTHER (SPECIFY)
A7	High blood pressure can increase or worsen (SELECT ALL THAT APPLY)	THE RISK OF A HEART ATTACK..........1<br>THE RISK OF STROKE..........2<br>THE RISK OF KIDNEY PROBLEMS..........3<br>ALL THE ABOVE..........4<br>I DON'T KNOW..........99<br>OTHER (SPECIFY)

Ghana Access and Affordability Program Pilot Study

Diabetes Knowledge Attitude and Practices (KAP) Questionnaire

24 MONTHS

Location and Date	(PLEASE CIRCLE THE CORRECT RESPONSE)
Name of person completing questionnaire	
Institution/ Facility ID	TAMALE TEACHING HOSPITAL..1
	KOMFO-ANOKYE TEACHING HOSPITAL..................2
	KORLE-BU TEACHING HOSPITAL...................................3
	KING'S MEDICAL CENTRE...4
	AGOGO PRESBYTERIAN HOSPITAL............................5
	ATUA GOVERNMENT HOSPITAL...................................6
Date of completion of the instrument	[___]/[___]/[___]<br>dd mm year

WE ARE INTERESTED IN SOME THINGS ABOUT YOU AND YOUR HEALTH. PLEASE RESPOND TO ALL THE QUESTIONS AS BEST AS YOU CAN. THERE ARE NO RIGHT OR WRONG ANSWERS. THE INFORMATION YOU PROVIDE WILL REMAIN CONFIDENTIAL.		
Question		(PLEASE CIRCLE THE CORRECT RESPONSE)
A1	Diabetes is a condition in which the body contains:(SELECT ALL THAT APPLY)	A HIGHER LEVEL OF SUGAR IN THE BODY THAN NORMAL...1
		A LOWER LEVEL OF SUGAR IN THE BODY THAN NORMAL...2
		EITHER A HIGHER OR A LOWER LEVEL OF SUGAR IN THE BODY THAN NORMAL...3
		I DON'T KNOW...4
		OTHER (SPECIFY)

A2	The symptom (s) of diabetes is/are: (SELECT ALL THAT APPLY)	INCREASED FREQUENCY OF URINATION.....1
		INCREASED THIRST AND HUNGER.....2
		INCREASED TIREDNESS.....3
		SLOW HEALING OF WOUNDS.....4
		ALL THE ABOVE.....5
		OTHER (SPECIFY)
A4	In a diabetic patient, high blood pressure can increase or worsen (SELECT ALL THAT APPLY)	THE RISK OF A HEART ATTACK.....1
		THE RISK OF STROKE.....2
		THE RISK OF EYE PROBLEMS.....3
		THE RISK OF KIDNEY PROBLEMS.....4
		ALL THE ABOVE.....5
		I DON'T KNOW.....6
		OTHER (SPECIFY)
A5	The lifestyle modification (s) required for diabetic patients are/is? (SELECT ALL THAT APPLY)	WEIGHT REDUCTION.....1
		STOPPING SMOKING.....2
		STOPPING ALCOHOL INTAKE.....3
		ALL THE ABOVE.....4
		I DON'T KNOW.....5
		OTHER (SPECIFY)
A6	The important factors that help in controlling blood sugar are (SELECT ALL THAT APPLY)	A CONTROLLED AND PLANNED DIET.....1
		REGULAR EXERCISE.....2
		MEDICATION.....3
		ALL THE ABOVE.....4
		NONE.....5
		OTHER (SPECIFY)

A7	A well-balanced diet includes: (SELECT ALL THAT APPLY)	VEGETABLES........1
		FRUITS........2
		CARBOHYDRATES........3
		ALL THE ABOVE........4
		NONE........5
		OTHER (SPECIFY)
A8	For proper foot care, a diabetic patient (SELECT ALL THAT APPLY)	SHOULD INSPECT AND WASH THE FEET DAILY........1
		SHOULD SELECT THE BEST POSSIBLE FOOTWEAR........2
		SHOULD WALK BAREFOOT INSIDE AND OUTSIDE THE HOUSE........3
		SHOULD NOT WALK BAREFOOT INSIDE AND OUTSIDE THE HOUSE........4
		OTHER (SPECIFY)
A9	Upon control of diabetes, the medicines: (SELECT ALL THAT APPLY)	CAN BE STOPPED IMMEDIATELY........1
		CAN BE STOPPED AFTER ONE MONTH........2
		SHOULD BE CONTINUED FOR LIFE........3
		I DON'T KNOW........99
		OTHER (SPECIFY)
A10	How do you manage low blood sugar symptoms? (SELECT ALL THAT APPLY)	BY TAKING SUGAR........1
		BY TAKING MEDICINES........2
		BY TAKING INSULIN........3
		I DON'T KNOW........99
		OTHER (SPECIFY)

Ghana Access and Affordability Program Pilot Study

Study Outcomes

**(1) Did patient complete follow-up for entire duration of study?**	Yes...................................1
	No....................................2
**(2) If no to question 1, when was patient last visit? Date**	
**(3) Indicate why patient did not complete follow-up**	Lost-to-follow up....................1
	Died...2
	Moved out of study area.......................3
	Transferred to another clinic......... 4

Ghana Access and Affordability Program Pilot Study

© 2014
