# Supplementary material for: A multi-center prospective cohort study to evaluate the effect of differential pricing and health systems strengthening on access to medicines and management of hypertension and diabetes in Ghana: A study protocol
Source: Gates Open Res. 2018 May 3;2:6. Originally published 2018 Feb 7. [Version 2] doi: 10.12688/gatesopenres.12797.2 (PMC5941285; doi:10.12688/gatesopenres.12797.2)
Supplement: Supplementary file 3 [file gatesopenres-2-13889-s0002.tgz › 4dd10987-e8ed-44b0-80bd-b229fe524c9f.zip!/Supplementary File 3 - Data collection tools/Supply Chain Questionnaire ver 4.doc]

GHANA ACCESS AND AFFORDABILITY

RESEARCH STUDY

SUPPLY CHAIN QUESTIONNAIRE

| Location and Date | | Response | | Code | |
| --- | --- | --- | --- | --- | --- |
| Name of person completing questionnaire |  | |  | |  |
| Institution/ Facility ID  1=Tamale Teaching Hospital 2=Komfo-Anokye Teaching Hospital  3= Korle BuTeaching Hospital 4= King’s Medical Centre  5= Agogo Presbyterian Hospital 6= Atua Government Hospital | └──┘ | |  | |  |
| Date of completion of the instrument | dd mm year | |  | |  |

| **Supply Chain** | | | | |
| --- | --- | --- | --- | --- |
| **Question** | **Response** | | | **Code** |
| Does the hospital have a drug bulletin to inform prescribers of medicines available for hypertension and diabetes management? | Yes | | 1 |  |
|  | No | | 2 | A1 |
| Has the drug bulletin been updated in the last 6 months? | Yes | | 1 | A2 |
|  | No | | 2 |  |
| Have there been stock outs for any of the hypertension drugs in the last 30 days? | Tab Atenolol 25mg, 50mg **(STATE STRENGTH(S) AVAILABLE)** | | 1 | A3 |
|  | Tab Atenolol/ Hydrochlorothiazide | | 2 |  |
|  | Tab Bendroflumethiazide 2.5mg | | 3 |  |
|  | Tab Hydrochlorthiazide  25 mg | | 4 |  |
|  | Tab Hydralazine 25mg | | 5 |  |
|  | Inj Hydrallazine | | 6 |  |
|  | Tab Labetalol | | 7 |  |
|  | Inj. Labetalol | | 8 |  |
|  | Tab Lisinopril, 5mg, 10mg (**STATE STRENGTH(S) AVAILABLE)** | | 9 |  |
|  | Lisinopril/Hydrochlorothiazide | | 10 |  |
|  | Tab Losartan 50mg | | 11 |  |
|  | Tab Methyldopa 250 mg | | 12 |  |
|  | Nifedipine (sustained release) 10mg, 20mg, 30mg **(STATE STRENGTH(S) AVAILABLE)** | | 13 |  |
|  | Amlodipine 5mg, 10 mg**(STATE STRENGTH(S) AVAILABLE)** | | 14 |  |
|  | Prazosin 0.5mg, 1mg**(STATE STRENGTH(S) AVAILABLE)** | | 15 |  |
|  | Others **(SPECIFY TYPE & STRENGTH(S)……)** | | 16 |  |
|  | DP meds**( SPECIFY FOR EACH PRODUCT & DOSAGE FORM)** | | 14 |  |
| Have there been stock outs for any of the following diabetes drugs in the last 30 days? | Tab Glibenclamide **5mg** | | 1 | A4 |
|  | Tab Glimepiride 1mg, 2mg, 4mg **(STATE STRENGTH(S) AVAILABLE)** | | 2 |  |
|  | Tab Gliclazide 30mg | | 3 |  |
|  | Inj Insulin **(STATE TYPE & DOSAGE FORM)** | | 4 |  |
|  | Tab Pioglitazone 15mg, 30mg | | 5 |  |
|  | Metformin | | 6 |  |
|  | Others **(SPECIFY TYPE & STRENGTH(S) …….)** | | 7 |  |
|  | DP meds**( SPECIFY FOR EACH PRODUCT & DOSAGE FORM)**… | | 8 |  |
| Have there been any forecasting reports done in the last 90 days for hypertension and diabetes medicines? If yes, please show report.  **PLEASE TICK IF REPORT IS SHOWN [ ]** | Yes | | 1 | A5 |
|  | No | | 2 |  |
| Have there been any requisition orders done in the last 90 days for hypertension and diabetes medicines? If yes, please show report. **{TICK IF REPORT IS SHOWN [ ]}** | | Yes | 1 | A6 |
|  |  | No | 2 |  |
| Is there a designated pharmacy outlet for diabetes medicines that is easily accessible to patients? | Yes | | 1 | A7 |
|  | No | | 2 |  |
| Are all the prescribed medicines for Diabetes obtained from the designated pharmacy outlet? | Yes | | 1 |  |
|  | No | | 2 |  |
| Is there a designated pharmacy outlet for hypertension medicines that is easily accessible to patients? | Yes | | 1 | A8 |
|  | No | | 2 |  |
| Are all the prescribed medicines for hypertension obtained from the designated pharmacy outlet? | Yes | | 1 | A9 |
|  | No | | 2 |  |
| Is there a functioning system in use for tracking medicines supplied to **patients** from **Pharmacy outlets**? | Yes | | 1 | A10 |
|  | No | | 2 |  |
| Is there a functioning system in use for tracking medicines supplied from **stores** to **pharmacy outlets**? | Yes | | 1 | A11 |
|  | No | | 2 |  |
| Are defective products (expired/damaged medicines etc) detected and moved away from the reach of patients? | Yes | | 1 | A12 |
|  | No | | 2 |  |
| Does the pharmacy outlet have the following functional/optimal conditions?  **TICK IF TEMPERATURE MONITORS ARE SEEN WHERE APPLICABLE** | Refrigerator    [ ] | | 1 | A13 |
|  | Air conditioner  [ ] | | 2 |  |
|  | Inventory software | | 3 |  |
|  | Organized, well ventilated storage space, | | 4 |  |
| Does the main store have the following functional/optimal conditions?  Tick if temperature monitors are seen where applicable  **[IN ADDITION TO THE QUESTIONNAIRE, INVESTIGATOR SHOULD ALSO OBSERVE/INSPECT TO CONFIRM]** | Refrigerator    [ ] | | 1 | A14 |
|  | Air conditioner  [ ] | | 2 |  |
|  | Inventory software | | 3 |  |
|  | Organized, well  ventilated storage space | | 4 |  |
| What is the human resource capacity at your pharmacy? Do you have any of the following? How many? **[INVESTIGATOR TO VERIFY]** | Trained pharmacist  Number___ | | 1 |  |
|  | Pharmacy technician Number___ | | 2 |  |
|  | Pharmacy Assistant/MCA | | 3 |  |
|  | Store personnel  Number___ | | 4 | A15 |
|  | Procurement personnel  Number___ | | 5 |  |
| What is the means of communication between pharmacy staff and clinicians on medicine availability and its efficient utilization in the system for patients benefit? | Phone calls | | 1 |  |
|  | Weekly review meetings of clinical team | | 2 | A15 |
|  | Via Intranet  None | | 3  4 |  |
|  | Other (specify) | | 5 |  |
