# Supplementary material for: A multi-center prospective cohort study to evaluate the effect of differential pricing and health systems strengthening on access to medicines and management of hypertension and diabetes in Ghana: A study protocol
Source: Gates Open Res. 2018 May 3;2:6. Originally published 2018 Feb 7. [Version 2] doi: 10.12688/gatesopenres.12797.2 (PMC5941285; doi:10.12688/gatesopenres.12797.2)
Supplement: Supplementary file 4 [file gatesopenres-2-13889-s0003.tgz › 9086a958-54fe-44af-b7f0-c2c4cd165634.zip!/FGD -patients who could not purchase medicine.docx]

**FGD-PATIENTS WHO COULD NOT PURCHASE STUDY MEDICINE (MEN & WOMEN)**

***INTRODUCTION****:*

Formal GREETING!!! We are health research workers and we are pleased to have you here to discuss health related issues. We are particularly interested in knowing how you think about the management and treatment of Hypertension, Diabetes & Cancer diseases.

In this discussion, there are no wrong or correct responses or answers. Therefore, we will want to encourage everybody to contribute to the discussion in order to make it more interesting and lovely. We hope that the information you will provide will help us achieve the aims and objectives of this work. This will enhance the management and treatment of these diseases in Ghana and it will also improve on the health status of people with health problems.

Confidentiality: All the information that will be collected in this study will be treated in strict confidence and will be used for the intended purposes only. The information will only be accessible to the study investigators.

We will like to ask for your consent to use the digital tape recorder to record the discussion. This will enable us capture all the issues that we will discuss here and then transcript it out when we get back to the office. The recordings will be deleted after they have been transcribed.

***INSTRUCTIONS***

- *After introduction, verbal consent and switching on the recorder, try to make the interview as natural as possible to an informal conversation. It is very important that the participants feel comfortable with you.*
- *You can change the order of the questions as they appear in the guide the way that you and the interviewee will be comfortable.*
- *Try to ask all questions and also probe for clarification of unclear statements and issues made during the discussion.*

**Background information (*take the background information for each participant)***

Age, Education, Ethnicity, Religion, Occupation

**Health seeking behavior**

1. What do people who are diagnosed with Hypertension, Diabetes & Cancer diseases normally do in terms of treatment? (**Probe for: modern treatment and other/local treatment**)
a. Also probe for the most patronized treatment source and why?
2. If mentioned, what are the types of other treatment sources/methods available for patients with Hypertension, Diabetes & Cancer in this area? **Probe for**
   a. Local treatment at home (herbs)
   b. Traditional healer
   c. Prayer camps

**Health Care financing**

3. How do people with these conditions (Hypertension, Diabetes & Cancer) finance their health care needs? (Probe for health insurance and out-of-pocket payment).
4. How do you pay for your medications when you don’t have money? **Probe on**:
   a. Whether they borrow, sell assets, get support from relatives etc.
   b. How does such kind of coping mechanisms affect their life

**Knowledge, access & adherence to treatment**

5. What type of health information have you received about hypertension, diabetes & cancer diseases? **Probe for**:
   a. Management of these diseases at home
   b. Health care/treatment
   c. Possible side effects and what to do
   d. Where they receive information from
6. What medicine have you been taken? Probe on:
   a. Where they got the medicine from
   b. Health information on the use of the medicine
   c. How they use the medicine (number of tabs to be taken & how many times a day)
7. How difficult or easy is it for you to use the medicine? **Probe on** whether they are able to use all medications given them.
8. For those who could not use all the medicine, get them to explain the reasons why they could not use all the medicine? **Probe on:**
   a. Recall on timing of frequency of dosing problem, taste of drug, number of tablets taken, side effects etc.
   b. Get participants to suggest ways to help address these problems.
9. What do you have to say concerning the availability and access to medicines for the treatment of hypertension, diabetes and cancer diseases? Probe on:
   a. Availability of medicines at the health facilities
   b. Whether it is easy for patients to get these medications? **Why & why not**?
   c. Whether it is easy to afford these medications? **Why & why not**?
   d. Other factors affecting availability, affordability & access to these medicines
   e. Get them to suggest ways to improve on availability & access to these medicines
10. Comparing 4 years ago to the current situation, have there been any changes concerning the availability and access to hypertension, diabetes and cancer medications? **Why & why not?**
11. Were you prescribed any medicine for your condition? **Why & why not?**
12. Were you able to get the medicine prescribed to you? **Why & why not?**
13. What other factors accounted for your inability to purchase the medicine prescribed to you? If not mentioned, probe for solutions to help solve these problems

**Perceptions on the burden of diseases & health outcomes**

14. How do you perceive your current health status? (**Probe on whether health status is: poor, fair, good, excellent**).
15. What do you think has contributed to your current (good or poor) health status?
16. What is your opinion on the current management/treatment of Hypertension, Diabetes & Cancer diseases in this health facility?
17. Are there any changes you have seen within the last 4 years concerning the management and treatment of Hypertension, Diabetes & Cancer diseases in this health facility? **Why & why not**? What brought about the changes?
    a. What do you have to say about the health workers attitude towards you?
18. Compare the current situation to 4 years ago, has there been any change in the burden of Hypertension, Diabetes & Cancer diseases? **Why & why not**?

**Impact on health system strengthening**

19. What is your opinion on the health system capacity to manage Hypertension, Diabetes & Cancer diseases especially in this health facility? **Probe on**:
    a. Availability of medical staff
    b. Attitude of medical staff towards patients with these conditions
    c. Availability of medicine for these patients
    d. Availability of equipment
20. Generally, what do you think could be done to enhance the management and treatment of these diseases?
21. What do you know about the activities of Ghana Access & Affordable Project? **Probe on:**
    a. Availability of medications
    b. Support for patients to get medications
    c. Health education
22. What is your perception about the GAAP pilot intervention? **Probe for views on price differential concept?**
23. Do you think the activities of GAAP project should be implemented in other districts, regions and countries? **Why & why not?**
24. What do you think could be done to help sustain the program? **Probe for the roles of:**
a. Health system
b. Other NGOs
c. Patients

**What else do you have to say concerning the activities of the GAAP project?**

**Thank you for your time!!!**
