# Supplementary material for: A multi-center prospective cohort study to evaluate the effect of differential pricing and health systems strengthening on access to medicines and management of hypertension and diabetes in Ghana: A study protocol
Source: Gates Open Res. 2018 May 3;2:6. Originally published 2018 Feb 7. [Version 2] doi: 10.12688/gatesopenres.12797.2 (PMC5941285; doi:10.12688/gatesopenres.12797.2)
Supplement: Supplementary file 4 [file gatesopenres-2-13889-s0003.tgz › 9086a958-54fe-44af-b7f0-c2c4cd165634.zip!/IDI for health workers.docx]

**IDI GUIDE FOR HEALTH WORKERS (Doctors & Nurses)**

***INTRODUCTION****:*

Formal GREETING!!! My name is…..i am a health research worker and I am pleased to have you here to discuss health issues related to Hypertension, Diabetes and Cancer diseases. We are particularly interested in knowing GAAP pilot interventions and its impact on the management and treatment of Hypertension, Diabetes and Cancer diseases particularly in this health facility.

We hope that the information you are going to provide will help us to achieve the purpose of this work and also go a long way to improve on the care and management of Hypertension, Diabetes and Cancer diseases in Ghana.

Confidentiality: All the information that will be collected in this study will be treated in strict confidence and will be used for the intended purposes only. The information will only be accessible to the study investigators.

We will like to ask for your consent to use the digital tape recorder to record the discussion. This will enable us capture all the issues that we will discuss here and then transcript it out when we get back to the office. The recordings will be deleted after they have been transcribed**.**

***INSTRUCTIONS***

- *After introduction, verbal consent and switching on the recorder, try to make the interview as natural as possible to an informal conversation. It is very important that the participants feel comfortable with you.*
- *You can change the order of the questions as they appear in the guide the way that you and the interviewee will be comfortable.*
- *Try to ask all questions and also probe for clarification of unclear statements and issues made during the discussion.*

**Background information**

Age, Education, Ethnicity, Religion, and position

**Health information & access to medications**

1. What type of health information do you give to people with hypertension, diabetes and cancer diseases? **Probe for**:
   a. Management of these diseases at home
   b. Health care/treatment
   c. The use of the medications (number of tabs to be taken & how many times a day)
   d. Possible side effects and what to do
2. What is your opinion concerning the availability and access to medicines for the treatment of hypertension, diabetes and cancer diseases in this health facility? **Probe for views on:**
   a. Access to these medications by patients
   b. Patient affordability
   c. Are there other factors affecting patients affordability & access to these medicines? **Explain**
   d. Suggestions to help improve availability and access to these medications
3. Comparing 5 years ago to the current situation, have there been any changes concerning the availability and access to these medicines for the management of hypertension, diabetes and cancer diseases? What brought about the change? Probe for the role of the GAAP pilot intervention.

**Supply chain/ drug stock**

4. Where does this facility usually get its supply of medicines (for treatment of hypertension, Diabetes & Cancer diseases) from?
5. What is the supply chain of the medications to the health facility?
6. When you run short of these medicines and patients come to the facility, what do you do?
7. What are the challenges with regards to the supply of these medicines (delay payment affecting supply)? Probe for solutions to these problems.

**Training**

8. Can you tell me the kind of training you received on the management of Hypertension, Diabetes & Cancer diseases?
9. Who organized the training? Probe on training received from GAAP pilot study.
10. What is your perception about the training you received from GAAP pilot study on the management & treatment of these diseases? **Probe on:**

a. The number of times training was organized for health workers at the facility.

b. Period used for the training

c. Improve knowledge and skills on management & treatment guidelines

11. What is your perception on the training you received on data management?
12. What problems were encountered during the training? If not mentioned, what recommendations would you have given to help improve on the training programme?

**Impact of GAAP pilot intervention on the burden of diseases & health outcomes**

13. What do you know about the activities of Ghana Access & Affordable Project (GAAP)? **Probe for**
    a. Their role in the management of these diseases
    b. Their support for patients to get medications
14. What role do you play as a health worker on the activities of GAAP project?
15. What is your opinion about the GAAP pilot interventions? **Probe for** views on the price differential concept?
16. Are there any changes concerning the management and treatment of Hypertension, Diabetes & Cancer diseases in this health facility? What brought about the changes?
17. Compare the current situation to 4 years ago, is there a change in the burden of Hypertension, Diabetes & Cancer diseases in catchment area of this facility? **What brought about the changes**
18. What do you have to say concerning the health status of this group of patients? Has there been any improvement in their health status compare to 4 years ago? What accounted for this? Probe for the role of GAAP activities.

**Impact of GAAP activities on health system strengthening**

19. Do you think the GAAP project has contributed in building the capacity of the health system to manage Hypertension, Diabetes & Cancer diseases? **Why & why not**? Probe also for:
    a. Availability of medical staff
    b. Availability of medications
    c. Availability of equipment
    d. Procedure used to get patients honor clinical appointments
    e. Method of evaluating treatment
20. Do you think the activities of GAAP project should be implemented in other districts, regions and countries? **Why & why not?**
21. What do you think could be done to help sustain the program? **Probe for the role of:**
a. Health system
b. Other NGOs
c. Patients
22. Generally, what are the problems associated with the implementation of the GAAP pilot interventions?
23. What do you think should have been done differently to improve on the program?

**What else do you have to say concerning the activities of the GAAP project?**

**Thank you for your time!!!**
