# Supplementary material for: A multi-center prospective cohort study to evaluate the effect of differential pricing and health systems strengthening on access to medicines and management of hypertension and diabetes in Ghana: A study protocol
Source: Gates Open Res. 2018 May 3;2:6. Originally published 2018 Feb 7. [Version 2] doi: 10.12688/gatesopenres.12797.2 (PMC5941285; doi:10.12688/gatesopenres.12797.2)
Supplement: Peer review history

# Peer review: Referee response for version 2

**DOI:** 10.21956/gatesopenres.13889.r26417

**Published:** 2018-05-21

**Authors:** Fatima Suleman

**Affiliations:** 1 Discipline of Pharmaceutical Sciences, School of Health Sciences, University of KwaZulu-Natal (UKZN), Durban, South Africa

I have read through the revised version and the authors comments. I am satisfied with the revisions and approve this submission.

I have read this submission. I believe that I have an appropriate level of expertise to confirm that it is of an acceptable scientific standard.

# Peer review: Referee response for version 2

**DOI:** 10.21956/gatesopenres.13889.r26416

**Published:** 2018-05-04

**Authors:** David Peiris

**Affiliations:** 1 The George Institute for Global Health, University of New South Wales (UNSW), Camperdown, NSW, Australia

Thanks for the revisions. The authors have done as much as they can to deal with the methodological limitations and I do not have anything further to add. It's good to see the process evaluation strategy has been given more thought. Whatever the outcomes, better understanding of the implementation process will yield valuable insights into understanding what role this type of strategy may have in strengthening health systems.

I have read this submission. I believe that I have an appropriate level of expertise to confirm that it is of an acceptable scientific standard.

# Peer review: Referee response for version 1

**DOI:** 10.21956/gatesopenres.13858.r26337

**Published:** 2018-04-27

**Authors:** Fatima Suleman

**Affiliations:** 1 Discipline of Pharmaceutical Sciences, School of Health Sciences, University of KwaZulu-Natal (UKZN), Durban, South Africa

2. Is the study design appropriate for the research question?

 Partly: The two groups being compared may not be matched or similar, so there is a danger of direct comparison. Also, access has not been defined clearly, and there is nothing that indicates measurement of prescriptions being filled or not and how that relates to the differential pricing model being offered. How will the fact that there are different models be considered in the analysis? Also, in the write up, the abstract indicates recently diagnosed participants and the methods just refer to previously diagnosed, but there is no detail on how far back the diagnosis must go.

 3. Are sufficient details of the methods provided to allow replication by others?

 No: What are the models of differential pricing? How are these factored into the analyses? How are the interventions described different from the existing system of care? Access has not been described for supply chain issues only. If access is to be defined at patient level, then actual filling of prescriptions need to be considered. At the moment this is being assumed. How are researchers dealing with recall bias?

 4. Are the datasets clearly presented in a useable and accessible format?

 Partly: What are the medicines being used in the study? How are the doctors being educated on these? Is it by brand name or by INN?

 In addition to the comments above I include the following:

- There is also nothing in the protocol about continuation of medicines for patients post the trial. What will happen once the trial is over? Will patients be switched back?
- Will the DP scheme continue indefinitely for enrolled patients?
- Is there an economic evaluation plan that is not being described in this article?
- What is the price differential of the medicines in the market and the DP prices offered?

I have read this submission. I believe that I have an appropriate level of expertise to confirm that it is of an acceptable scientific standard, however I have significant reservations, as outlined above.

# Response

**Published:** 2018-05-01

**Authors:** Linda Meta Mobula

2. Is the study design appropriate for the research question?

Partly: The two groups being compared may not be matched or similar, so there is a danger of direct comparison. Also, access has not been defined clearly, and there is nothing that indicates measurement of prescriptions being filled or not and how that relates to the differential pricing model being offered. How will the fact that there are different models be considered in the analysis? Also, in the write up, the abstract indicates recently diagnosed participants and the methods just refer to previously diagnosed, but there is no detail on how far back the diagnosis must go.

**We define access according to the World Health Organization definition as, “having medicines continuously available and affordable at public or private health facilities or medicine outlets that are within one hour's walk of the population”. We have included this definition in the manuscript.**

**Prescriptions refills and uptake of medicines (for both DP and MP) were routinely monitored.  Please see supplementary file 3.**

**The study will include patients with a new diagnosis or previously known diagnosis of hypertension and diabetes. We collected data on the number of years of diagnosis of diabetes as a variable and do not exclude patients based on the number of years of diagnosis. This has been amended in the abstract and in the methods section.**

**We recognize that the two groups are not similar and will be collecting data to determine confounding variables that will affect our primary and secondary outcomes. Our analysis will control for these confounding variables.**

3. Are sufficient details of the methods provided to allow replication by others?

No: What are the models of differential pricing? How are these factored into the analyses? How are the interventions described different from the existing system of care? Access has not been described for supply chain issues only. If access is to be defined at patient level, then actual filling of prescriptions need to be considered. At the moment this is being assumed. How are researchers dealing with recall bias?

**In-country differential pricing was the model of differential pricing being used for this study. Though DP and MP patients will be analyzed separately, all patients including those who have access to the Ghana National Health Insurance Scheme (NHIS) will be evaluated as part of a cohort study using longitudinal analysis methods. We will determine confounding variables and account for those in the analysis. We have specified that in-country differential pricing was used in the manuscript.**

**We will address recall bias by collecting data from medical records.** 

**We have also included the following in the manuscript to explain how reimbursement for the National Health Insurance Scheme (NHIS) occurs.**

**Reimbursement and Payment Mechanism**

**Under NHIS, payments to providers (clinics, hospitals, contracted private pharmacies) are made based on claims submitted by the provider to which the insured patient belongs. These rules are generally based on the Standard Treatment Guidelines (STG) published by GNDP, although NHIA issued its own abbreviated version of the guideline that lists treatment options for certain conditions and may differ from STG in some details. The NHIA Medicines List defines which drugs (listed by INN) can be prescribed and how much is reimbursed for each drug. It is generally based on the EML but again differs in some details and includes more drugs than the EML.**

**Consistent with anti-trust laws that govern industry interactions, each Participant Company independently and voluntarily developed its own marketing and pricing strategies and was solely responsible for its own compliance with applicable anti-trust laws.  Each of the participating companies independently and unilaterally made decisions involving pricing of medicines as part of the Access and Affordability Initiative.**

4. Are the datasets clearly presented in a useable and accessible format?

Partly: What are the medicines being used in the study? How are the doctors being educated on these? Is it by brand name or by INN?

**The medicines being used in the study are included as a supplementary file.  Clinical providers participated in a training seminar (for physicians, nurses, pharmacists). Clinical guidelines were developed for use in the training seminar (supplementary file 2). The Ghana EML lists medicines by INN. Both brand name and INN were used in the training.** 

In addition to the comments above I include the following:

- There is also nothing in the protocol about continuation of medicines for patients post the trial. What will happen once the trial is over? Will patients be switched back?

**Patients on study medicines will continue to have access to the medicines at the differential price after the study ends. The participating companies undertake to ensure study medicines are available at the project price to all participants that are enrolled in this pilot study and are on the study medicines, for as long as the medicines are available in Ghana. **

- Will the DP scheme continue indefinitely for enrolled patients?

**Medicines will continue to be available to patients at the DP rate (see above).**

- Is there an economic evaluation plan that is not being described in this article?

**An economic analysis will be conducted which will include a cost analysis of the interventions, analysis of  the following:  efficient use of differentially priced medicines, value analysis of DP, supply chain structure and incentives and affordability index. This is included in the updated manuscript.**

- What is the price differential of the medicines in the market and the DP prices offered?

**Due to anti-trust laws, we are unable to share the price of medicines for MP and DP.**

# Peer review: Referee response for version 1

**DOI:** 10.21956/gatesopenres.13858.r26334

**Published:** 2018-04-05

**Authors:** David Peiris

**Affiliations:** 1 The George Institute for Global Health, University of New South Wales (UNSW), Camperdown, NSW, Australia

This is an interesting study of a strategy to address both supply and demand side barriers to accessing recommended medications for diabetes and hypertension care in various hospital settings in Ghana. It has strong stakeholder engagement and in particular the PPPs that have been established between the pharma companies and the government will mean that the study is highly policy relevant.

 One critical overarching issue I have is the timing of this protocol paper. I note that the study was completed almost 1 year ago. Have analyses of the trial results already commenced? If so there are risks that this protocol has been developed post-hoc.

 Aside from this, I raise the following issues with the design some of which are clearly too late to be addressed given the study is now finished.

1. There is a major risk of bias in comparing the DP and MP groups given the eligibility criteria are based on poverty index scores. Clearly the MP group will be more affluent and with that comes a whole range of confounders that could potentially explain any differences in outcomes between the two groups. I think at best pre-post analyses of the two groups separately is the best that can be done here and that of course comes with caveats in interpretation particularly for the clinical outcomes where regression to the mean will play an important role. Alternatively a matched cohort from a non-participating hospital could help address this but again probably too late for that now.
2. Despite a major part of the intervention being about differential pricing there are actually no details on what the price implications will be for the "innovator medicines". This is essential information to get any sense of the strength of the price incentives being tested here.
3. The supply chain is quite rightly being targeted but the strategy is to in effect create an entirely separate supply chain with the participating pharma companies. It would have been much more attractive to see these drugs incorporated into the existing supply chain and efforts made to strengthen that.
4. Is it possible to provide a list of what drugs constitute innovator drugs and what is on the essential medicines list and what the differential prices are for these drugs? Related to the above issue of bias there could be disincentives to doctors opting for an innovator drug for people in the market price arm and therefore there is a risk of differential prescribing of drugs to the two groups due to price rather than for clinical reasons. Also what has happened post study with access to innovator drugs? Have patients been transitioned back to essential medicines?
5. The two primary outcomes are a series of several outcomes. The supply chain related outcomes are generally at the service level rather than the individual and I am not sure how they will be analysed for change given there are only 5 sites. It would be better to have a single composite outcome for supply and for disease control. There does not appear to be any accounting for clustering at the hospital or provider level in the sample size estimates.
6. Although costs of the medication will be captured, there does not appear to be a formal economic evaluation which again would seem to be very important given the need to demonstrate value to the various payers involved in this study.
7. Although there is a qualitative evaluation incorporated it would be good to anchor this in an overarching framework such as the MRC Guidance on process evaluations of complex interventions.

I have read this submission. I believe that I have an appropriate level of expertise to confirm that it is of an acceptable scientific standard, however I have significant reservations, as outlined above.

# Response

**Published:** 2018-04-23

**Authors:** Linda Meta Mobula

1. There is a major risk of bias in comparing the DP and MP groups given the eligibility criteria are based on poverty index scores. Clearly the MP group will be more affluent and with that comes a whole range of confounders that could potentially explain any differences in outcomes between the two groups. I think at best pre-post analyses of the two groups separately is the best that can be done here and that of course comes with caveats in interpretation particularly for the clinical outcomes where regression to the mean will play an important role. Alternatively a matched cohort from a non-participating hospital could help address this but again probably too late for that now. 

**The two groups (MP and DP) will be analyzed separately at baseline and at 18 months. It is in fact too late to use a matched cohort at a non-participating hospital.**

2. Despite a major part of the intervention being about differential pricing there are actually no details on what the price implications will be for the "innovator medicines". This is essential information to get any sense of the strength of the price incentives being tested here.

**The price of drugs were determined independently by the pharmaceutical companies and the authors did not have any influence on the price of medicines.**

3. The supply chain is quite rightly being targeted but the strategy is to in effect create an entirely separate supply chain with the participating pharma companies. It would have been much more attractive to see these drugs incorporated into the existing supply chain and efforts made to strengthen that.

**This is true and is a flaw of the study. However, once procurement was done and medicines delivered to the facilities, they were part of existing supply chain at facilities.**

4. Is it possible to provide a list of what drugs constitute innovator drugs and what is on the essential medicines list and what the differential prices are for these drugs? Related to the above issue of bias there could be disincentives to doctors opting for an innovator drug for people in the market price arm and therefore there is a risk of differential prescribing of drugs to the two groups due to price rather than for clinical reasons. Also what has happened post study with access to innovator drugs? Have patients been transitioned back to essential medicines?

**We have provided a list of the innovative medicines, but are unable to provide the prices due to anti-trust laws. The pharmaceutical companies independently determined the price of innovative medicines. Once the study has been completed, the innovative medicines were still made available to study participants. Providers were asked to prescribe based on clinical status rather than price, but this was difficult to control.**

5. The two primary outcomes are a series of several outcomes. The supply chain related outcomes are generally at the service level rather than the individual and I am not sure how they will be analysed for change given there are only 5 sites. It would be better to have a single composite outcome for supply and for disease control. There does not appear to be any accounting for clustering at the hospital or provider level in the sample size estimates.

**We are using standard measures to evaluate hypertension and diabetes control that have been used elsewhere in the literature, rather than a composite outcome for disease control.**

**We were more interested in service level supply chain outcomes at the health facility level and how this would affect access to medicines and  health outcomes, given that we were interested in how strengthening health systems would lead to improved health outcomes.**

 6.Although costs of the medication will be captured, there does not appear to be a formal economic evaluation which again would seem to be very important given the need to demonstrate value to the various payers involved in this study. 

**There is a formal economic analysis which will include a cost analysis of the interventions, as well as analysis of the following:  efficient use of differentially priced medicines, value analysis of DP, supply chain structure and incentives and affordability index.**

7. Although there is a qualitative evaluation incorporated it would be good to anchor this in an overarching framework such as the MRC Guidance on process evaluations of complex interventions.

**The Medical Research Council guidance for complex interventions will be considered in the analysis of the results.**

# Peer review: Referee response for version 1

**DOI:** 10.21956/gatesopenres.13858.r26236

**Published:** 2018-02-21

**Authors:** Sonak D. Pastakia

**Affiliations:** 1 Purdue Kenya Partnership, Purdue University, Indianapolis, IN, USA

Summary:

 This study describes their approach for studying the impact of inclusion of medications for NCDs when using a differential pricing mechanism.

 My comments are meant to identify some of the limitations of the study design which are important and are not intended to suggest that the study is fatally flawed. They are merely concerns and tips for things to include if possible.

1. Is the study design appropriate for the research question?
  a. I worry that the design will have considerable confounding simply because I would anticipate that the patients that participate in the differential pricing would be the ones who are more financially sound and thus might not be exposed to the many health consequences that come along with low socioeconomic status.  I would have preferred either a stepped wedge design, a study where you select two different regions entirely, or a cluster-randomized trial where the intervention regions are compared to non-intervention regions
  b.  Will there be any efforts to validate the results of the poverty index through home visits are any other means? Self reported responses on income have inherent limitations.
2. Are sufficient details of the methods provided to allow replication by others?
  a. With the emergence of Ghana's National Health Insurance Scheme, I am curious to hear how these medications fit within national health insurance scheme. Will somebody with NHIS be able to access these meds as part of their insurance?
  b. I would want to see the list of the innovator medicines included in this program, the potential substitutes on the EML, and the price differences between them.  
  c. If possible, I doubt it is, I would be interested in seeing the uptake of the innovator medicines based on the availability of the EML drugs in the facilities. I would bet that uptake of innovators would be strongly influenced by eml drug availability
  d. Another nice feature to have, but probably not possible, would be an estimate of the overhead costs with including these innnovator meds. This does not have to be described here but if estimates could be provided when you publish the paper, that would be great.
  e. When you analyze patients with diabetes or hypertension, it would be wise to do separate evaluations for new vs previously diagnosed patients. I would imagine there would be dramatic differences in uptake of medications based on this factor alone
  f. Are the innovator medications included only for public sector clients or will the private sector components of these hospitals be stocking the innovator medications as well.
  g. Another point of curiousity, how do prescribers generally write down medications, do they write generic names or the brand names. How will they do it within this study for the innovator medications.
  h. Page 7, recruitment process and description of physician prescribing-physicians can use their discretion to decide mp or dp, why would they ever select MP? If I was the prescriber, I would always use DP and think of a reason why i would want my patients to pay more. I bet prescribers would get issues if patients started to realize that they were being charged differently based on the prescriber's opinion.
  i. Can you include the protocols for diabetes and hypertension care so we can see how the eml and innovator meds fit into the protocol? It would be an interesting analysis to see how many prescribers deviate from this.
  j. For the study sample size and the primary outcome, I would have preferred a continuous variable rather than the dichotomous one selected but you mention that you will report the continuous variable so it is not a big deal. The reason for this is that I'm not exactly sure how you define control for htn and dm.  (i'm assuming bp <140/90 and Hba1c <7 / fasting glucose <126.) If a patient were to experience a dramatic drop in a1c from 13 to 10 over the period of the study, I would be pretty happy with their progress but they would be concerned as not having achieved control.  
  k. For adherence, I would also recommend tracking refill history to complement questionnaire.
  l. Is it expected that patients will purchase two months of medications since their visits are every two months? That sometimes becomes too expensive.

I have read this submission. I believe that I have an appropriate level of expertise to confirm that it is of an acceptable scientific standard.

# Response

**Published:** 2018-04-23

**Authors:** Linda Meta Mobula

1. Is the study design appropriate for the research question?a. I worry that the design will have considerable confounding simply because I would anticipate that the patients that participate in the differential pricing would be the ones who are more financially sound and thus might not be exposed to the many health consequences that come along with low socioeconomic status.  I would have preferred either a stepped wedge design, a study where you select two different regions entirely, or a cluster-randomized trial where the intervention regions are compared to non-intervention regions 

**Patients that are categorized as “DP” and “MP” will both have access to interventions such as counseling and education, and will both be monitored as part of the cohort. We do not know if socio-economic status is in fact a confounder as the results have yet to be analyzed. We have gathered data on household income at baseline, which will be an independent variable in the analysis, and therefore will be used to determine if this is in fact a confounder.**

b.  Will there be any efforts to validate the results of the poverty index through home visits are any other means? Self reported responses on income have inherent limitations.

**There will not be efforts to conduct home visits in order to validate the results of the poverty index as the study has already ended. This has been included as a limitation in the discussion section.**

2. Are sufficient details of the methods provided to allow replication by others?

a. With the emergence of Ghana's National Health Insurance Scheme, I am curious to hear how these medications fit within national health insurance scheme. Will somebody with NHIS be able to access these meds as part of their insurance? 

**Individuals that currently have NHIS are also included in the study and eligible to access these medicines. However, their insurance will not pay for these medicines, as they are not on the Ghana EML.  Ghana Health Services and the Ministry of Health have only allowed the innovator drugs to be used in the study and not included in the EMl.  The only medicine that is currently on the EML is losartan, which patients can access for free if they have NHIS.**

b. I would want to see the list of the innovator medicines included in this program, the potential substitutes on the EML, and the price differences between them.  

**Here is the list of innovative medicines. We are not allowed to share the price of medicines due to anti-trust laws.**

Amlodipine besylate

CARDIOVASCULAR DRUG

REGISTERED WITH FDA

Atorvastatin calcium

Ibersartan

CARDIOVASCULAR DRUG

REGISTERED WITH FDA

Ibersartan and HCTZ

CARDIOVASCULAR DRUG

REGISTERED WITH FDA

Ibersartan

CARDIOVASCULAR DRUG

REGISTERED WITH FDA

Losartan 50mg

CARDIOVASCULAR DRUG

REGISTERED WITH FDA

Losartan 50mg + HCTZ 12.5mg

CARDIOVASCULAR DRUG

REGISTERED WITH FDA

Losartan 100mg + HCTZ 12.5mg/tabs

CARDIOVASCULAR DRUG

REGISTERED WITH FDA

Sitagliptin 25mg, 50mg, 100mg

DIABETIC DRUG

REGISTERED WITH FDA

Sitagliptin 50mg +

Metformin 850mg

DIABETIC DRUG

REGISTERED WITH FDA

Sitagliptin 50mg +

Metformin 1000mg

DIABETIC DRUG

REGISTERED WITH FDA

Insuline Glargine

DIABETIC DRUG

REGISTERED WITH FDA

c. If possible, I doubt it is, I would be interested in seeing the uptake of the innovator medicines based on the availability of the EML drugs in the facilities. I would bet that uptake of innovators would be strongly influenced by eml drug availability

**Based on interim results, only few patients purchased the innovator drugs, but rather preferred to use NHIS to obtain drugs on the EML. Price, rather than availability of EML drugs influenced uptake of innovator medicines.  This will be described in a future manuscript, as publication of results is not appropriate for a protocol paper.**

d. Another nice feature to have, but probably not possible, would be an estimate of the overhead costs with including these innnovator meds. This does not have to be described here but if estimates could be provided when you publish the paper, that would be great.

**The overhead costs will be described in the economic analysis in future manuscripts.**

e. When you analyze patients with diabetes or hypertension, it would be wise to do separate evaluations for new vs previously diagnosed patients. I would imagine there would be dramatic differences in uptake of medications based on this factor alone

**In the baseline questionnaire, it was asked how long participants have had a diagnosis of hypertension and/or diabetes. Therefore this is being considered as part of the analysis.**

f. Are the innovator medications included only for public sector clients or will the private sector components of these hospitals be stocking the innovator medications as well.

**The innovator medicines are available for all patients that meet inclusion criteria for the study.  Each of the facilities that are involved in this study are public sector facilities.**

g. Another point of curiousity, how do prescribers generally write down medications, do they write generic names or the brand names. How will they do it within this study for the innovator medications.

**Both practices are done by providers.**

h. Page 7, recruitment process and description of physician prescribing-physicians can use their discretion to decide mp or dp, why would they ever select MP? If I was the prescriber, I would always use DP and think of a reason why i would want my patients to pay more. I bet prescribers would get issues if patients started to realize that they were being charged differently based on the prescriber's opinion.

**Upon enrollment, providers used the poverty index or HH income to determine whether patients paid DP or MP.** **This was changed once patients were unable to pay for innovator medicines, as these are not covered by the NHIS.**

i. Can you include the protocols for diabetes and hypertension care so we can see how the eml and innovator meds fit into the protocol? It would be an interesting analysis to see how many prescribers deviate from this.

**The protocols have been included as an annex.**

j. For the study sample size and the primary outcome, I would have preferred a continuous variable rather than the dichotomous one selected but you mention that you will report the continuous variable so it is not a big deal. The reason for this is that I'm not exactly sure how you define control for htn and dm.  (i'm assuming bp <140/90 and Hba1c <7 / fasting glucose <126.) If a patient were to experience a dramatic drop in a1c from 13 to 10 over the period of the study, I would be pretty happy with their progress but they would be concerned as not having achieved control.  

**We will also use continuous variables to analyze disease control as part of methods used for longitudinal analysis of the study results.**

k. For adherence, I would also recommend tracking refill history to complement questionnaire.

**As the study has been completed, we are unable to do this.**

l. Is it expected that patients will purchase two months of medications since their visits are every two months? That sometimes becomes too expensive.

**Outside of the study, patients routinely return every 3 months and are typically allowed to purchase a one month supply of drugs and obtain drugs before their next appointment. The study actually increased the frequency of visits to two months rather than spacing the visits out more.**
